# Supplementary material for: Social interactions between people of same and different generations shape longitudinal changes in interpersonal neural synchrony, loneliness, and social connection
Source: PLoS Biol. 2026 Aug 20;24(8):e3003899. doi: 10.1371/journal.pbio.3003899 (PMC13492753; doi:10.1371/journal.pbio.3003899)
Supplement: Supplementary file 1 [file pbio.3003899.s001.pdf]

Supplementary Materials: Moffat, Dumas and Cross (2026). *Social interactions between people of same and different generations shape longitudinal changes in interpersonal neural synchrony, loneliness, and social connection.*

Item	Pages
S1 Appendix (HbR Results)	2-3
S2 Appendix (Supp. methods figures)	
Fig A	4
Fig B	4
Fig C	5
Fig D	5
Fig E	6
Fig F	7
Fig G	7
Fig H	8
S3 Appendix (Supp. results figures)	
Fig A	9
Fig B	9
Fig C	10
Fig D	11
Fig E	12-13
S4 Appendix (Supp. methods tables)	
Table A	14
Table B	15
Table C	16
Table D	16
S5 Appendix (HbO Supp. results tables)	
Table A	17-18
Table B	19
Table C	20-21
Table D	22
Table E	23
Table F	24
Table G	25
Table H	26
Table I	27-28
Table J	29-30
Table K	31-32
Table L	33-34
Table M	35-36
Table N	37-38
Table O	39-40
Table P	41-42
Table Q	43-44
Table R	45-46
Table S	47-48
Table T	49-50
Table U	51-52
Table V	53-54
S6 Appendix (HbO Supp. results tables)	
Table A	55-56
Table B	57
Table C	58-59
Table D	60
Table E	61
Table F	62
Table G	63
Table H	64-65
Table I	66-67
Table J	68-69
Table K	70-71
Table L	72-73
Table M	74-75
Table N	76-77
Table O	78-79
Table P	80-81
Table Q	82-83
Table R	84-85
Table S	86-87
Table T	88-89
Table U	90-91

Supplementary Materials: Moffat, Dumas and Cross (2026). *Social interactions between people of same and different generations shape longitudinal changes in interpersonal neural synchrony, loneliness, and social connection.*

S1 Appendix – Analyses of INS in HbR

1. Real dyad vs. pseudo dyads

INS levels for all sessions combined. We found that all differences corresponded to greater INS for real than pseudo dyads (black squares in Fig C in S3 Appendix [panel A]; solid lines in Fig C in S3 Appendix [panel B]). The ROI pairs showing differences were no ROI pairs for intergenerational dyads drawing alone, LTPJ~LTPJ, LIFG~RIFG, and LIFG~LTPJ for intergenerational dyads drawing together, no ROI pairs for same-generation dyads drawing alone, and RIFG~RIFG and RIFG~LTPJ for same-generation dyads drawing together. Estimates and contrasts with HPD in Tables A-B in S6 Appendix.

Change in INS levels across sessions. We observed a more positive slope of change for two ROI pairs that differed between real and pseudo pairs, and a differing negative slope of change for one ROI pair (black squares in Fig D in S3 Appendix [panel A]; solid lines in Fig D in S3 Appendix [panel B]). The ROI pairs showing differences were RTPJ~RTPJ and RIFG~RTPJ for intergenerational dyads drawing alone, LIFG~LIFG for intergenerational dyads drawing together, no ROI pairs for same-generation dyads drawing alone or together. Estimates and contrasts with HPD in Tables C-D in S6 Appendix.

2. Composition of non-homologous ROI pairs in intergenerational dyads

INS levels for all sessions combined. For intergenerational dyads drawing together, we observed a trend of greater INS when the LIFG belonged to the older adult and the LTPJ belonged to the younger adult than vice versa (95% HPD include 0, 7% of HPD < 0). A pattern was observed where INS was greater when the RIFG belonged to the older adult and the LTPJ belonged to the younger adult than vice versa. **Change in INS levels across sessions.** No differences in the INS slopes across sessions for any composition of any ROI pair. A full list of point estimates and 95% HPDs from contrasts is presented in Table E in S6 Appendix.

3. Contrasting drawing conditions and groups

INS levels for all sessions combined. We observed no differences between groups but did observe differences between tasks. We observed greater INS for drawing together than alone in LIFG~RIFG and RIFG~LTPJ. We also found an interaction between group and task. In LIFG~RIFG, there was a task difference for intergenerational, but not same-generation, dyads (see Supplementary Results Figure 3C). A full list of point estimates and 95% HPDs from contrasts is presented in Table F in S6 Appendix.

Change in INS levels across sessions. We observed no differences between groups or tasks but did observe interactions between group and task. Intergenerational dyads showed a more positive INS slope for drawing alone than together in RTPJ~RTPJ and RIFG~RTPJ while same generation dyads did not. In RTPJ~RPTJ, we also observed a more positive slope for intergenerational than same generation dyads when drawing alone (see Fig D in S3 Appendix [panel C]). A full list of point estimates and 95% HPDs from contrasts is presented in Tables G-I in S6 Appendix.

4. Relationship between INS and self-report measures

Loneliness. *Summed.* We observed an association, whereby greater loneliness predicted lower INS in LIFG~RIFG, as well as a matching group difference for same generation dyads, and a matching task

difference for drawing alone (Fig E in S3 Appendix; Tables J-K in S6 Appendix). No interactions between group and task were observed. *Difference.* We found an association, whereby greater loneliness predicted lower INS in LTPJ~LTPJ, as well as a matching group difference for intergenerational dyads, and matching task difference for drawing together. RIFG~RTPJ showed a task difference, whereby the INS~loneliness relationship was more positive for drawing alone than together (Fig E in S3 Appendix; Tables L-M in S6 Appendix). No interactions between group and task were observed.

Social closeness (OIS). *Summed*. We did not observe an association between INS and summed social closeness scores. Neither group nor task influenced this relationship. We observed an interaction in RIFG~RTPJ, where intergenerational dyads showed a more positive INS~social closeness relationship than same generation dyads when drawing together. In LTPJ~LPTJ, intergenerational dyads also showed a more negative INS~social closeness relationship than same generation dyads when drawing alone (Fig E in S3 Appendix; Tables N-O in S6 Appendix). *Difference.* We did not observe an association between INS and social closeness difference scores. We found a group difference, whereby intergenerational dyads showed a positive relationship in RIFG~RIFG. We also observed a task difference, whereby the relationship in LIFG~RIFG differed between drawing together and alone. We also observed interactions between group and task. In RTPJ~RTPJ, intergenerational dyads showed a more negative slope than same generation dyads while drawing together. In RIFG~LTPJ, intergenerational dyads showed a more positive slope than same generation dyads when drawing together (Fig E in S3 Appendix; Tables P-Q in S6 Appendix).

Allophilia. *Summed*. We did not observe an association between INS and summed allophilia scores. Group did not influence this relationship. We observed a task difference in RTPJ~RTPJ, with a more positive slope for drawing together than drawing alone. We also observed interactions between group and task. In LTPJ~LPTJ, we found a more positive relationship for drawing alone than together for intergenerational dyads. In RTPJ~RTPJ, we observed a more positive relationship for drawing together than alone for same generation dyads (Fig E in S3 Appendix; Tables R-S in S6 Appendix). *Difference*. We did not observe an association between INS and allophilia difference scores. A group difference was found in LTPJ~LTPJ, where intergenerational dyads showed a more positive relationship, that differed from same generation dyads. A task difference was observed, where the INS~allophilia (dif) relationship was more positive for drawing together than alone in LIFG~RIFG. Interactions between group and task were observed in RIFG~RIFG, LTPJ~LTPJ, and RPTJ~RTPJ. For RIFG~RIFG, same generation dyads showed a more positive relationship relative to intergenerational dyads while drawing alone. For LTPJ~LTPJ and RTPJ~RTPJ, intergenerational dyads showed a more positive relationship than same generation dyads while drawing together (Fig E in S3 Appendix; Tables T-U in S6 Appendix).

Supplementary Materials: Moffat, Dumas and Cross (2026). *Social interactions between people of same and different generations shape longitudinal changes in interpersonal neural synchrony, loneliness, and social connection.*

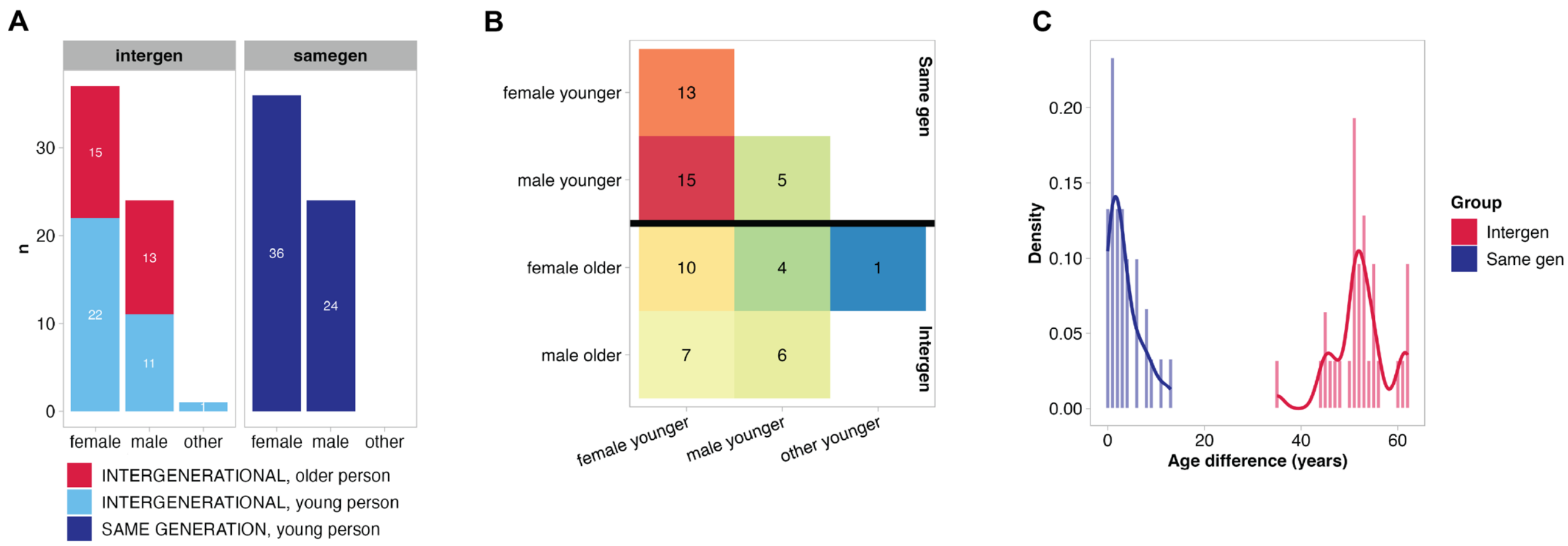

S2 Appendix – Fig A. A) Gender distribution per group. B) Gender/age pairings per group. C) Age difference with dyads, per group.

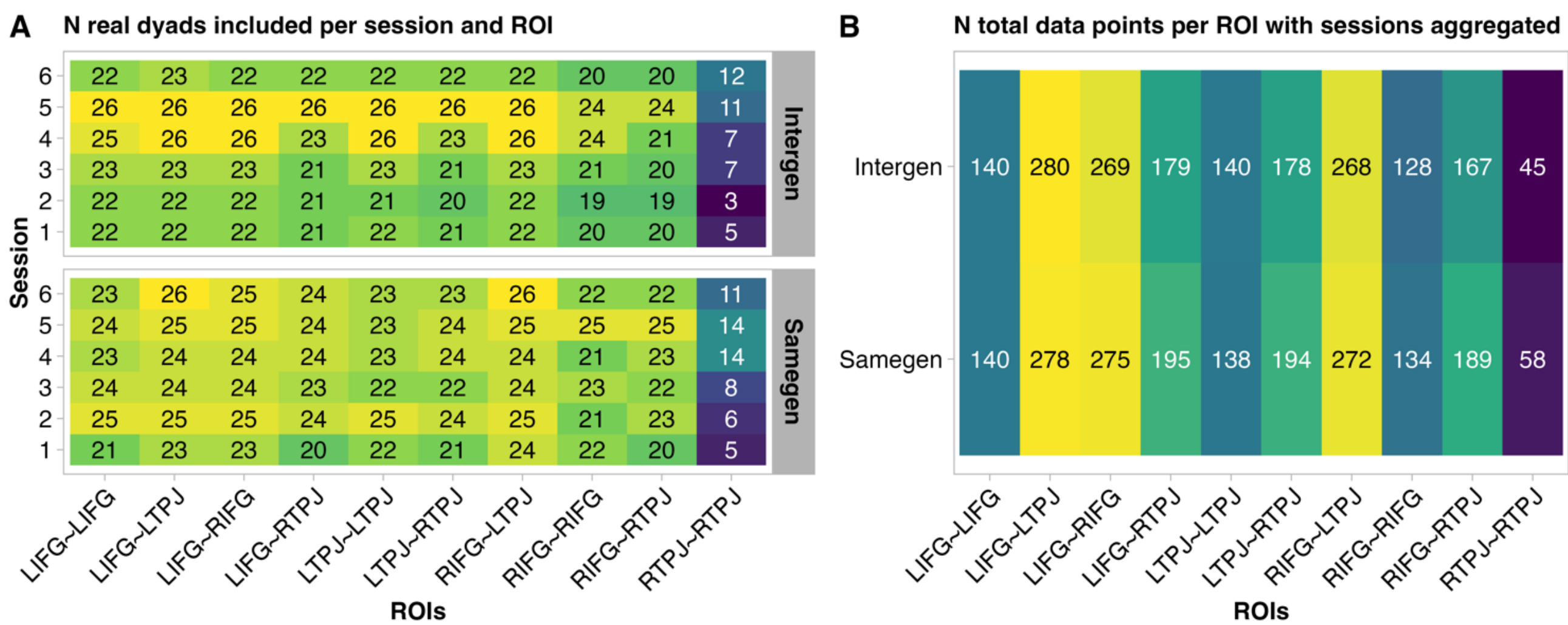

S2 Appendix – Fig B. A) Number of real dyads per ROI pair per session. B) Sum number of real dyads aggregated across sessions. In both A and B, the lower numbers for ROI pairs including RTPJ are the result of a period of data collection during which RTPJ optodes on one fNIRS device were broken. Data collection was not paused during this time, as not to interfere with the 6-session experimental design. New optodes were promptly ordered and installed.

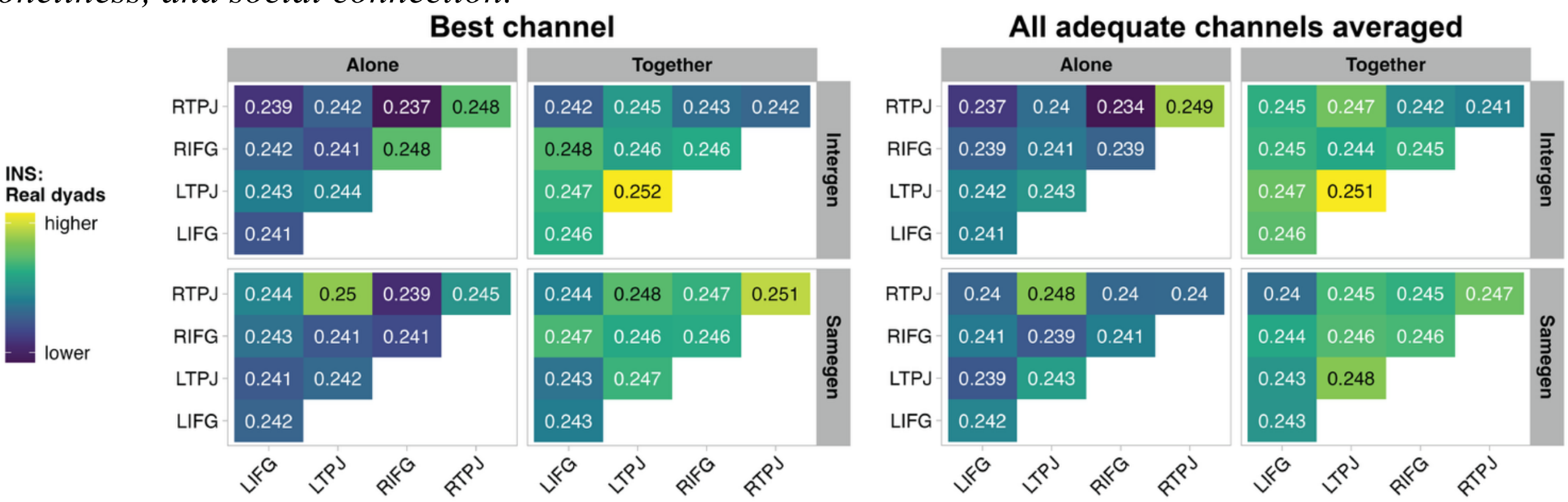

S2 Appendix – Fig C. Comparison of INS point estimates using the best channel (left) and the averaged INS values for all channels in each ROI (right). The best channels were selected as described in the Methods. Adequate channels had visual evidence of heart beats, channel lengths between 20 and 40 mm, and scalp-coupling indices < .7. Visual inspection reveals INS levels to be numerically similar, and the overall pattern of levels to be similar. For example, in both RIFG~RTPJ Intergen Alone is lowest value and LTPJ~LTPJ Intergen Together is highest value.

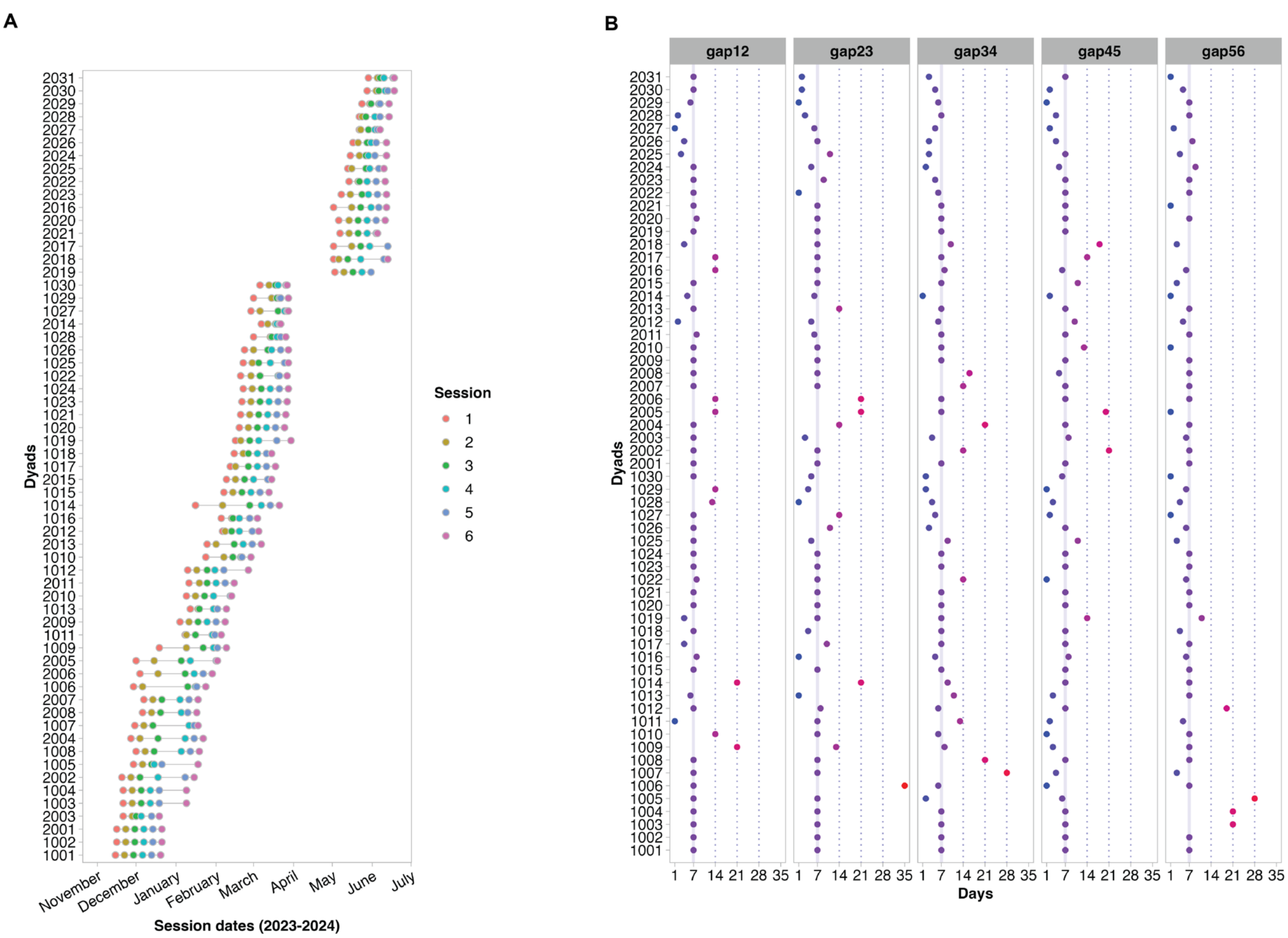

S2 Appendix – Fig D. A) Schedule of sessions per dyad. B) Number of days between sessions for each dyad and gap between sessions. The label *gap12* refers to the number of days between session 1 and session 2, *gap23* to the number of days between session 2 and session 3, and so on.

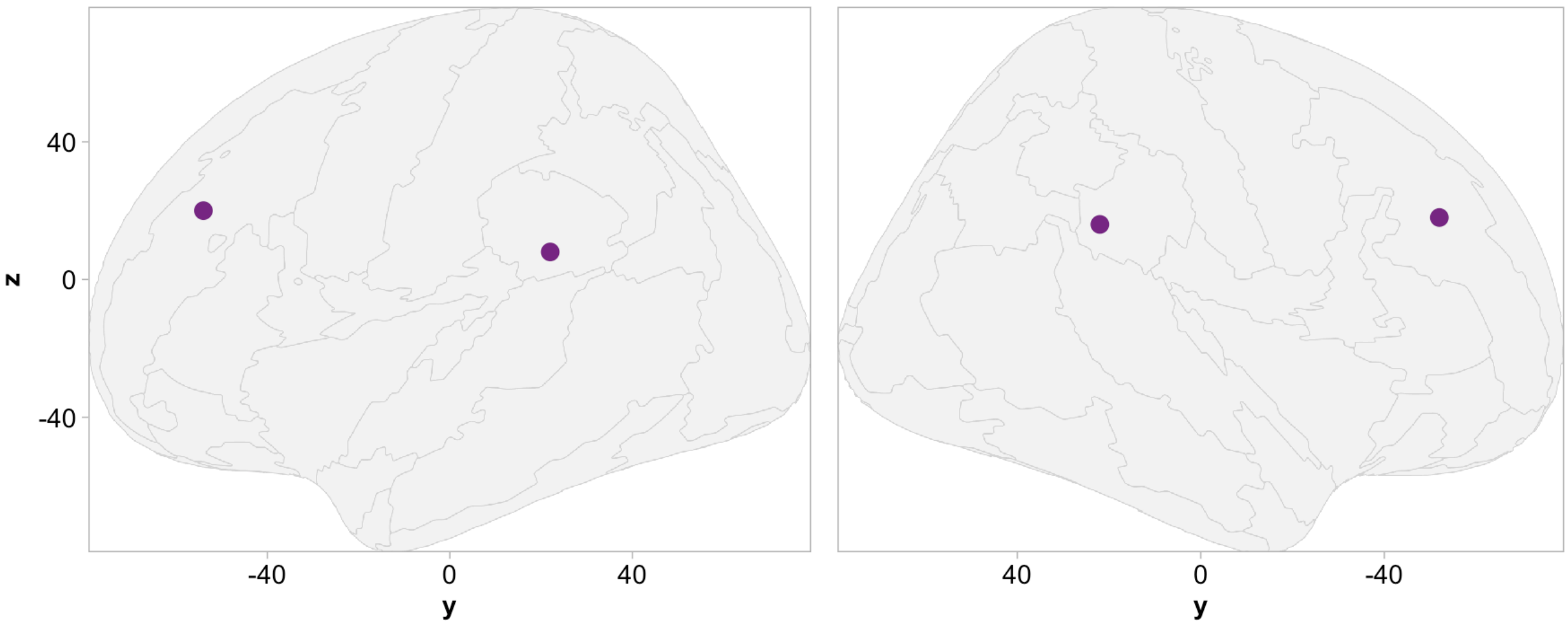

S2 Appendix – Fig E. Purple circles show centre of each ROI in MNI coordinates.

Supplementary Materials: Moffat, Dumas and Cross (2026). *Social interactions between people of same and different generations shape longitudinal changes in interpersonal neural synchrony, loneliness, and social connection.*

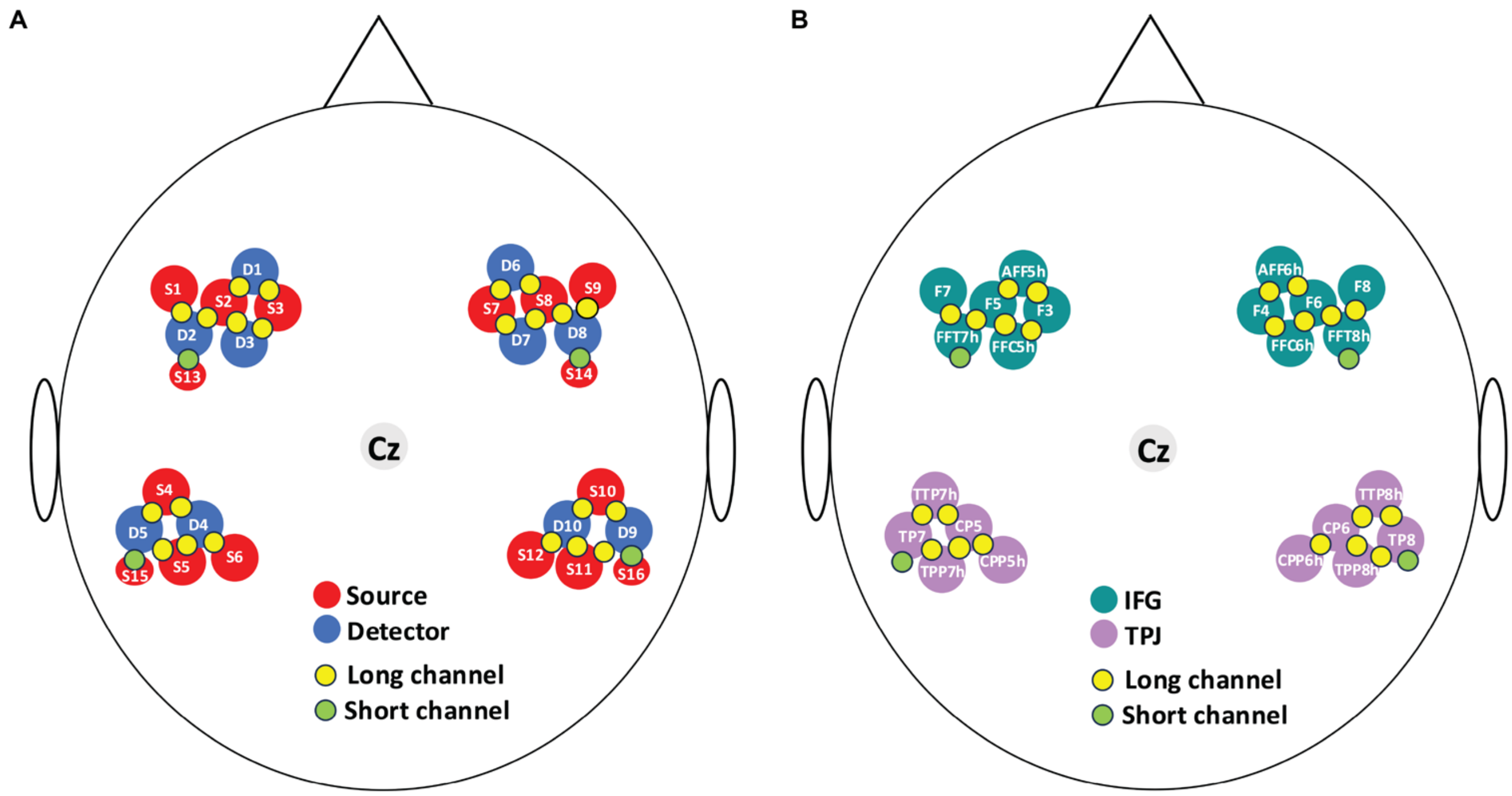

S2 Appendix – Fig F. A) Arrangement optodes, where numbers correspond to sensor numbers on Cortivision Photon Cap Device (S13-S16 are sources specifically designed to measure short channel signals). B) EEG 5-10 positions of each optode within the four ROIs (bilateral IFG and TPJ).

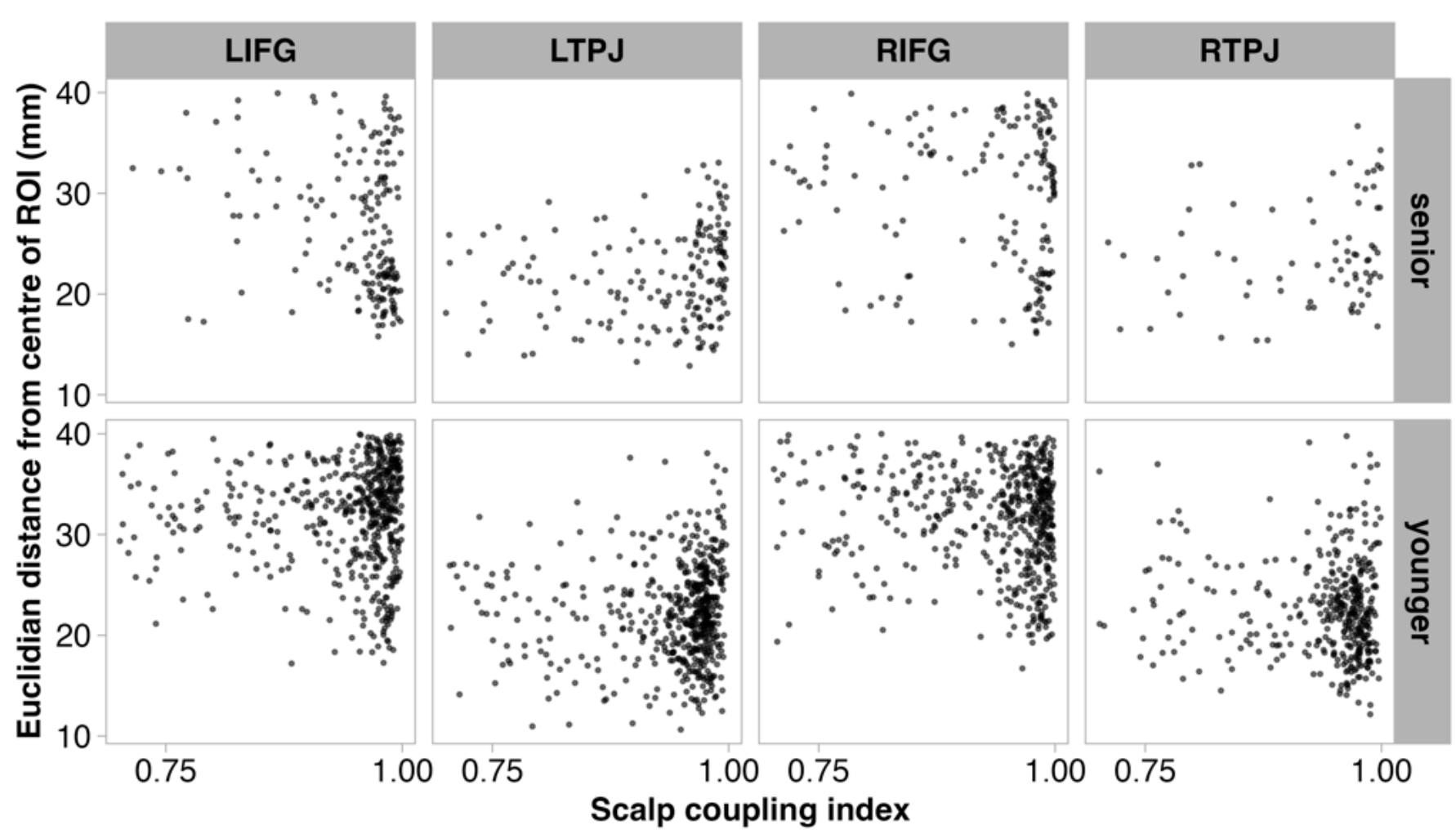

S2 Appendix – Fig G. Distances of selected channels from the coordinates in the centre of each ROI. One component of the distance (i.e., ~15mm) is the distance from the centre of the ROI being inside the cortex and the channel positions being on the scalp. Seniors = participants aged 69+ years. Younger = participants aged 18-35 years.

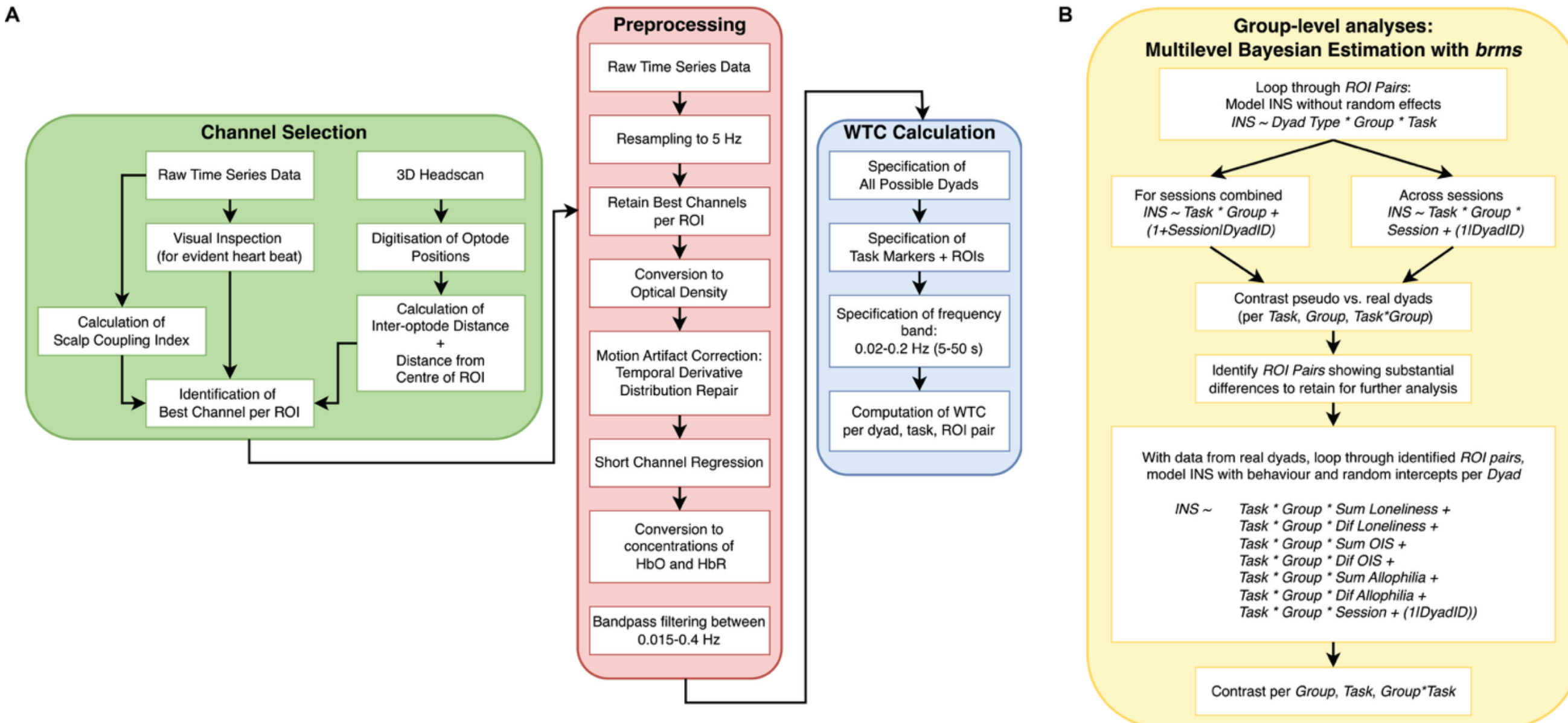

S2 Appendix – Fig H. A) Flowchart visualising the procedure for selecting which channels to include in analysis for individual participants, preprocessing steps for individual participants and calculation of WTC for dyads. B) Flowchart visualising the order in which group-level analyses were performed in the R package brms, and showing the models fit.

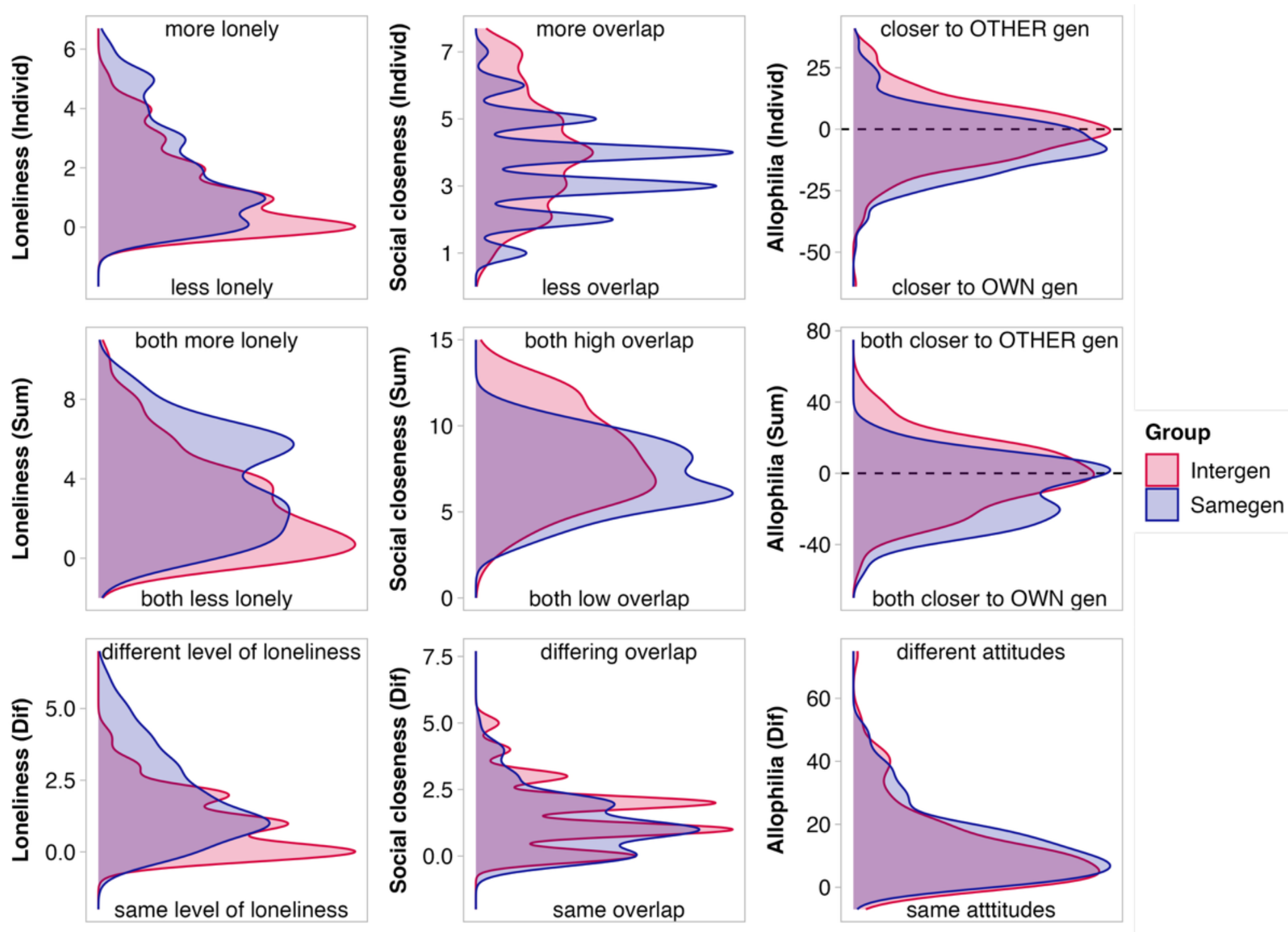

S3 Appendix – Fig A. Top: Distributions of self-reported loneliness, social closeness (OIS), and attitudes toward generations (allophilia) of individual participants. Middle: Distribution of summed scores per dyad. Bottom: Distribution of difference scores per dyads.

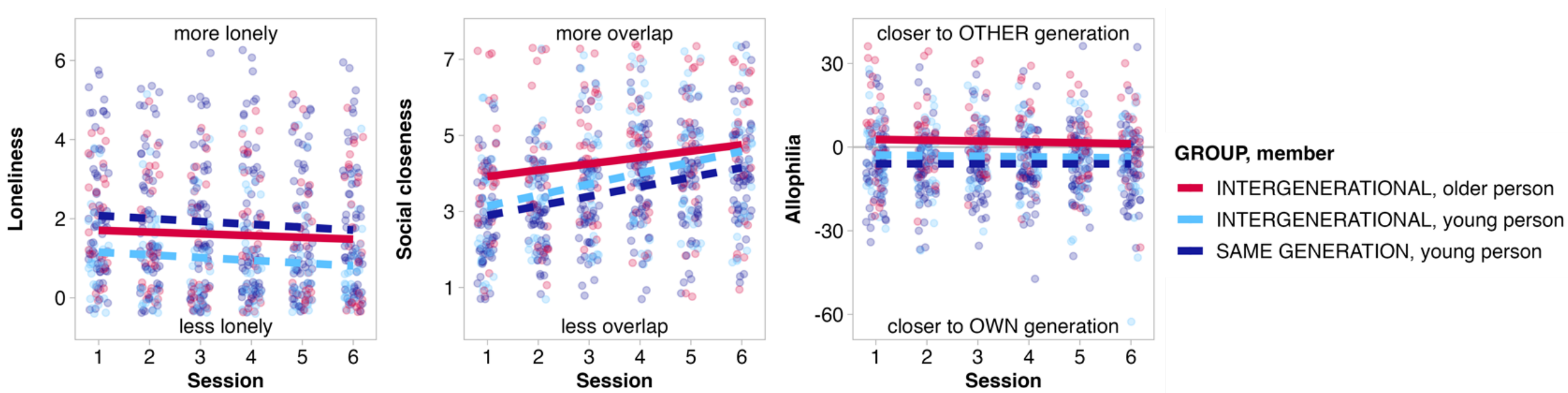

S3 Appendix – Fig B. Self-report scores for loneliness, social closeness, and attitudes towards generations (i.e., allophilia) plotted with younger and older members of intergenerational dyads separated.

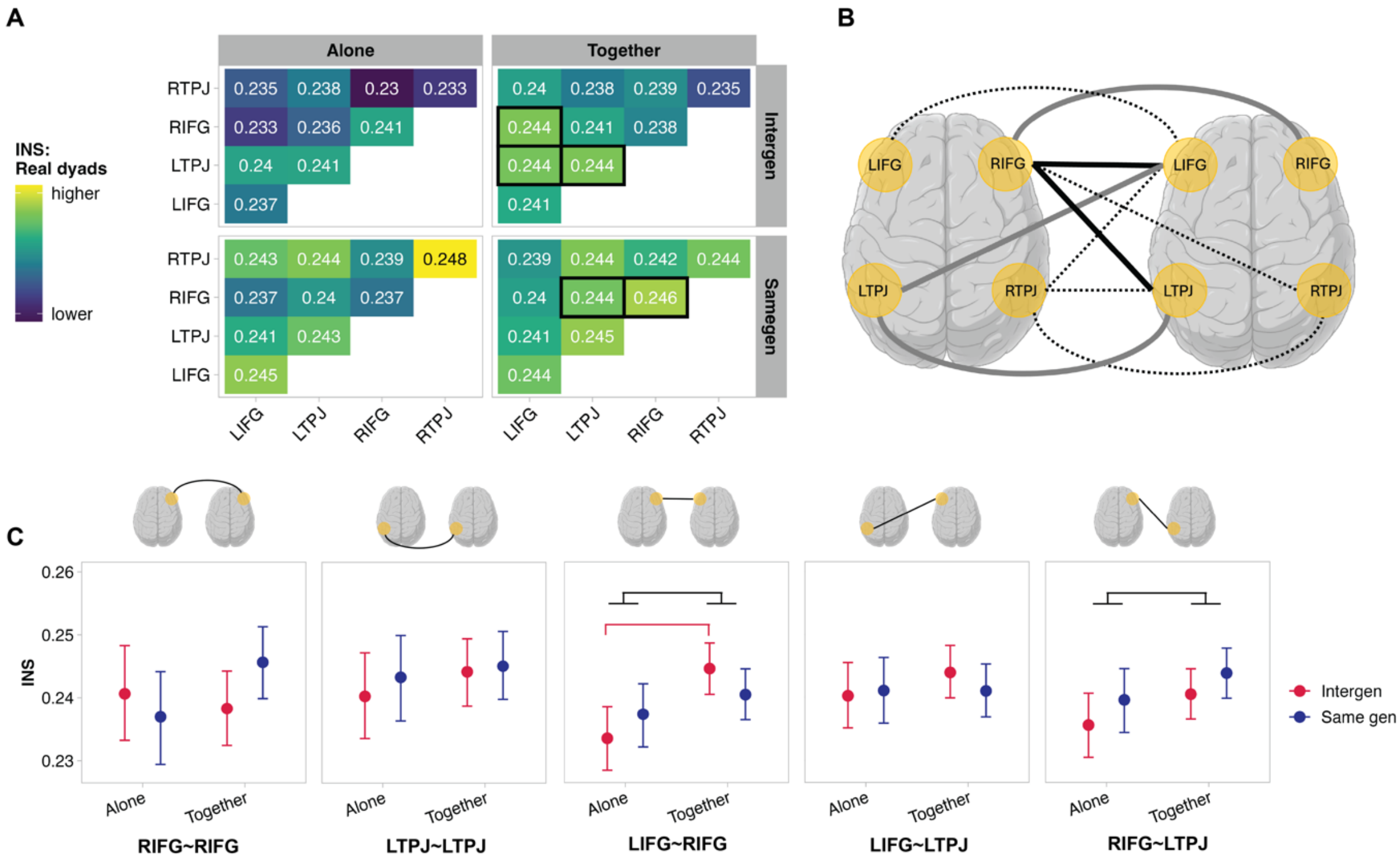

S3 Appendix – Fig C. HbR. INS for all ROI pairs per task and group; black squares indicate that INS levels differ between real and pseudo dyads (i.e., 95% HPD of contrast distribution excludes 0). B) Visualisation of all possible ROI pairs (n=10). Dashed lines indicate ROI pairs where INS did not differ between real and pseudo dyads. Solid lines indicate ROI pairs where INS differed between real and pseudo dyads. Black solid lines indicate ROI pairs where task, group, and task*group contrasts showed differences. Grey solid lines indicate ROI pairs where contrasts showed no differences for task, group, or task*group contrasts. C) INS levels as point estimates with 95% HPD for all ROI pairs that differed between real and pseudo dyads, for which task, group, and task*group contrasts were computed. RIFG~RIFG: No differences. LTPJ~LTPJ: No differences. LIFG~RIFG: Task difference, together > alone. Interaction, together > alone for intergenerational group. LIFG~LTPJ: No differences. RIFG~LTPJ: Task difference, together > alone. Full list of point estimates from models and contrasts with 95% HPDs available in Tables A-B in S6 Appendix. Brains created in BioRender. Moffat, R. (2026) https://BioRender.com/6p18lgg. A and C were created using code shared in the OSF repository "Longitudinal perspectives on intergenerational inter-brain synchrony": https://osf.io/xcgp6. Code for A: https://osf.io/xcgp6/files/pu7h2. Code to generate individual values, compute summary values, and generate each panel in: https://osf.io/xcgp6/files/4f8qw. Table of summary values shown in C: https://osf.io/xcgp6/files/4vynd.

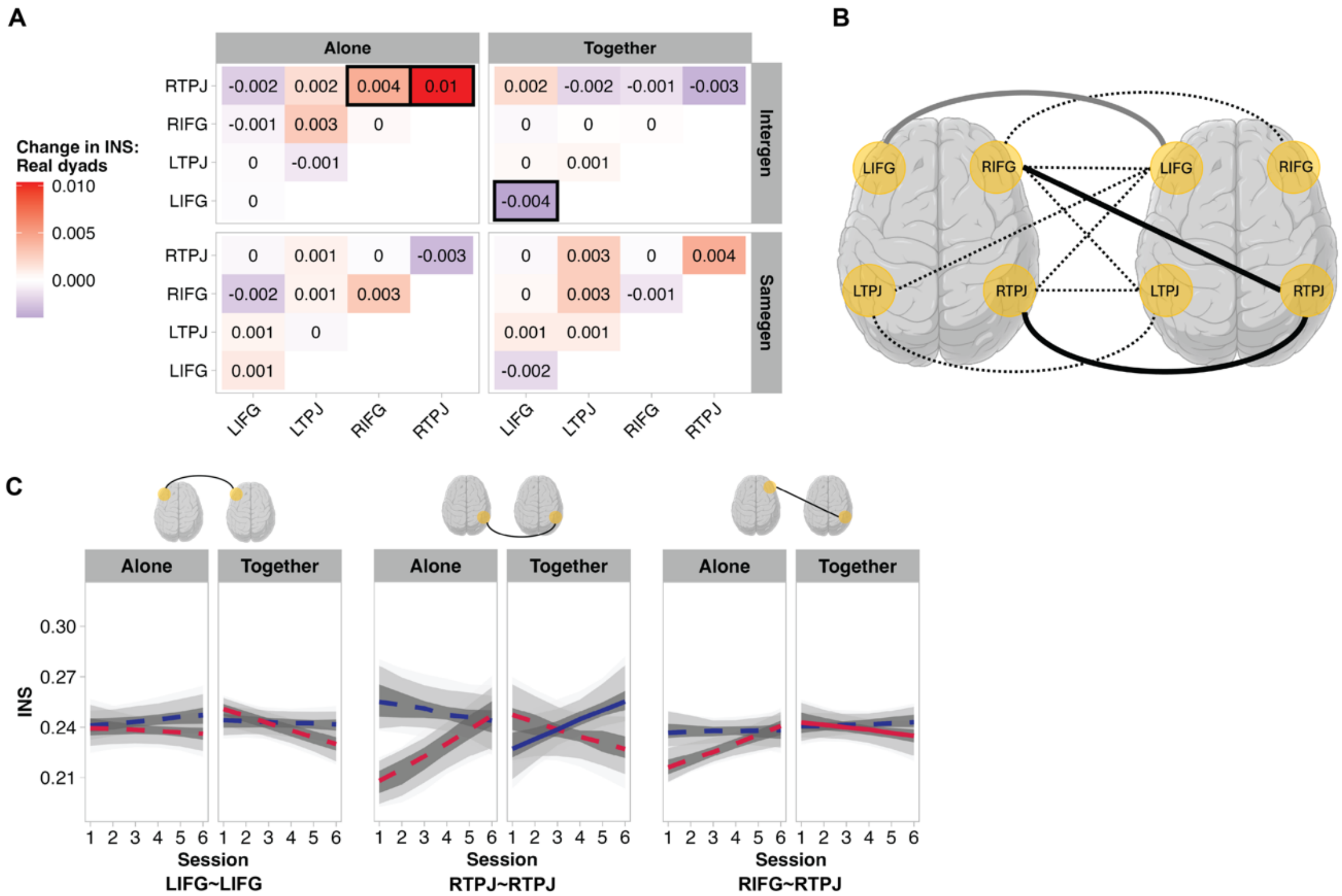

S3 Appendix – Fig D. HbR. A) Slope of change in INS across six sessions; black squares indicate that INS levels differ between real and pseudo dyads (i.e., 95% HPD of contrast distribution excludes 0). B) Visualisation of all possible ROI pairs (n=10). Dashed lines indicate ROI pairs where the slope of change in INS did not differ between real and pseudo dyads. Solid lines indicate ROI pairs where the slope of change in INS differed between real and pseudo dyads. Black solid lines indicate ROI pairs where task, group, and task*group contrasts showed differences. C) Slope of change in INS across sessions for all ROI pairs that differed between real and pseudo dyads, for which task, group, and task*group contrasts were computed. Solid lines indicate that the 95% HPD of an INS~Session slope excludes 0, indicating an association. Dashed lines indicate that the 95% HPD of an INS~Session slope includes 0, indicating no association. Grey shading shows 95%, 89%, and 50% intervals of the posterior predictive distributions. LIFG~LIFG: No differences. RTPJ~RTPJ: Interactions, together < alone for intergenerational group and intergenerational > same generation for alone. RIFG~RTPJ: Interaction, together < alone for intergenerational group. Full list of point estimates from models and contrasts with 95% HPDs available in Tables C-D in S6 Appendix. Brains created in BioRender. Moffat, R. (2026) https://BioRender.com/6p18lgg. A and C were created using code shared in the OSF repository “Longitudinal perspectives on intergenerational inter-brain synchrony”: https://osf.io/xcgp6. Code for A: https://osf.io/xcgp6/files/pu7h2. Code for C: https://osf.io/xcgp6/files/4f8qw.

Supplementary Materials: Moffat, Dumas and Cross (2026). *Social interactions between people of same and different generations shape longitudinal changes in interpersonal neural synchrony, loneliness, and social connection.*

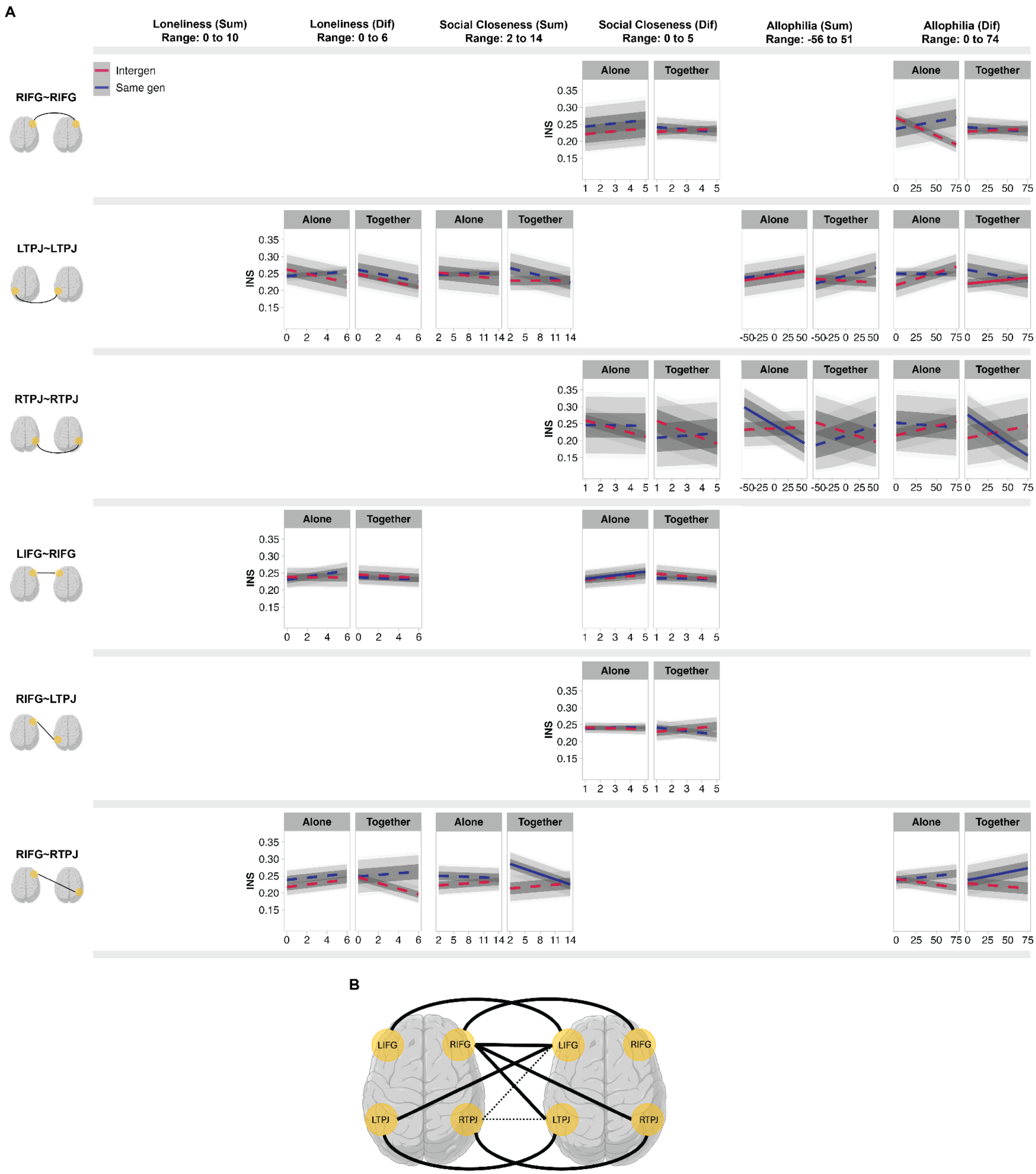

S3 Appendix – Fig E. HbR. A) Slope of change in INS across levels of self-report measures for ROI pairs with at least one real vs. pseudo difference revealed by contrasts. Lines indicate whether the 95% HPD for each INS~Self-report measure slope excludes 0 (solid), indicating an association, or includes 0 (dashed), indicating no association. Shading shows 95%, 89%, and 50% intervals for the posterior predictive distributions. Results of task, group, and task*group contrasts for the panels above are as follows. *RIFG~RIFG Social Closeness (Sum):* Group difference, positive relationship for intergenerational dyads only. *RIFG~RIFG Allophilia (Dif):* Interaction, intergenerational < same generation dyads for drawing alone. *LTPJ~LTPJ Loneliness (Dif):* Negative relationship. Group difference, negative relationship remains for intergenerational dyads only. Task difference, negative relationship remains for drawing together only. *LTPJ~LTPJ Social Closeness (Sum):* Interaction difference, intergenerational < same generation dyads for drawing alone. *LTPJ~LTPJ Allophilia (Sum):* Interaction, drawing alone > together for intergenerational dyads. *LTPJ~LTPJ Allophilia (Dif):* Group difference, negative relationship for intergenerational dyads and intergenerational > same generation dyads. Interaction, intergenerational > same generation dyads for drawing together. *RTPJ~RTPJ Social Closeness (Dif):* Interaction, intergenerational < same generation dyads for drawing together. *RTPJ~RTPJ Allophilia (Sum):* Task difference, drawing alone < together. Interaction, drawing alone < together for same generation dyads. *RTPJ~RTPJ Allophilia (Dif):* Interaction, intergenerational > same generation dyads for drawing together. *LIFG~RIFG Loneliness (Dif):* Negative relationship. Group difference, negative relationship for same generation dyads. Task difference, negative relationship for drawing alone. *LIFG~RIFG Social Closeness (Dif):* Task difference, positive relationship for drawing alone and drawing alone > together. *RIFG~LTPJ Social Closeness (Dif):* Interaction, intergenerational > same generation dyads for drawing together. *RIFG~RTPJ Loneliness (Dif):* Task difference, drawing alone > together. *RIFG~RTPJ Social Closeness (Sum):* Group difference, intergenerational > same generation dyads. Interaction, intergenerational > same generation dyads for drawing together. *RIFG~RTPJ Allophilia (Dif):* No differences. B) Visualisation of all possible ROI pairs (n=10). Dashed lines indicate ROI pairs where i) neither INS for all sessions combined nor ii) the slope of change in INS differed between real and pseudo dyads. Solid lines indicate ROI pairs where INS (for sessions combined or slope of change across sessions) differed between real and pseudo dyads. Black solid lines indicate ROI pairs where task, group, and task*group contrasts showed differences. Brains created in BioRender. Moffat, R. (2026) https://BioRender.com/6p18lgg. This figure was created using code shared in the OSF repository "Longitudinal perspectives on intergenerational inter-brain synchrony": https://osf.io/xcgp6. Code for individual panels in this figure: https://osf.io/xcgp6/files/ptyhe.

S4 Appendix – Table A. Comparison of preregistered predictions/analysis plan and the analyses undertaken to date. Preregistration is available on the Open Science Framework (https://osf.io/hz6tm). In this preregistration, we stated our plan to disseminate our findings across multiple publications.

#	Prediction	Status of analysis
1.	We predict that IOS and allophilia will increase across sessions, and that loneliness will decrease (simple terms). These effects may be more pronounced in the intergenerational group (2-way interaction).	Presented in Results of main manuscript.
2.	We predict that the degree of synchrony in the dyad's drawing-hand movements and the dyad's head movements will increase across sessions for joint drawing, but not solo drawing (2-way interaction). This effect may be stronger for intergenerational pairs than same generation pairs (3-way interaction).	Movement data have not yet been analysed. Analysis not yet performed.
3.	We predict that the degree of synchrony in the dyad's drawing-hand movements and the dyad's head movements [while drawing together] will be positively associated with the self-in-other scores, in a way that does not change across sessions (2-way interaction). This effect may be stronger for intergenerational pairs than same generation pairs (3-way interaction).	Movement data have not yet been analysed. Analysis not yet performed.
4.	We predict congruent increases in INS and movement synchrony for drawing together, relative to alone (2-way interaction). We will examine all ROI pairs, have not formed strong predictions (3-way interaction). These effects may grow stronger across sessions and/or differ between groups (4-/5-way interaction).	Movement data have not yet been analysed. Analysis not yet performed.
5.	We predict that INS during join drawing will be associated with IOS, allophilia, and loneliness (2-way interactions). These will likely become stronger across sessions and/or differ between groups (3-/4-way interactions)	Relationships between self-report measures and INS reported in Results of main manuscript (3-way interaction only). We did not pursue the 4-way interaction as we realized that the interpretability of these results would be low, particularly without directional hypotheses.
6.	We predict that INS during join drawing will be associated with ratings of coherence and temporal overlap during drawing (2-way interactions). These effects will likely become stronger across sessions and/or differ between groups (3-/4-way interactions).	During data collection, the research team discussed switching from subjective experimenter ratings of drawing coherence to ratings from a less partial population with minimal knowledge of the study, as well as objective kinematic measures of temporal overlap. Ratings of the drawing coherence (called 'collaboration visible in drawings') have since been collected (Moffat et al., 2026). Hand overlap can be extracted from movement coordinates in future analyses.

S4 Appendix – Table B. Breakdown of reasons for excluding dyads data. Recordings/dyads only included once in list. E.g., if a recording/dyad is counted among reasons unrelated to signal quality, they are not re-counted among reasons related to signal quality. In total 91 recordings of 732 recordings were excluded, impacting 85 of 366 dyadic sessions.

Reason	N individual recordings excluded	N dyadic sessions impacted
Unrelated to signal quality		
Recordings lost in transition between recording laptops	2	2
Triggers missing or unreliable	7	4
Participant unable to complete session (could not attend final session/consumed alcohol)	5	3
Multi-part recording (bluetooth or battery dropout)	6	4
Related to signal quality		
No short channels of adequate quality	71	69
No long channels of adequate quality (pilot montage used)	0	0
**Total**	**91**	**85**

S4 Appendix – Table C. Number of pseudo dyads per session, group and ROI.

ROIs	Group	Session 1	Session 2	Session 3	Session 4	Session 5	Session 6
LIFG~RIFG	Intergen	3386	3633	3980	4294	4323	3455
LIFG~RIFG	Samegen	5434	6268	6354	6120	6676	6198
LTPJ~RTPJ	Intergen	1679	1892	2437	2868	3193	2478
LTPJ~RTPJ	Samegen	3800	4726	4474	5003	5472	5024
LIFG~LIFG	Intergen	1754	1970	2083	2215	2188	1818
LIFG~LIFG	Samegen	2680	3378	3216	3137	3297	3137
LTPJ~LTPJ	Intergen	1726	1888	1977	2242	2188	1818
LTPJ~LTPJ	Samegen	2828	3378	3138	3217	3298	3137
LIFG~LTPJ	Intergen	3482	3858	4059	4457	4376	3636
LIFG~LTPJ	Samegen	5508	6756	6355	6355	6597	6277
LIFG~RTPJ	Intergen	1716	1949	2507	2854	3193	2478
LIFG~RTPJ	Samegen	3699	4726	4530	4941	5471	5025
RIFG~RIFG	Intergen	1630	1675	1899	2082	2134	1639
RIFG~RIFG	Samegen	2753	2905	3137	2982	3378	3059
RTPJ~RTPJ	Intergen	352	403	734	875	1145	820
RTPJ~RTPJ	Samegen	1270	1647	1588	1939	2264	2005
RIFG~LTPJ	Intergen	3355	3557	3876	4321	4323	3455
RIFG~LTPJ	Samegen	5583	6268	6277	6197	6677	6199
RIFG~RTPJ	Intergen	1622	1794	2382	2765	3144	2339
RIFG~RTPJ	Samegen	3750	4384	4473	4818	5537	4961

S4 Appendix – Table D. The weakly informed priors used to impose a constrained distribution on expected results.

Outcome measure	Numeric range	Syntax for priors
INS<br>Real and pseudo dyads	0 to 1	weakly_informative_priors <- c(<br>set_prior('normal(0, 1)', class = 'Intercept'),<br>set_prior('normal(0, .5)', class = 'b'))
INS<br>Real dyads only	0 to 1	weakly_informative_priors <- c(<br>set_prior('normal(0, 1)', class = 'Intercept'),<br>set_prior('normal(0, .5)', class = 'b'),<br>set_prior('normal(0, .01)', class = 'sd'))
Loneliness	0 to 6	weakly_informative_priors <- c(<br>set_prior('normal(3, 1.5)', class = 'Intercept'),<br>set_prior('normal(0, 1)', class = 'b'),<br>set_prior('normal(0, .1)', class = 'sd'))
Social closeness	1 to 7	weakly_informative_priors <- c(<br>set_prior('normal(3, 5)', class = 'Intercept'),<br>set_prior('normal(0, 5)', class = 'b'),<br>set_prior('normal(0, 1)', class = 'sd'))
Allophilia	- 85 to 85	weakly_informative_priors <- c(<br>set_prior('normal(0, 10)', class = 'Intercept'),<br>set_prior('normal(0, 5)', class = 'b'),<br>set_prior('normal(0, 1)', class = 'sd'))

Supplementary Materials: Moffat, Dumas and Cross (2026). *Social interactions between people of same and different generations shape longitudinal changes in interpersonal neural synchrony, loneliness, and social connection.*

S5 Appendix – Table A. HbO. Estimates of INS levels for all sessions combined, for real and pseudo dyads per ROI pair and task in unstandardised units.

Dyad Type	Group	ROI Pair	Task	Estimate	Lower HPD	Upper HPD
Pseudo	Intergen	LIFG~RIFG	Alone	0.24	0.239	0.24
Real	Intergen	LIFG~RIFG	Alone	0.242	0.237	0.247
Pseudo	Intergen	LIFG~RIFG	Together	0.241	0.24	0.241
Real	Intergen	LIFG~RIFG	Together	0.248	0.244	0.251
Pseudo	Intergen	LTPJ~RTPJ	Alone	0.24	0.239	0.241
Real	Intergen	LTPJ~RTPJ	Alone	0.242	0.236	0.248
Pseudo	Intergen	LTPJ~RTPJ	Together	0.241	0.241	0.242
Real	Intergen	LTPJ~RTPJ	Together	0.245	0.24	0.25
Pseudo	Intergen	LIFG~LIFG	Alone	0.241	0.24	0.242
Real	Intergen	LIFG~LIFG	Alone	0.241	0.235	0.248
Pseudo	Intergen	LIFG~LIFG	Together	0.241	0.241	0.242
Real	Intergen	LIFG~LIFG	Together	0.246	0.241	0.252
Pseudo	Intergen	LTPJ~LTPJ	Alone	0.242	0.241	0.243
Real	Intergen	LTPJ~LTPJ	Alone	0.244	0.237	0.25
Pseudo	Intergen	LTPJ~LTPJ	Together	0.243	0.243	0.244
Real	Intergen	LTPJ~LTPJ	Together	0.252	0.247	0.257
Pseudo	Intergen	LIFG~LTPJ	Alone	0.24	0.24	0.241
Real	Intergen	LIFG~LTPJ	Alone	0.243	0.239	0.248
Pseudo	Intergen	LIFG~LTPJ	Together	0.241	0.241	0.241
Real	Intergen	LIFG~LTPJ	Together	0.247	0.243	0.25
Pseudo	Intergen	LIFG~RTPJ	Alone	0.238	0.237	0.239
Real	Intergen	LIFG~RTPJ	Alone	0.239	0.233	0.245
Pseudo	Intergen	LIFG~RTPJ	Together	0.24	0.239	0.24
Real	Intergen	LIFG~RTPJ	Together	0.242	0.237	0.246
Pseudo	Intergen	RIFG~RIFG	Alone	0.239	0.238	0.239
Real	Intergen	RIFG~RIFG	Alone	0.248	0.241	0.255
Pseudo	Intergen	RIFG~RIFG	Together	0.24	0.239	0.24
Real	Intergen	RIFG~RIFG	Together	0.246	0.241	0.252
Pseudo	Intergen	RTPJ~RTPJ	Alone	0.238	0.237	0.239
Real	Intergen	RTPJ~RTPJ	Alone	0.248	0.236	0.259
Pseudo	Intergen	RTPJ~RTPJ	Together	0.239	0.238	0.24
Real	Intergen	RTPJ~RTPJ	Together	0.242	0.232	0.252
Pseudo	Intergen	LTPJ~RIFG	Alone	0.239	0.239	0.24
Real	Intergen	LTPJ~RIFG	Alone	0.241	0.236	0.245
Pseudo	Intergen	LTPJ~RIFG	Together	0.241	0.24	0.241
Real	Intergen	LTPJ~RIFG	Together	0.246	0.242	0.249
Pseudo	Intergen	RIFG~RTPJ	Alone	0.238	0.237	0.238
Real	Intergen	RIFG~RTPJ	Alone	0.237	0.231	0.243
Pseudo	Intergen	RIFG~RTPJ	Together	0.238	0.238	0.239
Real	Intergen	RIFG~RTPJ	Together	0.243	0.238	0.248
Pseudo	Samegen	LIFG~RIFG	Alone	0.242	0.241	0.242
Real	Samegen	LIFG~RIFG	Alone	0.243	0.238	0.247
Pseudo	Samegen	LIFG~RIFG	Together	0.243	0.243	0.243

Dyad Type	Group	ROI Pair	Task	Estimate	Lower HPD	Upper HPD
Real	Samegen	LIFG~RIFG	Together	0.247	0.244	0.251
Pseudo	Samegen	LTPJ~RTPJ	Alone	0.245	0.244	0.245
Real	Samegen	LTPJ~RTPJ	Alone	0.25	0.244	0.255
Pseudo	Samegen	LTPJ~RTPJ	Together	0.244	0.244	0.244
Real	Samegen	LTPJ~RTPJ	Together	0.248	0.243	0.252
Pseudo	Samegen	LIFG~LIFG	Alone	0.242	0.242	0.243
Real	Samegen	LIFG~LIFG	Alone	0.242	0.235	0.248
Pseudo	Samegen	LIFG~LIFG	Together	0.243	0.242	0.243
Real	Samegen	LIFG~LIFG	Together	0.243	0.238	0.248
Pseudo	Samegen	LTPJ~LTPJ	Alone	0.245	0.244	0.245
Real	Samegen	LTPJ~LTPJ	Alone	0.242	0.235	0.249
Pseudo	Samegen	LTPJ~LTPJ	Together	0.246	0.245	0.246
Real	Samegen	LTPJ~LTPJ	Together	0.247	0.242	0.252
Pseudo	Samegen	LIFG~LTPJ	Alone	0.241	0.241	0.242
Real	Samegen	LIFG~LTPJ	Alone	0.241	0.237	0.246
Pseudo	Samegen	LIFG~LTPJ	Together	0.243	0.242	0.243
Real	Samegen	LIFG~LTPJ	Together	0.243	0.239	0.246
Pseudo	Samegen	LIFG~RTPJ	Alone	0.241	0.241	0.242
Real	Samegen	LIFG~RTPJ	Alone	0.244	0.238	0.249
Pseudo	Samegen	LIFG~RTPJ	Together	0.241	0.241	0.242
Real	Samegen	LIFG~RTPJ	Together	0.244	0.239	0.248
Pseudo	Samegen	RIFG~RIFG	Alone	0.241	0.241	0.242
Real	Samegen	RIFG~RIFG	Alone	0.241	0.234	0.247
Pseudo	Samegen	RIFG~RIFG	Together	0.243	0.242	0.243
Real	Samegen	RIFG~RIFG	Together	0.246	0.241	0.251
Pseudo	Samegen	RTPJ~RTPJ	Alone	0.244	0.243	0.245
Real	Samegen	RTPJ~RTPJ	Alone	0.245	0.235	0.255
Pseudo	Samegen	RTPJ~RTPJ	Together	0.242	0.241	0.243
Real	Samegen	RTPJ~RTPJ	Together	0.251	0.242	0.26
Pseudo	Samegen	LTPJ~RIFG	Alone	0.242	0.241	0.242
Real	Samegen	LTPJ~RIFG	Alone	0.241	0.237	0.246
Pseudo	Samegen	LTPJ~RIFG	Together	0.243	0.243	0.244
Real	Samegen	LTPJ~RIFG	Together	0.246	0.243	0.25
Pseudo	Samegen	RIFG~RTPJ	Alone	0.242	0.241	0.242
Real	Samegen	RIFG~RTPJ	Alone	0.239	0.233	0.244
Pseudo	Samegen	RIFG~RTPJ	Together	0.242	0.242	0.242
Real	Samegen	RIFG~RTPJ	Together	0.247	0.242	0.251

S5 Appendix – Table B. HbO. Contrasts between real and pseudo dyads' INS levels for all sessions combined per ROI pair and task in unstandardised units.

Contrast	Group	ROI Pair	Task	Estimate	Lower HPD	Upper HPD	No overlap
Real - Pseudo	Intergen	LIFG~RIFG	Alone	-0.002	-0.007	0.003	
Real - Pseudo	Intergen	LIFG~RIFG	Together	-0.007	-0.011	-0.003	No overlap
Real - Pseudo	Intergen	LTPJ~RTPJ	Alone	-0.002	-0.008	0.004	
Real - Pseudo	Intergen	LTPJ~RTPJ	Together	-0.004	-0.009	0.001	
Real - Pseudo	Intergen	LIFG~LIFG	Alone	0	-0.007	0.006	
Real - Pseudo	Intergen	LIFG~LIFG	Together	-0.005	-0.01	0	No overlap
Real - Pseudo	Intergen	LTPJ~LTPJ	Alone	-0.002	-0.008	0.005	
Real - Pseudo	Intergen	LTPJ~LTPJ	Together	-0.009	-0.014	-0.004	No overlap
Real - Pseudo	Intergen	LIFG~LTPJ	Alone	-0.003	-0.008	0.001	
Real - Pseudo	Intergen	LIFG~LTPJ	Together	-0.006	-0.009	-0.002	No overlap
Real - Pseudo	Intergen	LIFG~RTPJ	Alone	-0.001	-0.007	0.005	
Real - Pseudo	Intergen	LIFG~RTPJ	Together	-0.002	-0.007	0.003	
Real - Pseudo	Intergen	RIFG~RIFG	Alone	-0.009	-0.016	-0.002	No overlap
Real - Pseudo	Intergen	RIFG~RIFG	Together	-0.007	-0.012	-0.001	No overlap
Real - Pseudo	Intergen	RTPJ~RTPJ	Alone	-0.01	-0.021	0.002	
Real - Pseudo	Intergen	RTPJ~RTPJ	Together	-0.003	-0.013	0.007	
Real - Pseudo	Intergen	LTPJ~RIFG	Alone	-0.002	-0.006	0.003	
Real - Pseudo	Intergen	LTPJ~RIFG	Together	-0.005	-0.009	-0.001	No overlap
Real - Pseudo	Intergen	RIFG~RTPJ	Alone	0	-0.006	0.006	
Real - Pseudo	Intergen	RIFG~RTPJ	Together	-0.004	-0.009	0.001	
Real - Pseudo	Samegen	LIFG~RIFG	Alone	-0.001	-0.006	0.004	
Real - Pseudo	Samegen	LIFG~RIFG	Together	-0.004	-0.008	-0.001	No overlap
Real - Pseudo	Samegen	LTPJ~RTPJ	Alone	-0.005	-0.011	0.001	
Real - Pseudo	Samegen	LTPJ~RTPJ	Together	-0.004	-0.008	0.001	
Real - Pseudo	Samegen	LIFG~LIFG	Alone	0	-0.006	0.007	
Real - Pseudo	Samegen	LIFG~LIFG	Together	-0.001	-0.006	0.005	
Real - Pseudo	Samegen	LTPJ~LTPJ	Alone	0.002	-0.004	0.009	
Real - Pseudo	Samegen	LTPJ~LTPJ	Together	-0.001	-0.006	0.004	
Real - Pseudo	Samegen	LIFG~LTPJ	Alone	0	-0.005	0.005	
Real - Pseudo	Samegen	LIFG~LTPJ	Together	0	-0.004	0.003	
Real - Pseudo	Samegen	LIFG~RTPJ	Alone	-0.003	-0.008	0.003	
Real - Pseudo	Samegen	LIFG~RTPJ	Together	-0.002	-0.007	0.002	
Real - Pseudo	Samegen	RIFG~RIFG	Alone	0.001	-0.006	0.007	
Real - Pseudo	Samegen	RIFG~RIFG	Together	-0.003	-0.009	0.002	
Real - Pseudo	Samegen	RTPJ~RTPJ	Alone	-0.001	-0.011	0.009	
Real - Pseudo	Samegen	RTPJ~RTPJ	Together	-0.009	-0.017	0	
Real - Pseudo	Samegen	LTPJ~RIFG	Alone	0	-0.004	0.005	
Real - Pseudo	Samegen	LTPJ~RIFG	Together	-0.003	-0.006	0.001	
Real - Pseudo	Samegen	RIFG~RTPJ	Alone	0.003	-0.003	0.008	
Real - Pseudo	Samegen	RIFG~RTPJ	Together	-0.005	-0.009	0	No overlap

Supplementary Materials: Moffat, Dumas and Cross (2026). *Social interactions between people of same and different generations shape longitudinal changes in interpersonal neural synchrony, loneliness, and social connection.*

S5 Appendix – Table C. HbO. Estimates of slope of change in INS across sessions for real and pseudo dyads per ROI pair and task in unstandardised units.

Dyad Type	Group	ROI Pair	Task	Slope estimate	Lower HPD	Upper HPD	No overlap
Pseudo	Intergen	LIFG~RIFG	Alone	0	0	0.001	No overlap
Real	Intergen	LIFG~RIFG	Alone	0	-0.003	0.003	
Pseudo	Intergen	LIFG~RIFG	Together	0	0	0.001	No overlap
Real	Intergen	LIFG~RIFG	Together	0.001	-0.002	0.004	
Pseudo	Intergen	LTPJ~RTPJ	Alone	0	0	0.001	No overlap
Real	Intergen	LTPJ~RTPJ	Alone	0.003	0	0.007	
Pseudo	Intergen	LTPJ~RTPJ	Together	0	-0.001	0	
Real	Intergen	LTPJ~RTPJ	Together	-0.003	-0.006	0	
Pseudo	Intergen	LIFG~LIFG	Alone	0	0	0.001	
Real	Intergen	LIFG~LIFG	Alone	-0.002	-0.006	0.002	
Pseudo	Intergen	LIFG~LIFG	Together	0	0	0	
Real	Intergen	LIFG~LIFG	Together	-0.001	-0.004	0.003	
Pseudo	Intergen	LTPJ~LTPJ	Alone	0.001	0	0.001	No overlap
Real	Intergen	LTPJ~LTPJ	Alone	-0.002	-0.006	0.002	
Pseudo	Intergen	LTPJ~LTPJ	Together	-0.001	-0.001	0	No overlap
Real	Intergen	LTPJ~LTPJ	Together	0.004	0	0.007	
Pseudo	Intergen	LIFG~LTPJ	Alone	0	0	0	
Real	Intergen	LIFG~LTPJ	Alone	0	-0.003	0.003	
Pseudo	Intergen	LIFG~LTPJ	Together	0	-0.001	0	
Real	Intergen	LIFG~LTPJ	Together	-0.002	-0.004	0.001	
Pseudo	Intergen	LIFG~RTPJ	Alone	0	0	0.001	
Real	Intergen	LIFG~RTPJ	Alone	0.004	0	0.007	No overlap
Pseudo	Intergen	LIFG~RTPJ	Together	0	0	0	
Real	Intergen	LIFG~RTPJ	Together	-0.002	-0.005	0.001	
Pseudo	Intergen	RIFG~RIFG	Alone	0.001	0	0.001	No overlap
Real	Intergen	RIFG~RIFG	Alone	0.002	-0.002	0.006	
Pseudo	Intergen	RIFG~RIFG	Together	0.001	0.001	0.002	No overlap
Real	Intergen	RIFG~RIFG	Together	0.002	-0.001	0.006	
Pseudo	Intergen	RTPJ~RTPJ	Alone	0	0	0.001	
Real	Intergen	RTPJ~RTPJ	Alone	0.006	-0.002	0.013	
Pseudo	Intergen	RTPJ~RTPJ	Together	0	-0.001	0	
Real	Intergen	RTPJ~RTPJ	Together	-0.005	-0.013	0.002	
Pseudo	Intergen	LTPJ~RIFG	Alone	0.001	0	0.001	No overlap
Real	Intergen	LTPJ~RIFG	Alone	0.002	-0.001	0.005	
Pseudo	Intergen	LTPJ~RIFG	Together	0	0	0.001	No overlap
Real	Intergen	LTPJ~RIFG	Together	-0.001	-0.003	0.002	
Pseudo	Intergen	RIFG~RTPJ	Alone	0.001	0	0.001	No overlap
Real	Intergen	RIFG~RTPJ	Alone	0.003	0	0.007	
Pseudo	Intergen	RIFG~RTPJ	Together	0.001	0.001	0.002	No overlap
Real	Intergen	RIFG~RTPJ	Together	-0.003	-0.007	0	
Pseudo	Samegen	LIFG~RIFG	Alone	0	0	0	
Real	Samegen	LIFG~RIFG	Alone	-0.002	-0.005	0.001	
Pseudo	Samegen	LIFG~RIFG	Together	0	0	0	

Dyad Type	Group	ROI Pair	Task	Slope Estimate	Lower HPD	Upper HPD	No overlap
Real	Samegen	LIFG~RIFG	Together	0.001	-0.001	0.004	
Pseudo	Samegen	LTPJ~RTPJ	Alone	0	0	0.001	No overlap
Real	Samegen	LTPJ~RTPJ	Alone	0	-0.004	0.003	
Pseudo	Samegen	LTPJ~RTPJ	Together	0.001	0	0.001	No overlap
Real	Samegen	LTPJ~RTPJ	Together	0.001	-0.002	0.005	
Pseudo	Samegen	LIFG~LIFG	Alone	0	0	0.001	
Real	Samegen	LIFG~LIFG	Alone	-0.003	-0.007	0.001	
Pseudo	Samegen	LIFG~LIFG	Together	0	0	0.001	No overlap
Real	Samegen	LIFG~LIFG	Together	0.002	-0.002	0.006	
Pseudo	Samegen	LTPJ~LTPJ	Alone	0	0	0.001	No overlap
Real	Samegen	LTPJ~LTPJ	Alone	-0.001	-0.004	0.003	
Pseudo	Samegen	LTPJ~LTPJ	Together	0	-0.001	0	
Real	Samegen	LTPJ~LTPJ	Together	-0.001	-0.004	0.003	
Pseudo	Samegen	LIFG~LTPJ	Alone	0	0	0	
Real	Samegen	LIFG~LTPJ	Alone	0.001	-0.002	0.004	
Pseudo	Samegen	LIFG~LTPJ	Together	0	0	0	
Real	Samegen	LIFG~LTPJ	Together	0.003	0.001	0.006	No overlap
Pseudo	Samegen	LIFG~RTPJ	Alone	0.001	0	0.001	No overlap
Real	Samegen	LIFG~RTPJ	Alone	-0.002	-0.006	0.001	
Pseudo	Samegen	LIFG~RTPJ	Together	0.001	0.001	0.001	No overlap
Real	Samegen	LIFG~RTPJ	Together	0.002	-0.001	0.005	
Pseudo	Samegen	RIFG~RIFG	Alone	-0.001	-0.001	0	No overlap
Real	Samegen	RIFG~RIFG	Alone	0	-0.004	0.004	
Pseudo	Samegen	RIFG~RIFG	Together	0	0	0	
Real	Samegen	RIFG~RIFG	Together	-0.002	-0.005	0.002	
Pseudo	Samegen	RTPJ~RTPJ	Alone	0	0	0.001	
Real	Samegen	RTPJ~RTPJ	Alone	0.003	-0.004	0.009	
Pseudo	Samegen	RTPJ~RTPJ	Together	0.002	0.002	0.003	No overlap
Real	Samegen	RTPJ~RTPJ	Together	0.009	0.002	0.016	No overlap
Pseudo	Samegen	LTPJ~RIFG	Alone	0	0	0	
Real	Samegen	LTPJ~RIFG	Alone	0.001	-0.002	0.004	
Pseudo	Samegen	LTPJ~RIFG	Together	0	0	0	
Real	Samegen	LTPJ~RIFG	Together	-0.002	-0.005	0	
Pseudo	Samegen	RIFG~RTPJ	Alone	0	0	0	
Real	Samegen	RIFG~RTPJ	Alone	-0.002	-0.006	0.001	
Pseudo	Samegen	RIFG~RTPJ	Together	0.001	0.001	0.001	No overlap
Real	Samegen	RIFG~RTPJ	Together	0.002	-0.001	0.006	

S5 Appendix – Table D. HbO. Contrasts between real and pseudo dyads' slopes of change in INS across sessions per ROI pair and task in unstandardised units.

Contrast	Group	ROI Pair	Task	Estimate	Lower HPD	Upper HPD	No overlap
Real - Pseudo	Intergen	LIFG~RIFG	Alone	0	-0.002	0.003	
Real - Pseudo	Intergen	LIFG~RIFG	Together	-0.001	-0.003	0.002	
Real - Pseudo	Intergen	LTPJ~RTPJ	Alone	-0.003	-0.006	0.001	
Real - Pseudo	Intergen	LTPJ~RTPJ	Together	0.003	-0.001	0.006	
Real - Pseudo	Intergen	LIFG~LIFG	Alone	0.002	-0.002	0.006	
Real - Pseudo	Intergen	LIFG~LIFG	Together	0.001	-0.003	0.004	
Real - Pseudo	Intergen	LTPJ~LTPJ	Alone	0.003	-0.001	0.007	
Real - Pseudo	Intergen	LTPJ~LTPJ	Together	-0.004	-0.008	-0.001	No overlap
Real - Pseudo	Intergen	LIFG~LTPJ	Alone	0	-0.003	0.003	
Real - Pseudo	Intergen	LIFG~LTPJ	Together	0.002	-0.001	0.004	
Real - Pseudo	Intergen	LIFG~RTPJ	Alone	-0.004	-0.007	0	No overlap
Real - Pseudo	Intergen	LIFG~RTPJ	Together	0.002	-0.001	0.005	
Real - Pseudo	Intergen	RIFG~RIFG	Alone	-0.001	-0.005	0.003	
Real - Pseudo	Intergen	RIFG~RIFG	Together	-0.001	-0.005	0.003	
Real - Pseudo	Intergen	RTPJ~RTPJ	Alone	-0.005	-0.012	0.002	
Real - Pseudo	Intergen	RTPJ~RTPJ	Together	0.005	-0.002	0.012	
Real - Pseudo	Intergen	LTPJ~RIFG	Alone	-0.001	-0.004	0.002	
Real - Pseudo	Intergen	LTPJ~RIFG	Together	0.001	-0.001	0.004	
Real - Pseudo	Intergen	RIFG~RTPJ	Alone	-0.002	-0.006	0.001	
Real - Pseudo	Intergen	RIFG~RTPJ	Together	0.005	0.001	0.008	No overlap
Real - Pseudo	Samegen	LIFG~RIFG	Alone	0.002	-0.001	0.005	
Real - Pseudo	Samegen	LIFG~RIFG	Together	-0.001	-0.003	0.002	
Real - Pseudo	Samegen	LTPJ~RTPJ	Alone	0.001	-0.003	0.004	
Real - Pseudo	Samegen	LTPJ~RTPJ	Together	-0.001	-0.004	0.003	
Real - Pseudo	Samegen	LIFG~LIFG	Alone	0.003	-0.001	0.007	
Real - Pseudo	Samegen	LIFG~LIFG	Together	-0.002	-0.005	0.002	
Real - Pseudo	Samegen	LTPJ~LTPJ	Alone	0.001	-0.003	0.005	
Real - Pseudo	Samegen	LTPJ~LTPJ	Together	0	-0.003	0.004	
Real - Pseudo	Samegen	LIFG~LTPJ	Alone	-0.001	-0.004	0.002	
Real - Pseudo	Samegen	LIFG~LTPJ	Together	-0.003	-0.006	0	No overlap
Real - Pseudo	Samegen	LIFG~RTPJ	Alone	0.003	0	0.006	
Real - Pseudo	Samegen	LIFG~RTPJ	Together	-0.001	-0.004	0.002	
Real - Pseudo	Samegen	RIFG~RIFG	Alone	-0.001	-0.005	0.003	
Real - Pseudo	Samegen	RIFG~RIFG	Together	0.002	-0.002	0.006	
Real - Pseudo	Samegen	RTPJ~RTPJ	Alone	-0.002	-0.009	0.005	
Real - Pseudo	Samegen	RTPJ~RTPJ	Together	-0.007	-0.014	0	No overlap
Real - Pseudo	Samegen	LTPJ~RIFG	Alone	-0.001	-0.004	0.002	
Real - Pseudo	Samegen	LTPJ~RIFG	Together	0.002	0	0.005	
Real - Pseudo	Samegen	RIFG~RTPJ	Alone	0.002	-0.001	0.006	
Real - Pseudo	Samegen	RIFG~RTPJ	Together	-0.002	-0.005	0.002	

S5 Appendix – Table E. HbO. Composition of non-homologous ROI pairs, to assess whether an ROI belonging to an older or younger member of a dyad influenced INS levels for all sessions (top) and across sessions (bottom). In contrast column, 'o' indicated that ROI belongs to older adult and 'y' indicates that ROI belongs to younger adult.

Contrast	Task	Estimate	Lower HPD	Upper HPD	No overlap
All sessions					
o_l_ifg_y_l_tpj - o_l_tpj_y_l_ifg	Together	0.007	0	0.014	trend
o_l_ifg_y_r_ifg - o_r_ifg_y_l_ifg	Together	-0.001	-0.009	0.006	
o_l_tpj_y_r_ifg - o_r_ifg_y_l_tpj	Together	0	-0.007	0.008	
Across sessions					
o_l_ifg_y_r_tpj - o_r_tpj_y_l_ifg	Alone	0	-0.011	0.012	
o_r_ifg_y_r_tpj - o_r_tpj_y_r_ifg	Together	-0.002	-0.014	0.009	

S5 Appendix – Table F. HbO. INS levels for all sessions: contrasts between tasks, groups and the task*group interaction per ROI pair in unstandardised units.

Contrast	ROI Pair	Estimate	Lower HPD	Upper HPD	No overlap
Tog-Alo	LIFG~RIFG	0.01	0.001	0.019	No overlap
Int-Sam	LIFG~RIFG	-0.001	-0.012	0.01	
Tog-Alo Int	LIFG~RIFG	0.006	-0.001	0.012	
Tog-Alo Sam	LIFG~RIFG	0.004	-0.002	0.011	
Int-Sam Alo	LIFG~RIFG	-0.001	-0.009	0.007	
Int-Sam Tog	LIFG~RIFG	0	-0.006	0.006	
Tog-Alo	LIFG~LIFG	0.007	-0.005	0.018	
Int-Sam	LIFG~LIFG	0.002	-0.011	0.014	
Tog-Alo Int	LIFG~LIFG	0.005	-0.003	0.014	
Tog-Alo Sam	LIFG~LIFG	0.001	-0.007	0.01	
Int-Sam Alo	LIFG~LIFG	-0.001	-0.01	0.009	
Int-Sam Tog	LIFG~LIFG	0.003	-0.004	0.01	
Tog-Alo	LTPJ~LTPJ	0.013	0.001	0.025	No overlap
Int-Sam	LTPJ~LTPJ	0.004	-0.012	0.02	
Tog-Alo Int	LTPJ~LTPJ	0.009	0	0.017	No overlap
Tog-Alo Sam	LTPJ~LTPJ	0.004	-0.004	0.013	
Int-Sam Alo	LTPJ~LTPJ	0	-0.011	0.011	
Int-Sam Tog	LTPJ~LTPJ	0.004	-0.005	0.013	
Tog-Alo	LIFG~LTPJ	0.005	-0.004	0.013	
Int-Sam	LIFG~LTPJ	0.006	-0.005	0.017	
Tog-Alo Int	LIFG~LTPJ	0.003	-0.003	0.009	
Tog-Alo Sam	LIFG~LTPJ	0.001	-0.005	0.008	
Int-Sam Alo	LIFG~LTPJ	0.002	-0.005	0.01	
Int-Sam Tog	LIFG~LTPJ	0.004	-0.002	0.01	
Tog-Alo	RIFG~RIFG	0.004	-0.009	0.018	
Int-Sam	RIFG~RIFG	0.007	-0.008	0.021	
Tog-Alo Int	RIFG~RIFG	-0.002	-0.011	0.008	
Tog-Alo Sam	RIFG~RIFG	0.006	-0.003	0.015	
Int-Sam Alo	RIFG~RIFG	0.007	-0.004	0.017	
Int-Sam Tog	RIFG~RIFG	0	-0.009	0.008	
Tog-Alo	RIFG~LTPJ	0.01	0.001	0.018	No overlap
Int-Sam	RIFG~LTPJ	-0.001	-0.011	0.008	
Tog-Alo Int	RIFG~LTPJ	0.005	-0.001	0.011	
Tog-Alo Sam	RIFG~LTPJ	0.005	-0.001	0.011	
Int-Sam Alo	RIFG~LTPJ	-0.001	-0.008	0.006	
Int-Sam Tog	RIFG~LTPJ	-0.001	-0.006	0.005	
Tog-Alo	RIFG~RTPJ	0.013	0.002	0.024	No overlap
Int-Sam	RIFG~RTPJ	-0.006	-0.02	0.007	
Tog-Alo Int	RIFG~RTPJ	0.006	-0.003	0.014	
Tog-Alo Sam	RIFG~RTPJ	0.008	0	0.015	No overlap
Int-Sam Alo	RIFG~RTPJ	-0.002	-0.012	0.007	
Int-Sam Tog	RIFG~RTPJ	-0.004	-0.012	0.004	

Supplementary Materials: Moffat, Dumas and Cross (2026). *Social interactions between people of same and different generations shape longitudinal changes in interpersonal neural synchrony, loneliness, and social connection.*

S5 Appendix – Table G. HbO. Contrasts between 1st and 2nd instance of drawing together for all sessions combined: contrasts between drawing instance and drawing instance* group interaction per ROI pair in unstandardised units. Tog1 = 1st instance of drawing together, Tog2 = 2nd instance of drawing together. Int–Same 1 = group contrast for 1st instance of drawing together, Int–Same 2 = group contrast for 2nd instance of drawing together.

Contrast	ROI Pair	Estimate	Lower HPD	Upper HPD	No overlap
Tog1-Tog2	LIFG~RIFG	0.006	-0.005	0.017	
Int1-2	LIFG~RIFG	0.000	-0.008	0.008	
Same1-2	LIFG~RIFG	0.006	-0.002	0.013	
Int-Same 1	LIFG~RIFG	-0.003	-0.012	0.006	
Int-Same 2	LIFG~RIFG	0.003	-0.006	0.012	
Tog1-Tog2	LIFG~LIFG	-0.001	-0.015	0.014	
Int1-2	LIFG~LIFG	0.001	-0.009	0.011	
Same1-2	LIFG~LIFG	-0.002	-0.012	0.008	
Int-Same 1	LIFG~LIFG	0.005	-0.006	0.015	
Int-Same 2	LIFG~LIFG	0.001	-0.009	0.012	
Tog1-Tog2	LTPJ~LTPJ	0.004	-0.012	0.019	
Int1-2	LTPJ~LTPJ	0.001	-0.010	0.012	
Same1-2	LTPJ~LTPJ	0.003	-0.007	0.014	
Int-Same 1	LTPJ~LTPJ	0.003	-0.009	0.015	
Int-Same 2	LTPJ~LTPJ	0.005	-0.007	0.017	
Tog1-Tog2	LIFG~LTPJ	-0.003	-0.013	0.007	
Int1-2	LIFG~LTPJ	0.000	-0.007	0.007	
Same1-2	LIFG~LTPJ	-0.003	-0.010	0.004	
Int-Same 1	LIFG~LTPJ	0.007	-0.002	0.015	
Int-Same 2	LIFG~LTPJ	0.004	-0.005	0.013	
Tog1-Tog2	RIFG~RIFG	0.002	-0.014	0.018	

S5 Appendix – Table H. HbO. Change in INS across sessions: contrasts between tasks, groups and the task*group interaction per ROI pair in unstandardised units.

Contrast	ROI Pair	Estimate	Lower HPD	Upper HPD	No overlap
Tog-Alo	LTPJ~LTPJ	0.006	-0.002	0.014	
Int-Sam	LTPJ~LTPJ	0.003	-0.005	0.01	
Tog-Alo Int	LTPJ~LTPJ	0.006	0	0.011	No overlap
Tog-Alo Sam	LTPJ~LTPJ	0	-0.006	0.005	
Int-Sam Alo	LTPJ~LTPJ	-0.002	-0.007	0.004	
Int-Sam Tog	LTPJ~LTPJ	0.004	-0.001	0.01	
Tog-Alo	LIFG~LTPJ	0	-0.005	0.005	
Int-Sam	LIFG~LTPJ	-0.006	-0.012	-0.001	No overlap
Tog-Alo Int	LIFG~LTPJ	-0.002	-0.006	0.002	
Tog-Alo Sam	LIFG~LTPJ	0.002	-0.002	0.006	
Int-Sam Alo	LIFG~LTPJ	-0.001	-0.005	0.003	
Int-Sam Tog	LIFG~LTPJ	-0.005	-0.009	-0.001	No overlap
Tog-Alo	LIFG~RTPJ	-0.002	-0.008	0.005	
Int-Sam	LIFG~RTPJ	0.002	-0.005	0.009	
Tog-Alo Int	LIFG~RTPJ	-0.006	-0.011	-0.001	No overlap
Tog-Alo Sam	LIFG~RTPJ	0.004	0	0.009	
Int-Sam Alo	LIFG~RTPJ	0.006	0.001	0.011	No overlap
Int-Sam Tog	LIFG~RTPJ	-0.004	-0.009	0	
Tog-Alo	RTPJ~RTPJ	-0.004	-0.019	0.009	
Int-Sam	RTPJ~RTPJ	-0.011	-0.026	0.003	
Tog-Alo Int	RTPJ~RTPJ	-0.011	-0.021	0	No overlap
Tog-Alo Sam	RTPJ~RTPJ	0.006	-0.003	0.016	
Int-Sam Alo	RTPJ~RTPJ	0.003	-0.007	0.013	
Int-Sam Tog	RTPJ~RTPJ	-0.014	-0.024	-0.004	No overlap
Tog-Alo	RIFG~RTPJ	-0.002	-0.009	0.005	
Int-Sam	RIFG~RTPJ	-0.001	-0.008	0.007	
Tog-Alo Int	RIFG~RTPJ	-0.007	-0.012	-0.002	No overlap
Tog-Alo Sam	RIFG~RTPJ	0.004	0	0.009	
Int-Sam Alo	RIFG~RTPJ	0.005	0	0.011	No overlap
Int-Sam Tog	RIFG~RTPJ	-0.006	-0.011	-0.001	No overlap

S5 Appendix – Table I. HbO. Relationships between session and INS in unstandardised units. Effects are presented in the following order: Main, group, task, and simple effects.

Measure	Group	Task	ROI Pairs	Estimate	Lower HPD	Upper HPD	No overlap
Main effect							
session			LIFG~RIFG	-0.0004	-0.0024	0.0016	
session			LIFG~LIFG	-0.0002	-0.0025	0.0019	
session			LTPJ~LTPJ	0.0004	-0.0011	0.0020	
session			LIFG~LTPJ	0.0002	-0.0017	0.0021	
session			LIFG~RTPJ	0.0008	-0.0015	0.0030	
session			RIFG~RIFG	0.0046	0.0002	0.0091	
session			RTPJ~RTPJ	-0.0003	-0.0018	0.0012	No overlap
session			RIFG~LTPJ	-0.0012	-0.0032	0.0008	
session			RIFG~RTPJ	0.0003	-0.0010	0.0016	
Group effect							
session	Intergen		LIFG~RIFG	0.0002	-0.0020	0.0024	
session	Samegen		LIFG~RIFG	-0.0007	-0.0030	0.0017	
session	Intergen		LIFG~LIFG	-0.0005	-0.0033	0.0023	
session	Samegen		LIFG~LIFG	-0.0003	-0.0034	0.0026	
session	Intergen		LTPJ~LTPJ	0.0009	-0.0022	0.0039	
session	Samegen		LTPJ~LTPJ	-0.0014	-0.0046	0.0019	
session	Intergen		LIFG~LTPJ	-0.0012	-0.0033	0.0009	
session	Samegen		LIFG~LTPJ	0.0021	-0.0001	0.0044	
session	Intergen		LIFG~RTPJ	0.0010	-0.0016	0.0036	
session	Samegen		LIFG~RTPJ	-0.0006	-0.0033	0.0020	
session	Intergen		RIFG~RIFG	0.0012	-0.0020	0.0044	
session	Samegen		RIFG~RIFG	0.0004	-0.0028	0.0036	
session	Intergen		RTPJ~RTPJ	0.0015	-0.0047	0.0078	
session	Samegen		RTPJ~RTPJ	0.0077	0.0013	0.0138	No overlap
session	Intergen		RIFG~LTPJ	0.0003	-0.0018	0.0024	
session	Samegen		RIFG~LTPJ	-0.0009	-0.0030	0.0013	
session	Intergen		RIFG~RTPJ	-0.0003	-0.0030	0.0026	
session	Samegen		RIFG~RTPJ	-0.0020	-0.0049	0.0008	
Task effect							
session		Alone	LIFG~RIFG	-0.0017	-0.0040	0.0006	
session		Together	LIFG~RIFG	0.0012	-0.0010	0.0032	
session		Alone	LIFG~LIFG	-0.0008	-0.0038	0.0023	
session		Together	LIFG~LIFG	0.0000	-0.0028	0.0027	
session		Alone	LTPJ~LTPJ	-0.0014	-0.0046	0.0019	
session		Together	LTPJ~LTPJ	0.0009	-0.0020	0.0039	
session		Alone	LIFG~LTPJ	0.0009	-0.0015	0.0031	
session		Together	LIFG~LTPJ	0.0000	-0.0020	0.0021	
session		Alone	LIFG~RTPJ	-0.0001	-0.0028	0.0027	
session		Together	LIFG~RTPJ	0.0005	-0.0019	0.0030	
session		Alone	RIFG~RIFG	0.0012	-0.0020	0.0046	
session		Together	RIFG~RIFG	0.0004	-0.0027	0.0034	
session		Alone	RTPJ~RTPJ	0.0040	-0.0020	0.0105	

Measure	Group	Task	ROI Pairs	Estimate	Lower HPD	Upper HPD	No overlap
session		Together	RTPJ~RTPJ	0.0051	-0.0008	0.0111	
session		Alone	RIFG~LTPJ	0.0013	-0.0010	0.0035	
session		Together	RIFG~LTPJ	-0.0019	-0.0039	0.0002	
session		Alone	RIFG~RTPJ	-0.0016	-0.0045	0.0013	
session		Together	RIFG~RTPJ	-0.0007	-0.0034	0.0019	
Simple effect							
session	Intergen	Alone	LIFG~RIFG	-0.0007	-0.0040	0.0026	
session	Intergen	Together	LIFG~RIFG	0.0011	-0.0018	0.0040	
session	Samegen	Alone	LIFG~RIFG	-0.0026	-0.0060	0.0008	
session	Samegen	Together	LIFG~RIFG	0.0012	-0.0019	0.0042	
session	Intergen	Alone	LIFG~LIFG	-0.0003	-0.0046	0.0037	
session	Intergen	Together	LIFG~LIFG	-0.0007	-0.0043	0.0032	
session	Samegen	Alone	LIFG~LIFG	-0.0013	-0.0058	0.0032	
session	Samegen	Together	LIFG~LIFG	0.0006	-0.0033	0.0046	
session	Intergen	Alone	LTPJ~LTPJ	-0.0016	-0.0060	0.0028	
session	Intergen	Together	LTPJ~LTPJ	0.0034	-0.0006	0.0075	
session	Samegen	Alone	LTPJ~LTPJ	-0.0012	-0.0058	0.0037	
session	Samegen	Together	LTPJ~LTPJ	-0.0015	-0.0056	0.0029	
session	Intergen	Alone	LIFG~LTPJ	0.0005	-0.0025	0.0036	
session	Intergen	Together	LIFG~LTPJ	-0.0029	-0.0058	-0.0001	No overlap
session	Samegen	Alone	LIFG~LTPJ	0.0012	-0.0021	0.0045	
session	Samegen	Together	LIFG~LTPJ	0.0029	0.0000	0.0059	
session	Intergen	Alone	LIFG~RTPJ	0.0034	-0.0003	0.0072	
session	Intergen	Together	LIFG~RTPJ	-0.0015	-0.0049	0.0020	
session	Samegen	Alone	LIFG~RTPJ	-0.0037	-0.0078	0.0002	
session	Samegen	Together	LIFG~RTPJ	0.0024	-0.0010	0.0060	
session	Intergen	Alone	RIFG~RIFG	0.0004	-0.0044	0.0051	
session	Intergen	Together	RIFG~RIFG	0.0021	-0.0023	0.0062	
session	Samegen	Alone	RIFG~RIFG	0.0021	-0.0025	0.0070	
session	Samegen	Together	RIFG~RIFG	-0.0014	-0.0056	0.0029	
session	Intergen	Alone	RTPJ~RTPJ	0.0061	-0.0024	0.0148	
session	Intergen	Together	RTPJ~RTPJ	-0.0032	-0.0119	0.0053	
session	Samegen	Alone	RTPJ~RTPJ	0.0021	-0.0074	0.0111	
session	Samegen	Together	RTPJ~RTPJ	0.0134	0.0052	0.0217	No overlap
session	Intergen	Alone	RIFG~LTPJ	0.0020	-0.0012	0.0051	
session	Intergen	Together	RIFG~LTPJ	-0.0015	-0.0043	0.0013	
session	Samegen	Alone	RIFG~LTPJ	0.0005	-0.0028	0.0037	
session	Samegen	Together	RIFG~LTPJ	-0.0022	-0.0052	0.0007	
session	Intergen	Alone	RIFG~RTPJ	0.0025	-0.0013	0.0067	
session	Intergen	Together	RIFG~RTPJ	-0.0031	-0.0069	0.0007	
session	Samegen	Alone	RIFG~RTPJ	-0.0058	-0.0100	-0.0016	No overlap
session	Samegen	Together	RIFG~RTPJ	0.0016	-0.0020	0.0054	

Supplementary Materials: Moffat, Dumas and Cross (2026). *Social interactions between people of same and different generations shape longitudinal changes in interpersonal neural synchrony, loneliness, and social connection.*

S5 Appendix – Table J. HbO. Contrasts pertaining to session ~ INS relationship in unstandardised units. Contrasts are presented in the following order: Group, task, and interaction contrasts.

Measure	Group	Task	ROI Pairs	Estimate	Lower HPD	Upper HPD	No overlap
Group contrast							
session	Intergen-Samegen		LIFG~RIFG	0.0009	-0.0023	0.0041	
session	Intergen-Samegen		LIFG~LIFG	-0.0002	-0.0043	0.0039	
session	Intergen-Samegen		LTPJ~LTPJ	0.0022	-0.0023	0.0067	
session	Intergen-Samegen		LIFG~LTPJ	-0.0033	-0.0064	-0.0002	No overlap
session	Intergen-Samegen		LIFG~RTPJ	0.0016	-0.0021	0.0053	
session	Intergen-Samegen		RIFG~RIFG	0.0008	-0.0037	0.0054	
session	Intergen-Samegen		RTPJ~RTPJ	-0.0062	-0.0150	0.0027	
session	Intergen-Samegen		RIFG~LTPJ	0.0011	-0.0020	0.0041	
session	Intergen-Samegen		RIFG~RTPJ	0.0018	-0.0021	0.0059	
Task contrast							
session		Alone-Together	LIFG~RIFG	-0.0028	-0.0060	0.0002	
session		Alone-Together	LIFG~LIFG	-0.0007	-0.0049	0.0032	
session		Alone-Together	LTPJ~LTPJ	-0.0023	-0.0066	0.0019	
session		Alone-Together	LIFG~LTPJ	0.0008	-0.0023	0.0038	
session		Alone-Together	LIFG~RTPJ	-0.0006	-0.0044	0.0030	
session		Alone-Together	RIFG~RIFG	0.0009	-0.0036	0.0054	
session		Alone-Together	RTPJ~RTPJ	-0.0010	-0.0099	0.0072	
session		Alone-Together	RIFG~LTPJ	0.0032	0.0002	0.0062	No overlap
session		Alone-Together	RIFG~RTPJ	-0.0009	-0.0048	0.0029	
Interactions							
session	Intergen-Samegen	Alone	LIFG~RIFG	0.0019	-0.0029	0.0065	
session	Intergen-Samegen	Together	LIFG~RIFG	0	-0.0044	0.0041	
session	Intergen-Samegen	Alone	LIFG~LIFG	0.001	-0.0052	0.0069	
session	Intergen-Samegen	Together	LIFG~LIFG	-0.0012	-0.0065	0.0043	
session	Intergen-Samegen	Alone	LTPJ~LTPJ	-5.00E-04	-0.0068	0.0063	
session	Intergen-Samegen	Together	LTPJ~LTPJ	0.0049	-0.001	0.0107	
session	Intergen-Samegen	Alone	LIFG~LTPJ	-7.00E-04	-0.0051	0.0039	
session	Intergen-Samegen	Together	LIFG~LTPJ	-0.0058	-0.0099	-0.0017	No overlap
session	Intergen-Samegen	Alone	LIFG~RTPJ	0.0072	0.0018	0.0127	No overlap
session	Intergen-Samegen	Together	LIFG~RTPJ	-0.004	-0.0091	7.00E-04	
session	Intergen-Samegen	Alone	RIFG~RIFG	-0.0017	-0.0082	0.0052	
session	Intergen-Samegen	Together	RIFG~RIFG	0.0034	-0.0027	0.0094	
session	Intergen-Samegen	Alone	RTPJ~RTPJ	0.0042	-0.0087	0.0167	
session	Intergen-Samegen	Together	RTPJ~RTPJ	-0.0165	-0.0285	-0.0048	No overlap
session	Intergen-Samegen	Alone	RIFG~LTPJ	0.0015	-0.0031	0.006	
session	Intergen-Samegen	Together	RIFG~LTPJ	7.00E-04	-0.0033	0.0049	
session	Intergen-Samegen	Alone	RIFG~RTPJ	0.0083	0.0024	0.0141	No overlap
session	Intergen-Samegen	Together	RIFG~RTPJ	-0.0047	-0.0101	6.00E-04	
session	Intergen	Alone-Together	LIFG~RIFG	-0.0019	-0.0062	0.0025	
session	Samegen	Alone-Together	LIFG~RIFG	-0.0038	-0.0082	0.0007	
session	Intergen	Alone-Together	LIFG~LIFG	0.0004	-0.0054	0.0058	
session	Samegen	Alone-Together	LIFG~LIFG	-0.0018	-0.0078	0.0041	

Measure	Group	Task	ROI Pairs	Estimate	Lower HPD	Upper HPD	No overlap
session	Intergen	Alone-Together	LTPJ~LTPJ	-0.0050	-0.0111	0.0007	
session	Samegen	Alone-Together	LTPJ~LTPJ	0.0003	-0.0059	0.0065	
session	Intergen	Alone-Together	LIFG~LTPJ	0.0034	-0.0008	0.0074	
session	Samegen	Alone-Together	LIFG~LTPJ	-0.0017	-0.0061	0.0027	
session	Intergen	Alone-Together	LIFG~RTPJ	0.0049	-0.0002	0.0099	
session	Samegen	Alone-Together	LIFG~RTPJ	-0.0062	-0.0116	-0.0011	No overlap
session	Intergen	Alone-Together	RIFG~RIFG	-0.0017	-0.0080	0.0046	
session	Samegen	Alone-Together	RIFG~RIFG	0.0035	-0.0029	0.0098	
session	Intergen	Alone-Together	RTPJ~RTPJ	0.0093	-0.0028	0.0210	
session	Samegen	Alone-Together	RTPJ~RTPJ	-0.0113	-0.0232	0.0011	
session	Intergen	Alone-Together	RIFG~LTPJ	0.0035	-0.0006	0.0078	
session	Samegen	Alone-Together	RIFG~LTPJ	0.0028	-0.0016	0.0071	
session	Intergen	Alone-Together	RIFG~RTPJ	0.0056	0.0003	0.0111	No overlap
session	Samegen	Alone-Together	RIFG~RTPJ	-0.0074	-0.0131	-0.0020	No overlap

S5 Appendix – Table K. HbO. Relationships between loneliness (sum) and INS in unstandardised units. Effects are presented in the following order: Main, group, task, and simple effects.

Measure	Group	Task	ROI Pairs	Estimate	Lower HPD	Upper HPD	No overlap
Main effect							
sum loneliness			LIFG~RIFG	0.0003	-0.001	0.0016	
sum loneliness			LIFG~LIFG	0.0002	-0.0015	0.0018	
sum loneliness			LTPJ~LTPJ	0.0004	-0.0016	0.0024	
sum loneliness			LIFG~LTPJ	0.0004	-0.0009	0.0017	
sum loneliness			LIFG~RTPJ	-0.0012	-0.0027	0.0003	
sum loneliness			RIFG~RIFG	-0.0003	-0.0021	0.0015	
sum loneliness			RTPJ~RTPJ	-0.0009	-0.0041	0.0021	
sum loneliness			RIFG~LTPJ	-0.0001	-0.0013	0.0011	
sum loneliness			RIFG~RTPJ	-0.0006	-0.0023	0.0011	
Group effect							
sum loneliness	Intergen		LIFG~RIFG	-0.0005	-0.0023	0.0013	
sum loneliness	Samegen		LIFG~RIFG	0.001	-0.0008	0.0029	
sum loneliness	Intergen		LIFG~LIFG	0.0001	-0.0022	0.0023	
sum loneliness	Samegen		LIFG~LIFG	0.0004	-0.0019	0.0027	
sum loneliness	Intergen		LTPJ~LTPJ	0.0007	-0.002	0.0034	
sum loneliness	Samegen		LTPJ~LTPJ	0.0001	-0.0027	0.003	
sum loneliness	Intergen		LIFG~LTPJ	0.0002	-0.0016	0.002	
sum loneliness	Samegen		LIFG~LTPJ	0.0005	-0.0014	0.0024	
sum loneliness	Intergen		LIFG~RTPJ	-0.0012	-0.0032	0.0009	
sum loneliness	Samegen		LIFG~RTPJ	-0.0011	-0.0033	0.001	
sum loneliness	Intergen		RIFG~RIFG	-0.0003	-0.0028	0.0022	
sum loneliness	Samegen		RIFG~RIFG	-0.0004	-0.0029	0.0022	
sum loneliness	Intergen		RTPJ~RTPJ	-0.0004	-0.0045	0.0037	
sum loneliness	Samegen		RTPJ~RTPJ	-0.0014	-0.0061	0.0033	
sum loneliness	Intergen		RIFG~LTPJ	0	-0.0016	0.0017	
sum loneliness	Samegen		RIFG~LTPJ	-0.0003	-0.0021	0.0014	
sum loneliness	Intergen		RIFG~RTPJ	-0.0017	-0.004	0.0007	
sum loneliness	Samegen		RIFG~RTPJ	0.0005	-0.0018	0.0029	
Task effect							
sum loneliness		Alone	LIFG~RIFG	-0.0001	-0.002	0.0018	
sum loneliness		Together	LIFG~RIFG	0.0006	-0.0009	0.0022	
sum loneliness		Alone	LIFG~LIFG	0.0004	-0.002	0.0029	
sum loneliness		Together	LIFG~LIFG	0	-0.0019	0.002	
sum loneliness		Alone	LTPJ~LTPJ	0.0016	-0.0013	0.0043	
sum loneliness		Together	LTPJ~LTPJ	-0.0008	-0.003	0.0015	
sum loneliness		Alone	LIFG~LTPJ	0.0007	-0.0012	0.0026	
sum loneliness		Together	LIFG~LTPJ	0	-0.0015	0.0016	
sum loneliness		Alone	LIFG~RTPJ	-0.0017	-0.0039	0.0005	
sum loneliness		Together	LIFG~RTPJ	-0.0006	-0.0024	0.0011	
sum loneliness		Alone	RIFG~RIFG	-0.0017	-0.0044	0.001	
sum loneliness		Together	RIFG~RIFG	0.0011	-0.0011	0.0032	
sum loneliness		Alone	RTPJ~RTPJ	-0.0013	-0.0059	0.0031	

Measure	Group	Task	ROI Pairs	Estimate	Lower HPD	Upper HPD	No overlap
sum loneliness		Together	RTPJ~RTPJ	-0.0005	-0.0043	0.0034	
sum loneliness		Alone	RIFG~LTPJ	-0.0003	-0.0021	0.0015	
sum loneliness		Together	RIFG~LTPJ	0.0001	-0.0014	0.0015	
sum loneliness		Alone	RIFG~RTPJ	-0.0016	-0.004	0.0008	
sum loneliness		Together	RIFG~RTPJ	0.0005	-0.0015	0.0024	
Simple effect							
sum loneliness	Intergen	Alone	LIFG~RIFG	-0.0014	-0.0040	0.0013	
sum loneliness	Intergen	Together	LIFG~RIFG	0.0004	-0.0018	0.0024	
sum loneliness	Samegen	Alone	LIFG~RIFG	0.0012	-0.0016	0.0038	
sum loneliness	Samegen	Together	LIFG~RIFG	0.0009	-0.0012	0.0031	
sum loneliness	Intergen	Alone	LIFG~LIFG	0.0017	-0.0017	0.0051	
sum loneliness	Intergen	Together	LIFG~LIFG	-0.0016	-0.0043	0.0010	
sum loneliness	Samegen	Alone	LIFG~LIFG	-0.0009	-0.0044	0.0027	
sum loneliness	Samegen	Together	LIFG~LIFG	0.0016	-0.0012	0.0044	
sum loneliness	Intergen	Alone	LTPJ~LTPJ	0.0020	-0.0018	0.0058	
sum loneliness	Intergen	Together	LTPJ~LTPJ	-0.0005	-0.0036	0.0026	
sum loneliness	Samegen	Alone	LTPJ~LTPJ	0.0012	-0.0028	0.0052	
sum loneliness	Samegen	Together	LTPJ~LTPJ	-0.0010	-0.0043	0.0023	
sum loneliness	Intergen	Alone	LIFG~LTPJ	0.0001	-0.0025	0.0027	
sum loneliness	Intergen	Together	LIFG~LTPJ	0.0004	-0.0016	0.0026	
sum loneliness	Samegen	Alone	LIFG~LTPJ	0.0014	-0.0014	0.0041	
sum loneliness	Samegen	Together	LIFG~LTPJ	-0.0003	-0.0026	0.0019	
sum loneliness	Intergen	Alone	LIFG~RTPJ	-0.0024	-0.0054	0.0006	
sum loneliness	Intergen	Together	LIFG~RTPJ	0.0000	-0.0024	0.0025	
sum loneliness	Samegen	Alone	LIFG~RTPJ	-0.0010	-0.0042	0.0021	
sum loneliness	Samegen	Together	LIFG~RTPJ	-0.0013	-0.0038	0.0013	
sum loneliness	Intergen	Alone	RIFG~RIFG	-0.0009	-0.0047	0.0028	
sum loneliness	Intergen	Together	RIFG~RIFG	0.0003	-0.0027	0.0033	
sum loneliness	Samegen	Alone	RIFG~RIFG	-0.0026	-0.0064	0.0012	
sum loneliness	Samegen	Together	RIFG~RIFG	0.0019	-0.0012	0.0050	
sum loneliness	Intergen	Alone	RTPJ~RTPJ	-0.0005	-0.0065	0.0053	
sum loneliness	Intergen	Together	RTPJ~RTPJ	-0.0002	-0.0054	0.0048	
sum loneliness	Samegen	Alone	RTPJ~RTPJ	-0.0021	-0.0089	0.0048	
sum loneliness	Samegen	Together	RTPJ~RTPJ	-0.0007	-0.0066	0.0051	
sum loneliness	Intergen	Alone	RIFG~LTPJ	-0.0009	-0.0035	0.0016	
sum loneliness	Intergen	Together	RIFG~LTPJ	0.0009	-0.0011	0.0029	
sum loneliness	Samegen	Alone	RIFG~LTPJ	0.0002	-0.0023	0.0029	
sum loneliness	Samegen	Together	RIFG~LTPJ	-0.0008	-0.0029	0.0012	
sum loneliness	Intergen	Alone	RIFG~RTPJ	-0.0036	-0.0069	-0.0003	No overlap
sum loneliness	Intergen	Together	RIFG~RTPJ	0.0002	-0.0026	0.0028	
sum loneliness	Samegen	Alone	RIFG~RTPJ	0.0003	-0.0032	0.0037	
sum loneliness	Samegen	Together	RIFG~RTPJ	0.0007	-0.0019	0.0036	
sum loneliness	Intergen	Alone	LIFG~RIFG	-0.0014	-0.0040	0.0013	

Supplementary Materials: Moffat, Dumas and Cross (2026). *Social interactions between people of same and different generations shape longitudinal changes in interpersonal neural synchrony, loneliness, and social connection.*

S5 Appendix – Table L. HbO. Contrasts pertaining to loneliness (sum) ~ INS relationship in unstandardised units. Contrasts are presented in the following order: Group, task, and interaction contrasts.

Measure	Group	Task	ROI Pairs	Estimate	Lower HPD	Upper HPD	No overlap
Group contrast							
sum loneliness	Intergen-Samegen		LIFG~RIFG	-0.0015	-0.0041	0.0010	
sum loneliness	Intergen-Samegen		LIFG~LIFG	-0.0003	-0.0036	0.0029	
sum loneliness	Intergen-Samegen		LTPJ~LTPJ	0.0007	-0.0032	0.0047	
sum loneliness	Intergen-Samegen		LIFG~LTPJ	-0.0003	-0.0030	0.0023	
sum loneliness	Intergen-Samegen		LIFG~RTPJ	-0.0001	-0.0029	0.0029	
sum loneliness	Intergen-Samegen		RIFG~RIFG	0.0001	-0.0035	0.0036	
sum loneliness	Intergen-Samegen		RTPJ~RTPJ	0.0010	-0.0051	0.0073	
sum loneliness	Intergen-Samegen		RIFG~LTPJ	0.0003	-0.0021	0.0027	
sum loneliness	Intergen-Samegen		RIFG~RTPJ	-0.0022	-0.0055	0.0010	
Task contrast							
sum loneliness		Alone-Together	LIFG~RIFG	-0.0007	-0.0030	0.0016	
sum loneliness		Alone-Together	LIFG~LIFG	0.0004	-0.0026	0.0034	
sum loneliness		Alone-Together	LTPJ~LTPJ	0.0023	-0.0009	0.0055	
sum loneliness		Alone-Together	LIFG~LTPJ	0.0007	-0.0015	0.0029	
sum loneliness		Alone-Together	LIFG~RTPJ	-0.0011	-0.0037	0.0015	
sum loneliness		Alone-Together	RIFG~RIFG	-0.0028	-0.0062	0.0004	
sum loneliness		Alone-Together	RTPJ~RTPJ	-0.0009	-0.0065	0.0050	
sum loneliness		Alone-Together	RIFG~LTPJ	-0.0004	-0.0026	0.0019	
sum loneliness		Alone-Together	RIFG~RTPJ	-0.0021	-0.0049	0.0007	
Interactions							
sum loneliness	Intergen-Samegen	Alone	LIFG~RIFG	-0.0025	-0.0062	0.0014	
sum loneliness	Intergen-Samegen	Together	LIFG~RIFG	-0.0005	-0.0035	0.0026	
sum loneliness	Intergen-Samegen	Alone	LIFG~LIFG	0.0026	-0.0023	0.0075	
sum loneliness	Intergen-Samegen	Together	LIFG~LIFG	-0.0032	-0.0070	0.0007	
sum loneliness	Intergen-Samegen	Alone	LTPJ~LTPJ	0.0008	-0.0047	0.0063	
sum loneliness	Intergen-Samegen	Together	LTPJ~LTPJ	0.0005	-0.0040	0.0051	
sum loneliness	Intergen-Samegen	Alone	LIFG~LTPJ	-0.0013	-0.0050	0.0025	
sum loneliness	Intergen-Samegen	Together	LIFG~LTPJ	0.0007	-0.0024	0.0037	
sum loneliness	Intergen-Samegen	Alone	LIFG~RTPJ	-0.0014	-0.0057	0.0029	
sum loneliness	Intergen-Samegen	Together	LIFG~RTPJ	0.0013	-0.0021	0.0048	
sum loneliness	Intergen-Samegen	Alone	RIFG~RIFG	0.0017	-0.0036	0.0071	
sum loneliness	Intergen-Samegen	Together	RIFG~RIFG	-0.0016	-0.0060	0.0025	
sum loneliness	Intergen-Samegen	Alone	RTPJ~RTPJ	0.0016	-0.0074	0.0107	
sum loneliness	Intergen-Samegen	Together	RTPJ~RTPJ	0.0004	-0.0073	0.0083	
sum loneliness	Intergen-Samegen	Alone	RIFG~LTPJ	-0.0012	-0.0048	0.0024	
sum loneliness	Intergen-Samegen	Together	RIFG~LTPJ	0.0017	-0.0011	0.0046	
sum loneliness	Intergen-Samegen	Alone	RIFG~RTPJ	-0.0039	-0.0086	0.0008	
sum loneliness	Intergen-Samegen	Together	RIFG~RTPJ	-0.0005	-0.0043	0.0034	
sum loneliness	Intergen	Alone-Together	LIFG~RIFG	-0.0017	-0.0049	0.0015	
sum loneliness	Samegen	Alone-Together	LIFG~RIFG	0.0002	-0.0031	0.0035	
sum loneliness	Intergen	Alone-Together	LIFG~LIFG	0.0033	-0.0007	0.0076	
sum loneliness	Samegen	Alone-Together	LIFG~LIFG	-0.0025	-0.0068	0.0019	

Measure	Group	Task	ROI Pairs	Estimate	Lower HPD	Upper HPD	No overlap
sum loneliness	Intergen	Alone-Together	LTPJ~LTPJ	0.0025	-0.0019	0.0068	
sum loneliness	Samegen	Alone-Together	LTPJ~LTPJ	0.0022	-0.0024	0.0068	
sum loneliness	Intergen	Alone-Together	LIFG~LTPJ	-0.0003	-0.0034	0.0028	
sum loneliness	Samegen	Alone-Together	LIFG~LTPJ	0.0017	-0.0015	0.0050	
sum loneliness	Intergen	Alone-Together	LIFG~RTPJ	-0.0024	-0.0060	0.0013	
sum loneliness	Samegen	Alone-Together	LIFG~RTPJ	0.0003	-0.0035	0.0041	
sum loneliness	Intergen	Alone-Together	RIFG~RIFG	-0.0011	-0.0058	0.0033	
sum loneliness	Samegen	Alone-Together	RIFG~RIFG	-0.0045	-0.0090	0.0002	
sum loneliness	Intergen	Alone-Together	RTPJ~RTPJ	-0.0003	-0.0078	0.0072	
sum loneliness	Samegen	Alone-Together	RTPJ~RTPJ	-0.0014	-0.0103	0.0072	
sum loneliness	Intergen	Alone-Together	RIFG~LTPJ	-0.0018	-0.0049	0.0013	
sum loneliness	Samegen	Alone-Together	RIFG~LTPJ	0.0011	-0.0021	0.0042	
sum loneliness	Intergen	Alone-Together	RIFG~RTPJ	-0.0038	-0.0077	0.0001	
sum loneliness	Samegen	Alone-Together	RIFG~RTPJ	-0.0004	-0.0045	0.0036	

S5 Appendix – Table M. HbO. Relationships between loneliness (dif) and INS in unstandardised units. Effects are presented in the following order: Main, group, task, and simple effects.

Measure	Group	Task	ROI Pairs	Estimate	Lower HPD	Upper HPD	No overlap
Main effect							
dif loneliness			LIFG~RIFG	-0.0001	-0.0025	0.0022	
dif loneliness			LIFG~LIFG	-0.0006	-0.0035	0.0023	
dif loneliness			LTPJ~LTPJ	-0.0016	-0.005	0.0018	
dif loneliness			LIFG~LTPJ	-0.0014	-0.0037	0.0009	
dif loneliness			LIFG~RTPJ	0	-0.0027	0.0027	
dif loneliness			RIFG~RIFG	-0.0014	-0.0047	0.002	
dif loneliness			RTPJ~RTPJ	-0.0001	-0.0057	0.0056	
dif loneliness			RIFG~LTPJ	-0.0003	-0.0025	0.0019	
dif loneliness			RIFG~RTPJ	-0.0004	-0.0034	0.0025	
Group effect							
dif loneliness	Intergen		LIFG~RIFG	0.0001	-0.0036	0.0038	
dif loneliness	Samegen		LIFG~RIFG	-0.0003	-0.0031	0.0023	
dif loneliness	Intergen		LIFG~LIFG	-0.0008	-0.0055	0.0037	
dif loneliness	Samegen		LIFG~LIFG	-0.0004	-0.0037	0.0032	
dif loneliness	Intergen		LTPJ~LTPJ	-0.0047	-0.0099	0.0006	
dif loneliness	Samegen		LTPJ~LTPJ	0.0016	-0.0027	0.0058	
dif loneliness	Intergen		LIFG~LTPJ	-0.003	-0.0066	0.0006	
dif loneliness	Samegen		LIFG~LTPJ	0.0001	-0.0026	0.0029	
dif loneliness	Intergen		LIFG~RTPJ	0.001	-0.0033	0.0053	
dif loneliness	Samegen		LIFG~RTPJ	-0.0011	-0.0043	0.002	
dif loneliness	Intergen		RIFG~RIFG	-0.0022	-0.0077	0.0033	
dif loneliness	Samegen		RIFG~RIFG	-0.0005	-0.0044	0.0032	
dif loneliness	Intergen		RTPJ~RTPJ	-0.003	-0.0124	0.0066	
dif loneliness	Samegen		RTPJ~RTPJ	0.0028	-0.0037	0.0093	
dif loneliness	Intergen		RIFG~LTPJ	-0.001	-0.0046	0.0025	
dif loneliness	Samegen		RIFG~LTPJ	0.0005	-0.002	0.003	
dif loneliness	Intergen		RIFG~RTPJ	-0.0019	-0.0069	0.003	
dif loneliness	Samegen		RIFG~RTPJ	0.0011	-0.0023	0.0047	
Task effect							
dif loneliness		Alone	LIFG~RIFG	0.0012	-0.0023	0.0046	
dif loneliness		Together	LIFG~RIFG	-0.0015	-0.0043	0.0013	
dif loneliness		Alone	LIFG~LIFG	-0.0014	-0.0058	0.0028	
dif loneliness		Together	LIFG~LIFG	0.0002	-0.0033	0.0038	
dif loneliness		Alone	LTPJ~LTPJ	-0.0038	-0.0087	0.001	
dif loneliness		Together	LTPJ~LTPJ	0.0006	-0.0035	0.0045	
dif loneliness		Alone	LIFG~LTPJ	-0.0013	-0.0046	0.0021	
dif loneliness		Together	LIFG~LTPJ	-0.0016	-0.0044	0.0011	
dif loneliness		Alone	LIFG~RTPJ	0.0017	-0.0024	0.0057	
dif loneliness		Together	LIFG~RTPJ	-0.0017	-0.0049	0.0016	
dif loneliness		Alone	RIFG~RIFG	0.0026	-0.0023	0.0078	
dif loneliness		Together	RIFG~RIFG	-0.0054	-0.0095	-0.0014	No overlap
dif loneliness		Alone	RTPJ~RTPJ	-0.0028	-0.0114	0.0057	

Measure	Group	Task	ROI Pairs	Estimate	Lower HPD	Upper HPD	No overlap
dif loneliness		Together	RTPJ~RTPJ	0.0025	-0.0049	0.0096	
dif loneliness		Alone	RIFG~LTPJ	0.0003	-0.0029	0.0037	
dif loneliness		Together	RIFG~LTPJ	-0.0009	-0.0035	0.0018	
dif loneliness		Alone	RIFG~RTPJ	0.002	-0.0023	0.0063	
dif loneliness		Together	RIFG~RTPJ	-0.0027	-0.0063	0.0008	
Simple effect							
dif loneliness	Intergen	Alone	LIFG~RIFG	0.0016	-0.0039	0.0071	
dif loneliness	Intergen	Together	LIFG~RIFG	-0.0015	-0.0062	0.0030	
dif loneliness	Samegen	Alone	LIFG~RIFG	0.0008	-0.0031	0.0049	
dif loneliness	Samegen	Together	LIFG~RIFG	-0.0015	-0.0047	0.0017	
dif loneliness	Intergen	Alone	LIFG~LIFG	-0.0038	-0.0106	0.0031	
dif loneliness	Intergen	Together	LIFG~LIFG	0.0021	-0.0037	0.0077	
dif loneliness	Samegen	Alone	LIFG~LIFG	0.0009	-0.0043	0.0062	
dif loneliness	Samegen	Together	LIFG~LIFG	-0.0016	-0.0057	0.0026	
dif loneliness	Intergen	Alone	LTPJ~LTPJ	-0.0093	-0.0169	-0.0016	No overlap
dif loneliness	Intergen	Together	LTPJ~LTPJ	-0.0001	-0.0066	0.0060	
dif loneliness	Samegen	Alone	LTPJ~LTPJ	0.0017	-0.0042	0.0076	
dif loneliness	Samegen	Together	LTPJ~LTPJ	0.0015	-0.0034	0.0064	
dif loneliness	Intergen	Alone	LIFG~LTPJ	-0.0017	-0.0069	0.0037	
dif loneliness	Intergen	Together	LIFG~LTPJ	-0.0043	-0.0088	-0.0001	No overlap
dif loneliness	Samegen	Alone	LIFG~LTPJ	-0.0009	-0.0050	0.0032	
dif loneliness	Samegen	Together	LIFG~LTPJ	0.0011	-0.0022	0.0044	
dif loneliness	Intergen	Alone	LIFG~RTPJ	0.0037	-0.0028	0.0101	
dif loneliness	Intergen	Together	LIFG~RTPJ	-0.0015	-0.0069	0.0038	
dif loneliness	Samegen	Alone	LIFG~RTPJ	-0.0003	-0.0050	0.0044	
dif loneliness	Samegen	Together	LIFG~RTPJ	-0.0018	-0.0056	0.0018	
dif loneliness	Intergen	Alone	RIFG~RIFG	0.0027	-0.0053	0.0113	
dif loneliness	Intergen	Together	RIFG~RIFG	-0.0071	-0.0137	-0.0006	No overlap
dif loneliness	Samegen	Alone	RIFG~RIFG	0.0026	-0.0029	0.0083	
dif loneliness	Samegen	Together	RIFG~RIFG	-0.0037	-0.0083	0.0007	
dif loneliness	Intergen	Alone	RTPJ~RTPJ	-0.0058	-0.0199	0.0088	
dif loneliness	Intergen	Together	RTPJ~RTPJ	-0.0003	-0.0126	0.0118	
dif loneliness	Samegen	Alone	RTPJ~RTPJ	0.0003	-0.0094	0.0103	
dif loneliness	Samegen	Together	RTPJ~RTPJ	0.0053	-0.0024	0.0130	
dif loneliness	Intergen	Alone	RIFG~LTPJ	-0.0004	-0.0057	0.0050	
dif loneliness	Intergen	Together	RIFG~LTPJ	-0.0017	-0.0062	0.0025	
dif loneliness	Samegen	Alone	RIFG~LTPJ	0.0011	-0.0027	0.0049	
dif loneliness	Samegen	Together	RIFG~LTPJ	-0.0001	-0.0031	0.0030	
dif loneliness	Intergen	Alone	RIFG~RTPJ	-0.0005	-0.0079	0.0064	
dif loneliness	Intergen	Together	RIFG~RTPJ	-0.0033	-0.0093	0.0026	
dif loneliness	Samegen	Alone	RIFG~RTPJ	0.0045	-0.0004	0.0096	
dif loneliness	Samegen	Together	RIFG~RTPJ	-0.0022	-0.0063	0.0019	
dif loneliness	Intergen	Alone	LIFG~RIFG	0.0016	-0.0039	0.0071	

S5 Appendix – Table N. HbO. Contrasts pertaining to loneliness (dif) ~ INS relationship in unstandardised units. Contrasts are presented in the following order: Group, task, and interaction contrasts.

Measure	Group	Task	ROI Pairs	Estimate	Lower HPD	Upper HPD	No overlap
Group contrast							
dif loneliness	Intergen-Samegen		LIFG~RIFG	0.0004	-0.0042	0.0048	
dif loneliness	Intergen-Samegen		LIFG~LIFG	-0.0005	-0.0062	0.0053	
dif loneliness	Intergen-Samegen		LTPJ~LTPJ	-0.0063	-0.0129	0.0005	
dif loneliness	Intergen-Samegen		LIFG~LTPJ	-0.0031	-0.0076	0.0014	
dif loneliness	Intergen-Samegen		LIFG~RTPJ	0.0021	-0.0032	0.0072	
dif loneliness	Intergen-Samegen		RIFG~RIFG	-0.0016	-0.0082	0.0051	
dif loneliness	Intergen-Samegen		RTPJ~RTPJ	-0.0058	-0.0177	0.0057	
dif loneliness	Intergen-Samegen		RIFG~LTPJ	-0.0015	-0.0058	0.0028	
dif loneliness	Intergen-Samegen		RIFG~RTPJ	-0.0031	-0.0092	0.0030	
Task contrast							
dif loneliness		Alone-Together	LIFG~RIFG	0.0027	-0.0015	0.0069	
dif loneliness		Alone-Together	LIFG~LIFG	-0.0017	-0.0071	0.0037	
dif loneliness		Alone-Together	LTPJ~LTPJ	-0.0045	-0.0101	0.0014	
dif loneliness		Alone-Together	LIFG~LTPJ	0.0003	-0.0039	0.0043	
dif loneliness		Alone-Together	LIFG~RTPJ	0.0034	-0.0017	0.0083	
dif loneliness		Alone-Together	RIFG~RIFG	0.0081	0.0019	0.0143	No overlap
dif loneliness		Alone-Together	RTPJ~RTPJ	-0.0053	-0.0164	0.0056	
dif loneliness		Alone-Together	RIFG~LTPJ	0.0012	-0.0029	0.0052	
dif loneliness		Alone-Together	RIFG~RTPJ	0.0047	-0.0006	0.0100	
Interactions							
dif loneliness	Intergen-Samegen	Alone	LIFG~RIFG	0.0008	-0.0058	0.0078	
dif loneliness	Intergen-Samegen	Together	LIFG~RIFG	0.0000	-0.0056	0.0055	
dif loneliness	Intergen-Samegen	Alone	LIFG~LIFG	-0.0047	-0.0133	0.0043	
dif loneliness	Intergen-Samegen	Together	LIFG~LIFG	0.0037	-0.0037	0.0104	
dif loneliness	Intergen-Samegen	Alone	LTPJ~LTPJ	-0.0111	-0.0205	-0.0011	No overlap
dif loneliness	Intergen-Samegen	Together	LTPJ~LTPJ	-0.0016	-0.0094	0.0066	
dif loneliness	Intergen-Samegen	Alone	LIFG~LTPJ	-0.0008	-0.0073	0.0059	
dif loneliness	Intergen-Samegen	Together	LIFG~LTPJ	-0.0053	-0.0106	0.0002	
dif loneliness	Intergen-Samegen	Alone	LIFG~RTPJ	0.0040	-0.0040	0.0119	
dif loneliness	Intergen-Samegen	Together	LIFG~RTPJ	0.0003	-0.0060	0.0069	
dif loneliness	Intergen-Samegen	Alone	RIFG~RIFG	0.0001	-0.0099	0.0099	
dif loneliness	Intergen-Samegen	Together	RIFG~RIFG	-0.0034	-0.0112	0.0046	
dif loneliness	Intergen-Samegen	Alone	RTPJ~RTPJ	-0.0060	-0.0238	0.0112	
dif loneliness	Intergen-Samegen	Together	RTPJ~RTPJ	-0.0056	-0.0199	0.0092	
dif loneliness	Intergen-Samegen	Alone	RIFG~LTPJ	-0.0015	-0.0080	0.0050	
dif loneliness	Intergen-Samegen	Together	RIFG~LTPJ	-0.0016	-0.0068	0.0038	
dif loneliness	Intergen-Samegen	Alone	RIFG~RTPJ	-0.0050	-0.0137	0.0038	
dif loneliness	Intergen-Samegen	Together	RIFG~RTPJ	-0.0011	-0.0084	0.0062	
dif loneliness	Intergen	Alone-Together	LIFG~RIFG	0.0031	-0.0039	0.0101	
dif loneliness	Samegen	Alone-Together	LIFG~RIFG	0.0023	-0.0026	0.0072	
dif loneliness	Intergen	Alone-Together	LIFG~LIFG	-0.0058	-0.0142	0.0033	
dif loneliness	Samegen	Alone-Together	LIFG~LIFG	0.0024	-0.0041	0.0089	

Measure	Group	Task	ROI Pairs	Estimate	Lower HPD	Upper HPD	No overlap
dif loneliness	Intergen	Alone-Together	LTPJ~LTPJ	-0.0092	-0.0186	0.0000	
dif loneliness	Samegen	Alone-Together	LTPJ~LTPJ	0.0002	-0.0066	0.0070	
dif loneliness	Intergen	Alone-Together	LIFG~LTPJ	0.0026	-0.0040	0.0091	
dif loneliness	Samegen	Alone-Together	LIFG~LTPJ	-0.0019	-0.0068	0.0029	
dif loneliness	Intergen	Alone-Together	LIFG~RTPJ	0.0052	-0.0028	0.0133	
dif loneliness	Samegen	Alone-Together	LIFG~RTPJ	0.0015	-0.0042	0.0072	
dif loneliness	Intergen	Alone-Together	RIFG~RIFG	0.0098	-0.0006	0.0198	
dif loneliness	Samegen	Alone-Together	RIFG~RIFG	0.0063	-0.0003	0.0133	
dif loneliness	Intergen	Alone-Together	RTPJ~RTPJ	-0.0055	-0.0242	0.0132	
dif loneliness	Samegen	Alone-Together	RTPJ~RTPJ	-0.0051	-0.0170	0.0067	
dif loneliness	Intergen	Alone-Together	RIFG~LTPJ	0.0013	-0.0053	0.0080	
dif loneliness	Samegen	Alone-Together	RIFG~LTPJ	0.0012	-0.0035	0.0059	
dif loneliness	Intergen	Alone-Together	RIFG~RTPJ	0.0027	-0.0060	0.0112	
dif loneliness	Samegen	Alone-Together	RIFG~RTPJ	0.0066	0.0007	0.0126	No overlap

S5 Appendix – Table O. HbO. Relationships between social closeness (sum) and INS in unstandardised units. Effects are presented in the following order: Main, group, task, and simple effects.

Measure	Group	Task	ROI Pairs	Estimate	Lower HPD	Upper HPD	No overlap
Main effect							
sum social closeness			LIFG~RIFG	0.0007	-0.0006	0.0021	
sum social closeness			LIFG~LIFG	-0.0007	-0.0025	0.0011	
sum social closeness			LTPJ~LTPJ	0.0005	-0.0015	0.0025	
sum social closeness			LIFG~LTPJ	0.0003	-0.0011	0.0016	
sum social closeness			LIFG~RTPJ	0	-0.0016	0.0017	
sum social closeness			RIFG~RIFG	0	-0.002	0.002	
sum social closeness			RTPJ~RTPJ	-0.0012	-0.0047	0.0023	
sum social closeness			RIFG~LTPJ	0.0006	-0.0007	0.0019	
sum social closeness			RIFG~RTPJ	0.0021	0.0004	0.0039	No overlap
Group effect							
sum social closeness	Intergen		LIFG~RIFG	0.0006	-0.001	0.0022	
sum social closeness	Samegen		LIFG~RIFG	0.0009	-0.0014	0.0033	
sum social closeness	Intergen		LIFG~LIFG	-0.0016	-0.0036	0.0003	
sum social closeness	Samegen		LIFG~LIFG	0.0002	-0.0027	0.0032	
sum social closeness	Intergen		LTPJ~LTPJ	-0.0005	-0.0029	0.0018	
sum social closeness	Samegen		LTPJ~LTPJ	0.0015	-0.0018	0.0049	
sum social closeness	Intergen		LIFG~LTPJ	0.0004	-0.0012	0.0018	
sum social closeness	Samegen		LIFG~LTPJ	0.0002	-0.0021	0.0025	
sum social closeness	Intergen		LIFG~RTPJ	-0.0006	-0.0025	0.0012	
sum social closeness	Samegen		LIFG~RTPJ	0.0006	-0.0021	0.0032	
sum social closeness	Intergen		RIFG~RIFG	0.0022	-0.0001	0.0044	
sum social closeness	Samegen		RIFG~RIFG	-0.0021	-0.0054	0.0012	
sum social closeness	Intergen		RTPJ~RTPJ	0.0001	-0.0039	0.0043	
sum social closeness	Samegen		RTPJ~RTPJ	-0.0025	-0.0081	0.0033	
sum social closeness	Intergen		RIFG~LTPJ	0.0006	-0.0008	0.0021	
sum social closeness	Samegen		RIFG~LTPJ	0.0005	-0.0017	0.0027	
sum social closeness	Intergen		RIFG~RTPJ	-0.0001	-0.0022	0.0019	
sum social closeness	Samegen		RIFG~RTPJ	0.0044	0.0016	0.0073	No overlap
Task effect							
sum social closeness		Alone	LIFG~RIFG	0.0011	-0.001	0.0033	
sum social closeness		Together	LIFG~RIFG	0.0004	-0.0013	0.0021	
sum social closeness		Alone	LIFG~LIFG	-0.0029	-0.0057	-0.0003	No overlap
sum social closeness		Together	LIFG~LIFG	0.0015	-0.0007	0.0036	
sum social closeness		Alone	LTPJ~LTPJ	0.0005	-0.0025	0.0035	
sum social closeness		Together	LTPJ~LTPJ	0.0005	-0.0019	0.0029	
sum social closeness		Alone	LIFG~LTPJ	-0.0001	-0.0022	0.0019	
sum social closeness		Together	LIFG~LTPJ	0.0007	-0.001	0.0023	
sum social closeness		Alone	LIFG~RTPJ	0.0009	-0.0016	0.0032	
sum social closeness		Together	LIFG~RTPJ	-0.0009	-0.0029	0.0011	
sum social closeness		Alone	RIFG~RIFG	-0.0011	-0.0041	0.0019	
sum social closeness		Together	RIFG~RIFG	0.0012	-0.0012	0.0037	
sum social closeness		Alone	RTPJ~RTPJ	0.001	-0.0042	0.0063	

Measure	Group	Task	ROI Pairs	Estimate	Lower HPD	Upper HPD	No overlap
sum social closeness		Together	RTPJ~RTPJ	-0.0033	-0.0078	0.001	
sum social closeness		Alone	RIFG~LTPJ	0.0006	-0.0014	0.0027	
sum social closeness		Together	RIFG~LTPJ	0.0005	-0.001	0.0022	
sum social closeness		Alone	RIFG~RTPJ	0.0033	0.0006	0.0059	No overlap
sum social closeness		Together	RIFG~RTPJ	0.001	-0.0011	0.0031	
Simple effect							
sum social closeness	Intergen	Alone	LIFG~RIFG	0.0007	-0.0017	0.0030	
sum social closeness	Intergen	Together	LIFG~RIFG	0.0004	-0.0014	0.0023	
sum social closeness	Samegen	Alone	LIFG~RIFG	0.0015	-0.0020	0.0051	
sum social closeness	Samegen	Together	LIFG~RIFG	0.0003	-0.0025	0.0031	
sum social closeness	Intergen	Alone	LIFG~LIFG	-0.0029	-0.0058	0.0002	
sum social closeness	Intergen	Together	LIFG~LIFG	-0.0003	-0.0026	0.0021	
sum social closeness	Samegen	Alone	LIFG~LIFG	-0.0029	-0.0074	0.0018	
sum social closeness	Samegen	Together	LIFG~LIFG	0.0033	-0.0002	0.0069	
sum social closeness	Intergen	Alone	LTPJ~LTPJ	-0.0008	-0.0041	0.0027	
sum social closeness	Intergen	Together	LTPJ~LTPJ	-0.0002	-0.0029	0.0025	
sum social closeness	Samegen	Alone	LTPJ~LTPJ	0.0018	-0.0031	0.0068	
sum social closeness	Samegen	Together	LTPJ~LTPJ	0.0012	-0.0029	0.0051	
sum social closeness	Intergen	Alone	LIFG~LTPJ	-0.0005	-0.0027	0.0018	
sum social closeness	Intergen	Together	LIFG~LTPJ	0.0012	-0.0006	0.0030	
sum social closeness	Samegen	Alone	LIFG~LTPJ	0.0002	-0.0031	0.0038	
sum social closeness	Samegen	Together	LIFG~LTPJ	0.0001	-0.0026	0.0029	
sum social closeness	Intergen	Alone	LIFG~RTPJ	-0.0006	-0.0033	0.0021	
sum social closeness	Intergen	Together	LIFG~RTPJ	-0.0006	-0.0028	0.0017	
sum social closeness	Samegen	Alone	LIFG~RTPJ	0.0024	-0.0017	0.0064	
sum social closeness	Samegen	Together	LIFG~RTPJ	-0.0012	-0.0045	0.0020	
sum social closeness	Intergen	Alone	RIFG~RIFG	0.0015	-0.0020	0.0047	
sum social closeness	Intergen	Together	RIFG~RIFG	0.0029	0.0001	0.0054	No overlap
sum social closeness	Samegen	Alone	RIFG~RIFG	-0.0037	-0.0087	0.0013	
sum social closeness	Samegen	Together	RIFG~RIFG	-0.0004	-0.0044	0.0036	
sum social closeness	Intergen	Alone	RTPJ~RTPJ	0.0011	-0.0049	0.0067	
sum social closeness	Intergen	Together	RTPJ~RTPJ	-0.0009	-0.0064	0.0046	
sum social closeness	Samegen	Alone	RTPJ~RTPJ	0.0008	-0.0078	0.0097	
sum social closeness	Samegen	Together	RTPJ~RTPJ	-0.0058	-0.0126	0.0013	
sum social closeness	Intergen	Alone	RIFG~LTPJ	-0.0001	-0.0024	0.0021	
sum social closeness	Intergen	Together	RIFG~LTPJ	0.0013	-0.0004	0.0031	
sum social closeness	Samegen	Alone	RIFG~LTPJ	0.0014	-0.0020	0.0047	
sum social closeness	Samegen	Together	RIFG~LTPJ	-0.0003	-0.0029	0.0025	
sum social closeness	Intergen	Alone	RIFG~RTPJ	-0.0008	-0.0038	0.0021	
sum social closeness	Intergen	Together	RIFG~RTPJ	0.0006	-0.0019	0.0030	
sum social closeness	Samegen	Alone	RIFG~RTPJ	0.0073	0.0031	0.0117	No overlap
sum social closeness	Samegen	Together	RIFG~RTPJ	0.0014	-0.0019	0.0049	
sum social closeness	Intergen	Alone	LIFG~RIFG	0.0007	-0.0017	0.0030	

S5 Appendix – Table P. HbO. Contrasts pertaining to social closeness (sum) ~ INS relationship in unstandardised units. Contrasts are presented in the following order: Group, task, and interaction contrasts.

Measure	Group	Task	ROI Pairs	Estimate	Lower HPD	Upper HPD	No overlap
Group contrast							
sum social closeness	Int-Samegen		LIFG~RIFG	-0.0004	-0.0032	0.0024	
sum social closeness	Int-Samegen		LIFG~LIFG	-0.0018	-0.0054	0.0018	
sum social closeness	Int-Samegen		LTPJ~LTPJ	-0.0020	-0.0060	0.0021	
sum social closeness	Int-Samegen		LIFG~LTPJ	0.0002	-0.0025	0.0030	
sum social closeness	Int-Samegen		LIFG~RTPJ	-0.0012	-0.0044	0.0022	
sum social closeness	Int-Samegen		RIFG~RIFG	0.0042	0.0003	0.0083	No overlap
sum social closeness	Int-Samegen		RTPJ~RTPJ	0.0026	-0.0045	0.0095	
sum social closeness	Int-Samegen		RIFG~LTPJ	0.0001	-0.0026	0.0027	
sum social closeness	Int-Samegen		RIFG~RTPJ	-0.0045	-0.0079	-0.0009	No overlap
Task contrast							
sum social closeness		Alone-Together	LIFG~RIFG	0.0007	-0.0019	0.0034	
sum social closeness		Alone-Together	LIFG~LIFG	-0.0044	-0.0079	-0.0010	No overlap
sum social closeness		Alone-Together	LTPJ~LTPJ	0.0000	-0.0036	0.0035	
sum social closeness		Alone-Together	LIFG~LTPJ	-0.0008	-0.0034	0.0017	
sum social closeness		Alone-Together	LIFG~RTPJ	0.0018	-0.0012	0.0048	
sum social closeness		Alone-Together	RIFG~RIFG	-0.0024	-0.0062	0.0014	
sum social closeness		Alone-Together	RTPJ~RTPJ	0.0043	-0.0026	0.0109	
sum social closeness		Alone-Together	RIFG~LTPJ	0.0001	-0.0024	0.0027	
sum social closeness		Alone-Together	RIFG~RTPJ	0.0022	-0.0010	0.0054	
Interactions							
sum social closeness	Int-Samegen	Alone	LIFG~RIFG	-0.0008	-0.0050	0.0037	
sum social closeness	Int-Samegen	Together	LIFG~RIFG	0.0001	-0.0032	0.0034	
sum social closeness	Int-Samegen	Alone	LIFG~LIFG	0.0000	-0.0056	0.0054	
sum social closeness	Int-Samegen	Together	LIFG~LIFG	-0.0037	-0.0080	0.0006	
sum social closeness	Int-Samegen	Alone	LTPJ~LTPJ	-0.0026	-0.0085	0.0035	
sum social closeness	Int-Samegen	Together	LTPJ~LTPJ	-0.0015	-0.0063	0.0034	
sum social closeness	Int-Samegen	Alone	LIFG~LTPJ	-0.0007	-0.0047	0.0036	
sum social closeness	Int-Samegen	Together	LIFG~LTPJ	0.0011	-0.0023	0.0044	
sum social closeness	Int-Samegen	Alone	LIFG~RTPJ	-0.0030	-0.0080	0.0018	
sum social closeness	Int-Samegen	Together	LIFG~RTPJ	0.0006	-0.0034	0.0044	
sum social closeness	Int-Samegen	Alone	RIFG~RIFG	0.0052	-0.0008	0.0112	
sum social closeness	Int-Samegen	Together	RIFG~RIFG	0.0033	-0.0014	0.0082	
sum social closeness	Int-Samegen	Alone	RTPJ~RTPJ	0.0003	-0.0105	0.0105	
sum social closeness	Int-Samegen	Together	RTPJ~RTPJ	0.0050	-0.0041	0.0136	
sum social closeness	Int-Samegen	Alone	RIFG~LTPJ	-0.0014	-0.0056	0.0025	
sum social closeness	Int-Samegen	Together	RIFG~LTPJ	0.0016	-0.0015	0.0049	
sum social closeness	Int-Samegen	Alone	RIFG~RTPJ	-0.0081	-0.0132	-0.0026	No overlap
sum social closeness	Int-Samegen	Together	RIFG~RTPJ	-0.0008	-0.0050	0.0034	
sum social closeness	Intergen	Alone-Together	LIFG~RIFG	0.0003	-0.0025	0.0033	
sum social closeness	Samegen	Alone-Together	LIFG~RIFG	0.0012	-0.0033	0.0055	
sum social closeness	Intergen	Alone-Together	LIFG~LIFG	-0.0026	-0.0062	0.0012	
sum social closeness	Samegen	Alone-Together	LIFG~LIFG	-0.0063	-0.0120	-0.0006	No overlap

Measure	Group	Task	ROI Pairs	Estimate	Lower HPD	Upper HPD	No overlap
sum social closeness	Intergen	Alone-Together	LTPJ~LTPJ	-0.0005	-0.0044	0.0034	
sum social closeness	Samegen	Alone-Together	LTPJ~LTPJ	0.0006	-0.0054	0.0066	
sum social closeness	Intergen	Alone-Together	LIFG~LTPJ	-0.0017	-0.0044	0.0011	
sum social closeness	Samegen	Alone-Together	LIFG~LTPJ	0.0001	-0.0042	0.0043	
sum social closeness	Intergen	Alone-Together	LIFG~RTPJ	0.0000	-0.0033	0.0035	
sum social closeness	Samegen	Alone-Together	LIFG~RTPJ	0.0036	-0.0014	0.0086	
sum social closeness	Intergen	Alone-Together	RIFG~RIFG	-0.0014	-0.0056	0.0026	
sum social closeness	Samegen	Alone-Together	RIFG~RIFG	-0.0033	-0.0095	0.0029	
sum social closeness	Intergen	Alone-Together	RTPJ~RTPJ	0.0020	-0.0057	0.0101	
sum social closeness	Samegen	Alone-Together	RTPJ~RTPJ	0.0066	-0.0043	0.0175	
sum social closeness	Intergen	Alone-Together	RIFG~LTPJ	-0.0014	-0.0042	0.0014	
sum social closeness	Samegen	Alone-Together	RIFG~LTPJ	0.0016	-0.0028	0.0057	
sum social closeness	Intergen	Alone-Together	RIFG~RTPJ	-0.0014	-0.0050	0.0023	
sum social closeness	Samegen	Alone-Together	RIFG~RTPJ	0.0059	0.0007	0.0113	No overlap

S5 Appendix – Table Q. HbO. Relationships between social closeness (dif) and INS in unstandardised units. Effects are presented in the following order: Main, group, task, and simple effects.

Measure	Group	Task	ROI Pairs	Estimate	Lower HPD	Upper HPD	No overlap
Main effect							
dif social closeness			LIFG~RIFG	-0.0004	-0.0027	0.0018	
dif social closeness			LIFG~LIFG	-0.0003	-0.0031	0.0025	
dif social closeness			LTPJ~LTPJ	-0.001	-0.0042	0.0021	
dif social closeness			LIFG~LTPJ	-0.0005	-0.0026	0.0017	
dif social closeness			LIFG~RTPJ	-0.0014	-0.0039	0.0012	
dif social closeness			RIFG~RIFG	0.0018	-0.0015	0.005	
dif social closeness			RTPJ~RTPJ	0.0018	-0.0035	0.007	
dif social closeness			RIFG~LTPJ	0.0011	-0.001	0.0032	
dif social closeness			RIFG~RTPJ	-0.002	-0.0048	0.0009	
Group effect							
dif social closeness	Intergen		LIFG~RIFG	-0.0002	-0.0032	0.0027	
dif social closeness	Samegen		LIFG~RIFG	-0.0005	-0.004	0.0028	
dif social closeness	Intergen		LIFG~LIFG	-0.0011	-0.0048	0.0025	
dif social closeness	Samegen		LIFG~LIFG	0.0005	-0.0037	0.0047	
dif social closeness	Intergen		LTPJ~LTPJ	-0.0005	-0.0047	0.0038	
dif social closeness	Samegen		LTPJ~LTPJ	-0.0015	-0.0063	0.0033	
dif social closeness	Intergen		LIFG~LTPJ	-0.0004	-0.0032	0.0026	
dif social closeness	Samegen		LIFG~LTPJ	-0.0005	-0.0038	0.0027	
dif social closeness	Intergen		LIFG~RTPJ	-0.0027	-0.0061	0.0008	
dif social closeness	Samegen		LIFG~RTPJ	0	-0.0039	0.0035	
dif social closeness	Intergen		RIFG~RIFG	0.0004	-0.0039	0.0046	
dif social closeness	Samegen		RIFG~RIFG	0.0031	-0.0016	0.008	
dif social closeness	Intergen		RTPJ~RTPJ	0.0005	-0.0073	0.0086	
dif social closeness	Samegen		RTPJ~RTPJ	0.0032	-0.0038	0.0103	
dif social closeness	Intergen		RIFG~LTPJ	0.0013	-0.0014	0.004	
dif social closeness	Samegen		RIFG~LTPJ	0.0009	-0.0023	0.0041	
dif social closeness	Intergen		RIFG~RTPJ	-0.0016	-0.0055	0.0023	
dif social closeness	Samegen		RIFG~RTPJ	-0.0024	-0.0066	0.0018	
Task effect							
dif social closeness		Alone	LIFG~RIFG	-0.0007	-0.0042	0.0027	
dif social closeness		Together	LIFG~RIFG	-0.0001	-0.0027	0.0027	
dif social closeness		Alone	LIFG~LIFG	0.0013	-0.0028	0.0057	
dif social closeness		Together	LIFG~LIFG	-0.002	-0.0054	0.0014	
dif social closeness		Alone	LTPJ~LTPJ	-0.0002	-0.0049	0.0046	
dif social closeness		Together	LTPJ~LTPJ	-0.0019	-0.0056	0.002	
dif social closeness		Alone	LIFG~LTPJ	-0.0006	-0.0038	0.0027	
dif social closeness		Together	LIFG~LTPJ	-0.0003	-0.0028	0.0024	
dif social closeness		Alone	LIFG~RTPJ	-0.0023	-0.0062	0.0016	
dif social closeness		Together	LIFG~RTPJ	-0.0004	-0.0035	0.0026	
dif social closeness		Alone	RIFG~RIFG	0.0024	-0.0026	0.0072	
dif social closeness		Together	RIFG~RIFG	0.0012	-0.0027	0.0051	
dif social closeness		Alone	RTPJ~RTPJ	-0.0005	-0.0085	0.0073	

Measure	Group	Task	ROI Pairs	Estimate	Lower HPD	Upper HPD	No overlap
dif social closeness		Together	RTPJ~RTPJ	0.0041	-0.0022	0.0109	
dif social closeness		Alone	RIFG~LTPJ	0.0014	-0.0019	0.0048	
dif social closeness		Together	RIFG~LTPJ	0.0008	-0.0017	0.0034	
dif social closeness		Alone	RIFG~RTPJ	-0.0037	-0.008	0.0006	
dif social closeness		Together	RIFG~RTPJ	-0.0004	-0.0038	0.003	
Simple effect							
dif social closeness	Intergen	Alone	LIFG~RIFG	-0.0019	-0.0063	0.0027	
dif social closeness	Intergen	Together	LIFG~RIFG	0.0014	-0.0020	0.0049	
dif social closeness	Samegen	Alone	LIFG~RIFG	0.0005	-0.0047	0.0056	
dif social closeness	Samegen	Together	LIFG~RIFG	-0.0017	-0.0058	0.0024	
dif social closeness	Intergen	Alone	LIFG~LIFG	-0.0019	-0.0077	0.0037	
dif social closeness	Intergen	Together	LIFG~LIFG	-0.0004	-0.0047	0.0040	
dif social closeness	Samegen	Alone	LIFG~LIFG	0.0045	-0.0018	0.0109	
dif social closeness	Samegen	Together	LIFG~LIFG	-0.0035	-0.0088	0.0014	
dif social closeness	Intergen	Alone	LTPJ~LTPJ	0.0002	-0.0059	0.0065	
dif social closeness	Intergen	Together	LTPJ~LTPJ	-0.0013	-0.0062	0.0036	
dif social closeness	Samegen	Alone	LTPJ~LTPJ	-0.0006	-0.0077	0.0065	
dif social closeness	Samegen	Together	LTPJ~LTPJ	-0.0025	-0.0081	0.0033	
dif social closeness	Intergen	Alone	LIFG~LTPJ	0.0002	-0.0042	0.0045	
dif social closeness	Intergen	Together	LIFG~LTPJ	-0.0008	-0.0042	0.0026	
dif social closeness	Samegen	Alone	LIFG~LTPJ	-0.0014	-0.0062	0.0035	
dif social closeness	Samegen	Together	LIFG~LTPJ	0.0003	-0.0036	0.0042	
dif social closeness	Intergen	Alone	LIFG~RTPJ	-0.0031	-0.0083	0.0022	
dif social closeness	Intergen	Together	LIFG~RTPJ	-0.0024	-0.0065	0.0018	
dif social closeness	Samegen	Alone	LIFG~RTPJ	-0.0016	-0.0071	0.0042	
dif social closeness	Samegen	Together	LIFG~RTPJ	0.0015	-0.0028	0.0060	
dif social closeness	Intergen	Alone	RIFG~RIFG	-0.0037	-0.0102	0.0027	
dif social closeness	Intergen	Together	RIFG~RIFG	0.0044	-0.0005	0.0095	
dif social closeness	Samegen	Alone	RIFG~RIFG	0.0084	0.0012	0.0158	No overlap
dif social closeness	Samegen	Together	RIFG~RIFG	-0.0021	-0.0080	0.0039	
dif social closeness	Intergen	Alone	RTPJ~RTPJ	-0.0015	-0.0138	0.0099	
dif social closeness	Intergen	Together	RTPJ~RTPJ	0.0023	-0.0081	0.0122	
dif social closeness	Samegen	Alone	RTPJ~RTPJ	0.0005	-0.0102	0.0108	
dif social closeness	Samegen	Together	RTPJ~RTPJ	0.0059	-0.0023	0.0143	
dif social closeness	Intergen	Alone	RIFG~LTPJ	0.0000	-0.0042	0.0043	
dif social closeness	Intergen	Together	RIFG~LTPJ	0.0026	-0.0005	0.0060	
dif social closeness	Samegen	Alone	RIFG~LTPJ	0.0027	-0.0021	0.0078	
dif social closeness	Samegen	Together	RIFG~LTPJ	-0.0010	-0.0048	0.0029	
dif social closeness	Intergen	Alone	RIFG~RTPJ	-0.0029	-0.0087	0.0029	
dif social closeness	Intergen	Together	RIFG~RTPJ	-0.0004	-0.0049	0.0043	
dif social closeness	Samegen	Alone	RIFG~RTPJ	-0.0044	-0.0107	0.0020	
dif social closeness	Samegen	Together	RIFG~RTPJ	-0.0004	-0.0053	0.0046	
dif social closeness	Intergen	Alone	LIFG~RIFG	-0.0019	-0.0063	0.0027	

S5 Appendix – Table R. HbO. Contrasts pertaining to social closeness (dif) ~ INS relationship in unstandardised units. Contrasts are presented in the following order: Group, task, and interaction contrasts.

Measure	Group	Task	ROI Pairs	Estimate	Lower HPD	Upper HPD	No overlap
Group contrast							
dif social closeness	Int-Samegen		LIFG~RIFG	0.0003	-0.0041	0.0048	
dif social closeness	Int-Samegen		LIFG~LIFG	-0.0016	-0.0072	0.0040	
dif social closeness	Int-Samegen		LTPJ~LTPJ	0.0010	-0.0054	0.0073	
dif social closeness	Int-Samegen		LIFG~LTPJ	0.0002	-0.0041	0.0046	
dif social closeness	Int-Samegen		LIFG~RTPJ	-0.0027	-0.0077	0.0024	
dif social closeness	Int-Samegen		RIFG~RIFG	-0.0028	-0.0091	0.0037	
dif social closeness	Int-Samegen		RTPJ~RTPJ	-0.0028	-0.0136	0.0079	
dif social closeness	Int-Samegen		RIFG~LTPJ	0.0004	-0.0037	0.0045	
dif social closeness	Int-Samegen		RIFG~RTPJ	0.0007	-0.0050	0.0064	
Task contrast							
dif social closeness		Alone-Together	LIFG~RIFG	-0.0005	-0.0047	0.0037	
dif social closeness		Alone-Together	LIFG~LIFG	0.0033	-0.0021	0.0085	
dif social closeness		Alone-Together	LTPJ~LTPJ	0.0017	-0.0040	0.0073	
dif social closeness		Alone-Together	LIFG~LTPJ	-0.0003	-0.0043	0.0037	
dif social closeness		Alone-Together	LIFG~RTPJ	-0.0019	-0.0067	0.0028	
dif social closeness		Alone-Together	RIFG~RIFG	0.0012	-0.0049	0.0073	
dif social closeness		Alone-Together	RTPJ~RTPJ	-0.0046	-0.0145	0.0054	
dif social closeness		Alone-Together	RIFG~LTPJ	0.0005	-0.0035	0.0047	
dif social closeness		Alone-Together	RIFG~RTPJ	-0.0033	-0.0084	0.0020	
Interactions							
dif social closeness	Int-Samegen	Alone	LIFG~RIFG	-0.0024	-0.0093	0.0044	
dif social closeness	Int-Samegen	Together	LIFG~RIFG	0.0031	-0.0022	0.0086	
dif social closeness	Int-Samegen	Alone	LIFG~LIFG	-0.0064	-0.0150	0.0023	
dif social closeness	Int-Samegen	Together	LIFG~LIFG	0.0032	-0.0034	0.0101	
dif social closeness	Int-Samegen	Alone	LTPJ~LTPJ	0.0008	-0.0084	0.0103	
dif social closeness	Int-Samegen	Together	LTPJ~LTPJ	0.0012	-0.0063	0.0085	
dif social closeness	Int-Samegen	Alone	LIFG~LTPJ	0.0016	-0.0049	0.0081	
dif social closeness	Int-Samegen	Together	LIFG~LTPJ	-0.0011	-0.0064	0.0041	
dif social closeness	Int-Samegen	Alone	LIFG~RTPJ	-0.0014	-0.0091	0.0062	
dif social closeness	Int-Samegen	Together	LIFG~RTPJ	-0.0039	-0.0098	0.0021	
dif social closeness	Int-Samegen	Alone	RIFG~RIFG	-0.0121	-0.0221	-0.0026	No overlap
dif social closeness	Int-Samegen	Together	RIFG~RIFG	0.0066	-0.0011	0.0144	
dif social closeness	Int-Samegen	Alone	RTPJ~RTPJ	-0.0020	-0.0180	0.0138	
dif social closeness	Int-Samegen	Together	RTPJ~RTPJ	-0.0036	-0.0167	0.0095	
dif social closeness	Int-Samegen	Alone	RIFG~LTPJ	-0.0028	-0.0094	0.0034	
dif social closeness	Int-Samegen	Together	RIFG~LTPJ	0.0036	-0.0015	0.0086	
dif social closeness	Int-Samegen	Alone	RIFG~RTPJ	0.0015	-0.0073	0.0097	
dif social closeness	Int-Samegen	Together	RIFG~RTPJ	0.0000	-0.0068	0.0068	
dif social closeness	Intergen	Alone-Together	LIFG~RIFG	-0.0033	-0.0086	0.0023	
dif social closeness	Samegen	Alone-Together	LIFG~RIFG	0.0022	-0.0040	0.0087	
dif social closeness	Intergen	Alone-Together	LIFG~LIFG	-0.0015	-0.0084	0.0057	
dif social closeness	Samegen	Alone-Together	LIFG~LIFG	0.0081	0.0000	0.0159	

Measure	Group	Task	ROI Pairs	Estimate	Lower HPD	Upper HPD	No overlap
dif social closeness	Intergen	Alone-Together	LTPJ~LTPJ	0.0015	-0.0060	0.0086	
dif social closeness	Samegen	Alone-Together	LTPJ~LTPJ	0.0019	-0.0065	0.0107	
dif social closeness	Intergen	Alone-Together	LIFG~LTPJ	0.0010	-0.0042	0.0062	
dif social closeness	Samegen	Alone-Together	LIFG~LTPJ	-0.0017	-0.0077	0.0044	
dif social closeness	Intergen	Alone-Together	LIFG~RTPJ	-0.0007	-0.0072	0.0058	
dif social closeness	Samegen	Alone-Together	LIFG~RTPJ	-0.0031	-0.0101	0.0036	
dif social closeness	Intergen	Alone-Together	RIFG~RIFG	-0.0081	-0.0160	-0.0002	No overlap
dif social closeness	Samegen	Alone-Together	RIFG~RIFG	0.0105	0.0013	0.0198	No overlap
dif social closeness	Intergen	Alone-Together	RTPJ~RTPJ	-0.0038	-0.0192	0.0111	
dif social closeness	Samegen	Alone-Together	RTPJ~RTPJ	-0.0054	-0.0182	0.0070	
dif social closeness	Intergen	Alone-Together	RIFG~LTPJ	-0.0026	-0.0079	0.0026	
dif social closeness	Samegen	Alone-Together	RIFG~LTPJ	0.0037	-0.0025	0.0098	
dif social closeness	Intergen	Alone-Together	RIFG~RTPJ	-0.0025	-0.0097	0.0042	
dif social closeness	Samegen	Alone-Together	RIFG~RTPJ	-0.0041	-0.0117	0.0035	

S5 Appendix – Table S. HbO. Relationships between allophilia (sum) and INS in unstandardised units. Effects are presented in the following order: Main, group, task, and simple effects.

Measure	Group	Task	ROI Pairs	Estimate	Lower HPD	Upper HPD	No overlap
Main effect							
sum allophilia			LIFG~RIFG	-0.0002	-0.0003	0	No overlap
sum allophilia			LIFG~LIFG	0.0001	-0.0001	0.0003	
sum allophilia			LTPJ~LTPJ	-0.0004	-0.0006	-0.0001	No overlap
sum allophilia			LIFG~LTPJ	-0.0001	-0.0003	0.0001	
sum allophilia			LIFG~RTPJ	0	-0.0002	0.0002	
sum allophilia			RIFG~RIFG	0	-0.0002	0.0002	
sum allophilia			RTPJ~RTPJ	0	-0.0004	0.0004	
sum allophilia			RIFG~LTPJ	-0.0001	-0.0002	0.0001	
sum allophilia			RIFG~RTPJ	0	-0.0003	0.0002	
Group effect							
sum allophilia	Intergen		LIFG~RIFG	-0.0001	-0.0003	0.0002	
sum allophilia	Samegen		LIFG~RIFG	-0.0003	-0.0005	-0.0001	No overlap
sum allophilia	Intergen		LIFG~LIFG	0.0001	-0.0002	0.0004	
sum allophilia	Samegen		LIFG~LIFG	0.0001	-0.0002	0.0004	
sum allophilia	Intergen		LTPJ~LTPJ	-0.0004	-0.0008	-0.0001	No overlap
sum allophilia	Samegen		LTPJ~LTPJ	-0.0003	-0.0007	0	
sum allophilia	Intergen		LIFG~LTPJ	-0.0001	-0.0004	0.0001	
sum allophilia	Samegen		LIFG~LTPJ	-0.0001	-0.0003	0.0002	
sum allophilia	Intergen		LIFG~RTPJ	0.0001	-0.0002	0.0003	
sum allophilia	Samegen		LIFG~RTPJ	-0.0001	-0.0004	0.0002	
sum allophilia	Intergen		RIFG~RIFG	0	-0.0003	0.0004	
sum allophilia	Samegen		RIFG~RIFG	-0.0001	-0.0004	0.0003	
sum allophilia	Intergen		RTPJ~RTPJ	0.0001	-0.0005	0.0007	
sum allophilia	Samegen		RTPJ~RTPJ	-0.0001	-0.0006	0.0004	
sum allophilia	Intergen		RIFG~LTPJ	-0.0001	-0.0003	0.0001	
sum allophilia	Samegen		RIFG~LTPJ	-0.0001	-0.0003	0.0001	
sum allophilia	Intergen		RIFG~RTPJ	0.0002	-0.0001	0.0005	
sum allophilia	Samegen		RIFG~RTPJ	-0.0003	-0.0006	0	
Task effect							
sum allophilia		Alone	LIFG~RIFG	-0.0002	-0.0004	0.0001	
sum allophilia		Together	LIFG~RIFG	-0.0002	-0.0004	0	
sum allophilia		Alone	LIFG~LIFG	0.0002	-0.0001	0.0005	
sum allophilia		Together	LIFG~LIFG	0.0001	-0.0002	0.0003	
sum allophilia		Alone	LTPJ~LTPJ	-0.0004	-0.0007	-0.0001	No overlap
sum allophilia		Together	LTPJ~LTPJ	-0.0003	-0.0006	0	No overlap
sum allophilia		Alone	LIFG~LTPJ	-0.0001	-0.0003	0.0002	
sum allophilia		Together	LIFG~LTPJ	-0.0001	-0.0003	0.0001	
sum allophilia		Alone	LIFG~RTPJ	-0.0001	-0.0004	0.0002	
sum allophilia		Together	LIFG~RTPJ	0.0001	-0.0002	0.0003	
sum allophilia		Alone	RIFG~RIFG	-0.0002	-0.0005	0.0002	
sum allophilia		Together	RIFG~RIFG	0.0001	-0.0001	0.0004	
sum allophilia		Alone	RTPJ~RTPJ	-0.0001	-0.0007	0.0005	

Measure	Group	Task	ROI Pairs	Estimate	Lower HPD	Upper HPD	No overlap
sum allophilia		Together	RTPJ~RTPJ	0.0001	-0.0004	0.0006	
sum allophilia		Alone	RIFG~LTPJ	-0.0001	-0.0003	0.0001	
sum allophilia		Together	RIFG~LTPJ	-0.0001	-0.0003	0.0001	
sum allophilia		Alone	RIFG~RTPJ	-0.0001	-0.0005	0.0002	
sum allophilia		Together	RIFG~RTPJ	0.0001	-0.0002	0.0003	
Simple effect							
sum allophilia	Intergen	Alone	LIFG~RIFG	0.0000	-0.0003	0.0003	
sum allophilia	Intergen	Together	LIFG~RIFG	-0.0001	-0.0004	0.0002	
sum allophilia	Samegen	Alone	LIFG~RIFG	-0.0003	-0.0007	0.0000	
sum allophilia	Samegen	Together	LIFG~RIFG	-0.0003	-0.0005	0.0000	
sum allophilia	Intergen	Alone	LIFG~LIFG	0.0001	-0.0004	0.0005	
sum allophilia	Intergen	Together	LIFG~LIFG	0.0001	-0.0002	0.0005	
sum allophilia	Samegen	Alone	LIFG~LIFG	0.0003	-0.0002	0.0007	
sum allophilia	Samegen	Together	LIFG~LIFG	0.0000	-0.0004	0.0003	
sum allophilia	Intergen	Alone	LTPJ~LTPJ	-0.0004	-0.0009	0.0001	
sum allophilia	Intergen	Together	LTPJ~LTPJ	-0.0004	-0.0008	0.0000	No overlap
sum allophilia	Samegen	Alone	LTPJ~LTPJ	-0.0004	-0.0009	0.0001	
sum allophilia	Samegen	Together	LTPJ~LTPJ	-0.0002	-0.0007	0.0002	
sum allophilia	Intergen	Alone	LIFG~LTPJ	0.0001	-0.0003	0.0004	
sum allophilia	Intergen	Together	LIFG~LTPJ	-0.0003	-0.0006	0.0000	No overlap
sum allophilia	Samegen	Alone	LIFG~LTPJ	-0.0002	-0.0006	0.0001	
sum allophilia	Samegen	Together	LIFG~LTPJ	0.0001	-0.0002	0.0003	
sum allophilia	Intergen	Alone	LIFG~RTPJ	0.0000	-0.0004	0.0004	
sum allophilia	Intergen	Together	LIFG~RTPJ	0.0002	-0.0002	0.0005	
sum allophilia	Samegen	Alone	LIFG~RTPJ	-0.0001	-0.0006	0.0002	
sum allophilia	Samegen	Together	LIFG~RTPJ	0.0000	-0.0003	0.0003	
sum allophilia	Intergen	Alone	RIFG~RIFG	-0.0001	-0.0006	0.0003	
sum allophilia	Intergen	Together	RIFG~RIFG	0.0002	-0.0002	0.0006	
sum allophilia	Samegen	Alone	RIFG~RIFG	-0.0002	-0.0007	0.0003	
sum allophilia	Samegen	Together	RIFG~RIFG	0.0001	-0.0003	0.0005	
sum allophilia	Intergen	Alone	RTPJ~RTPJ	0.0003	-0.0006	0.0012	
sum allophilia	Intergen	Together	RTPJ~RTPJ	-0.0001	-0.0009	0.0007	
sum allophilia	Samegen	Alone	RTPJ~RTPJ	-0.0005	-0.0013	0.0002	
sum allophilia	Samegen	Together	RTPJ~RTPJ	0.0004	-0.0003	0.0010	
sum allophilia	Intergen	Alone	RIFG~LTPJ	-0.0001	-0.0004	0.0002	
sum allophilia	Intergen	Together	RIFG~LTPJ	-0.0001	-0.0003	0.0002	
sum allophilia	Samegen	Alone	RIFG~LTPJ	-0.0001	-0.0004	0.0003	
sum allophilia	Samegen	Together	RIFG~LTPJ	-0.0001	-0.0003	0.0002	
sum allophilia	Intergen	Alone	RIFG~RTPJ	0.0001	-0.0003	0.0005	
sum allophilia	Intergen	Together	RIFG~RTPJ	0.0003	-0.0001	0.0006	
sum allophilia	Samegen	Alone	RIFG~RTPJ	-0.0004	-0.0008	0.0001	
sum allophilia	Samegen	Together	RIFG~RTPJ	-0.0002	-0.0005	0.0002	
sum allophilia	Intergen	Alone	LIFG~RIFG	0.0000	-0.0003	0.0003	

S5 Appendix – Table T. HbO. Contrasts pertaining to allophilia (sum) ~ INS relationship in unstandardised units. Contrasts are presented in the following order: Group, task, and interaction contrasts.

Measure	Group	Task	ROI Pairs	Estimate	Lower HPD	Upper HPD	No overlap
Group contrast							
sum allophilia	Int-Samegen		LIFG~RIFG	0.0002	-0.0001	0.0006	
sum allophilia	Int-Samegen		LIFG~LIFG	0.0000	-0.0005	0.0004	
sum allophilia	Int-Samegen		LTPJ~LTPJ	-0.0001	-0.0006	0.0004	
sum allophilia	Int-Samegen		LIFG~LTPJ	0.0000	-0.0004	0.0003	
sum allophilia	Int-Samegen		LIFG~RTPJ	0.0002	-0.0002	0.0005	
sum allophilia	Int-Samegen		RIFG~RIFG	0.0001	-0.0004	0.0006	
sum allophilia	Int-Samegen		RTPJ~RTPJ	0.0002	-0.0006	0.0010	
sum allophilia	Int-Samegen		RIFG~LTPJ	0.0000	-0.0003	0.0003	
sum allophilia	Int-Samegen		RIFG~RTPJ	0.0005	0.0000	0.0009	No overlap
Task contrast							
sum allophilia		Alone-Together	LIFG~RIFG	0.0000	-0.0003	0.0003	
sum allophilia		Alone-Together	LIFG~LIFG	0.0001	-0.0003	0.0005	
sum allophilia		Alone-Together	LTPJ~LTPJ	-0.0001	-0.0005	0.0003	
sum allophilia		Alone-Together	LIFG~LTPJ	0.0000	-0.0002	0.0003	
sum allophilia		Alone-Together	LIFG~RTPJ	-0.0001	-0.0005	0.0002	
sum allophilia		Alone-Together	RIFG~RIFG	-0.0003	-0.0007	0.0001	
sum allophilia		Alone-Together	RTPJ~RTPJ	-0.0003	-0.0010	0.0005	
sum allophilia		Alone-Together	RIFG~LTPJ	0.0000	-0.0003	0.0002	
sum allophilia		Alone-Together	RIFG~RTPJ	-0.0002	-0.0006	0.0002	
Interactions							
sum allophilia	Int-Samegen	Alone	LIFG~RIFG	0.0003	-0.0001	0.0008	
sum allophilia	Int-Samegen	Together	LIFG~RIFG	0.0001	-0.0002	0.0005	
sum allophilia	Int-Samegen	Alone	LIFG~LIFG	-0.0002	-0.0008	0.0004	
sum allophilia	Int-Samegen	Together	LIFG~LIFG	0.0001	-0.0004	0.0006	
sum allophilia	Int-Samegen	Alone	LTPJ~LTPJ	0.0000	-0.0007	0.0007	
sum allophilia	Int-Samegen	Together	LTPJ~LTPJ	-0.0002	-0.0007	0.0004	
sum allophilia	Int-Samegen	Alone	LIFG~LTPJ	0.0003	-0.0002	0.0008	
sum allophilia	Int-Samegen	Together	LIFG~LTPJ	-0.0004	-0.0008	0.0000	
sum allophilia	Int-Samegen	Alone	LIFG~RTPJ	0.0001	-0.0004	0.0007	
sum allophilia	Int-Samegen	Together	LIFG~RTPJ	0.0002	-0.0003	0.0006	
sum allophilia	Int-Samegen	Alone	RIFG~RIFG	0.0001	-0.0006	0.0007	
sum allophilia	Int-Samegen	Together	RIFG~RIFG	0.0001	-0.0004	0.0007	
sum allophilia	Int-Samegen	Alone	RTPJ~RTPJ	0.0009	-0.0003	0.0020	
sum allophilia	Int-Samegen	Together	RTPJ~RTPJ	-0.0004	-0.0014	0.0006	
sum allophilia	Int-Samegen	Alone	RIFG~LTPJ	-0.0001	-0.0005	0.0004	
sum allophilia	Int-Samegen	Together	RIFG~LTPJ	0.0000	-0.0003	0.0004	
sum allophilia	Int-Samegen	Alone	RIFG~RTPJ	0.0005	-0.0002	0.0011	
sum allophilia	Int-Samegen	Together	RIFG~RTPJ	0.0004	-0.0001	0.0009	
sum allophilia	Intergen	Alone-Together	LIFG~RIFG	0.0001	-0.0003	0.0005	
sum allophilia	Samegen	Alone-Together	LIFG~RIFG	-0.0001	-0.0005	0.0003	
sum allophilia	Intergen	Alone-Together	LIFG~LIFG	-0.0001	-0.0006	0.0005	
sum allophilia	Samegen	Alone-Together	LIFG~LIFG	0.0003	-0.0003	0.0008	

Measure	Group	Task	ROI Pairs	Estimate	Lower HPD	Upper HPD	No overlap
sum allophilia	Intergen	Alone-Together	LTPJ~LTPJ	0.0000	-0.0005	0.0005	
sum allophilia	Samegen	Alone-Together	LTPJ~LTPJ	-0.0001	-0.0007	0.0004	
sum allophilia	Intergen	Alone-Together	LIFG~LTPJ	0.0004	0.0000	0.0008	No overlap
sum allophilia	Samegen	Alone-Together	LIFG~LTPJ	-0.0003	-0.0007	0.0001	
sum allophilia	Intergen	Alone-Together	LIFG~RTPJ	-0.0002	-0.0006	0.0003	
sum allophilia	Samegen	Alone-Together	LIFG~RTPJ	-0.0001	-0.0006	0.0004	
sum allophilia	Intergen	Alone-Together	RIFG~RIFG	-0.0003	-0.0009	0.0002	
sum allophilia	Samegen	Alone-Together	RIFG~RIFG	-0.0003	-0.0009	0.0004	
sum allophilia	Intergen	Alone-Together	RTPJ~RTPJ	0.0004	-0.0008	0.0015	
sum allophilia	Samegen	Alone-Together	RTPJ~RTPJ	-0.0009	-0.0018	0.0000	
sum allophilia	Intergen	Alone-Together	RIFG~LTPJ	-0.0001	-0.0005	0.0003	
sum allophilia	Samegen	Alone-Together	RIFG~LTPJ	0.0000	-0.0004	0.0004	
sum allophilia	Intergen	Alone-Together	RIFG~RTPJ	-0.0002	-0.0007	0.0003	
sum allophilia	Samegen	Alone-Together	RIFG~RTPJ	-0.0002	-0.0007	0.0003	

S5 Appendix – Table U. HbO. Relationships between allophilia (dif) and INS in unstandardised units. Effects are presented in the following order: Main, group, task, and simple effects.

Measure	Group	Task	ROI Pairs	Estimate	Lower HPD	Upper HPD	No overlap
Main effect							
dif allophilia			LIFG~RIFG	0.0001	-0.0002	0.0003	
dif allophilia			LIFG~LIFG	0	-0.0002	0.0003	
dif allophilia			LTPJ~LTPJ	0.0002	-0.0002	0.0005	
dif allophilia			LIFG~LTPJ	-0.0001	-0.0004	0.0001	
dif allophilia			LIFG~RTPJ	-0.0001	-0.0004	0.0001	
dif allophilia			RIFG~RIFG	0.0001	-0.0002	0.0004	
dif allophilia			RTPJ~RTPJ	0.0001	-0.0003	0.0006	
dif allophilia			RIFG~LTPJ	-0.0001	-0.0003	0.0001	
dif allophilia			RIFG~RTPJ	0	-0.0003	0.0003	
Group effect							
dif allophilia	Intergen		LIFG~RIFG	0.0002	-0.0001	0.0005	
dif allophilia	Samegen		LIFG~RIFG	-0.0001	-0.0004	0.0003	
dif allophilia	Intergen		LIFG~LIFG	0.0001	-0.0003	0.0004	
dif allophilia	Samegen		LIFG~LIFG	0	-0.0004	0.0004	
dif allophilia	Intergen		LTPJ~LTPJ	0.0002	-0.0002	0.0006	
dif allophilia	Samegen		LTPJ~LTPJ	0.0001	-0.0003	0.0007	
dif allophilia	Intergen		LIFG~LTPJ	-0.0002	-0.0005	0.0001	
dif allophilia	Samegen		LIFG~LTPJ	-0.0001	-0.0004	0.0002	
dif allophilia	Intergen		LIFG~RTPJ	0	-0.0003	0.0003	
dif allophilia	Samegen		LIFG~RTPJ	-0.0002	-0.0006	0.0001	
dif allophilia	Intergen		RIFG~RIFG	0.0002	-0.0003	0.0006	
dif allophilia	Samegen		RIFG~RIFG	0.0001	-0.0004	0.0005	
dif allophilia	Intergen		RTPJ~RTPJ	0.0003	-0.0003	0.0009	
dif allophilia	Samegen		RTPJ~RTPJ	-0.0001	-0.0007	0.0006	
dif allophilia	Intergen		RIFG~LTPJ	-0.0003	-0.0005	0	
dif allophilia	Samegen		RIFG~LTPJ	0	-0.0003	0.0003	
dif allophilia	Intergen		RIFG~RTPJ	0.0001	-0.0003	0.0004	
dif allophilia	Samegen		RIFG~RTPJ	-0.0001	-0.0005	0.0003	
Task effect							
dif allophilia		Alone	LIFG~RIFG	0	-0.0003	0.0003	
dif allophilia		Together	LIFG~RIFG	0.0001	-0.0001	0.0004	
dif allophilia		Alone	LIFG~LIFG	0.0002	-0.0002	0.0006	
dif allophilia		Together	LIFG~LIFG	-0.0001	-0.0004	0.0003	
dif allophilia		Alone	LTPJ~LTPJ	0.0001	-0.0003	0.0006	
dif allophilia		Together	LTPJ~LTPJ	0.0002	-0.0002	0.0006	
dif allophilia		Alone	LIFG~LTPJ	-0.0001	-0.0004	0.0002	
dif allophilia		Together	LIFG~LTPJ	-0.0001	-0.0004	0.0001	
dif allophilia		Alone	LIFG~RTPJ	-0.0002	-0.0006	0.0001	
dif allophilia		Together	LIFG~RTPJ	0	-0.0003	0.0003	
dif allophilia		Alone	RIFG~RIFG	-0.0003	-0.0007	0.0002	
dif allophilia		Together	RIFG~RIFG	0.0005	0.0002	0.0009	No overlap
dif allophilia		Alone	RTPJ~RTPJ	0.0004	-0.0003	0.0011	

Measure	Group	Task	ROI Pairs	Estimate	Lower HPD	Upper HPD	No overlap
dif allophilia		Together	RTPJ~RTPJ	-0.0001	-0.0007	0.0005	
dif allophilia		Alone	RIFG~LTPJ	-0.0002	-0.0005	0.0001	
dif allophilia		Together	RIFG~LTPJ	-0.0001	-0.0004	0.0001	
dif allophilia		Alone	RIFG~RTPJ	-0.0001	-0.0004	0.0003	
dif allophilia		Together	RIFG~RTPJ	0	-0.0003	0.0003	
Simple effect							
dif allophilia	Intergen	Alone	LIFG~RIFG	0.0002	-0.0002	0.0006	
dif allophilia	Intergen	Together	LIFG~RIFG	0.0001	-0.0002	0.0005	
dif allophilia	Samegen	Alone	LIFG~RIFG	-0.0002	-0.0007	0.0002	
dif allophilia	Samegen	Together	LIFG~RIFG	0.0001	-0.0002	0.0005	
dif allophilia	Intergen	Alone	LIFG~LIFG	0.0002	-0.0004	0.0007	
dif allophilia	Intergen	Together	LIFG~LIFG	-0.0001	-0.0006	0.0004	
dif allophilia	Samegen	Alone	LIFG~LIFG	0.0002	-0.0004	0.0008	
dif allophilia	Samegen	Together	LIFG~LIFG	-0.0001	-0.0006	0.0004	
dif allophilia	Intergen	Alone	LTPJ~LTPJ	0.0002	-0.0004	0.0008	
dif allophilia	Intergen	Together	LTPJ~LTPJ	0.0002	-0.0004	0.0007	
dif allophilia	Samegen	Alone	LTPJ~LTPJ	0.0000	-0.0007	0.0007	
dif allophilia	Samegen	Together	LTPJ~LTPJ	0.0003	-0.0003	0.0009	
dif allophilia	Intergen	Alone	LIFG~LTPJ	-0.0002	-0.0006	0.0002	
dif allophilia	Intergen	Together	LIFG~LTPJ	-0.0002	-0.0005	0.0002	
dif allophilia	Samegen	Alone	LIFG~LTPJ	-0.0001	-0.0005	0.0004	
dif allophilia	Samegen	Together	LIFG~LTPJ	-0.0001	-0.0005	0.0002	
dif allophilia	Intergen	Alone	LIFG~RTPJ	0.0000	-0.0005	0.0004	
dif allophilia	Intergen	Together	LIFG~RTPJ	0.0001	-0.0003	0.0005	
dif allophilia	Samegen	Alone	LIFG~RTPJ	-0.0004	-0.0009	0.0001	
dif allophilia	Samegen	Together	LIFG~RTPJ	0.0000	-0.0005	0.0004	
dif allophilia	Intergen	Alone	RIFG~RIFG	-0.0002	-0.0008	0.0004	
dif allophilia	Intergen	Together	RIFG~RIFG	0.0005	0.0000	0.0010	
dif allophilia	Samegen	Alone	RIFG~RIFG	-0.0003	-0.0010	0.0003	
dif allophilia	Samegen	Together	RIFG~RIFG	0.0005	0.0000	0.0011	
dif allophilia	Intergen	Alone	RTPJ~RTPJ	0.0009	-0.0001	0.0018	
dif allophilia	Intergen	Together	RTPJ~RTPJ	-0.0003	-0.0011	0.0006	
dif allophilia	Samegen	Alone	RTPJ~RTPJ	-0.0001	-0.0011	0.0009	
dif allophilia	Samegen	Together	RTPJ~RTPJ	0.0000	-0.0008	0.0008	
dif allophilia	Intergen	Alone	RIFG~LTPJ	-0.0004	-0.0008	0.0000	
dif allophilia	Intergen	Together	RIFG~LTPJ	-0.0001	-0.0005	0.0002	
dif allophilia	Samegen	Alone	RIFG~LTPJ	0.0000	-0.0004	0.0005	
dif allophilia	Samegen	Together	RIFG~LTPJ	-0.0001	-0.0004	0.0003	
dif allophilia	Intergen	Alone	RIFG~RTPJ	0.0000	-0.0005	0.0005	
dif allophilia	Intergen	Together	RIFG~RTPJ	0.0002	-0.0003	0.0006	
dif allophilia	Samegen	Alone	RIFG~RTPJ	-0.0001	-0.0007	0.0005	
dif allophilia	Samegen	Together	RIFG~RTPJ	-0.0001	-0.0006	0.0003	
dif allophilia	Intergen	Alone	LIFG~RIFG	0.0002	-0.0002	0.0006	

S5 Appendix – Table V. HbO. Contrasts pertaining to allophilia (dif) ~ INS relationship in unstandardised units. Contrasts are presented in the following order: Group, task, and interaction contrasts.

Measure	Group	Task	ROI Pairs	Estimate	Lower HPD	Upper HPD	No overlap
Group contrast							
dif allophilia	Int-Samegen		LIFG~RIFG	0.0002	-0.0002	0.0007	
dif allophilia	Int-Samegen		LIFG~LIFG	0.0000	-0.0005	0.0006	
dif allophilia	Int-Samegen		LTPJ~LTPJ	0.0000	-0.0006	0.0007	
dif allophilia	Int-Samegen		LIFG~LTPJ	-0.0001	-0.0005	0.0004	
dif allophilia	Int-Samegen		LIFG~RTPJ	0.0002	-0.0003	0.0007	
dif allophilia	Int-Samegen		RIFG~RIFG	0.0001	-0.0006	0.0007	
dif allophilia	Int-Samegen		RTPJ~RTPJ	0.0004	-0.0006	0.0013	
dif allophilia	Int-Samegen		RIFG~LTPJ	-0.0002	-0.0006	0.0002	
dif allophilia	Int-Samegen		RIFG~RTPJ	0.0002	-0.0003	0.0008	
Task contrast							
dif allophilia		Alone-Together	LIFG~RIFG	-0.0002	-0.0006	0.0002	
dif allophilia		Alone-Together	LIFG~LIFG	0.0003	-0.0002	0.0008	
dif allophilia		Alone-Together	LTPJ~LTPJ	-0.0001	-0.0007	0.0004	
dif allophilia		Alone-Together	LIFG~LTPJ	0.0000	-0.0004	0.0004	
dif allophilia		Alone-Together	LIFG~RTPJ	-0.0002	-0.0007	0.0002	
dif allophilia		Alone-Together	RIFG~RIFG	-0.0008	-0.0013	-0.0002	No overlap
dif allophilia		Alone-Together	RTPJ~RTPJ	0.0005	-0.0003	0.0014	
dif allophilia		Alone-Together	RIFG~LTPJ	-0.0001	-0.0004	0.0003	
dif allophilia		Alone-Together	RIFG~RTPJ	-0.0001	-0.0005	0.0004	
Interactions							
dif allophilia	Int-Samegen	Alone	LIFG~RIFG	0.0004	-0.0002	0.0011	
dif allophilia	Int-Samegen	Together	LIFG~RIFG	0.0000	-0.0005	0.0005	
dif allophilia	Int-Samegen	Alone	LIFG~LIFG	0.0000	-0.0008	0.0008	
dif allophilia	Int-Samegen	Together	LIFG~LIFG	0.0000	-0.0006	0.0007	
dif allophilia	Int-Samegen	Alone	LTPJ~LTPJ	0.0002	-0.0007	0.0011	
dif allophilia	Int-Samegen	Together	LTPJ~LTPJ	-0.0001	-0.0009	0.0007	
dif allophilia	Int-Samegen	Alone	LIFG~LTPJ	-0.0001	-0.0007	0.0005	
dif allophilia	Int-Samegen	Together	LIFG~LTPJ	0.0000	-0.0005	0.0005	
dif allophilia	Int-Samegen	Alone	LIFG~RTPJ	0.0004	-0.0004	0.0011	
dif allophilia	Int-Samegen	Together	LIFG~RTPJ	0.0001	-0.0005	0.0007	
dif allophilia	Int-Samegen	Alone	RIFG~RIFG	0.0002	-0.0007	0.0011	
dif allophilia	Int-Samegen	Together	RIFG~RIFG	0.0000	-0.0008	0.0007	
dif allophilia	Int-Samegen	Alone	RTPJ~RTPJ	0.0009	-0.0004	0.0023	
dif allophilia	Int-Samegen	Together	RTPJ~RTPJ	-0.0002	-0.0014	0.0010	
dif allophilia	Int-Samegen	Alone	RIFG~LTPJ	-0.0004	-0.0010	0.0002	
dif allophilia	Int-Samegen	Together	RIFG~LTPJ	-0.0001	-0.0006	0.0004	
dif allophilia	Int-Samegen	Alone	RIFG~RTPJ	0.0001	-0.0007	0.0009	
dif allophilia	Int-Samegen	Together	RIFG~RTPJ	0.0003	-0.0003	0.0010	
dif allophilia	Intergen	Alone-Together	LIFG~RIFG	0.0001	-0.0005	0.0006	
dif allophilia	Samegen	Alone-Together	LIFG~RIFG	-0.0004	-0.0009	0.0002	
dif allophilia	Intergen	Alone-Together	LIFG~LIFG	0.0003	-0.0004	0.0009	
dif allophilia	Samegen	Alone-Together	LIFG~LIFG	0.0003	-0.0005	0.0010	

Measure	Group	Task	ROI Pairs	Estimate	Lower HPD	Upper HPD	No overlap
sum allophilia	Intergen	Alone-Together	LTPJ~LTPJ	0.0001	-0.0007	0.0008	
sum allophilia	Samegen	Alone-Together	LTPJ~LTPJ	-0.0003	-0.0011	0.0005	
sum allophilia	Intergen	Alone-Together	LIFG~LTPJ	0.0000	-0.0005	0.0005	
sum allophilia	Samegen	Alone-Together	LIFG~LTPJ	0.0000	-0.0005	0.0006	
sum allophilia	Intergen	Alone-Together	LIFG~RTPJ	-0.0001	-0.0007	0.0005	
sum allophilia	Samegen	Alone-Together	LIFG~RTPJ	-0.0003	-0.0010	0.0003	
sum allophilia	Intergen	Alone-Together	RIFG~RIFG	-0.0007	-0.0015	0.0001	
sum allophilia	Samegen	Alone-Together	RIFG~RIFG	-0.0009	-0.0017	-0.0001	No overlap
sum allophilia	Intergen	Alone-Together	RTPJ~RTPJ	0.0011	-0.0002	0.0023	
sum allophilia	Samegen	Alone-Together	RTPJ~RTPJ	0.0000	-0.0013	0.0012	
sum allophilia	Intergen	Alone-Together	RIFG~LTPJ	-0.0002	-0.0007	0.0003	
sum allophilia	Samegen	Alone-Together	RIFG~LTPJ	0.0001	-0.0004	0.0007	
sum allophilia	Intergen	Alone-Together	RIFG~RTPJ	-0.0002	-0.0008	0.0004	
sum allophilia	Samegen	Alone-Together	RIFG~RTPJ	0.0000	-0.0007	0.0007	

S6 Appendix – Table A. HbR. Estimates of INS levels for all sessions combined for real and pseudo dyads per ROI pair and task in unstandardised units.

Dyad Type	Group	ROI Pair	Task	Estimate	Lower HPD	Upper HPD
Pseudo	Intergen	LIFG~RIFG	Alone	0.238	0.238	0.239
Real	Intergen	LIFG~RIFG	Alone	0.233	0.228	0.238
Pseudo	Intergen	LIFG~RIFG	Together	0.239	0.238	0.239
Real	Intergen	LIFG~RIFG	Together	0.244	0.241	0.248
Pseudo	Intergen	LTPJ~RTPJ	Alone	0.236	0.236	0.237
Real	Intergen	LTPJ~RTPJ	Alone	0.238	0.231	0.243
Pseudo	Intergen	LTPJ~RTPJ	Together	0.237	0.236	0.237
Real	Intergen	LTPJ~RTPJ	Together	0.238	0.233	0.242
Pseudo	Intergen	LIFG~LIFG	Alone	0.24	0.239	0.241
Real	Intergen	LIFG~LIFG	Alone	0.237	0.231	0.244
Pseudo	Intergen	LIFG~LIFG	Together	0.24	0.24	0.241
Real	Intergen	LIFG~LIFG	Together	0.241	0.236	0.246
Pseudo	Intergen	LTPJ~LTPJ	Alone	0.237	0.237	0.238
Real	Intergen	LTPJ~LTPJ	Alone	0.241	0.234	0.247
Pseudo	Intergen	LTPJ~LTPJ	Together	0.237	0.237	0.238
Real	Intergen	LTPJ~LTPJ	Together	0.244	0.239	0.249
Pseudo	Intergen	LIFG~LTPJ	Alone	0.238	0.237	0.238
Real	Intergen	LIFG~LTPJ	Alone	0.24	0.236	0.245
Pseudo	Intergen	LIFG~LTPJ	Together	0.239	0.238	0.239
Real	Intergen	LIFG~LTPJ	Together	0.244	0.24	0.248
Pseudo	Intergen	LIFG~RTPJ	Alone	0.237	0.236	0.237
Real	Intergen	LIFG~RTPJ	Alone	0.235	0.229	0.241
Pseudo	Intergen	LIFG~RTPJ	Together	0.238	0.237	0.238
Real	Intergen	LIFG~RTPJ	Together	0.24	0.236	0.245
Pseudo	Intergen	RIFG~RIFG	Alone	0.237	0.236	0.238
Real	Intergen	RIFG~RIFG	Alone	0.241	0.233	0.247
Pseudo	Intergen	RIFG~RIFG	Together	0.237	0.237	0.238
Real	Intergen	RIFG~RIFG	Together	0.238	0.233	0.243
Pseudo	Intergen	RTPJ~RTPJ	Alone	0.235	0.233	0.236
Real	Intergen	RTPJ~RTPJ	Alone	0.233	0.221	0.245
Pseudo	Intergen	RTPJ~RTPJ	Together	0.236	0.235	0.237
Real	Intergen	RTPJ~RTPJ	Together	0.235	0.225	0.244
Pseudo	Intergen	LTPJ~RIFG	Alone	0.237	0.236	0.237
Real	Intergen	LTPJ~RIFG	Alone	0.236	0.231	0.24
Pseudo	Intergen	LTPJ~RIFG	Together	0.237	0.237	0.237
Real	Intergen	LTPJ~RIFG	Together	0.241	0.237	0.244
Pseudo	Intergen	RIFG~RTPJ	Alone	0.235	0.235	0.236
Real	Intergen	RIFG~RTPJ	Alone	0.23	0.224	0.236
Pseudo	Intergen	RIFG~RTPJ	Together	0.236	0.236	0.237
Real	Intergen	RIFG~RTPJ	Together	0.239	0.234	0.244
Pseudo	Samegen	LIFG~RIFG	Alone	0.24	0.24	0.24
Real	Samegen	LIFG~RIFG	Alone	0.237	0.232	0.241
Pseudo	Samegen	LIFG~RIFG	Together	0.24	0.24	0.241

Dyad Type	Group	ROI Pair	Task	Estimate	Lower HPD	Upper HPD
Real	Samegen	LIFG~RIFG	Together	0.24	0.236	0.244
Pseudo	Samegen	LTPJ~RTPJ	Alone	0.242	0.241	0.242
Real	Samegen	LTPJ~RTPJ	Alone	0.244	0.239	0.25
Pseudo	Samegen	LTPJ~RTPJ	Together	0.241	0.241	0.242
Real	Samegen	LTPJ~RTPJ	Together	0.244	0.24	0.249
Pseudo	Samegen	LIFG~LIFG	Alone	0.241	0.24	0.241
Real	Samegen	LIFG~LIFG	Alone	0.245	0.238	0.252
Pseudo	Samegen	LIFG~LIFG	Together	0.241	0.241	0.242
Real	Samegen	LIFG~LIFG	Together	0.244	0.239	0.249
Pseudo	Samegen	LTPJ~LTPJ	Alone	0.241	0.24	0.242
Real	Samegen	LTPJ~LTPJ	Alone	0.243	0.237	0.25
Pseudo	Samegen	LTPJ~LTPJ	Together	0.242	0.242	0.243
Real	Samegen	LTPJ~LTPJ	Together	0.245	0.24	0.25
Pseudo	Samegen	LIFG~LTPJ	Alone	0.24	0.24	0.241
Real	Samegen	LIFG~LTPJ	Alone	0.241	0.236	0.246
Pseudo	Samegen	LIFG~LTPJ	Together	0.241	0.24	0.241
Real	Samegen	LIFG~LTPJ	Together	0.241	0.238	0.245
Pseudo	Samegen	LIFG~RTPJ	Alone	0.24	0.24	0.241
Real	Samegen	LIFG~RTPJ	Alone	0.243	0.238	0.249
Pseudo	Samegen	LIFG~RTPJ	Together	0.24	0.24	0.24
Real	Samegen	LIFG~RTPJ	Together	0.239	0.235	0.244
Pseudo	Samegen	RIFG~RIFG	Alone	0.239	0.238	0.239
Real	Samegen	RIFG~RIFG	Alone	0.237	0.23	0.244
Pseudo	Samegen	RIFG~RIFG	Together	0.24	0.239	0.24
Real	Samegen	RIFG~RIFG	Together	0.246	0.24	0.251
Pseudo	Samegen	RTPJ~RTPJ	Alone	0.242	0.241	0.243
Real	Samegen	RTPJ~RTPJ	Alone	0.248	0.238	0.259
Pseudo	Samegen	RTPJ~RTPJ	Together	0.24	0.24	0.241
Real	Samegen	RTPJ~RTPJ	Together	0.244	0.236	0.252
Pseudo	Samegen	LTPJ~RIFG	Alone	0.239	0.238	0.239
Real	Samegen	LTPJ~RIFG	Alone	0.24	0.235	0.244
Pseudo	Samegen	LTPJ~RIFG	Together	0.24	0.24	0.24
Real	Samegen	LTPJ~RIFG	Together	0.244	0.24	0.247
Pseudo	Samegen	RIFG~RTPJ	Alone	0.239	0.239	0.24
Real	Samegen	RIFG~RTPJ	Alone	0.239	0.233	0.245
Pseudo	Samegen	RIFG~RTPJ	Together	0.239	0.239	0.24
Real	Samegen	RIFG~RTPJ	Together	0.242	0.237	0.246

S6 Appendix – Table B. HbR. Contrasts between real and pseudo dyads' INS levels for all sessions combined per ROI pair and task in unstandardised units.

Contrast	Group	ROI Pair	Task	Estimate	Lower HPD	Upper HPD	No overlap
Real - Pseudo	Intergen	LIFG~RIFG	Alone	0.005	0	0.009	
Real - Pseudo	Intergen	LIFG~RIFG	Together	-0.006	-0.009	-0.002	No overlap
Real - Pseudo	Intergen	LTPJ~RTPJ	Alone	-0.001	-0.007	0.005	
Real - Pseudo	Intergen	LTPJ~RTPJ	Together	-0.001	-0.006	0.004	
Real - Pseudo	Intergen	LIFG~LIFG	Alone	0.003	-0.004	0.01	
Real - Pseudo	Intergen	LIFG~LIFG	Together	-0.001	-0.006	0.005	
Real - Pseudo	Intergen	LTPJ~LTPJ	Alone	-0.003	-0.01	0.003	
Real - Pseudo	Intergen	LTPJ~LTPJ	Together	-0.007	-0.012	-0.002	No overlap
Real - Pseudo	Intergen	LIFG~LTPJ	Alone	-0.003	-0.007	0.002	
Real - Pseudo	Intergen	LIFG~LTPJ	Together	-0.005	-0.009	-0.002	No overlap
Real - Pseudo	Intergen	LIFG~RTPJ	Alone	0.001	-0.004	0.007	
Real - Pseudo	Intergen	LIFG~RTPJ	Together	-0.002	-0.007	0.002	
Real - Pseudo	Intergen	RIFG~RIFG	Alone	-0.004	-0.011	0.003	
Real - Pseudo	Intergen	RIFG~RIFG	Together	-0.001	-0.006	0.004	
Real - Pseudo	Intergen	RTPJ~RTPJ	Alone	0.002	-0.011	0.014	
Real - Pseudo	Intergen	RTPJ~RTPJ	Together	0.001	-0.009	0.011	
Real - Pseudo	Intergen	LTPJ~RIFG	Alone	0.001	-0.004	0.006	
Real - Pseudo	Intergen	LTPJ~RIFG	Together	-0.004	-0.007	0	
Real - Pseudo	Intergen	RIFG~RTPJ	Alone	0.005	-0.001	0.011	
Real - Pseudo	Intergen	RIFG~RTPJ	Together	-0.003	-0.007	0.002	
Real - Pseudo	Samegen	LIFG~RIFG	Alone	0.003	-0.002	0.008	
Real - Pseudo	Samegen	LIFG~RIFG	Together	0	-0.003	0.004	
Real - Pseudo	Samegen	LTPJ~RTPJ	Alone	-0.003	-0.008	0.003	
Real - Pseudo	Samegen	LTPJ~RTPJ	Together	-0.003	-0.008	0.001	
Real - Pseudo	Samegen	LIFG~LIFG	Alone	-0.004	-0.011	0.003	
Real - Pseudo	Samegen	LIFG~LIFG	Together	-0.003	-0.008	0.003	
Real - Pseudo	Samegen	LTPJ~LTPJ	Alone	-0.002	-0.009	0.004	
Real - Pseudo	Samegen	LTPJ~LTPJ	Together	-0.003	-0.008	0.002	
Real - Pseudo	Samegen	LIFG~LTPJ	Alone	-0.001	-0.006	0.004	
Real - Pseudo	Samegen	LIFG~LTPJ	Together	0	-0.004	0.003	
Real - Pseudo	Samegen	LIFG~RTPJ	Alone	-0.003	-0.008	0.003	
Real - Pseudo	Samegen	LIFG~RTPJ	Together	0.001	-0.004	0.005	
Real - Pseudo	Samegen	RIFG~RIFG	Alone	0.002	-0.005	0.009	
Real - Pseudo	Samegen	RIFG~RIFG	Together	-0.006	-0.011	0	No overlap
Real - Pseudo	Samegen	RTPJ~RTPJ	Alone	-0.006	-0.017	0.004	
Real - Pseudo	Samegen	RTPJ~RTPJ	Together	-0.003	-0.012	0.005	
Real - Pseudo	Samegen	LTPJ~RIFG	Alone	-0.001	-0.006	0.004	
Real - Pseudo	Samegen	LTPJ~RIFG	Together	-0.004	-0.008	0	No overlap
Real - Pseudo	Samegen	RIFG~RTPJ	Alone	0	-0.005	0.006	
Real - Pseudo	Samegen	RIFG~RTPJ	Together	-0.002	-0.007	0.002	

S6 Appendix – Table C. HbR. Estimates of slope of change in INS across sessions for real and pseudo dyads per ROI pair and task in unstandardised units.

Dyad Type	Group	ROI Pair	Task	Slope Estimate	Lower HPD	Upper HPD	No overlap
Pseudo	Intergen	LIFG~RIFG	Alone	0.001	0	0.001	No overlap
Real	Intergen	LIFG~RIFG	Alone	-0.001	-0.003	0.002	
Pseudo	Intergen	LIFG~RIFG	Together	0.001	0	0.001	No overlap
Real	Intergen	LIFG~RIFG	Together	0	-0.003	0.002	
Pseudo	Intergen	LTPJ~RTPJ	Alone	0	0	0.001	No overlap
Real	Intergen	LTPJ~RTPJ	Alone	0.002	-0.002	0.005	
Pseudo	Intergen	LTPJ~RTPJ	Together	0	-0.001	0	No overlap
Real	Intergen	LTPJ~RTPJ	Together	-0.002	-0.005	0.001	
Pseudo	Intergen	LIFG~LIFG	Alone	0	0	0.001	
Real	Intergen	LIFG~LIFG	Alone	0	-0.004	0.004	
Pseudo	Intergen	LIFG~LIFG	Together	0	0	0.001	No overlap
Real	Intergen	LIFG~LIFG	Together	-0.004	-0.007	0	No overlap
Pseudo	Intergen	LTPJ~LTPJ	Alone	0.001	0	0.001	No overlap
Real	Intergen	LTPJ~LTPJ	Alone	-0.001	-0.005	0.003	
Pseudo	Intergen	LTPJ~LTPJ	Together	0	-0.001	0	
Real	Intergen	LTPJ~LTPJ	Together	0.001	-0.003	0.004	
Pseudo	Intergen	LIFG~LTPJ	Alone	0	0	0.001	
Real	Intergen	LIFG~LTPJ	Alone	0	-0.003	0.003	
Pseudo	Intergen	LIFG~LTPJ	Together	0	0	0	
Real	Intergen	LIFG~LTPJ	Together	0	-0.002	0.003	
Pseudo	Intergen	LIFG~RTPJ	Alone	0	0	0.001	
Real	Intergen	LIFG~RTPJ	Alone	-0.002	-0.006	0.001	
Pseudo	Intergen	LIFG~RTPJ	Together	0	-0.001	0	
Real	Intergen	LIFG~RTPJ	Together	0.002	-0.001	0.005	
Pseudo	Intergen	RIFG~RIFG	Alone	0.001	0	0.001	No overlap
Real	Intergen	RIFG~RIFG	Alone	0	-0.004	0.005	
Pseudo	Intergen	RIFG~RIFG	Together	0.001	0.001	0.001	No overlap
Real	Intergen	RIFG~RIFG	Together	0	-0.004	0.004	
Pseudo	Intergen	RTPJ~RTPJ	Alone	0.001	0	0.002	
Real	Intergen	RTPJ~RTPJ	Alone	0.01	0.003	0.018	No overlap
Pseudo	Intergen	RTPJ~RTPJ	Together	0	-0.001	0.001	
Real	Intergen	RTPJ~RTPJ	Together	-0.003	-0.011	0.004	
Pseudo	Intergen	LTPJ~RIFG	Alone	0.001	0.001	0.001	No overlap
Real	Intergen	LTPJ~RIFG	Alone	0.003	0	0.006	No overlap
Pseudo	Intergen	LTPJ~RIFG	Together	0	0	0.001	
Real	Intergen	LTPJ~RIFG	Together	0	-0.003	0.003	
Pseudo	Intergen	RIFG~RTPJ	Alone	0.001	0	0.001	No overlap
Real	Intergen	RIFG~RTPJ	Alone	0.004	0.001	0.008	No overlap
Pseudo	Intergen	RIFG~RTPJ	Together	0.001	0	0.001	No overlap
Real	Intergen	RIFG~RTPJ	Together	-0.001	-0.005	0.002	
Pseudo	Samegen	LIFG~RIFG	Alone	0	0	0	
Real	Samegen	LIFG~RIFG	Alone	-0.002	-0.005	0	
Pseudo	Samegen	LIFG~RIFG	Together	0	0	0.001	No overlap

Dyad Type	Group	ROI Pair	Task	Slope Estimate	Lower HPD	Upper HPD	No overlap
Real	Samegen	LIFG~RIFG	Together	0	-0.002	0.003	
Pseudo	Samegen	LTPJ~RTPJ	Alone	0	0	0.001	No overlap
Real	Samegen	LTPJ~RTPJ	Alone	0.001	-0.003	0.004	
Pseudo	Samegen	LTPJ~RTPJ	Together	0.001	0	0.001	No overlap
Real	Samegen	LTPJ~RTPJ	Together	0.003	0	0.006	
Pseudo	Samegen	LIFG~LIFG	Alone	0.001	0	0.001	No overlap
Real	Samegen	LIFG~LIFG	Alone	0.001	-0.003	0.005	
Pseudo	Samegen	LIFG~LIFG	Together	0	0	0.001	No overlap
Real	Samegen	LIFG~LIFG	Together	-0.002	-0.005	0.002	
Pseudo	Samegen	LTPJ~LTPJ	Alone	0.001	0	0.001	No overlap
Real	Samegen	LTPJ~LTPJ	Alone	0	-0.004	0.003	
Pseudo	Samegen	LTPJ~LTPJ	Together	0	0	0	
Real	Samegen	LTPJ~LTPJ	Together	0.001	-0.002	0.005	
Pseudo	Samegen	LIFG~LTPJ	Alone	0	0	0.001	No overlap
Real	Samegen	LIFG~LTPJ	Alone	0.001	-0.002	0.003	
Pseudo	Samegen	LIFG~LTPJ	Together	0	0	0	
Real	Samegen	LIFG~LTPJ	Together	0.001	-0.002	0.004	
Pseudo	Samegen	LIFG~RTPJ	Alone	0	0	0	
Real	Samegen	LIFG~RTPJ	Alone	0	-0.004	0.003	
Pseudo	Samegen	LIFG~RTPJ	Together	0.001	0	0.001	No overlap
Real	Samegen	LIFG~RTPJ	Together	0	-0.003	0.003	
Pseudo	Samegen	RIFG~RIFG	Alone	-0.001	-0.001	0	No overlap
Real	Samegen	RIFG~RIFG	Alone	0.003	-0.001	0.007	
Pseudo	Samegen	RIFG~RIFG	Together	0	0	0.001	No overlap
Real	Samegen	RIFG~RIFG	Together	-0.001	-0.005	0.002	
Pseudo	Samegen	RTPJ~RTPJ	Alone	0	0	0.001	
Real	Samegen	RTPJ~RTPJ	Alone	-0.003	-0.01	0.004	
Pseudo	Samegen	RTPJ~RTPJ	Together	0.002	0.001	0.002	No overlap
Real	Samegen	RTPJ~RTPJ	Together	0.004	-0.002	0.011	
Pseudo	Samegen	LTPJ~RIFG	Alone	0	0	0	
Real	Samegen	LTPJ~RIFG	Alone	0.001	-0.002	0.004	
Pseudo	Samegen	LTPJ~RIFG	Together	0	0	0.001	No overlap
Real	Samegen	LTPJ~RIFG	Together	0.003	0	0.005	No overlap
Pseudo	Samegen	RIFG~RTPJ	Alone	0	-0.001	0	No overlap
Real	Samegen	RIFG~RTPJ	Alone	0	-0.004	0.003	
Pseudo	Samegen	RIFG~RTPJ	Together	0.001	0.001	0.001	No overlap
Real	Samegen	RIFG~RTPJ	Together	0	-0.003	0.003	

S6 Appendix – Table D. HbR. Contrasts between real and pseudo dyads' slopes of change in INS across sessions per ROI pair and task in unstandardised units.

Contrast	Group	ROI Pair	Task	Estimate	Lower HPD	Upper HPD	No overlap
Real - Pseudo	Intergen	LIFG~RIFG	Alone	0.001	-0.002	0.004	
Real - Pseudo	Intergen	LIFG~RIFG	Together	0.001	-0.002	0.003	
Real - Pseudo	Intergen	LTPJ~RTPJ	Alone	-0.001	-0.005	0.002	
Real - Pseudo	Intergen	LTPJ~RTPJ	Together	0.001	-0.002	0.005	
Real - Pseudo	Intergen	LIFG~LIFG	Alone	0	-0.003	0.004	
Real - Pseudo	Intergen	LIFG~LIFG	Together	0.004	0.001	0.008	No overlap
Real - Pseudo	Intergen	LTPJ~LTPJ	Alone	0.002	-0.002	0.006	
Real - Pseudo	Intergen	LTPJ~LTPJ	Together	-0.001	-0.005	0.003	
Real - Pseudo	Intergen	LIFG~LTPJ	Alone	0	-0.002	0.003	
Real - Pseudo	Intergen	LIFG~LTPJ	Together	0	-0.003	0.002	
Real - Pseudo	Intergen	LIFG~RTPJ	Alone	0.003	-0.001	0.006	
Real - Pseudo	Intergen	LIFG~RTPJ	Together	-0.002	-0.005	0.001	
Real - Pseudo	Intergen	RIFG~RIFG	Alone	0	-0.004	0.005	
Real - Pseudo	Intergen	RIFG~RIFG	Together	0.001	-0.003	0.005	
Real - Pseudo	Intergen	RTPJ~RTPJ	Alone	-0.01	-0.017	-0.002	No overlap
Real - Pseudo	Intergen	RTPJ~RTPJ	Together	0.003	-0.004	0.01	
Real - Pseudo	Intergen	LTPJ~RIFG	Alone	-0.002	-0.005	0.001	
Real - Pseudo	Intergen	LTPJ~RIFG	Together	0	-0.002	0.003	
Real - Pseudo	Intergen	RIFG~RTPJ	Alone	-0.004	-0.007	0	No overlap
Real - Pseudo	Intergen	RIFG~RTPJ	Together	0.002	-0.002	0.005	
Real - Pseudo	Samegen	LIFG~RIFG	Alone	0.002	0	0.005	
Real - Pseudo	Samegen	LIFG~RIFG	Together	0	-0.003	0.003	
Real - Pseudo	Samegen	LTPJ~RTPJ	Alone	0	-0.004	0.003	
Real - Pseudo	Samegen	LTPJ~RTPJ	Together	-0.002	-0.005	0.001	
Real - Pseudo	Samegen	LIFG~LIFG	Alone	0	-0.004	0.004	
Real - Pseudo	Samegen	LIFG~LIFG	Together	0.002	-0.002	0.006	
Real - Pseudo	Samegen	LTPJ~LTPJ	Alone	0.001	-0.003	0.005	
Real - Pseudo	Samegen	LTPJ~LTPJ	Together	-0.001	-0.005	0.003	
Real - Pseudo	Samegen	LIFG~LTPJ	Alone	0	-0.003	0.003	
Real - Pseudo	Samegen	LIFG~LTPJ	Together	-0.001	-0.004	0.002	
Real - Pseudo	Samegen	LIFG~RTPJ	Alone	0.001	-0.003	0.004	
Real - Pseudo	Samegen	LIFG~RTPJ	Together	0.001	-0.002	0.004	
Real - Pseudo	Samegen	RIFG~RIFG	Alone	-0.003	-0.007	0	
Real - Pseudo	Samegen	RIFG~RIFG	Together	0.002	-0.002	0.005	
Real - Pseudo	Samegen	RTPJ~RTPJ	Alone	0.003	-0.004	0.01	
Real - Pseudo	Samegen	RTPJ~RTPJ	Together	-0.003	-0.009	0.003	
Real - Pseudo	Samegen	LTPJ~RIFG	Alone	-0.001	-0.003	0.002	
Real - Pseudo	Samegen	LTPJ~RIFG	Together	-0.002	-0.005	0	
Real - Pseudo	Samegen	RIFG~RTPJ	Alone	0	-0.004	0.003	
Real - Pseudo	Samegen	RIFG~RTPJ	Together	0.001	-0.002	0.004	

S6 Appendix – Table E. HbR. Composition of non-homologous ROI pairs, to assess whether an ROI belonging to an older or younger member of a dyad influenced INS levels for all sessions (top) and across sessions (bottom). In contrast column, ‘o’ indicated that ROI belongs to older adult and ‘y’ indicates that ROI belongs to younger adult.

Contrast	Task	Estimate	Lower HPD	Upper HPD	No overlap
All sessions					
o_l_ifg_y_l_tpj - o_l_tpj_y_l_ifg	Together	0.006	-0.001	0.014	trend
o_l_ifg_y_r_ifg - o_r_ifg_y_l_ifg	Together	0	-0.007	0.007	
o_l_tpj_y_r_ifg - o_r_ifg_y_l_tpj	Together	-0.008	-0.015	0	No overlap
Across sessions					
o_r_ifg_y_r_tpj - o_r_tpj_y_r_ifg	Alone	-0.004	-0.017	0.008	

S6 Appendix – Table F. HbR. INS levels for all sessions: contrasts between tasks, groups and the task*group interaction per ROI pair in unstandardised units.

Contrast	ROI Pair	Estimate	Lower HPD	Upper HPD	No overlap
Tog-Alo	LIFG~RIFG	0.014	0.006	0.023	No overlap
Int-Sam	LIFG~RIFG	0	-0.01	0.01	
Tog-Alo Int	LIFG~RIFG	0.011	0.005	0.017	No overlap
Tog-Alo Sam	LIFG~RIFG	0.003	-0.003	0.009	
Int-Sam Alo	LIFG~RIFG	-0.004	-0.011	0.003	
Int-Sam Tog	LIFG~RIFG	0.004	-0.002	0.01	
Tog-Alo	LTPJ~LTPJ	0.006	-0.006	0.017	
Int-Sam	LTPJ~LTPJ	-0.004	-0.017	0.009	
Tog-Alo Int	LTPJ~LTPJ	0.004	-0.004	0.013	
Tog-Alo Sam	LTPJ~LTPJ	0.002	-0.006	0.01	
Int-Sam Alo	LTPJ~LTPJ	-0.003	-0.012	0.007	
Int-Sam Tog	LTPJ~LTPJ	-0.001	-0.009	0.007	
Tog-Alo	LIFG~LTPJ	0.004	-0.005	0.012	
Int-Sam	LIFG~LTPJ	0.002	-0.008	0.013	
Tog-Alo Int	LIFG~LTPJ	0.004	-0.002	0.01	
Tog-Alo Sam	LIFG~LTPJ	0	-0.006	0.006	
Int-Sam Alo	LIFG~LTPJ	-0.001	-0.008	0.007	
Int-Sam Tog	LIFG~LTPJ	0.003	-0.003	0.009	
Tog-Alo	RIFG~RIFG	0.006	-0.006	0.019	
Int-Sam	RIFG~RIFG	-0.004	-0.017	0.01	
Tog-Alo Int	RIFG~RIFG	-0.002	-0.011	0.007	
Tog-Alo Sam	RIFG~RIFG	0.009	0	0.018	
Int-Sam Alo	RIFG~RIFG	0.004	-0.007	0.014	
Int-Sam Tog	RIFG~RIFG	-0.007	-0.016	0.001	
Tog-Alo	RIFG~LTPJ	0.009	0	0.018	No overlap
Int-Sam	RIFG~LTPJ	-0.007	-0.017	0.002	
Tog-Alo Int	RIFG~LTPJ	0.005	-0.001	0.011	
Tog-Alo Sam	RIFG~LTPJ	0.004	-0.002	0.01	
Int-Sam Alo	RIFG~LTPJ	-0.004	-0.011	0.003	
Int-Sam Tog	RIFG~LTPJ	-0.003	-0.009	0.002	

S6 Appendix – Table G. HbR. Change in INS across sessions: contrasts between tasks, groups and the task*group interaction per ROI pair in unstandardised units.

Contrast	ROI Pair	Estimate	Lower HPD	Upper HPD	No overlap
Tog-Alo	LIFG~LIFG	-0.007	-0.014	0.001	
Int-Sam	LIFG~LIFG	-0.004	-0.011	0.004	
Tog-Alo Int	LIFG~LIFG	-0.004	-0.009	0.002	
Tog-Alo Sam	LIFG~LIFG	-0.003	-0.008	0.002	
Int-Sam Alo	LIFG~LIFG	-0.001	-0.007	0.004	
Int-Sam Tog	LIFG~LIFG	-0.002	-0.007	0.003	
Tog-Alo	RTPJ~RTPJ	-0.006	-0.02	0.007	
Int-Sam	RTPJ~RTPJ	0.005	-0.008	0.019	
Tog-Alo Int	RTPJ~RTPJ	-0.014	-0.023	-0.004	No overlap
Tog-Alo Sam	RTPJ~RTPJ	0.007	-0.002	0.016	
Int-Sam Alo	RTPJ~RTPJ	0.013	0.004	0.023	No overlap
Int-Sam Tog	RTPJ~RTPJ	-0.008	-0.017	0.002	
Tog-Alo	RIFG~RTPJ	-0.006	-0.012	0.001	
Int-Sam	RIFG~RTPJ	0.003	-0.004	0.01	
Tog-Alo Int	RIFG~RTPJ	-0.006	-0.01	-0.001	No overlap
Tog-Alo Sam	RIFG~RTPJ	0	-0.005	0.005	
Int-Sam Alo	RIFG~RTPJ	0.004	-0.001	0.009	
Int-Sam Tog	RIFG~RTPJ	-0.001	-0.006	0.003	

S6 Appendix – Table H. HbR. Relationships between session and INS in unstandardised units. Effects are presented in the following order: Main, group, task, and simple effects.

Measure	Group	Task	ROI Pairs	Estimate	Lower HPD	Upper HPD	No overlap
Main effect							
session			LIFG~RIFG	-0.0009	-0.0023	0.0006	
session			LIFG~LIFG	-0.0009	-0.0030	0.0012	
session			LTPJ~LTPJ	-0.0004	-0.0026	0.0016	
session			LIFG~LTPJ	0.0002	-0.0013	0.0017	
session			RIFG~RIFG	0.0010	-0.0012	0.0032	
session			RTPJ~RTPJ	0.0003	-0.0039	0.0043	
session			RIFG~LTPJ	0.0012	-0.0003	0.0027	
session			RIFG~RTPJ	0.0010	-0.0009	0.0029	
Group effect							
session	Intergen		LIFG~RIFG	-0.0005	-0.0026	0.0015	
session	Samegen		LIFG~RIFG	-0.0012	-0.0033	0.0010	
session	Intergen		LIFG~LIFG	-0.0024	-0.0053	0.0005	
session	Samegen		LIFG~LIFG	0.0006	-0.0025	0.0036	
session	Intergen		LTPJ~LTPJ	0.0002	-0.0026	0.0031	
session	Samegen		LTPJ~LTPJ	-0.0011	-0.0042	0.0019	
session	Intergen		LIFG~LTPJ	-0.0004	-0.0025	0.0017	
session	Samegen		LIFG~LTPJ	0.0008	-0.0014	0.0030	
session	Intergen		RIFG~RIFG	0.0002	-0.0030	0.0033	
session	Samegen		RIFG~RIFG	0.0018	-0.0014	0.0050	
session	Intergen		RTPJ~RTPJ	0.0017	-0.0041	0.0074	
session	Samegen		RTPJ~RTPJ	-0.0011	-0.0069	0.0047	
session	Intergen		RIFG~LTPJ	0.0014	-0.0007	0.0035	
session	Samegen		RIFG~LTPJ	0.0010	-0.0012	0.0031	
session	Intergen		RIFG~RTPJ	0.0011	-0.0015	0.0038	
session	Samegen		RIFG~RTPJ	0.0008	-0.0019	0.0035	
Task effect							
session		Alone	LIFG~RIFG	-0.0013	-0.0035	0.0009	
session		Together	LIFG~RIFG	-0.0004	-0.0023	0.0016	
session		Alone	LIFG~LIFG	0.0010	-0.0022	0.0041	
session		Together	LIFG~LIFG	-0.0028	-0.0057	-0.0001	No overlap
session		Alone	LTPJ~LTPJ	-0.0010	-0.0041	0.0021	
session		Together	LTPJ~LTPJ	0.0001	-0.0027	0.0028	
session		Alone	LIFG~LTPJ	0.0001	-0.0021	0.0023	
session		Together	LIFG~LTPJ	0.0003	-0.0017	0.0024	
session		Alone	RIFG~RIFG	0.0026	-0.0007	0.0059	
session		Together	RIFG~RIFG	-0.0006	-0.0036	0.0023	
session		Alone	RTPJ~RTPJ	0.0005	-0.0055	0.0064	
session		Together	RTPJ~RTPJ	0.0001	-0.0053	0.0058	
session		Alone	RIFG~LTPJ	0.0018	-0.0004	0.0041	
session		Together	RIFG~LTPJ	0.0006	-0.0014	0.0027	
session		Alone	RIFG~RTPJ	0.0018	-0.0011	0.0045	
session		Together	RIFG~RTPJ	0.0002	-0.0023	0.0028	

Measure	Group	Task	ROI Pairs	Estimate	Lower HPD	Upper HPD	No overlap
Simple effect							
session	Intergen	Alone	LIFG~RIFG	-0.0002	-0.0033	0.0028	
session	Intergen	Together	LIFG~RIFG	-0.0008	-0.0036	0.0019	
session	Samegen	Alone	LIFG~RIFG	-0.0024	-0.0054	0.0008	
session	Samegen	Together	LIFG~RIFG	0.0000	-0.0029	0.0028	
session	Intergen	Alone	LIFG~LIFG	-0.0007	-0.0049	0.0036	
session	Intergen	Together	LIFG~LIFG	-0.0041	-0.0079	-0.0001	No overlap
session	Samegen	Alone	LIFG~LIFG	0.0027	-0.0019	0.0072	
session	Samegen	Together	LIFG~LIFG	-0.0015	-0.0057	0.0025	
session	Intergen	Alone	LTPJ~LTPJ	0.0005	-0.0037	0.0048	
session	Intergen	Together	LTPJ~LTPJ	0.0000	-0.0038	0.0038	
session	Samegen	Alone	LTPJ~LTPJ	-0.0024	-0.0069	0.0021	
session	Samegen	Together	LTPJ~LTPJ	0.0002	-0.0037	0.0044	
session	Intergen	Alone	LIFG~LTPJ	-0.0009	-0.0040	0.0021	
session	Intergen	Together	LIFG~LTPJ	0.0002	-0.0026	0.0030	
session	Samegen	Alone	LIFG~LTPJ	0.0011	-0.0021	0.0044	
session	Samegen	Together	LIFG~LTPJ	0.0004	-0.0025	0.0033	
session	Intergen	Alone	RIFG~RIFG	0.0007	-0.0039	0.0053	
session	Intergen	Together	RIFG~RIFG	-0.0003	-0.0043	0.0039	
session	Samegen	Alone	RIFG~RIFG	0.0045	-0.0001	0.0093	
session	Samegen	Together	RIFG~RIFG	-0.0009	-0.0051	0.0033	
session	Intergen	Alone	RTPJ~RTPJ	0.0086	0.0005	0.0166	No overlap
session	Intergen	Together	RTPJ~RTPJ	-0.0052	-0.0134	0.0029	
session	Samegen	Alone	RTPJ~RTPJ	-0.0076	-0.0160	0.0012	
session	Samegen	Together	RTPJ~RTPJ	0.0054	-0.0024	0.0127	
session	Intergen	Alone	RIFG~LTPJ	0.0033	0.0002	0.0064	No overlap
session	Intergen	Together	RIFG~LTPJ	-0.0005	-0.0033	0.0023	
session	Samegen	Alone	RIFG~LTPJ	0.0002	-0.0031	0.0034	
session	Samegen	Together	RIFG~LTPJ	0.0017	-0.0012	0.0046	
session	Intergen	Alone	RIFG~RTPJ	0.0034	-0.0006	0.0072	
session	Intergen	Together	RIFG~RTPJ	-0.0011	-0.0047	0.0025	
session	Samegen	Alone	RIFG~RTPJ	0.0001	-0.0040	0.0041	
session	Samegen	Together	RIFG~RTPJ	0.0015	-0.0021	0.0051	

S6 Appendix – Table I. HbR. Contrasts pertaining to session ~ INS relationship in unstandardised units. Contrasts are presented in the following order: Group, task, and interaction contrasts.

Measure	Group	Task	ROI Pairs	Estimate	Lower HPD	Upper HPD	No overlap
Group contrast							
session	Intergen-Samegen		LIFG~RIFG	0.0007	-0.0023	0.0036	
session	Intergen-Samegen		LIFG~LIFG	-0.0030	-0.0072	0.0013	
session	Intergen-Samegen		LTPJ~LTPJ	0.0013	-0.0029	0.0054	
session	Intergen-Samegen		LIFG~LTPJ	-0.0011	-0.0041	0.0020	
session	Intergen-Samegen		RIFG~RIFG	-0.0016	-0.0062	0.0028	
session	Intergen-Samegen		RTPJ~RTPJ	0.0028	-0.0054	0.0111	
session	Intergen-Samegen		RIFG~LTPJ	0.0004	-0.0026	0.0035	
session	Intergen-Samegen		RIFG~RTPJ	0.0003	-0.0034	0.0042	
Task contrast							
session		Alone-Together	LIFG~RIFG	-0.0009	-0.0039	0.0020	
session		Alone-Together	LIFG~LIFG	0.0038	-0.0004	0.0079	
session		Alone-Together	LTPJ~LTPJ	-0.0011	-0.0051	0.0032	
session		Alone-Together	LIFG~LTPJ	-0.0002	-0.0032	0.0028	
session		Alone-Together	RIFG~RIFG	0.0033	-0.0012	0.0076	
session		Alone-Together	RTPJ~RTPJ	0.0004	-0.0078	0.0083	
session		Alone-Together	RIFG~LTPJ	0.0011	-0.0019	0.0040	
session		Alone-Together	RIFG~RTPJ	0.0016	-0.0021	0.0054	
Interactions							
session	Intergen-Samegen	Alone	LIFG~RIFG	0.0021	-0.0023	0.0064	
session	Intergen-Samegen	Together	LIFG~RIFG	-0.0008	-0.0047	0.0032	
session	Intergen-Samegen	Alone	LIFG~LIFG	-0.0034	-0.0095	0.0030	
session	Intergen-Samegen	Together	LIFG~LIFG	-0.0026	-0.0081	0.0031	
session	Intergen-Samegen	Alone	LTPJ~LTPJ	0.0029	-0.0036	0.0089	
session	Intergen-Samegen	Together	LTPJ~LTPJ	-0.0003	-0.0057	0.0055	
session	Intergen-Samegen	Alone	LIFG~LTPJ	-0.0020	-0.0065	0.0024	
session	Intergen-Samegen	Together	LIFG~LTPJ	-0.0002	-0.0041	0.0040	
session	Intergen-Samegen	Alone	RIFG~RIFG	-0.0038	-0.0102	0.0030	
session	Intergen-Samegen	Together	RIFG~RIFG	0.0006	-0.0052	0.0066	
session	Intergen-Samegen	Alone	RTPJ~RTPJ	0.0161	0.0044	0.0279	No overlap
session	Intergen-Samegen	Together	RTPJ~RTPJ	-0.0104	-0.0217	0.0006	
session	Intergen-Samegen	Alone	RIFG~LTPJ	0.0031	-0.0013	0.0076	
session	Intergen-Samegen	Together	RIFG~LTPJ	-0.0022	-0.0062	0.0018	
session	Intergen-Samegen	Alone	RIFG~RTPJ	0.0032	-0.0024	0.0090	
session	Intergen-Samegen	Together	RIFG~RTPJ	-0.0026	-0.0078	0.0023	
session	Intergen	Alone-Together	LIFG~RIFG	0.0006	-0.0034	0.0048	
session	Samegen	Alone-Together	LIFG~RIFG	-0.0023	-0.0065	0.0018	
session	Intergen	Alone-Together	LIFG~LIFG	0.0034	-0.0024	0.0091	
session	Samegen	Alone-Together	LIFG~LIFG	0.0042	-0.0019	0.0101	
session	Intergen	Alone-Together	LTPJ~LTPJ	0.0005	-0.0053	0.0062	
session	Samegen	Alone-Together	LTPJ~LTPJ	-0.0026	-0.0085	0.0036	
session	Intergen	Alone-Together	LIFG~LTPJ	-0.0011	-0.0052	0.0030	
session	Samegen	Alone-Together	LIFG~LTPJ	0.0007	-0.0037	0.0049	

Measure	Group	Task	ROI Pairs	Estimate	Lower HPD	Upper HPD	No overlap
session	Intergen	Alone-Together	RIFG~RIFG	0.0010	-0.0051	0.0072	
session	Samegen	Alone-Together	RIFG~RIFG	0.0054	-0.0008	0.0116	
session	Intergen	Alone-Together	RTPJ~RTPJ	0.0137	0.0022	0.0248	No overlap
session	Samegen	Alone-Together	RTPJ~RTPJ	-0.0129	-0.0247	-0.0018	No overlap
session	Intergen	Alone-Together	RIFG~LTPJ	0.0039	-0.0003	0.0081	
session	Samegen	Alone-Together	RIFG~LTPJ	-0.0015	-0.0059	0.0028	
session	Intergen	Alone-Together	RIFG~RTPJ	0.0045	-0.0008	0.0097	
session	Samegen	Alone-Together	RIFG~RTPJ	-0.0014	-0.0066	0.0041	

S6 Appendix – Table J. HbR. Relationships between loneliness (sum) and INS in unstandardised units. Effects are presented in the following order: Main, group, task, and simple effects.

Measure	Group	Task	ROI Pairs	Estimate	Lower HPD	Upper HPD	No overlap
Main effect							
sum loneliness			LIFG~RIFG	-0.0017	-0.0029	-0.0004	No overlap
sum loneliness			LIFG~LIFG	-0.0007	-0.0023	0.0009	
sum loneliness			LTPJ~LTPJ	-0.0004	-0.0020	0.0011	
sum loneliness			LIFG~LTPJ	-0.0010	-0.0023	0.0002	
sum loneliness			RIFG~RIFG	-0.0010	-0.0027	0.0008	
sum loneliness			RTPJ~RTPJ	0.0003	-0.0025	0.0032	
sum loneliness			RIFG~LTPJ	-0.0004	-0.0016	0.0007	
sum loneliness			RIFG~RTPJ	-0.0008	-0.0022	0.0007	
Group effect							
sum loneliness	Intergen		LIFG~RIFG	-0.0014	-0.0032	0.0003	
sum loneliness	Samegen		LIFG~RIFG	-0.0020	-0.0038	-0.0003	No overlap
sum loneliness	Intergen		LIFG~LIFG	-0.0011	-0.0033	0.0011	
sum loneliness	Samegen		LIFG~LIFG	-0.0003	-0.0027	0.0020	
sum loneliness	Intergen		LTPJ~LTPJ	-0.0010	-0.0032	0.0012	
sum loneliness	Samegen		LTPJ~LTPJ	0.0002	-0.0021	0.0026	
sum loneliness	Intergen		LIFG~LTPJ	-0.0014	-0.0031	0.0004	
sum loneliness	Samegen		LIFG~LTPJ	-0.0007	-0.0026	0.0012	
sum loneliness	Intergen		RIFG~RIFG	-0.0009	-0.0033	0.0015	
sum loneliness	Samegen		RIFG~RIFG	-0.0011	-0.0035	0.0014	
sum loneliness	Intergen		RTPJ~RTPJ	-0.0003	-0.0040	0.0034	
sum loneliness	Samegen		RTPJ~RTPJ	0.0009	-0.0035	0.0051	
sum loneliness	Intergen		RIFG~LTPJ	0.0007	-0.0009	0.0023	
sum loneliness	Samegen		RIFG~LTPJ	-0.0015	-0.0031	0.0002	
sum loneliness	Intergen		RIFG~RTPJ	0.0002	-0.0018	0.0023	
sum loneliness	Samegen		RIFG~RTPJ	-0.0017	-0.0038	0.0004	
Task effect							
sum loneliness		Alone	LIFG~RIFG	-0.0020	-0.0038	-0.0001	No overlap
sum loneliness		Together	LIFG~RIFG	-0.0014	-0.0028	0.0000	
sum loneliness		Alone	LIFG~LIFG	-0.0011	-0.0036	0.0014	
sum loneliness		Together	LIFG~LIFG	-0.0003	-0.0022	0.0017	
sum loneliness		Alone	LTPJ~LTPJ	0.0002	-0.0023	0.0027	
sum loneliness		Together	LTPJ~LTPJ	-0.0011	-0.0031	0.0008	
sum loneliness		Alone	LIFG~LTPJ	-0.0010	-0.0029	0.0008	
sum loneliness		Together	LIFG~LTPJ	-0.0010	-0.0025	0.0004	
sum loneliness		Alone	RIFG~RIFG	-0.0008	-0.0035	0.0018	
sum loneliness		Together	RIFG~RIFG	-0.0011	-0.0032	0.0010	
sum loneliness		Alone	RTPJ~RTPJ	0.0010	-0.0032	0.0053	
sum loneliness		Together	RTPJ~RTPJ	-0.0003	-0.0040	0.0032	
sum loneliness		Alone	RIFG~LTPJ	0.0001	-0.0017	0.0019	
sum loneliness		Together	RIFG~LTPJ	-0.0009	-0.0023	0.0005	
sum loneliness		Alone	RIFG~RTPJ	-0.0007	-0.0029	0.0015	
sum loneliness		Together	RIFG~RTPJ	-0.0008	-0.0026	0.0009	

Measure	Group	Task	ROI Pairs	Estimate	Lower HPD	Upper HPD	No overlap
Main effect							
sum loneliness	Intergen	Alone	LIFG~RIFG	-0.0008	-0.0034	0.0017	
sum loneliness	Intergen	Together	LIFG~RIFG	-0.0020	-0.0040	0.0000	
sum loneliness	Samegen	Alone	LIFG~RIFG	-0.0031	-0.0057	-0.0006	No overlap
sum loneliness	Samegen	Together	LIFG~RIFG	-0.0008	-0.0029	0.0013	
sum loneliness	Intergen	Alone	LIFG~LIFG	-0.0002	-0.0037	0.0031	
sum loneliness	Intergen	Together	LIFG~LIFG	-0.0020	-0.0047	0.0006	
sum loneliness	Samegen	Alone	LIFG~LIFG	-0.0021	-0.0056	0.0016	
sum loneliness	Samegen	Together	LIFG~LIFG	0.0014	-0.0014	0.0042	
sum loneliness	Intergen	Alone	LTPJ~LTPJ	-0.0011	-0.0044	0.0024	
sum loneliness	Intergen	Together	LTPJ~LTPJ	-0.0010	-0.0036	0.0017	
sum loneliness	Samegen	Alone	LTPJ~LTPJ	0.0015	-0.0022	0.0050	
sum loneliness	Samegen	Together	LTPJ~LTPJ	-0.0012	-0.0041	0.0016	
sum loneliness	Intergen	Alone	LIFG~LTPJ	-0.0007	-0.0033	0.0019	
sum loneliness	Intergen	Together	LIFG~LTPJ	-0.0020	-0.0041	0.0000	
sum loneliness	Samegen	Alone	LIFG~LTPJ	-0.0014	-0.0041	0.0013	
sum loneliness	Samegen	Together	LIFG~LTPJ	0.0000	-0.0021	0.0022	
sum loneliness	Intergen	Alone	RIFG~RIFG	-0.0008	-0.0044	0.0030	
sum loneliness	Intergen	Together	RIFG~RIFG	-0.0010	-0.0040	0.0018	
sum loneliness	Samegen	Alone	RIFG~RIFG	-0.0009	-0.0047	0.0028	
sum loneliness	Samegen	Together	RIFG~RIFG	-0.0012	-0.0041	0.0017	
sum loneliness	Intergen	Alone	RTPJ~RTPJ	-0.0008	-0.0064	0.0047	
sum loneliness	Intergen	Together	RTPJ~RTPJ	0.0003	-0.0046	0.0049	
sum loneliness	Samegen	Alone	RTPJ~RTPJ	0.0027	-0.0038	0.0090	
sum loneliness	Samegen	Together	RTPJ~RTPJ	-0.0010	-0.0065	0.0044	
sum loneliness	Intergen	Alone	RIFG~LTPJ	0.0014	-0.0011	0.0039	
sum loneliness	Intergen	Together	RIFG~LTPJ	0.0000	-0.0020	0.0019	
sum loneliness	Samegen	Alone	RIFG~LTPJ	-0.0011	-0.0037	0.0015	
sum loneliness	Samegen	Together	RIFG~LTPJ	-0.0018	-0.0039	0.0001	
sum loneliness	Intergen	Alone	RIFG~RTPJ	-0.0003	-0.0033	0.0029	
sum loneliness	Intergen	Together	RIFG~RTPJ	0.0007	-0.0018	0.0031	
sum loneliness	Samegen	Alone	RIFG~RTPJ	-0.0011	-0.0043	0.0021	
sum loneliness	Samegen	Together	RIFG~RTPJ	-0.0023	-0.0048	0.0002	

S6 Appendix – Table K. HbR. Contrasts pertaining to loneliness (sum) ~ INS relationship in unstandardised units. Contrasts are presented in the following order: Group, task, and interaction contrasts.

Measure	Group	Task	ROI Pairs	Estimate	Lower HPD	Upper HPD	No overlap
Group contrast							
sum loneliness	Intergen-Samegen		LIFG~RIFG	0.0006	-0.0018	0.0031	
sum loneliness	Intergen-Samegen		LIFG~LIFG	-0.0008	-0.0039	0.0025	
sum loneliness	Intergen-Samegen		LTPJ~LTPJ	-0.0012	-0.0044	0.0020	
sum loneliness	Intergen-Samegen		LIFG~LTPJ	-0.0007	-0.0033	0.0019	
sum loneliness	Intergen-Samegen		RIFG~RIFG	0.0001	-0.0033	0.0037	
sum loneliness	Intergen-Samegen		RTPJ~RTPJ	-0.0011	-0.0068	0.0046	
sum loneliness	Intergen-Samegen		RIFG~LTPJ	0.0022	-0.0001	0.0045	
sum loneliness	Intergen-Samegen		RIFG~RTPJ	0.0019	-0.0010	0.0049	
Task contrast							
sum loneliness		Alone-Together	LIFG~RIFG	-0.0006	-0.0027	0.0016	
sum loneliness		Alone-Together	LIFG~LIFG	-0.0008	-0.0039	0.0023	
sum loneliness		Alone-Together	LTPJ~LTPJ	0.0013	-0.0018	0.0044	
sum loneliness		Alone-Together	LIFG~LTPJ	0.0000	-0.0023	0.0021	
sum loneliness		Alone-Together	RIFG~RIFG	0.0003	-0.0029	0.0036	
sum loneliness		Alone-Together	RTPJ~RTPJ	0.0013	-0.0041	0.0068	
sum loneliness		Alone-Together	RIFG~LTPJ	0.0010	-0.0012	0.0033	
sum loneliness		Alone-Together	RIFG~RTPJ	0.0001	-0.0026	0.0029	
Interactions							
sum loneliness	Intergen-Samegen	Alone	LIFG~RIFG	0.0023	-0.0012	0.0060	
sum loneliness	Intergen-Samegen	Together	LIFG~RIFG	-0.0012	-0.0039	0.0018	
sum loneliness	Intergen-Samegen	Alone	LIFG~LIFG	0.0019	-0.0031	0.0068	
sum loneliness	Intergen-Samegen	Together	LIFG~LIFG	-0.0034	-0.0073	0.0004	
sum loneliness	Intergen-Samegen	Alone	LTPJ~LTPJ	-0.0026	-0.0075	0.0023	
sum loneliness	Intergen-Samegen	Together	LTPJ~LTPJ	0.0002	-0.0037	0.0041	
sum loneliness	Intergen-Samegen	Alone	LIFG~LTPJ	0.0007	-0.0031	0.0044	
sum loneliness	Intergen-Samegen	Together	LIFG~LTPJ	-0.0020	-0.0051	0.0010	
sum loneliness	Intergen-Samegen	Alone	RIFG~RIFG	0.0001	-0.0050	0.0054	
sum loneliness	Intergen-Samegen	Together	RIFG~RIFG	0.0002	-0.0038	0.0044	
sum loneliness	Intergen-Samegen	Alone	RTPJ~RTPJ	-0.0035	-0.0121	0.0049	
sum loneliness	Intergen-Samegen	Together	RTPJ~RTPJ	0.0012	-0.0059	0.0084	
sum loneliness	Intergen-Samegen	Alone	RIFG~LTPJ	0.0025	-0.0010	0.0062	
sum loneliness	Intergen-Samegen	Together	RIFG~LTPJ	0.0018	-0.0010	0.0046	
sum loneliness	Intergen-Samegen	Alone	RIFG~RTPJ	0.0008	-0.0035	0.0053	
sum loneliness	Intergen-Samegen	Together	RIFG~RTPJ	0.0029	-0.0007	0.0063	
sum loneliness	Intergen	Alone-Together	LIFG~RIFG	0.0012	-0.0018	0.0042	
sum loneliness	Samegen	Alone-Together	LIFG~RIFG	-0.0023	-0.0054	0.0007	
sum loneliness	Intergen	Alone-Together	LIFG~LIFG	0.0018	-0.0024	0.0062	
sum loneliness	Samegen	Alone-Together	LIFG~LIFG	-0.0035	-0.0079	0.0011	
sum loneliness	Intergen	Alone-Together	LTPJ~LTPJ	-0.0001	-0.0044	0.0042	
sum loneliness	Samegen	Alone-Together	LTPJ~LTPJ	0.0027	-0.0017	0.0073	
sum loneliness	Intergen	Alone-Together	LIFG~LTPJ	0.0013	-0.0018	0.0043	
sum loneliness	Samegen	Alone-Together	LIFG~LTPJ	-0.0014	-0.0045	0.0018	

Measure	Group	Task	ROI Pairs	Estimate	Lower HPD	Upper HPD	No overlap
sum loneliness	Intergen	Alone-Together	RIFG~RIFG	0.0002	-0.0042	0.0048	
sum loneliness	Samegen	Alone-Together	RIFG~RIFG	0.0004	-0.0042	0.0049	
sum loneliness	Intergen	Alone-Together	RTPJ~RTPJ	-0.0010	-0.0081	0.0060	
sum loneliness	Samegen	Alone-Together	RTPJ~RTPJ	0.0037	-0.0045	0.0120	
sum loneliness	Intergen	Alone-Together	RIFG~LTPJ	0.0014	-0.0018	0.0045	
sum loneliness	Samegen	Alone-Together	RIFG~LTPJ	0.0007	-0.0025	0.0039	
sum loneliness	Intergen	Alone-Together	RIFG~RTPJ	-0.0010	-0.0047	0.0029	
sum loneliness	Samegen	Alone-Together	RIFG~RTPJ	0.0011	-0.0027	0.0050	

S6 Appendix – Table L. HbR. Relationships between loneliness (dif) and INS in unstandardised units. Effects are presented in the following order: Main, group, task, and simple effects.

Measure	Group	Task	ROI Pairs	Estimate	Lower HPD	Upper HPD	No overlap
Main effect							
dif loneliness			LIFG~RIFG	0.0010	-0.0013	0.0032	
dif loneliness			LIFG~LIFG	0.0002	-0.0026	0.0031	
dif loneliness			LTPJ~LTPJ	-0.0035	-0.0064	-0.0007	No overlap
dif loneliness			LIFG~LTPJ	0.0010	-0.0013	0.0032	
dif loneliness			RIFG~RIFG	0.0010	-0.0022	0.0043	
dif loneliness			RTPJ~RTPJ	-0.0018	-0.0072	0.0035	
dif loneliness			RIFG~LTPJ	-0.0002	-0.0024	0.0019	
dif loneliness			RIFG~RTPJ	0.0011	-0.0015	0.0039	
Group effect							
dif loneliness	Intergen		LIFG~RIFG	0.0007	-0.0029	0.0041	
dif loneliness	Samegen		LIFG~RIFG	0.0013	-0.0012	0.0040	
dif loneliness	Intergen		LIFG~LIFG	0.0013	-0.0033	0.0059	
dif loneliness	Samegen		LIFG~LIFG	-0.0009	-0.0043	0.0026	
dif loneliness	Intergen		LTPJ~LTPJ	-0.0051	-0.0098	-0.0005	No overlap
dif loneliness	Samegen		LTPJ~LTPJ	-0.0019	-0.0055	0.0014	
dif loneliness	Intergen		LIFG~LTPJ	0.0009	-0.0026	0.0044	
dif loneliness	Samegen		LIFG~LTPJ	0.0010	-0.0017	0.0039	
dif loneliness	Intergen		RIFG~RIFG	0.0004	-0.0047	0.0059	
dif loneliness	Samegen		RIFG~RIFG	0.0016	-0.0019	0.0054	
dif loneliness	Intergen		RTPJ~RTPJ	-0.0027	-0.0117	0.0061	
dif loneliness	Samegen		RTPJ~RTPJ	-0.0009	-0.0067	0.0051	
dif loneliness	Intergen		RIFG~LTPJ	-0.0015	-0.0050	0.0020	
dif loneliness	Samegen		RIFG~LTPJ	0.0011	-0.0013	0.0037	
dif loneliness	Intergen		RIFG~RTPJ	-0.0005	-0.0049	0.0039	
dif loneliness	Samegen		RIFG~RTPJ	0.0027	-0.0004	0.0058	
Task effect							
dif loneliness		Alone	LIFG~RIFG	0.0016	-0.0016	0.0049	
dif loneliness		Together	LIFG~RIFG	0.0004	-0.0022	0.0031	
dif loneliness		Alone	LIFG~LIFG	0.0007	-0.0039	0.0049	
dif loneliness		Together	LIFG~LIFG	-0.0002	-0.0037	0.0034	
dif loneliness		Alone	LTPJ~LTPJ	-0.0029	-0.0074	0.0015	
dif loneliness		Together	LTPJ~LTPJ	-0.0042	-0.0077	-0.0006	No overlap
dif loneliness		Alone	LIFG~LTPJ	0.0019	-0.0014	0.0052	
dif loneliness		Together	LIFG~LTPJ	0.0000	-0.0028	0.0026	
dif loneliness		Alone	RIFG~RIFG	0.0037	-0.0011	0.0087	
dif loneliness		Together	RIFG~RIFG	-0.0017	-0.0056	0.0022	
dif loneliness		Alone	RTPJ~RTPJ	-0.0041	-0.0124	0.0038	
dif loneliness		Together	RTPJ~RTPJ	0.0006	-0.0062	0.0072	
dif loneliness		Alone	RIFG~LTPJ	0.0007	-0.0025	0.0040	
dif loneliness		Together	RIFG~LTPJ	-0.0011	-0.0038	0.0014	
dif loneliness		Alone	RIFG~RTPJ	0.0037	-0.0004	0.0079	
dif loneliness		Together	RIFG~RTPJ	-0.0015	-0.0048	0.0018	

Measure	Group	Task	ROI Pairs	Estimate	Lower HPD	Upper HPD	No overlap
Main effect							
dif loneliness	Intergen	Alone	LIFG~RIFG	0.0006	-0.0045	0.0060	
dif loneliness	Intergen	Together	LIFG~RIFG	0.0007	-0.0036	0.0049	
dif loneliness	Samegen	Alone	LIFG~RIFG	0.0025	-0.0013	0.0063	
dif loneliness	Samegen	Together	LIFG~RIFG	0.0001	-0.0029	0.0032	
dif loneliness	Intergen	Alone	LIFG~LIFG	0.0012	-0.0058	0.0082	
dif loneliness	Intergen	Together	LIFG~LIFG	0.0014	-0.0044	0.0072	
dif loneliness	Samegen	Alone	LIFG~LIFG	0.0001	-0.0053	0.0055	
dif loneliness	Samegen	Together	LIFG~LIFG	-0.0018	-0.0059	0.0024	
dif loneliness	Intergen	Alone	LTPJ~LTPJ	-0.0048	-0.0117	0.0025	
dif loneliness	Intergen	Together	LTPJ~LTPJ	-0.0054	-0.0113	0.0002	
dif loneliness	Samegen	Alone	LTPJ~LTPJ	-0.0009	-0.0062	0.0045	
dif loneliness	Samegen	Together	LTPJ~LTPJ	-0.0029	-0.0070	0.0015	
dif loneliness	Intergen	Alone	LIFG~LTPJ	0.0009	-0.0044	0.0060	
dif loneliness	Intergen	Together	LIFG~LTPJ	0.0009	-0.0035	0.0051	
dif loneliness	Samegen	Alone	LIFG~LTPJ	0.0030	-0.0011	0.0069	
dif loneliness	Samegen	Together	LIFG~LTPJ	-0.0009	-0.0041	0.0023	
dif loneliness	Intergen	Alone	RIFG~RIFG	0.0049	-0.0031	0.0129	
dif loneliness	Intergen	Together	RIFG~RIFG	-0.0041	-0.0106	0.0023	
dif loneliness	Samegen	Alone	RIFG~RIFG	0.0024	-0.0031	0.0079	
dif loneliness	Samegen	Together	RIFG~RIFG	0.0008	-0.0036	0.0051	
dif loneliness	Intergen	Alone	RTPJ~RTPJ	-0.0050	-0.0179	0.0087	
dif loneliness	Intergen	Together	RTPJ~RTPJ	-0.0005	-0.0121	0.0108	
dif loneliness	Samegen	Alone	RTPJ~RTPJ	-0.0033	-0.0123	0.0061	
dif loneliness	Samegen	Together	RTPJ~RTPJ	0.0016	-0.0054	0.0086	
dif loneliness	Intergen	Alone	RIFG~LTPJ	-0.0009	-0.0063	0.0045	
dif loneliness	Intergen	Together	RIFG~LTPJ	-0.0022	-0.0065	0.0020	
dif loneliness	Samegen	Alone	RIFG~LTPJ	0.0023	-0.0015	0.0061	
dif loneliness	Samegen	Together	RIFG~LTPJ	-0.0001	-0.0031	0.0029	
dif loneliness	Intergen	Alone	RIFG~RTPJ	0.0030	-0.0039	0.0097	
dif loneliness	Intergen	Together	RIFG~RTPJ	-0.0039	-0.0094	0.0016	
dif loneliness	Samegen	Alone	RIFG~RTPJ	0.0044	-0.0003	0.0091	
dif loneliness	Samegen	Together	RIFG~RTPJ	0.0009	-0.0028	0.0046	

Supplementary Materials: Moffat, Dumas and Cross (2026). *Social interactions between people of same and different generations shape longitudinal changes in interpersonal neural synchrony, loneliness, and social connection.*

S6 Appendix – Table M. HbR. Contrasts pertaining to loneliness (dif) ~ INS relationship in unstandardised units. Contrasts are presented in the following order: Group, task, and interaction contrasts.

Measure	Group	Task	ROI Pairs	Estimate	Lower HPD	Upper HPD	No overlap
Group contrast							
dif loneliness	Intergen-Samegen		LIFG~RIFG	-0.0007	-0.0050	0.0037	
dif loneliness	Intergen-Samegen		LIFG~LIFG	0.0021	-0.0035	0.0080	
dif loneliness	Intergen-Samegen		LTPJ~LTPJ	-0.0031	-0.0088	0.0027	
dif loneliness	Intergen-Samegen		LIFG~LTPJ	-0.0002	-0.0047	0.0042	
dif loneliness	Intergen-Samegen		RIFG~RIFG	-0.0012	-0.0076	0.0052	
dif loneliness	Intergen-Samegen		RTPJ~RTPJ	-0.0018	-0.0126	0.0088	
dif loneliness	Intergen-Samegen		RIFG~LTPJ	-0.0026	-0.0069	0.0016	
dif loneliness	Intergen-Samegen		RIFG~RTPJ	-0.0031	-0.0084	0.0024	
Task contrast							
dif loneliness		Alone-Together	LIFG~RIFG	0.0012	-0.0028	0.0050	
dif loneliness		Alone-Together	LIFG~LIFG	0.0009	-0.0048	0.0064	
dif loneliness		Alone-Together	LTPJ~LTPJ	0.0013	-0.0042	0.0071	
dif loneliness		Alone-Together	LIFG~LTPJ	0.0019	-0.0020	0.0060	
dif loneliness		Alone-Together	RIFG~RIFG	0.0053	-0.0008	0.0115	
dif loneliness		Alone-Together	RTPJ~RTPJ	-0.0047	-0.0152	0.0055	
dif loneliness		Alone-Together	RIFG~LTPJ	0.0019	-0.0023	0.0060	
dif loneliness		Alone-Together	RIFG~RTPJ	0.0052	0.0001	0.0104	No overlap
Interactions							
dif loneliness	Intergen-Samegen	Alone	LIFG~RIFG	-0.0019	-0.0087	0.0042	
dif loneliness	Intergen-Samegen	Together	LIFG~RIFG	0.0006	-0.0047	0.0057	
dif loneliness	Intergen-Samegen	Alone	LIFG~LIFG	0.0011	-0.0076	0.0100	
dif loneliness	Intergen-Samegen	Together	LIFG~LIFG	0.0032	-0.0039	0.0102	
dif loneliness	Intergen-Samegen	Alone	LTPJ~LTPJ	-0.0038	-0.0124	0.0052	
dif loneliness	Intergen-Samegen	Together	LTPJ~LTPJ	-0.0024	-0.0096	0.0046	
dif loneliness	Intergen-Samegen	Alone	LIFG~LTPJ	-0.0021	-0.0084	0.0047	
dif loneliness	Intergen-Samegen	Together	LIFG~LTPJ	0.0018	-0.0038	0.0070	
dif loneliness	Intergen-Samegen	Alone	RIFG~RIFG	0.0025	-0.0074	0.0121	
dif loneliness	Intergen-Samegen	Together	RIFG~RIFG	-0.0048	-0.0127	0.0029	
dif loneliness	Intergen-Samegen	Alone	RTPJ~RTPJ	-0.0016	-0.0180	0.0144	
dif loneliness	Intergen-Samegen	Together	RTPJ~RTPJ	-0.0020	-0.0155	0.0112	
dif loneliness	Intergen-Samegen	Alone	RIFG~LTPJ	-0.0032	-0.0099	0.0033	
dif loneliness	Intergen-Samegen	Together	RIFG~LTPJ	-0.0020	-0.0073	0.0031	
dif loneliness	Intergen-Samegen	Alone	RIFG~RTPJ	-0.0014	-0.0096	0.0068	
dif loneliness	Intergen-Samegen	Together	RIFG~RTPJ	-0.0049	-0.0115	0.0018	
dif loneliness	Intergen	Alone-Together	LIFG~RIFG	-0.0001	-0.0066	0.0063	
dif loneliness	Samegen	Alone-Together	LIFG~RIFG	0.0024	-0.0022	0.0068	
dif loneliness	Intergen	Alone-Together	LIFG~LIFG	-0.0001	-0.0093	0.0087	
dif loneliness	Samegen	Alone-Together	LIFG~LIFG	0.0019	-0.0047	0.0085	
dif loneliness	Intergen	Alone-Together	LTPJ~LTPJ	0.0006	-0.0087	0.0093	
dif loneliness	Samegen	Alone-Together	LTPJ~LTPJ	0.0020	-0.0046	0.0088	
dif loneliness	Intergen	Alone-Together	LIFG~LTPJ	0.0000	-0.0064	0.0066	
dif loneliness	Samegen	Alone-Together	LIFG~LTPJ	0.0038	-0.0008	0.0086	

Measure	Group	Task	ROI Pairs	Estimate	Lower HPD	Upper HPD	No overlap
dif loneliness	Intergen	Alone-Together	RIFG~RIFG	0.0090	-0.0011	0.0191	
dif loneliness	Samegen	Alone-Together	RIFG~RIFG	0.0017	-0.0050	0.0086	
dif loneliness	Intergen	Alone-Together	RTPJ~RTPJ	-0.0044	-0.0219	0.0128	
dif loneliness	Samegen	Alone-Together	RTPJ~RTPJ	-0.0049	-0.0162	0.0061	
dif loneliness	Intergen	Alone-Together	RIFG~LTPJ	0.0013	-0.0056	0.0080	
dif loneliness	Samegen	Alone-Together	RIFG~LTPJ	0.0024	-0.0023	0.0072	
dif loneliness	Intergen	Alone-Together	RIFG~RTPJ	0.0069	-0.0014	0.0156	
dif loneliness	Samegen	Alone-Together	RIFG~RTPJ	0.0034	-0.0022	0.0093	

S6 Appendix – Table N. HbR. Relationships between social closeness (sum) and INS in unstandardised units. Effects are presented in the following order: Main, group, task, and simple effects.

Measure	Group	Task	ROI Pairs	Estimate	Lower HPD	Upper HPD	No overlap
Main effect							
sum social closeness			LIFG~RIFG	0.0000	-0.0013	0.0013	
sum social closeness			LIFG~LIFG	-0.0007	-0.0025	0.0011	
sum social closeness			LTPJ~LTPJ	0.0008	-0.0009	0.0026	
sum social closeness			LIFG~LTPJ	0.0002	-0.0012	0.0016	
sum social closeness			RIFG~RIFG	-0.0009	-0.0028	0.0011	
sum social closeness			RTPJ~RTPJ	0.0025	-0.0007	0.0059	
sum social closeness			RIFG~LTPJ	0.0007	-0.0006	0.0020	
sum social closeness			RIFG~RTPJ	-0.0004	-0.0020	0.0012	
Group effect							
sum social closeness	Intergen		LIFG~RIFG	0.0000	-0.0014	0.0016	
sum social closeness	Samegen		LIFG~RIFG	0.0000	-0.0022	0.0022	
sum social closeness	Intergen		LIFG~LIFG	0.0002	-0.0018	0.0021	
sum social closeness	Samegen		LIFG~LIFG	-0.0016	-0.0046	0.0014	
sum social closeness	Intergen		LTPJ~LTPJ	-0.0007	-0.0026	0.0013	
sum social closeness	Samegen		LTPJ~LTPJ	0.0023	-0.0006	0.0054	
sum social closeness	Intergen		LIFG~LTPJ	0.0003	-0.0012	0.0018	
sum social closeness	Samegen		LIFG~LTPJ	0.0002	-0.0020	0.0025	
sum social closeness	Intergen		RIFG~RIFG	0.0004	-0.0017	0.0026	
sum social closeness	Samegen		RIFG~RIFG	-0.0022	-0.0054	0.0011	
sum social closeness	Intergen		RTPJ~RTPJ	0.0024	-0.0015	0.0062	
sum social closeness	Samegen		RTPJ~RTPJ	0.0027	-0.0027	0.0079	
sum social closeness	Intergen		RIFG~LTPJ	0.0004	-0.0011	0.0017	
sum social closeness	Samegen		RIFG~LTPJ	0.0010	-0.0011	0.0032	
sum social closeness	Intergen		RIFG~RTPJ	0.0013	-0.0005	0.0032	
sum social closeness	Samegen		RIFG~RTPJ	-0.0020	-0.0047	0.0006	
Task effect							
sum social closeness		Alone	LIFG~RIFG	-0.0006	-0.0026	0.0014	
sum social closeness		Together	LIFG~RIFG	0.0006	-0.0010	0.0022	
sum social closeness		Alone	LIFG~LIFG	-0.0016	-0.0043	0.0013	
sum social closeness		Together	LIFG~LIFG	0.0002	-0.0020	0.0023	
sum social closeness		Alone	LTPJ~LTPJ	0.0007	-0.0021	0.0035	
sum social closeness		Together	LTPJ~LTPJ	0.0010	-0.0011	0.0032	
sum social closeness		Alone	LIFG~LTPJ	0.0001	-0.0020	0.0020	
sum social closeness		Together	LIFG~LTPJ	0.0004	-0.0012	0.0022	
sum social closeness		Alone	RIFG~RIFG	-0.0017	-0.0046	0.0014	
sum social closeness		Together	RIFG~RIFG	-0.0002	-0.0025	0.0022	
sum social closeness		Alone	RTPJ~RTPJ	0.0037	-0.0015	0.0084	
sum social closeness		Together	RTPJ~RTPJ	0.0014	-0.0028	0.0055	
sum social closeness		Alone	RIFG~LTPJ	0.0004	-0.0015	0.0025	
sum social closeness		Together	RIFG~LTPJ	0.0009	-0.0006	0.0026	
sum social closeness		Alone	RIFG~RTPJ	0.0010	-0.0014	0.0035	
sum social closeness		Together	RIFG~RTPJ	-0.0017	-0.0037	0.0002	

Measure	Group	Task	ROI Pairs	Estimate	Lower HPD	Upper HPD	No overlap
Main effect							
sum social closeness	Intergen	Alone	LIFG~RIFG	-0.0006	-0.0028	0.0017	
sum social closeness	Intergen	Together	LIFG~RIFG	0.0007	-0.0012	0.0025	
sum social closeness	Samegen	Alone	LIFG~RIFG	-0.0006	-0.0040	0.0026	
sum social closeness	Samegen	Together	LIFG~RIFG	0.0005	-0.0022	0.0031	
sum social closeness	Intergen	Alone	LIFG~LIFG	0.0006	-0.0025	0.0036	
sum social closeness	Intergen	Together	LIFG~LIFG	-0.0001	-0.0025	0.0023	
sum social closeness	Samegen	Alone	LIFG~LIFG	-0.0037	-0.0082	0.0012	
sum social closeness	Samegen	Together	LIFG~LIFG	0.0004	-0.0031	0.0041	
sum social closeness	Intergen	Alone	LTPJ~LTPJ	-0.0024	-0.0055	0.0006	
sum social closeness	Intergen	Together	LTPJ~LTPJ	0.0011	-0.0013	0.0035	
sum social closeness	Samegen	Alone	LTPJ~LTPJ	0.0037	-0.0009	0.0084	
sum social closeness	Samegen	Together	LTPJ~LTPJ	0.0010	-0.0027	0.0046	
sum social closeness	Intergen	Alone	LIFG~LTPJ	0.0010	-0.0013	0.0033	
sum social closeness	Intergen	Together	LIFG~LTPJ	-0.0004	-0.0022	0.0014	
sum social closeness	Samegen	Alone	LIFG~LTPJ	-0.0008	-0.0042	0.0026	
sum social closeness	Samegen	Together	LIFG~LTPJ	0.0013	-0.0015	0.0040	
sum social closeness	Intergen	Alone	RIFG~RIFG	-0.0003	-0.0038	0.0029	
sum social closeness	Intergen	Together	RIFG~RIFG	0.0011	-0.0015	0.0037	
sum social closeness	Samegen	Alone	RIFG~RIFG	-0.0030	-0.0079	0.0021	
sum social closeness	Samegen	Together	RIFG~RIFG	-0.0014	-0.0053	0.0025	
sum social closeness	Intergen	Alone	RTPJ~RTPJ	0.0016	-0.0039	0.0071	
sum social closeness	Intergen	Together	RTPJ~RTPJ	0.0032	-0.0019	0.0084	
sum social closeness	Samegen	Alone	RTPJ~RTPJ	0.0058	-0.0026	0.0139	
sum social closeness	Samegen	Together	RTPJ~RTPJ	-0.0004	-0.0070	0.0061	
sum social closeness	Intergen	Alone	RIFG~LTPJ	-0.0002	-0.0024	0.0020	
sum social closeness	Intergen	Together	RIFG~LTPJ	0.0009	-0.0009	0.0026	
sum social closeness	Samegen	Alone	RIFG~LTPJ	0.0011	-0.0023	0.0044	
sum social closeness	Samegen	Together	RIFG~LTPJ	0.0009	-0.0018	0.0036	
sum social closeness	Intergen	Alone	RIFG~RTPJ	0.0021	-0.0007	0.0049	
sum social closeness	Intergen	Together	RIFG~RTPJ	0.0005	-0.0018	0.0028	
sum social closeness	Samegen	Alone	RIFG~RTPJ	-0.0001	-0.0043	0.0039	
sum social closeness	Samegen	Together	RIFG~RTPJ	-0.0039	-0.0072	-0.0008	No overlap

S6 Appendix – Table O. HbR. Contrasts pertaining to social closeness (sum) ~ INS relationship in unstandardised units. Contrasts are presented in the following order: Group, task, and interaction contrasts.

Measure	Group	Task	ROI Pairs	Estimate	Lower HPD	Upper HPD	No overlap
Group contrast							
sum social closeness	Int-Samegen		LIFG~RIFG	0.0001	-0.0025	0.0028	
sum social closeness	Int-Samegen		LIFG~LIFG	0.0018	-0.0017	0.0054	
sum social closeness	Int-Samegen		LTPJ~LTPJ	-0.0030	-0.0067	0.0004	
sum social closeness	Int-Samegen		LIFG~LTPJ	0.0001	-0.0027	0.0029	
sum social closeness	Int-Samegen		RIFG~RIFG	0.0025	-0.0015	0.0064	
sum social closeness	Int-Samegen		RTPJ~RTPJ	-0.0003	-0.0069	0.0061	
sum social closeness	Int-Samegen		RIFG~LTPJ	-0.0006	-0.0032	0.0020	
sum social closeness	Int-Samegen		RIFG~RTPJ	0.0033	0.0001	0.0065	No overlap
Task contrast							
sum social closeness		Alone-Together	LIFG~RIFG	-0.0012	-0.0036	0.0013	
sum social closeness		Alone-Together	LIFG~LIFG	-0.0017	-0.0052	0.0018	
sum social closeness		Alone-Together	LTPJ~LTPJ	-0.0004	-0.0039	0.0030	
sum social closeness		Alone-Together	LIFG~LTPJ	-0.0004	-0.0029	0.0020	
sum social closeness		Alone-Together	RIFG~RIFG	-0.0015	-0.0052	0.0022	
sum social closeness		Alone-Together	RTPJ~RTPJ	0.0023	-0.0040	0.0088	
sum social closeness		Alone-Together	RIFG~LTPJ	-0.0005	-0.0030	0.0020	
sum social closeness		Alone-Together	RIFG~RTPJ	0.0027	-0.0004	0.0058	
Interactions							
sum social closeness	Int-Samegen	Alone	LIFG~RIFG	0.0000	-0.0039	0.0041	
sum social closeness	Int-Samegen	Together	LIFG~RIFG	0.0002	-0.0031	0.0033	
sum social closeness	Int-Samegen	Alone	LIFG~LIFG	0.0042	-0.0013	0.0099	
sum social closeness	Int-Samegen	Together	LIFG~LIFG	-0.0005	-0.0048	0.0039	
sum social closeness	Int-Samegen	Alone	LTPJ~LTPJ	-0.0061	-0.0117	-0.0006	No overlap
sum social closeness	Int-Samegen	Together	LTPJ~LTPJ	0.0001	-0.0042	0.0045	
sum social closeness	Int-Samegen	Alone	LIFG~LTPJ	0.0018	-0.0022	0.0060	
sum social closeness	Int-Samegen	Together	LIFG~LTPJ	-0.0017	-0.0049	0.0016	
sum social closeness	Int-Samegen	Alone	RIFG~RIFG	0.0027	-0.0036	0.0083	
sum social closeness	Int-Samegen	Together	RIFG~RIFG	0.0024	-0.0023	0.0071	
sum social closeness	Int-Samegen	Alone	RTPJ~RTPJ	-0.0042	-0.0141	0.0057	
sum social closeness	Int-Samegen	Together	RTPJ~RTPJ	0.0036	-0.0049	0.0119	
sum social closeness	Int-Samegen	Alone	RIFG~LTPJ	-0.0013	-0.0052	0.0029	
sum social closeness	Int-Samegen	Together	RIFG~LTPJ	0.0000	-0.0032	0.0032	
sum social closeness	Int-Samegen	Alone	RIFG~RTPJ	0.0022	-0.0026	0.0074	
sum social closeness	Int-Samegen	Together	RIFG~RTPJ	0.0044	0.0004	0.0084	No overlap
sum social closeness	Intergen	Alone-Together	LIFG~RIFG	-0.0013	-0.0039	0.0015	
sum social closeness	Samegen	Alone-Together	LIFG~RIFG	-0.0011	-0.0050	0.0031	
sum social closeness	Intergen	Alone-Together	LIFG~LIFG	0.0007	-0.0032	0.0045	
sum social closeness	Samegen	Alone-Together	LIFG~LIFG	-0.0041	-0.0097	0.0019	
sum social closeness	Intergen	Alone-Together	LTPJ~LTPJ	-0.0035	-0.0074	0.0002	
sum social closeness	Samegen	Alone-Together	LTPJ~LTPJ	0.0028	-0.0030	0.0087	
sum social closeness	Intergen	Alone-Together	LIFG~LTPJ	0.0014	-0.0013	0.0041	
sum social closeness	Samegen	Alone-Together	LIFG~LTPJ	-0.0021	-0.0063	0.0020	

Measure	Group	Task	ROI Pairs	Estimate	Lower HPD	Upper HPD	No overlap
sum social closeness	Intergen	Alone-Together	RIFG~RIFG	-0.0014	-0.0054	0.0028	
sum social closeness	Samegen	Alone-Together	RIFG~RIFG	-0.0016	-0.0080	0.0045	
sum social closeness	Intergen	Alone-Together	RTPJ~RTPJ	-0.0016	-0.0091	0.0058	
sum social closeness	Samegen	Alone-Together	RTPJ~RTPJ	0.0062	-0.0041	0.0166	
sum social closeness	Intergen	Alone-Together	RIFG~LTPJ	-0.0011	-0.0039	0.0017	
sum social closeness	Samegen	Alone-Together	RIFG~LTPJ	0.0002	-0.0040	0.0044	
sum social closeness	Intergen	Alone-Together	RIFG~RTPJ	0.0016	-0.0019	0.0052	
sum social closeness	Samegen	Alone-Together	RIFG~RTPJ	0.0038	-0.0015	0.0087	

S6 Appendix – Table P. HbR. Relationships between social closeness (dif) and INS in unstandardised units. Effects are presented in the following order: Main, group, task, and simple effects.

Measure	Group	Task	ROI Pairs	Estimate	Lower HPD	Upper HPD	No overlap
Main effect							
dif social closeness			LIFG~RIFG	0.0019	-0.0002	0.0040	
dif social closeness			LIFG~LIFG	0.0016	-0.0011	0.0045	
dif social closeness			LTPJ~LTPJ	0.0009	-0.0019	0.0037	
dif social closeness			LIFG~LTPJ	0.0012	-0.0010	0.0033	
dif social closeness			RIFG~RIFG	0.0027	-0.0005	0.0058	
dif social closeness			RTPJ~RTPJ	-0.0026	-0.0075	0.0023	
dif social closeness			RIFG~LTPJ	0.0003	-0.0019	0.0022	
dif social closeness			RIFG~RTPJ	0.0009	-0.0017	0.0036	
Group effect							
dif social closeness	Intergen		LIFG~RIFG	0.0013	-0.0015	0.0040	
dif social closeness	Samegen		LIFG~RIFG	0.0024	-0.0008	0.0056	
dif social closeness	Intergen		LIFG~LIFG	0.0001	-0.0036	0.0037	
dif social closeness	Samegen		LIFG~LIFG	0.0032	-0.0009	0.0075	
dif social closeness	Intergen		LTPJ~LTPJ	0.0004	-0.0033	0.0040	
dif social closeness	Samegen		LTPJ~LTPJ	0.0014	-0.0029	0.0056	
dif social closeness	Intergen		LIFG~LTPJ	0.0017	-0.0011	0.0045	
dif social closeness	Samegen		LIFG~LTPJ	0.0006	-0.0025	0.0040	
dif social closeness	Intergen		RIFG~RIFG	0.0047	0.0006	0.0087	No overlap
dif social closeness	Samegen		RIFG~RIFG	0.0007	-0.0042	0.0054	
dif social closeness	Intergen		RTPJ~RTPJ	-0.0063	-0.0137	0.0009	
dif social closeness	Samegen		RTPJ~RTPJ	0.0010	-0.0054	0.0074	
dif social closeness	Intergen		RIFG~LTPJ	0.0023	-0.0003	0.0051	
dif social closeness	Samegen		RIFG~LTPJ	-0.0019	-0.0049	0.0014	
dif social closeness	Intergen		RIFG~RTPJ	-0.0005	-0.0040	0.0031	
dif social closeness	Samegen		RIFG~RTPJ	0.0023	-0.0016	0.0061	
Task effect							
dif social closeness		Alone	LIFG~RIFG	0.0042	0.0011	0.0074	No overlap
dif social closeness		Together	LIFG~RIFG	-0.0004	-0.0030	0.0020	
dif social closeness		Alone	LIFG~LIFG	0.0039	-0.0005	0.0082	
dif social closeness		Together	LIFG~LIFG	-0.0006	-0.0039	0.0028	
dif social closeness		Alone	LTPJ~LTPJ	0.0006	-0.0038	0.0049	
dif social closeness		Together	LTPJ~LTPJ	0.0012	-0.0022	0.0046	
dif social closeness		Alone	LIFG~LTPJ	0.0015	-0.0018	0.0046	
dif social closeness		Together	LIFG~LTPJ	0.0009	-0.0017	0.0034	
dif social closeness		Alone	RIFG~RIFG	0.0038	-0.0012	0.0084	
dif social closeness		Together	RIFG~RIFG	0.0016	-0.0022	0.0054	
dif social closeness		Alone	RTPJ~RTPJ	-0.0046	-0.0123	0.0025	
dif social closeness		Together	RTPJ~RTPJ	-0.0007	-0.0067	0.0053	
dif social closeness		Alone	RIFG~LTPJ	0.0007	-0.0025	0.0040	
dif social closeness		Together	RIFG~LTPJ	-0.0002	-0.0027	0.0023	
dif social closeness		Alone	RIFG~RTPJ	0.0014	-0.0027	0.0054	
dif social closeness		Together	RIFG~RTPJ	0.0004	-0.0027	0.0036	

Measure	Group	Task	ROI Pairs	Estimate	Lower HPD	Upper HPD	No overlap
Simple effect							
dif social closeness	Intergen	Alone	LIFG~RIFG	0.0032	-0.0011	0.0073	
dif social closeness	Intergen	Together	LIFG~RIFG	-0.0006	-0.0038	0.0027	
dif social closeness	Samegen	Alone	LIFG~RIFG	0.0052	0.0004	0.0099	No overlap
dif social closeness	Samegen	Together	LIFG~RIFG	-0.0002	-0.0041	0.0035	
dif social closeness	Intergen	Alone	LIFG~LIFG	0.0017	-0.0040	0.0075	
dif social closeness	Intergen	Together	LIFG~LIFG	-0.0015	-0.0057	0.0030	
dif social closeness	Samegen	Alone	LIFG~LIFG	0.0061	-0.0004	0.0126	
dif social closeness	Samegen	Together	LIFG~LIFG	0.0002	-0.0050	0.0054	
dif social closeness	Intergen	Alone	LTPJ~LTPJ	0.0028	-0.0029	0.0084	
dif social closeness	Intergen	Together	LTPJ~LTPJ	-0.0021	-0.0065	0.0023	
dif social closeness	Samegen	Alone	LTPJ~LTPJ	-0.0016	-0.0083	0.0049	
dif social closeness	Samegen	Together	LTPJ~LTPJ	0.0044	-0.0008	0.0096	
dif social closeness	Intergen	Alone	LIFG~LTPJ	0.0030	-0.0013	0.0072	
dif social closeness	Intergen	Together	LIFG~LTPJ	0.0005	-0.0028	0.0039	
dif social closeness	Samegen	Alone	LIFG~LTPJ	0.0000	-0.0047	0.0049	
dif social closeness	Samegen	Together	LIFG~LTPJ	0.0013	-0.0026	0.0051	
dif social closeness	Intergen	Alone	RIFG~RIFG	0.0047	-0.0016	0.0110	
dif social closeness	Intergen	Together	RIFG~RIFG	0.0047	0.0000	0.0096	
dif social closeness	Samegen	Alone	RIFG~RIFG	0.0029	-0.0046	0.0100	
dif social closeness	Samegen	Together	RIFG~RIFG	-0.0014	-0.0071	0.0048	
dif social closeness	Intergen	Alone	RTPJ~RTPJ	-0.0042	-0.0153	0.0066	
dif social closeness	Intergen	Together	RTPJ~RTPJ	-0.0084	-0.0176	0.0010	
dif social closeness	Samegen	Alone	RTPJ~RTPJ	-0.0050	-0.0148	0.0048	
dif social closeness	Samegen	Together	RTPJ~RTPJ	0.0071	-0.0006	0.0146	
dif social closeness	Intergen	Alone	RIFG~LTPJ	0.0018	-0.0024	0.0059	
dif social closeness	Intergen	Together	RIFG~LTPJ	0.0029	-0.0003	0.0062	
dif social closeness	Samegen	Alone	RIFG~LTPJ	-0.0004	-0.0053	0.0045	
dif social closeness	Samegen	Together	RIFG~LTPJ	-0.0033	-0.0073	0.0005	
dif social closeness	Intergen	Alone	RIFG~RTPJ	-0.0010	-0.0066	0.0044	
dif social closeness	Intergen	Together	RIFG~RTPJ	0.0001	-0.0044	0.0043	
dif social closeness	Samegen	Alone	RIFG~RTPJ	0.0038	-0.0021	0.0096	
dif social closeness	Samegen	Together	RIFG~RTPJ	0.0008	-0.0037	0.0055	

S6 Appendix – Table Q. HbR. Contrasts pertaining to social closeness (dif) ~ INS relationship in unstandardised units. Contrasts are presented in the following order: Group, task, and interaction contrasts.

Measure	Group	Task	ROI Pairs	Estimate	Lower HPD	Upper HPD	No overlap
Group contrast							
dif social closeness	Int-Samegen		LIFG~RIFG	-0.0012	-0.0053	0.0030	
dif social closeness	Int-Samegen		LIFG~LIFG	-0.0031	-0.0087	0.0024	
dif social closeness	Int-Samegen		LTPJ~LTPJ	-0.0011	-0.0067	0.0044	
dif social closeness	Int-Samegen		LIFG~LTPJ	0.0011	-0.0031	0.0054	
dif social closeness	Int-Samegen		RIFG~RIFG	0.0039	-0.0022	0.0104	
dif social closeness	Int-Samegen		RTPJ~RTPJ	-0.0073	-0.0169	0.0024	
dif social closeness	Int-Samegen		RIFG~LTPJ	0.0042	-0.0001	0.0083	
dif social closeness	Int-Samegen		RIFG~RTPJ	-0.0027	-0.0080	0.0024	
Task contrast							
dif social closeness		Alone-Together	LIFG~RIFG	0.0045	0.0006	0.0084	No overlap
dif social closeness		Alone-Together	LIFG~LIFG	0.0045	-0.0009	0.0101	
dif social closeness		Alone-Together	LTPJ~LTPJ	-0.0005	-0.0060	0.0050	
dif social closeness		Alone-Together	LIFG~LTPJ	0.0006	-0.0033	0.0045	
dif social closeness		Alone-Together	RIFG~RIFG	0.0022	-0.0038	0.0082	
dif social closeness		Alone-Together	RTPJ~RTPJ	-0.0040	-0.0132	0.0055	
dif social closeness		Alone-Together	RIFG~LTPJ	0.0009	-0.0031	0.0050	
dif social closeness		Alone-Together	RIFG~RTPJ	0.0009	-0.0040	0.0061	
Interactions							
dif social closeness	Int-Samegen	Alone	LIFG~RIFG	-0.0020	-0.0084	0.0043	
dif social closeness	Int-Samegen	Together	LIFG~RIFG	-0.0003	-0.0053	0.0047	
dif social closeness	Int-Samegen	Alone	LIFG~LIFG	-0.0044	-0.0132	0.0042	
dif social closeness	Int-Samegen	Together	LIFG~LIFG	-0.0017	-0.0084	0.0051	
dif social closeness	Int-Samegen	Alone	LTPJ~LTPJ	0.0044	-0.0045	0.0130	
dif social closeness	Int-Samegen	Together	LTPJ~LTPJ	-0.0065	-0.0131	0.0004	
dif social closeness	Int-Samegen	Alone	LIFG~LTPJ	0.0029	-0.0035	0.0094	
dif social closeness	Int-Samegen	Together	LIFG~LTPJ	-0.0008	-0.0058	0.0045	
dif social closeness	Int-Samegen	Alone	RIFG~RIFG	0.0018	-0.0080	0.0116	
dif social closeness	Int-Samegen	Together	RIFG~RIFG	0.0061	-0.0016	0.0137	
dif social closeness	Int-Samegen	Alone	RTPJ~RTPJ	0.0007	-0.0143	0.0151	
dif social closeness	Int-Samegen	Together	RTPJ~RTPJ	-0.0155	-0.0274	-0.0032	No overlap
dif social closeness	Int-Samegen	Alone	RIFG~LTPJ	0.0021	-0.0042	0.0087	
dif social closeness	Int-Samegen	Together	RIFG~LTPJ	0.0063	0.0011	0.0112	No overlap
dif social closeness	Int-Samegen	Alone	RIFG~RTPJ	-0.0049	-0.0130	0.0031	
dif social closeness	Int-Samegen	Together	RIFG~RTPJ	-0.0006	-0.0068	0.0059	
dif social closeness	Intergen	Alone-Together	LIFG~RIFG	0.0037	-0.0013	0.0088	
dif social closeness	Samegen	Alone-Together	LIFG~RIFG	0.0054	-0.0006	0.0112	
dif social closeness	Intergen	Alone-Together	LIFG~LIFG	0.0032	-0.0041	0.0103	
dif social closeness	Samegen	Alone-Together	LIFG~LIFG	0.0058	-0.0025	0.0139	
dif social closeness	Intergen	Alone-Together	LTPJ~LTPJ	0.0049	-0.0020	0.0122	
dif social closeness	Samegen	Alone-Together	LTPJ~LTPJ	-0.0059	-0.0142	0.0023	
dif social closeness	Intergen	Alone-Together	LIFG~LTPJ	0.0024	-0.0027	0.0076	
dif social closeness	Samegen	Alone-Together	LIFG~LTPJ	-0.0012	-0.0072	0.0047	

Measure	Group	Task	ROI Pairs	Estimate	Lower HPD	Upper HPD	No overlap
dif social closeness	Intergen	Alone-Together	RIFG~RIFG	0.0000	-0.0076	0.0080	
dif social closeness	Samegen	Alone-Together	RIFG~RIFG	0.0043	-0.0050	0.0134	
dif social closeness	Intergen	Alone-Together	RTPJ~RTPJ	0.0041	-0.0095	0.0190	
dif social closeness	Samegen	Alone-Together	RTPJ~RTPJ	-0.0120	-0.0241	0.0000	
dif social closeness	Intergen	Alone-Together	RIFG~LTPJ	-0.0011	-0.0064	0.0041	
dif social closeness	Samegen	Alone-Together	RIFG~LTPJ	0.0030	-0.0030	0.0092	
dif social closeness	Intergen	Alone-Together	RIFG~RTPJ	-0.0012	-0.0078	0.0060	
dif social closeness	Samegen	Alone-Together	RIFG~RTPJ	0.0031	-0.0043	0.0103	

S6 Appendix – Table R. HbR. Relationships between allophilia (sum) and INS in unstandardised units. Effects are presented in the following order: Main, group, task, and simple effects.

Measure	Group	Task	ROI Pairs	Estimate	Lower HPD	Upper HPD	No overlap
Main effect							
sum allophilia			LIFG~RIFG	0.0000	-0.0002	0.0001	
sum allophilia			LIFG~LIFG	0.0001	-0.0002	0.0003	
sum allophilia			LTPJ~LTPJ	0.0001	-0.0001	0.0003	
sum allophilia			LIFG~LTPJ	0.0001	0.0000	0.0003	
sum allophilia			RIFG~RIFG	0.0000	-0.0002	0.0003	
sum allophilia			RTPJ~RTPJ	0.0000	-0.0004	0.0004	
sum allophilia			RIFG~LTPJ	-0.0001	-0.0002	0.0001	
sum allophilia			RIFG~RTPJ	0.0000	-0.0002	0.0002	
Group effect							
sum allophilia	Intergen		LIFG~RIFG	0.0001	-0.0002	0.0003	
sum allophilia	Samegen		LIFG~RIFG	-0.0001	-0.0004	0.0001	
sum allophilia	Intergen		LIFG~LIFG	0.0000	-0.0002	0.0003	
sum allophilia	Samegen		LIFG~LIFG	0.0001	-0.0002	0.0004	
sum allophilia	Intergen		LTPJ~LTPJ	0.0002	0.0000	0.0005	
sum allophilia	Samegen		LTPJ~LTPJ	0.0000	-0.0003	0.0003	
sum allophilia	Intergen		LIFG~LTPJ	0.0001	-0.0001	0.0003	
sum allophilia	Samegen		LIFG~LTPJ	0.0001	-0.0001	0.0004	
sum allophilia	Intergen		RIFG~RIFG	0.0002	-0.0001	0.0005	
sum allophilia	Samegen		RIFG~RIFG	-0.0001	-0.0004	0.0002	
sum allophilia	Intergen		RTPJ~RTPJ	0.0001	-0.0005	0.0007	
sum allophilia	Samegen		RTPJ~RTPJ	-0.0001	-0.0006	0.0003	
sum allophilia	Intergen		RIFG~LTPJ	0.0000	-0.0002	0.0002	
sum allophilia	Samegen		RIFG~LTPJ	-0.0002	-0.0004	0.0001	
sum allophilia	Intergen		RIFG~RTPJ	0.0000	-0.0003	0.0003	
sum allophilia	Samegen		RIFG~RTPJ	0.0001	-0.0002	0.0003	
Task effect							
sum allophilia		Alone	LIFG~RIFG	-0.0001	-0.0003	0.0001	
sum allophilia		Together	LIFG~RIFG	0.0000	-0.0002	0.0002	
sum allophilia		Alone	LIFG~LIFG	0.0001	-0.0002	0.0004	
sum allophilia		Together	LIFG~LIFG	0.0000	-0.0002	0.0003	
sum allophilia		Alone	LTPJ~LTPJ	0.0003	0.0000	0.0006	
sum allophilia		Together	LTPJ~LTPJ	-0.0001	-0.0003	0.0002	
sum allophilia		Alone	LIFG~LTPJ	0.0001	-0.0001	0.0003	
sum allophilia		Together	LIFG~LTPJ	0.0001	0.0000	0.0003	
sum allophilia		Alone	RIFG~RIFG	-0.0001	-0.0004	0.0002	
sum allophilia		Together	RIFG~RIFG	0.0002	-0.0001	0.0005	
sum allophilia		Alone	RTPJ~RTPJ	-0.0004	-0.0010	0.0001	
sum allophilia		Together	RTPJ~RTPJ	0.0004	-0.0001	0.0009	
sum allophilia		Alone	RIFG~LTPJ	-0.0001	-0.0003	0.0001	
sum allophilia		Together	RIFG~LTPJ	-0.0001	-0.0003	0.0001	
sum allophilia		Alone	RIFG~RTPJ	-0.0001	-0.0004	0.0002	
sum allophilia		Together	RIFG~RTPJ	0.0001	-0.0001	0.0004	

Measure	Group	Task	ROI Pairs	Estimate	Lower HPD	Upper HPD	No overlap
Simple effect							
sum allophilia	Intergen	Alone	LIFG~RIFG	0.0000	-0.0003	0.0004	
sum allophilia	Intergen	Together	LIFG~RIFG	0.0001	-0.0002	0.0003	
sum allophilia	Samegen	Alone	LIFG~RIFG	-0.0003	-0.0006	0.0001	
sum allophilia	Samegen	Together	LIFG~RIFG	0.0000	-0.0003	0.0002	
sum allophilia	Intergen	Alone	LIFG~LIFG	-0.0001	-0.0005	0.0003	
sum allophilia	Intergen	Together	LIFG~LIFG	0.0001	-0.0002	0.0005	
sum allophilia	Samegen	Alone	LIFG~LIFG	0.0002	-0.0002	0.0007	
sum allophilia	Samegen	Together	LIFG~LIFG	-0.0001	-0.0004	0.0003	
sum allophilia	Intergen	Alone	LTPJ~LTPJ	0.0006	0.0002	0.0010	No overlap
sum allophilia	Intergen	Together	LTPJ~LTPJ	-0.0001	-0.0004	0.0002	
sum allophilia	Samegen	Alone	LTPJ~LTPJ	0.0000	-0.0005	0.0004	
sum allophilia	Samegen	Together	LTPJ~LTPJ	0.0000	-0.0003	0.0003	
sum allophilia	Intergen	Alone	LIFG~LTPJ	0.0000	-0.0003	0.0003	
sum allophilia	Intergen	Together	LIFG~LTPJ	0.0002	-0.0001	0.0004	
sum allophilia	Samegen	Alone	LIFG~LTPJ	0.0002	-0.0002	0.0005	
sum allophilia	Samegen	Together	LIFG~LTPJ	0.0001	-0.0002	0.0004	
sum allophilia	Intergen	Alone	RIFG~RIFG	0.0000	-0.0004	0.0005	
sum allophilia	Intergen	Together	RIFG~RIFG	0.0004	0.0000	0.0007	
sum allophilia	Samegen	Alone	RIFG~RIFG	-0.0002	-0.0007	0.0003	
sum allophilia	Samegen	Together	RIFG~RIFG	0.0000	-0.0004	0.0004	
sum allophilia	Intergen	Alone	RTPJ~RTPJ	-0.0001	-0.0010	0.0007	
sum allophilia	Intergen	Together	RTPJ~RTPJ	0.0004	-0.0004	0.0011	
sum allophilia	Samegen	Alone	RTPJ~RTPJ	-0.0007	-0.0014	0.0000	No overlap
sum allophilia	Samegen	Together	RTPJ~RTPJ	0.0005	-0.0001	0.0010	
sum allophilia	Intergen	Alone	RIFG~LTPJ	0.0000	-0.0003	0.0003	
sum allophilia	Intergen	Together	RIFG~LTPJ	0.0000	-0.0002	0.0002	
sum allophilia	Samegen	Alone	RIFG~LTPJ	-0.0002	-0.0005	0.0002	
sum allophilia	Samegen	Together	RIFG~LTPJ	-0.0001	-0.0004	0.0001	
sum allophilia	Intergen	Alone	RIFG~RTPJ	-0.0001	-0.0005	0.0004	
sum allophilia	Intergen	Together	RIFG~RTPJ	0.0000	-0.0003	0.0003	
sum allophilia	Samegen	Alone	RIFG~RTPJ	-0.0001	-0.0005	0.0003	
sum allophilia	Samegen	Together	RIFG~RTPJ	0.0002	-0.0001	0.0005	

S6 Appendix – Table S. HbR. Contrasts pertaining to allophilia (sum) ~ INS relationship in unstandardised units. Contrasts are presented in the following order: Group, task, and interaction contrasts.

Measure	Group	Task	ROI Pairs	Estimate	Lower HPD	Upper HPD	No overlap
Group contrast							
sum allophilia	Int-Samegen		LIFG~RIFG	0.0002	-0.0001	0.0005	
sum allophilia	Int-Samegen		LIFG~LIFG	-0.0001	-0.0004	0.0004	
sum allophilia	Int-Samegen		LTPJ~LTPJ	0.0003	-0.0001	0.0007	
sum allophilia	Int-Samegen		LIFG~LTPJ	0.0000	-0.0004	0.0003	
sum allophilia	Int-Samegen		RIFG~RIFG	0.0003	-0.0001	0.0008	
sum allophilia	Int-Samegen		RTPJ~RTPJ	0.0002	-0.0005	0.0010	
sum allophilia	Int-Samegen		RIFG~LTPJ	0.0002	-0.0001	0.0005	
sum allophilia	Int-Samegen		RIFG~RTPJ	-0.0001	-0.0005	0.0003	
Task contrast							
sum allophilia		Alone-Together	LIFG~RIFG	-0.0001	-0.0004	0.0001	
sum allophilia		Alone-Together	LIFG~LIFG	0.0001	-0.0004	0.0004	
sum allophilia		Alone-Together	LTPJ~LTPJ	0.0003	0.0000	0.0007	
sum allophilia		Alone-Together	LIFG~LTPJ	0.0000	-0.0003	0.0002	
sum allophilia		Alone-Together	RIFG~RIFG	-0.0003	-0.0007	0.0001	
sum allophilia		Alone-Together	RTPJ~RTPJ	-0.0008	-0.0015	-0.0001	No overlap
sum allophilia		Alone-Together	RIFG~LTPJ	0.0000	-0.0003	0.0003	
sum allophilia		Alone-Together	RIFG~RTPJ	-0.0002	-0.0005	0.0002	
Interactions							
sum allophilia	Int-Samegen	Alone	LIFG~RIFG	0.0003	-0.0002	0.0008	
sum allophilia	Int-Samegen	Together	LIFG~RIFG	0.0001	-0.0003	0.0005	
sum allophilia	Int-Samegen	Alone	LIFG~LIFG	-0.0003	-0.0009	0.0003	
sum allophilia	Int-Samegen	Together	LIFG~LIFG	0.0002	-0.0003	0.0007	
sum allophilia	Int-Samegen	Alone	LTPJ~LTPJ	0.0006	0.0000	0.0012	
sum allophilia	Int-Samegen	Together	LTPJ~LTPJ	-0.0001	-0.0006	0.0004	
sum allophilia	Int-Samegen	Alone	LIFG~LTPJ	-0.0002	-0.0007	0.0003	
sum allophilia	Int-Samegen	Together	LIFG~LTPJ	0.0001	-0.0003	0.0005	
sum allophilia	Int-Samegen	Alone	RIFG~RIFG	0.0003	-0.0004	0.0010	
sum allophilia	Int-Samegen	Together	RIFG~RIFG	0.0003	-0.0002	0.0009	
sum allophilia	Int-Samegen	Alone	RTPJ~RTPJ	0.0006	-0.0005	0.0017	
sum allophilia	Int-Samegen	Together	RTPJ~RTPJ	-0.0001	-0.0010	0.0008	
sum allophilia	Int-Samegen	Alone	RIFG~LTPJ	0.0002	-0.0003	0.0006	
sum allophilia	Int-Samegen	Together	RIFG~LTPJ	0.0001	-0.0002	0.0005	
sum allophilia	Int-Samegen	Alone	RIFG~RTPJ	0.0000	-0.0006	0.0006	
sum allophilia	Int-Samegen	Together	RIFG~RTPJ	-0.0002	-0.0007	0.0003	
sum allophilia	Intergen	Alone-Together	LIFG~RIFG	0.0000	-0.0004	0.0003	
sum allophilia	Samegen	Alone-Together	LIFG~RIFG	-0.0002	-0.0006	0.0002	
sum allophilia	Intergen	Alone-Together	LIFG~LIFG	-0.0002	-0.0007	0.0003	
sum allophilia	Samegen	Alone-Together	LIFG~LIFG	0.0003	-0.0003	0.0009	
sum allophilia	Intergen	Alone-Together	LTPJ~LTPJ	0.0007	0.0002	0.0012	No overlap
sum allophilia	Samegen	Alone-Together	LTPJ~LTPJ	0.0000	-0.0006	0.0005	
sum allophilia	Intergen	Alone-Together	LIFG~LTPJ	-0.0002	-0.0006	0.0002	
sum allophilia	Samegen	Alone-Together	LIFG~LTPJ	0.0001	-0.0003	0.0005	

Measure	Group	Task	ROI Pairs	Estimate	Lower HPD	Upper HPD	No overlap
sum allophilia	Intergen	Alone-Together	RIFG~RIFG	-0.0003	-0.0009	0.0002	
sum allophilia	Samegen	Alone-Together	RIFG~RIFG	-0.0003	-0.0009	0.0004	
sum allophilia	Intergen	Alone-Together	RTPJ~RTPJ	-0.0005	-0.0016	0.0006	
sum allophilia	Samegen	Alone-Together	RTPJ~RTPJ	-0.0012	-0.0020	-0.0003	No overlap
sum allophilia	Intergen	Alone-Together	RIFG~LTPJ	0.0000	-0.0004	0.0004	
sum allophilia	Samegen	Alone-Together	RIFG~LTPJ	0.0000	-0.0004	0.0004	
sum allophilia	Intergen	Alone-Together	RIFG~RTPJ	-0.0001	-0.0006	0.0004	
sum allophilia	Samegen	Alone-Together	RIFG~RTPJ	-0.0003	-0.0008	0.0002	

S6 Appendix – Table T. HbR. Relationships between allophilia (dif) and INS in unstandardised units. Effects are presented in the following order: Main, group, task, and simple effects.

Measure	Group	Task	ROI Pairs	Estimate	Lower HPD	Upper HPD	No overlap
Main effect							
dif allophilia			LIFG~RIFG	0.0000	-0.0002	0.0003	
dif allophilia			LIFG~LIFG	0.0001	-0.0002	0.0003	
dif allophilia			LTPJ~LTPJ	0.0001	-0.0002	0.0003	
dif allophilia			LIFG~LTPJ	-0.0001	-0.0003	0.0002	
dif allophilia			RIFG~RIFG	0.0001	-0.0002	0.0004	
dif allophilia			RTPJ~RTPJ	-0.0002	-0.0006	0.0003	
dif allophilia			RIFG~LTPJ	-0.0001	-0.0003	0.0001	
dif allophilia			RIFG~RTPJ	0.0000	-0.0002	0.0003	
Group effect							
dif allophilia	Intergen		LIFG~RIFG	0.0002	-0.0001	0.0005	
dif allophilia	Samegen		LIFG~RIFG	-0.0001	-0.0004	0.0002	
dif allophilia	Intergen		LIFG~LIFG	0.0002	-0.0002	0.0006	
dif allophilia	Samegen		LIFG~LIFG	-0.0001	-0.0005	0.0003	
dif allophilia	Intergen		LTPJ~LTPJ	0.0004	0.0000	0.0007	No overlap
dif allophilia	Samegen		LTPJ~LTPJ	-0.0002	-0.0006	0.0001	
dif allophilia	Intergen		LIFG~LTPJ	0.0001	-0.0002	0.0003	
dif allophilia	Samegen		LIFG~LTPJ	-0.0002	-0.0005	0.0001	
dif allophilia	Intergen		RIFG~RIFG	-0.0002	-0.0006	0.0002	
dif allophilia	Samegen		RIFG~RIFG	0.0004	-0.0001	0.0008	
dif allophilia	Intergen		RTPJ~RTPJ	0.0002	-0.0004	0.0007	
dif allophilia	Samegen		RTPJ~RTPJ	-0.0005	-0.0011	0.0001	
dif allophilia	Intergen		RIFG~LTPJ	-0.0002	-0.0005	0.0001	
dif allophilia	Samegen		RIFG~LTPJ	0.0001	-0.0002	0.0004	
dif allophilia	Intergen		RIFG~RTPJ	-0.0002	-0.0005	0.0002	
dif allophilia	Samegen		RIFG~RTPJ	0.0002	-0.0002	0.0006	
Task effect							
dif allophilia		Alone	LIFG~RIFG	-0.0002	-0.0005	0.0001	
dif allophilia		Together	LIFG~RIFG	0.0003	0.0000	0.0005	
dif allophilia		Alone	LIFG~LIFG	0.0000	-0.0004	0.0004	
dif allophilia		Together	LIFG~LIFG	0.0001	-0.0002	0.0005	
dif allophilia		Alone	LTPJ~LTPJ	-0.0001	-0.0005	0.0003	
dif allophilia		Together	LTPJ~LTPJ	0.0002	-0.0001	0.0006	
dif allophilia		Alone	LIFG~LTPJ	-0.0002	-0.0005	0.0002	
dif allophilia		Together	LIFG~LTPJ	0.0000	-0.0002	0.0003	
dif allophilia		Alone	RIFG~RIFG	0.0000	-0.0005	0.0004	
dif allophilia		Together	RIFG~RIFG	0.0002	-0.0002	0.0006	
dif allophilia		Alone	RTPJ~RTPJ	-0.0001	-0.0007	0.0005	
dif allophilia		Together	RTPJ~RTPJ	-0.0002	-0.0008	0.0003	
dif allophilia		Alone	RIFG~LTPJ	-0.0001	-0.0004	0.0002	
dif allophilia		Together	RIFG~LTPJ	0.0000	-0.0003	0.0002	
dif allophilia		Alone	RIFG~RTPJ	-0.0001	-0.0005	0.0002	
dif allophilia		Together	RIFG~RTPJ	0.0002	-0.0001	0.0005	

Measure	Group	Task	ROI Pairs	Estimate	Lower HPD	Upper HPD	No overlap
Simple effect							
dif allophilia	Intergen	Alone	LIFG~RIFG	0.0000	-0.0004	0.0004	
dif allophilia	Intergen	Together	LIFG~RIFG	0.0004	0.0000	0.0007	
dif allophilia	Samegen	Alone	LIFG~RIFG	-0.0004	-0.0008	0.0001	
dif allophilia	Samegen	Together	LIFG~RIFG	0.0001	-0.0002	0.0005	
dif allophilia	Intergen	Alone	LIFG~LIFG	0.0001	-0.0005	0.0007	
dif allophilia	Intergen	Together	LIFG~LIFG	0.0002	-0.0002	0.0007	
dif allophilia	Samegen	Alone	LIFG~LIFG	-0.0001	-0.0008	0.0005	
dif allophilia	Samegen	Together	LIFG~LIFG	0.0000	-0.0004	0.0005	
dif allophilia	Intergen	Alone	LTPJ~LTPJ	0.0001	-0.0005	0.0006	
dif allophilia	Intergen	Together	LTPJ~LTPJ	0.0007	0.0002	0.0012	No overlap
dif allophilia	Samegen	Alone	LTPJ~LTPJ	-0.0002	-0.0008	0.0004	
dif allophilia	Samegen	Together	LTPJ~LTPJ	-0.0002	-0.0007	0.0002	
dif allophilia	Intergen	Alone	LIFG~LTPJ	0.0001	-0.0003	0.0005	
dif allophilia	Intergen	Together	LIFG~LTPJ	0.0000	-0.0003	0.0004	
dif allophilia	Samegen	Alone	LIFG~LTPJ	-0.0004	-0.0008	0.0001	
dif allophilia	Samegen	Together	LIFG~LTPJ	0.0000	-0.0004	0.0004	
dif allophilia	Intergen	Alone	RIFG~RIFG	-0.0005	-0.0011	0.0001	
dif allophilia	Intergen	Together	RIFG~RIFG	0.0001	-0.0004	0.0006	
dif allophilia	Samegen	Alone	RIFG~RIFG	0.0004	-0.0002	0.0011	
dif allophilia	Samegen	Together	RIFG~RIFG	0.0003	-0.0002	0.0008	
dif allophilia	Intergen	Alone	RTPJ~RTPJ	-0.0001	-0.0010	0.0007	
dif allophilia	Intergen	Together	RTPJ~RTPJ	0.0005	-0.0003	0.0012	
dif allophilia	Samegen	Alone	RTPJ~RTPJ	-0.0001	-0.0010	0.0009	
dif allophilia	Samegen	Together	RTPJ~RTPJ	-0.0009	-0.0017	-0.0001	No overlap
dif allophilia	Intergen	Alone	RIFG~LTPJ	-0.0002	-0.0006	0.0002	
dif allophilia	Intergen	Together	RIFG~LTPJ	-0.0003	-0.0006	0.0001	
dif allophilia	Samegen	Alone	RIFG~LTPJ	0.0000	-0.0004	0.0004	
dif allophilia	Samegen	Together	RIFG~LTPJ	0.0002	-0.0001	0.0005	
dif allophilia	Intergen	Alone	RIFG~RTPJ	-0.0003	-0.0008	0.0002	
dif allophilia	Intergen	Together	RIFG~RTPJ	-0.0001	-0.0005	0.0003	
dif allophilia	Samegen	Alone	RIFG~RTPJ	0.0000	-0.0006	0.0005	
dif allophilia	Samegen	Together	RIFG~RTPJ	0.0004	0.0000	0.0009	No overlap

Supplementary Materials: Moffat, Dumas and Cross (2026). *Social interactions between people of same and different generations shape longitudinal changes in interpersonal neural synchrony, loneliness, and social connection.*

S6 Appendix – Table U. HbR. Contrasts pertaining to allophilia (dif) ~ INS relationship in unstandardised units. Contrasts are presented in the following order: Group, task, and interaction contrasts.

Measure	Group	Task	ROI Pairs	Estimate	Lower HPD	Upper HPD	No overlap
Group contrast							
dif allophilia	Int-Samegen		LIFG~RIFG	0.0003	-0.0001	0.0007	
dif allophilia	Int-Samegen		LIFG~LIFG	0.0002	-0.0003	0.0008	
dif allophilia	Int-Samegen		LTPJ~LTPJ	0.0006	0.0001	0.0012	No overlap
dif allophilia	Int-Samegen		LIFG~LTPJ	0.0003	-0.0002	0.0007	
dif allophilia	Int-Samegen		RIFG~RIFG	-0.0006	-0.0012	0.0000	
dif allophilia	Int-Samegen		RTPJ~RTPJ	0.0006	-0.0002	0.0015	
dif allophilia	Int-Samegen		RIFG~LTPJ	-0.0003	-0.0007	0.0001	
dif allophilia	Int-Samegen		RIFG~RTPJ	-0.0004	-0.0009	0.0001	
Task contrast							
dif allophilia		Alone-Together	LIFG~RIFG	-0.0004	-0.0008	-0.0001	No overlap
dif allophilia		Alone-Together	LIFG~LIFG	-0.0002	-0.0007	0.0004	
dif allophilia		Alone-Together	LTPJ~LTPJ	-0.0003	-0.0008	0.0002	
dif allophilia		Alone-Together	LIFG~LTPJ	-0.0002	-0.0005	0.0002	
dif allophilia		Alone-Together	RIFG~RIFG	-0.0002	-0.0008	0.0003	
dif allophilia		Alone-Together	RTPJ~RTPJ	0.0001	-0.0007	0.0010	
dif allophilia		Alone-Together	RIFG~LTPJ	0.0000	-0.0004	0.0003	
dif allophilia		Alone-Together	RIFG~RTPJ	-0.0003	-0.0008	0.0001	
Interactions							
dif allophilia	Int-Samegen	Alone	LIFG~RIFG	0.0004	-0.0002	0.0010	
dif allophilia	Int-Samegen	Together	LIFG~RIFG	0.0002	-0.0003	0.0007	
dif allophilia	Int-Samegen	Alone	LIFG~LIFG	0.0003	-0.0006	0.0011	
dif allophilia	Int-Samegen	Together	LIFG~LIFG	0.0002	-0.0004	0.0009	
dif allophilia	Int-Samegen	Alone	LTPJ~LTPJ	0.0003	-0.0005	0.0011	
dif allophilia	Int-Samegen	Together	LTPJ~LTPJ	0.0009	0.0003	0.0016	No overlap
dif allophilia	Int-Samegen	Alone	LIFG~LTPJ	0.0005	-0.0002	0.0011	
dif allophilia	Int-Samegen	Together	LIFG~LTPJ	0.0001	-0.0005	0.0006	
dif allophilia	Int-Samegen	Alone	RIFG~RIFG	-0.0010	-0.0019	-0.0001	No overlap
dif allophilia	Int-Samegen	Together	RIFG~RIFG	-0.0002	-0.0009	0.0005	
dif allophilia	Int-Samegen	Alone	RTPJ~RTPJ	-0.0001	-0.0014	0.0012	
dif allophilia	Int-Samegen	Together	RTPJ~RTPJ	0.0013	0.0003	0.0025	No overlap
dif allophilia	Int-Samegen	Alone	RIFG~LTPJ	-0.0002	-0.0008	0.0004	
dif allophilia	Int-Samegen	Together	RIFG~LTPJ	-0.0005	-0.0010	0.0000	
dif allophilia	Int-Samegen	Alone	RIFG~RTPJ	-0.0003	-0.0010	0.0004	
dif allophilia	Int-Samegen	Together	RIFG~RTPJ	-0.0005	-0.0011	0.0001	
dif allophilia	Intergen	Alone-Together	LIFG~RIFG	-0.0003	-0.0009	0.0002	
dif allophilia	Samegen	Alone-Together	LIFG~RIFG	-0.0005	-0.0010	0.0000	
dif allophilia	Intergen	Alone-Together	LIFG~LIFG	-0.0001	-0.0008	0.0006	
dif allophilia	Samegen	Alone-Together	LIFG~LIFG	-0.0002	-0.0010	0.0006	
dif allophilia	Intergen	Alone-Together	LTPJ~LTPJ	-0.0006	-0.0013	0.0001	
dif allophilia	Samegen	Alone-Together	LTPJ~LTPJ	0.0000	-0.0008	0.0007	
dif allophilia	Intergen	Alone-Together	LIFG~LTPJ	0.0000	-0.0005	0.0005	
dif allophilia	Samegen	Alone-Together	LIFG~LTPJ	-0.0004	-0.0009	0.0002	

Measure	Group	Task	ROI Pairs	Estimate	Lower HPD	Upper HPD	No overlap
dif allophilia	Intergen	Alone-Together	RIFG~RIFG	-0.0006	-0.0014	0.0001	
dif allophilia	Samegen	Alone-Together	RIFG~RIFG	0.0002	-0.0006	0.0009	
dif allophilia	Intergen	Alone-Together	RTPJ~RTPJ	-0.0006	-0.0017	0.0006	
dif allophilia	Samegen	Alone-Together	RTPJ~RTPJ	0.0008	-0.0003	0.0020	
dif allophilia	Intergen	Alone-Together	RIFG~LTPJ	0.0001	-0.0004	0.0006	
dif allophilia	Samegen	Alone-Together	RIFG~LTPJ	-0.0002	-0.0007	0.0004	
dif allophilia	Intergen	Alone-Together	RIFG~RTPJ	-0.0002	-0.0008	0.0004	
dif allophilia	Samegen	Alone-Together	RIFG~RTPJ	-0.0004	-0.0011	0.0002	
