# Supplementary material for: Social interactions between people of same and different generations shape longitudinal changes in interpersonal neural synchrony, loneliness, and social connection
Source: PLoS Biol. 2026 Aug 20;24(8):e3003899. doi: 10.1371/journal.pbio.3003899 (PMC13492753; doi:10.1371/journal.pbio.3003899)
Supplement: Supplementary file 2 [file pbio.3003899.s004.pdf]

Dear Dr. Schnell,

We are pleased to submit our revised manuscript entitled "Longitudinal intergenerational hyperscanning indexes changes in social connection" for your consideration.

In response to the reviewer's constructive comments, we have made changes and additions to strengthen the manuscript. We have paid particular attention to improving transparency regarding preregistration, clarifying our statistical approach (including the presentation of null results in this approach), and bringing our guiding theoretical framework to the forefront.

We also found and corrected a small error in Figure 1. The red bar showing a task difference for the intergenerational group was previously in the wrong panel. We have painstakingly reviewed all figures and results in the main and supplementary documents.

Below, we provide detailed responses to each of the reviewers' comments. References to the new (tracked) manuscript are made by line number (e.g., Line XX). The reviewers' comments are provided in *blue italics*. Our responses and excerpts from the original manuscript are in black. New or altered text excerpts are presented in red. We use grey highlighting to show where small, important changes were made to existing text. In light of the additional analyses and changes to the manuscript, the order of supplementary figures and tables has changed. We direct the Reviewers to the new numbers.

We appreciate the time and thoughtful feedback provided by the reviewers and hope that the revised manuscript will now be suitable for publication.

Thank you very much for considering our work.

Yours sincerely,

Ryssa Moffat, Emily S. Cross and Guillaume Dumas

---

***Reviewer #1:***
*This manuscript addresses an important and timely topic and makes a potentially valuable contribution to social neuroscience. The longitudinal hyperscanning design is a clear strength, as multi-session interbrain studies remain rare. The inclusion of older adults further enhances the relevance of the work.*
*However, several conceptual and analytical issues substantially limit the interpretability of the findings. In its current form, the manuscript reads largely exploratory, despite being preregistered.*

We thank Reviewer 1 for this positive and thoughtful assessment of our manuscript.
We will respond to comments 1 and 3 together as they are closely related.

*1. A major concern is the limited alignment between the preregistered hypotheses and the reported analyses. The preregistration explicitly stated that interpersonal synchrony (INS) would increase across repeated interactions, particularly in intergenerational dyads. Instead, INS decreased across sessions in intergenerational dyads. This reversal of the primary hypothesis is not adequately addressed or theoretically reconciled. In addition, there appear to be further discrepancies between the preregistration and the manuscript. For example, the preregistration specifies an age cutoff of 70+ for older adults, whereas the manuscript reports 69+. Such deviations may be minor, but they should be explicitly documented and justified to maintain transparency and credibility. Moreover, no specific hypotheses were formulated for ROI-level effects. As a result, the numerous ROI-specific findings are de facto exploratory. This should be clearly acknowledged, and the confirmatory versus exploratory components of the manuscript should be more transparently separated.*

*3. The analytical scope is extremely broad: multiple ROI pairs, two tasks, two signal types, longitudinal slopes, interaction models, and several self-report measures (+ non-reported measures like empathy). This creates a highly complex analytical space with a substantial risk of overinterpretation. This concern is amplified by the*

*fact that the preregistered behavioral hypothesis (movement synchrony) is not reported. The omission of the hypothesized behavioral outcome further shifts the manuscript toward exploratory neural analyses. A clearer prioritization of analyses and stronger constraints on interpretation are needed.*

**Preregistration of exploratory research.** Our research group preregisters both confirmatory and exploratory research, for the reasons stated in a PLOS Biology editorial by Ulrik Dirnagl (2020). Nonetheless, we acknowledge that we regretfully neglected to clearly specify in the initial preregistration that the entirety of this project is exploratory. To address this oversight, we now include an additional section in the Methods, where we transparently report the oversight and explicitly detail deviations from the preregistration. Reference number 110 in manuscript = Lakens (2022).

> Lines 659-678, Methods:
> "**5.1 Preregistration**
> Prior to data collection, we preregistered our research plan to collect a multimodal data set (including fNIRS recordings, motion capture, self-report measures, behavioural collaboration tasks, as well as the experimenters' subjective ratings of collaboration). We specified that we would likely disseminate our findings across multiple publications, examining specific analyses in individual papers, due to the scale and complexity of the data collected. Due to the lack of existing research on longitudinal hyperscanning and intergenerational hyperscanning, we conceptualised this research as exploratory (though we regrettably did not explicitly state this in our preregistration). Nonetheless, our preregistration includes broadly formulated predictions. Supplementary Methods Table 1 details where each component of the preregistered analyses can be found (i.e., already disseminated or in progress), and summarises the alignment of our preregistered predictions with our findings.
>
> **Preregistered sample size.** We aimed to collect useable data from a minimum of 30 same generation and 30 intergenerational dyads between November 2023 and June 2024. This minimum reflected our best estimation of how we could maximise our financial, temporal, and human resources to address our preregistered research questions (110). Data collection began on November 15, 2023 and ended on June 18, 2024 as planned (Supplementary Methods Figure 4A).
>
> **Deviations from preregistration.** We intended to include only older adults aged 70+ years, however due to the difficulty during the recruitment process, we were concerned that we would not meet our pre-registered minimum of 30 intergenerational dyads and therefore included one 69-year-old participant.
>
> Lines 27-28, Abstract: "This exploratory research reinforces the multi-faceted nature of INS dynamics as social connections are forged."
>
> Lines 227-228, Present study: "In this preregistered study (https://osf.io/hz6tm), we undertook exploratory analyses to achieve three aims:"
>
> Lines 532-533, Discussion: "We preregistered and now report exploratory analyses of INS that include non-homologous ROI pairs (e.g., LIFG~RIFG) and offer possible reasoning for observations of heightened INS."

**Interpretation of exploratory results.** In drafting and revising this manuscript, we as authors have taken care not to over interpret. We also believe that emphasising the exploratory nature of this research helps remind the readers not to overinterpret our findings.

**Unreported data types.** As noted by Reviewer 1, we did not analyse the movement or empathy data described in the preregistration. We do not consider this to be an omission, as our preregistration specifically states that "we reserve[d] the option to include other factors such as gender in our analyses and anticipate that this study might be reported across several papers". We look forward to analysing the movement data in the near future.

*2. The manuscript frequently distinguishes between "substantial effects" and "no effect," but this binary framing obscures effect magnitude and evidential strength. Statements such as "intergenerational dyads reported feeling substantially less lonely" lack contextualization. Substantial in what sense? How meaningful is it in practical terms? Crucially, Bayes Factors are not reported. If conclusions such as "INS is largely comparable between groups" are drawn, the strength of evidence for the null hypothesis should be quantified. Without this, claims of*

*comparability remain ambiguous. In addition, the rationale for the sample size is not clearly justified. In a Bayesian framework, evidential strength depends in part on prior assumptions; without a transparent discussion of priors and design considerations, it remains difficult to evaluate how informative the data truly are for both null and alternative effects.*

We thank Reviewer 1 for their close attention to our analysis approach. We respond to the components of this comment individually.

**Estimation as opposed to hypothesis testing.** We take a Bayesian estimation approach, where a central aim is to describe the sign, magnitude, and uncertainty of effect sizes in unstandardized, raw units. In contrast to Bayesian hypothesis testing approaches, the Bayesian estimation approach does not seek to test hypotheses or to quantify the strength of evidence for or against hypotheses. Kruschke and Liddell (2018) provide an accessible overview of Bayesian and Frequentist estimation approaches and hypothesis testing approaches, arguing that Bayesian estimation approaches are useful for disseminating estimates with quantified uncertainty to enhance a field's understanding of phenomena of interest. Using this estimation approach, "we can assess the extent to which particular values of interest, such as null values, are among the most credible values" (Kruschke & Liddell, 2018, p. 184). This approach does not permit comparison of rival hypotheses. Should we find an effect with the distribution centered on 0, we would conclude with our evidence that there is no effect. We therefore present our results as recommended by proponents of Bayesian estimation (Kruschke & Liddell, 2018; McElreath, 2020): we describe distributions using a point estimate and credible intervals (95% HPD) in figure and table format and provide the code to generate complete distributions for closer inspection. We adapted the last part of the Introduction and the Methods section where we describe our approach, to make this more explicit and accessible. Reference numbers in excerpts from the manuscript: 89 = Kruschke & Liddell (2018), 90 = McElreath (2020), 91 = Cumming (2014), 92 = Frank et al. (2025).

> Lines 242-253, Introduction: "We employed a *Bayesian estimation* (as opposed to hypothesis testing) approach (89–92). Specifically, we built hierarchical models to estimate the magnitude and uncertainty of estimates using posterior predictive distributions. We summarise the distributions of model parameters and contrasts using point estimates and 95% credible intervals called 95% HPDs (highest posterior density regions). We use this estimation approach to assess the sign, magnitude and uncertainty of parameter estimates from our models and to assess the extent to which values of interest (e.g., 0, reflecting the absence of differences between groups or drawing conditions) are among the most credible values in a distribution (89). For example, if we compute a contrast between drawing alone and together, we obtain a distribution of the predicted difference between conditions. If the 95% HPD for this distribution excludes 0, we infer a difference between conditions. Aligned with the estimation approach (89–92), we do not compute Bayes factors."
>
> Lines 826-845, Methods: "For our preregistered statistical analyses, we take a Bayesian estimation approach, where, as we summarised in the Introduction, a central aim is to describe the sign, magnitude, and uncertainty of effect sizes in unstandardized, raw units (89–92). In contrast to (Bayesian or Frequentist) hypothesis testing approaches, the Bayesian estimation approach does not seek to test hypotheses or to quantify the strength of evidence for or against hypotheses. Kruschke and Liddell (89) provide an accessible overview of Bayesian and Frequentist estimation approaches and hypothesis testing approaches, arguing that Bayesian estimation approaches are useful for disseminating estimates with quantified uncertainty to enhance a field's understanding of phenomena of interest. Kruschke and Liddell also describe how "we can assess the extent to which particular values of interest, such as null values, are among the most credible values" (89, p. 184) using this estimation approach. In practical terms, when we compute a contrast between the posterior predictive distributions for drawing alone and together, we obtain a distribution of the predicted difference between conditions. If the 95% HPD for this distribution excludes 0, we infer a difference between conditions. We therefore present our results as recommended by proponents of Bayesian estimation (89,90,92), we describe distributions using a point estimate and credible intervals (95% HPD) in figure and table format and provide our code (https://osf.io/xcgp6/) to generate complete distributions for closer inspection."
>
> Lines 307-318, Reminder to readers before INS results (grey highlights the specific sections that were altered): "The first step in our analysis of INS was to perform contrasts between the posterior predictive distributions computed for real and pseudo dyads' INS levels. We first did this for all sessions combined and then for changes in INS levels across sessions per combination of ROI pair, task, and group. These contrasts serve to assess whether the level of observed INS (or the slope of

change in INS) differs meaningfully from a null distribution of INS levels (or slopes) made up of dyads who never interacted with one another. Further statistical analyses can then be performed on the ROI pairs where the posterior predictive distributions for real and pseudo dyads differ (i.e., the 95% HPD of the distribution of the difference excludes zero). To summarise these distributions for model parameters (e.g., INS levels for real dyads) and for contrasts (e.g., the difference between dyad types), we report point estimates from models and contrasts with 95% HPD in Supplementary Results Tables 1-4."

**Binary framing.** In light of our analysis approach, we agree that binary description as presented in the original version of the manuscript was not optimal. We began using "substantial" in our reporting, when in previous publication experiences, reviewers have requested heuristics for readers who are less familiar with Bayesian estimation. We are pleased to have the opportunity to remove "substantial" throughout the manuscript and supplementary material. We have also taken care to rephrase instances of "no effect". We have also added qualitative descriptions to help readers make sense of the effect sizes (see a few examples below).

> Lines 265-271: "**Loneliness.** At the beginning of the first session, both groups reported mid-low levels of loneliness (intergenerational mean = 1.50, SD = 1.30; same generation mean = 2.33, SD = 1.65 on a scale from 0 'least lonely' to 6 'most lonely'). Contrasts indicate that at the first session and for all sessions combined, intergenerational dyads reported feeling less lonely than same generation dyads (session 1 = 0.68 and combined sessions = 0.61 points higher). Across sessions, feelings of loneliness showed small reductions for both groups (~ 1 % per session), with no observed difference in rate of reduction between groups.

> Lines 385-391: "We observed greater INS for drawing together than alone in LTPJ~LTPJ, LIFG~RIFG, RIFG~LTPJ and RIFG~RTPJ. With respect to the interaction between group and task, we found that in LTPJ~LTPJ, INS was greater for drawing together than alone for intergenerational dyads. In RIFG~RTPJ, INS was greater for drawing together than alone for same generation dyads. These differences correspond to ~0.01 difference in coupling (equivalent to 39 % of the range between the minimum and maximum 95% HPD value observed in real and pseudo dyads for all sessions combined; Supplementary Results Table 1)."

> Lines 404-408: "Intergenerational dyads showed more positive INS slopes than same generation dyads when drawing alone in LIFG~RTPJ and RIFG~RTPJ. These differences correspond to changes in the magnitude of ~0.006 to 0.011 change in coupling from one session to the next (equivalent to 32 % of the range between the minimum and maximum 95% HPD values observed in real and pseudo dyads for all sessions combined; Supplementary Results Table 1)."

**Priors.** We thank Reviewer 1 for the encouragement to describe our priors in greater detail. We have added the priors for each analysis step in Supplementary Methods Table 4 and information about our prior selection in the Methods. Reference numbers in excerpts from the manuscript: 89 = Kruschke & Liddell (2018), 90 = McElreath (2020).

> Lines 843-845: "We defined weakly informed priors for our models (reported in Supplementary Methods Table 4), thereby imposing a constrained distribution on our expected results (89,90).

**Sample size justification.** Given the resource-intensive nature of this project, we determined the sample size that would allow us to address our research questions and maximise our financial, temporal and human resources. This approach is described by Lakens (2022) as "choosing a sample size based on resource constraints". Though Lakens' article is aimed primarily at Frequentist hypothesis testing approaches, we still follow the relevant recommendations for a Bayesian estimation approach, including report confidence intervals and considering how this work will be valuable for metanalyses, as other longitudinal studies of INS emerge. Our preregistration (posted on November 13, 2023) states "We aim to collect as many useable datasets as possible by June 2024. As a minimum, we hope to analyse data from 30 dyads per group. That is, 30 dyads in which both members are aged 18-35 and 30 dyads with one member aged 18-35 and one aged 70+". To align the preregistration with the manuscript, we have added the text below to the methods. Reference number 110 in manuscript = Lakens (2022).

> Lines 670-674: "**Preregistered sample size.** We aimed to collect useable data from a minimum of 30 same generation and 30 intergenerational dyads between November 2023 and June 2024. This minimum reflected our best estimation of how we could maximise our financial, temporal, and human

resources to address our preregistered research questions (Lakens, 2022). Data collection began on November 15, 2023 and ended on June 18, 2024 as planned (Supplementary Methods Figure 4A)."

*4. The manuscript does not sufficiently characterize baseline differences between groups. If intergenerational and same-generation dyads differed in loneliness or social closeness at baseline, these differences may partly drive longitudinal effects. Additionally, the drawing sessions are framed as an intervention to increase social connection, yet it remains unclear whether participants were meaningfully lonely to begin with. Without clearer baseline characterization, the interpretation of change effects remains limited.*

**Baseline scores.** We appreciate the suggestion to integrate richer baseline information into our interpretation of the overall findings. We have added the point estimates from models and contrasts to Table 1, to further contextualise the findings for all sessions combined and changes across sessions. We also refer to the first sessions score in the text reporting our findings.

Lines 265-271, example from results text: "**Loneliness.** At the beginning of the first session, both groups reported mid-low levels of loneliness (intergenerational mean = 1.50, SD = 1.30; same generation mean = 2.33, SD = 1.65 on a scale from 0 'least lonely' to 6 'most lonely'). Contrasts indicate that at the first session and for all sessions combined, intergenerational dyads reported feeling less lonely than same generation dyads (session 1 = 0.68 and combined sessions = 0.61 points higher). Across sessions, feelings of loneliness showed small reductions for both groups (~ 1 % per session), with no observed difference in rate of reduction between groups."

Lines 288-293: "**Table 1.** Unstandardised point estimates from models and group contrasts per self-reported measure with 95% HPD in square brackets. Point estimates for 1) the first session, 2) all six sessions combined, and 3) the slope of change across sessions. Self-report measures are loneliness, social closeness, and attitudes toward generations (allophilia). Visualisations of distributions for each measure and trajectory across sessions by age (i.e., older or younger adult) and group in Supplementary Results Figures 1 and 2."

| | **Loneliness** | **Social Closeness** | **Allophilia** |
|---|---|---|---|
| **First session** | | | |
| Intergen | 1.54 [1.16, 1.90] | 3.32 [2.97, 3.69] | -0.56 [-3.90, 2.74] |
| Same gen | 2.22 [1.85, 2.59] | 2.87 [2.51, 3.24] | -5.20 [-8.60, -1.90] |
| Intergen-Same gen | -0.68 [-1.19, -0.15] | 0.45 [-0.06, 0.96] | 4.65 [0.05, 8.97] |
| **Sessions combined** | | | |
| Intergen | 1.28 [1.02, 1.54] | 4.08 [3.76, 4.39] | -1.19 [-3.45, 1.05] |
| Same gen | 1.89 [1.62, 2.16] | 3.52 [3.19, 3.38] | -5.66 [-7.93, 3.28] |
| Intergen-Same gen | -0.61 [-0.99, -0.25] | 0.56 [0.10, 1.00] | 4.48 [1.29, 7.56] |
| **Change across six sessions** | | | |
| Intergen | -0.06 [-0.10, -0.02] | 0.24 [0.19, 0.27] | -0.21 [-0.55, 0.12] |
| Same gen | -0.07 [-.11, - 0.03] | 0.25 [0.20, 0.30] | -0.02 [-0.37, 0.30] |
| Intergen-Same gen | 0.01 [-0.05, 0.07] | -0.01 [-0.08, 0.07] | -0.18 [-0.66, 0.28] |

**Framing of drawing sessions.** Our internal framing was that we were replicating existing programs aiming to mitigate loneliness, rather than hosting a program specifically targeting lonely individuals. This framing allowed us to explore how such interventions achieve changes in loneliness among non-socially isolated community-dwelling members in a controlled experimental setting. For participants, we framed the experience very broadly as a study about social interactions. In the original manuscript, this is expressed at the end of the Introduction and at the beginning of the discussion to remind readers of our framing.

> Lines 256-257, Introduction: "Our findings pertaining to INS in intergenerational and same generation dyads, tracked across 6 sessions in a real-world context resembling a community art program…."
>
> Lines 480-481, Discussion: "We invited intergenerational and same generation dyads to complete six sessions of creative drawing (resembling a community art program)…"

*5. The abstract claims that the findings "can inform program design choices," yet the manuscript does not clearly articulate which design elements should be altered based on the results. Given the mixed and partially contradictory INS trajectories, the translational implications appear overstated.*

We have removed this statement.

*6. There appears to be inconsistency between main text and supplementary results regarding loneliness effects, which requires clarification. Main text: "We observed no substantial main, task, or group effect on the relationship between INS and summed loneliness scores." Supplement: "We observed a substantial main effect, whereby greater loneliness predicted lower INS in LIFG~RIFG, as well as a matching substantial group effect for same generation dyads, and a matching substantial task effect for drawing alone."*

We report analyses for oxygenated haemoglobin (HbO) in the main manuscript and deoxygenated haemoglobin (HbR) in the Supplementary Materials. In the original manuscript, we direct readers to each set of results at the end of the Introduction and at the end of the Figure 1, 2, and 3 captions. Below is the text in the Introduction:

> Lines 240-242: "According to fNIRS best practices, we report both fNIRS signals (oxygenated haemoglobin [HbO] and deoxygenated haemoglobin [HbR]), with HbO results presented in the main manuscript and HbR results presented in the Supplementary Materials."

*7. It is also unclear whether prior acquaintance between dyad members or gender composition were statistically controlled. Given unequal gender distributions, this is a non-trivial issue.*

Indeed, while we agree this is a non-trivial issue, the number of pairing possibilities (and the number of dyads within each category) would add a large degree of complexity to our already complex models. As shown in Supplementary Methods Figure 1, there are eight possible gender compositions. We have refined the statement that balancing gender pairings was not prioritised during recruitment (Participants section):

> Lines 683-686: "Due to the logistical challenge posed by recruiting for and scheduling a multi-session study of this scale, specifically balanced or otherwise targeted gender pairings were not prioritised (see Supplementary Methods Figures 1 and 4 for summary of gender pairings and session spacing)."

We have also added the following to the Limitations so that readers are aware of the potential influence of gender pairings:

> Lines 625-630: "Another design limitation pertains to gender pairings. We could not control for differences resulting from same gender and different gender pairings, because the combination of different ages and different genders resulted in eight unique gender pairings, each with a small number of dyads (Supplementary Methods Figure 1). Future research could focus on specific intergenerational gender pairings to gain a deeper understanding of the role of gender in INS trajectories."

***Reviewer #2:***
*The manuscript reports rare longitudinal and intergenerational study using fNIRS hyperscanning. An impressive dataset from 61 dyads across 6 time points was collected, including young adult dyads and intergenerational younger-older adult dyads creating artworks together or alone. I think this is a very timely and interesting study with sound methods and exemplary open science practices. I should note that I am not an expert on Bayesian statistics, so I won't comment on the statistical analyses. While the manuscript is generally well-written, I do have some requests for clarification and recommendations.*

We thank Reviewer 2 for their encouraging evaluation of the study.

*Introduction:*
*1. The empirical background is nicely sketched out, but I was missing a guiding theoretical framework. Of course, in the hyperscanning literature there are many, so I'm not suggesting an exhaustive review. Rather, I'm interested in the authors' notion of why and how they would expect synchrony to emerge in their task and what could be the underlying psychological mechanisms, if any. I noted that predictability of behavior was mentioned in the introduction, which is of course discussed a lot in the INS literature, but I wasn't sure whether the authors suggest predictability/prediction as a core mechanism underlying INS. Please elaborate on your theoretical model. This will also help to contextualize and interpret the results in the discussion section which in part seems a bit speculative.*

We thank Reviewer 2 for this valuable feedback and respond first regarding the theoretical framework, then regarding our cautious interpretation of results.

**Theoretical framework.** We have made our guiding theoretical framework explicit by adding a paragraph to the Introduction describing two main frameworks and stating that we are working within the mutual prediction framework. We have taken the opportunity to clarify this throughout the introduction and discussion (see response to final comment from Reviewer 2). Reference numbers in excerpts from the manuscript: 40 = Hamilton (2021), 41 = Novembre & Iannetti (2021), 42 = Moreau & Dumas (2021), 43 = De Felice et al. (2025), 44 = Burgess (2013), 45 = Bilek et al. (2022), 46 = Wolpert et al. (2003), 47 = Kingsbury et al. (2019).

> Lines 94-105, new paragraph in Introduction: "The role of INS in social behaviour remains debated (40–43), with two main theoretical conceptualisations: common cognitive processing and mutual prediction. The common cognitive processing framework posits that interacting people perceive similar stimuli, which results in both people's brains responding in comparable ways at comparable time delays (44). This framework can explain why individuals viewing the same movie or simultaneously imitating an instructor may show heightened INS. Capturing the interactive and turn-based aspects of real social interactions, the mutual prediction framework posits that interacting people predict potential changes in the environment that they share, such as social information about each other, and that the interdependence of these continuous predictions leads to heightened INS (45–47). We embed our interpretation of INS during collaborative drawing in the mutual prediction framework, in that we would argue that collaborative drawing inherently involves different (even if complementary) stimuli for each participant created by the other."
>
> Lines 121-124 (grey highlights specific sections that were altered): "In combination, the findings from fNIRS and EEG hyperscanning studies indicate that INS levels during collaboration are shaped by processes such as joint attention and perspective taking, which underpin the mutual prediction framework (41)."
>
> Lines 207-209: "Within the framework of mutual prediction, we propose that social closeness and the degree of predictability of behaviour induced by the task contribute to the opposite INS gradients."

**Speculative results.** The results are in part speculative, as this research is exploratory and not strongly driven by directional hypotheses. As described in our response to Reviewer 1's first and third comment, we have added emphasis to the exploratory nature of the work throughout the manuscript.

*Methods/ Results:*
*2. The excluded dyads/ sessions are listed in the supplementary material, but I was wondering whether there were any differences in compliance/ dropout across the groups (same generation/ intergenerational).*

The dropout rate was quite low given the time-demands of the study, and similar across groups. In total, 7 dyads (3 same generation, 4 intergenerational) completed the initial session and then dropped out, typically because the weekly attendance did not fit in one individual's schedule. We have added this information in the Methods:

> Lines 691-693: "Seven additional dyads (3 same generation, 4 intergenerational) completed the initial session, then left the study due to scheduling difficulties. In accordance with our preregistration, their data are not included in the analysis."

*3. I suggest to briefly mention the instruments for the self-reports in the results section. I'm not sure all readers are familiar with the IOS, so the abbreviation has to be properly introduced.*

We thank Reviewer 2 for noting this detail. We removed the abbreviation, as it was not adding value or clarity to the methods section, and removing it helps with consistency across self-report measures.

*4. How exactly were the pseudo dyads created and how many?*

We describe the process of creating pseudo dyads in the Methods (section 5.5.1.2.) in the original manuscript. In the revised manuscript, we now add the number of pseudo dyads to the existing description in the Methods and added a short definition of pseudo dyads to the Results:

> Lines 815-818: "Pseudo dyads are dyads made by pairing participants who never interacted, but align with our group types (e.g., both younger adults, or one older and one younger adult). The numbers of pseudo dyads per group, session, and ROI are listed in Supplementary Methods Table 3 (mean = 3467, SD = 1621)."

> Lines 307-310: "The first step in our analysis of INS was to perform contrasts between the posterior predictive distributions computed for real and pseudo dyads' INS levels. Pseudo dyads consist of pairs of participants who never interacted, but match a group type (e.g., both younger adults, or one older and one younger adult)."

*5. Did you assess any more sociodemographic data that would allow to contextualize the results? E.g. socioeconomic status, education, cultural background…*

Unfortunately, we did not. As described in the methods, the 90 younger adults were recruited through a portal that is mainly used by university students to sign up for research studies. We touch on the value of richer sociodemographic and lived experience data in the discussion of the original manuscript and suggest that this data could absolutely deepen our understanding of mutual prediction as a mechanism for INS.

> Lines 599-604: "These findings can serve as a useful point of departure for research examining the impact of different sources of 'common ground' on INS. For example, studies could explore whether cumulative life experience (e.g., number of years lived) gives older adults an advantage for finding common ground and/or whether similarities in socialisation (e.g., culture, age, socio-economic status, hobbies) play bigger role in establishing common ground that could shape the mutual prediction underpinning INS levels."

*6. The description of the procedures is not entirely clear: Did the participants spend 5 minutes per drawing or five minutes in total drawing together and alone? I assume the former, but I wasn't sure based on the description.*

We thank Reviewer 2 for the recommendation to clarify the total time spent drawing.

> Lines 752-756: "Next, participants drew three drawings with oil pastels, without talking. Participants were allotted 5 min per drawing for each of 3 drawings, totalling 15 min of drawing per session. Participants were instructed that they would complete three drawings, the first drawing alone and the second and third together with their partner on a single piece of paper. They were instructed that they would have 5 min per drawing and could draw anything they liked."

*7. The sequence of conditions was fixed with participants always drawing alone first. This is a bit of a concern for the condition effect. Could increased INS when drawing together vs. alone be (in part) due to increasing INS levels over the course of each session? Have you looked at differences between the second and third drawing?*

We appreciate the encouragement to dig into this possibility. We conducted additional analyses, fitting models for all ROI pairs that differed between pseudo and real dyads for all sessions combined. Contrasts between the 1st and 2nd instances of drawing together did not show differences overall (groups considered together). We have reported these in Supplementary Results Table 7. We have also added a sentence with these findings in the Results:

> Lines 393-395: "We also contrasted the first and second instances of drawing together. We found no differences and ruled out order-related changes in INS level while drawing together (Supplementary Results Table 7)."

*8. Have there been any diversions from the pre-registration? If yes, these should be listed somewhere.*
We thank Reviewer 2 for raising this topic, which was also raised by Reviewer 1. To address this, we have added the text below to a new section dedicated to describing our preregistration:

> Lines 675-678: "**Deviations from preregistration.** We intended to include only older adults aged 70+ years. However, due to the difficulty of the recruitment process, we were concerned that we would not meet our preregistered minimum of 30 intergenerational dyads and therefore included one 69-year-old participant."

*Discussion:*
*9. As mentioned above, the manuscript would benefit from a clearer theoretical framework. Predictions are mentioned again in the discussion, but it is not clear how mutual predictability/ predictions relate to INS in the authors' view. Joint attention and arousal are also discussed, but given the complexities of functions associated with the involved brain areas that seems a bit speculative again. Also, INS in these regions does not equate to heightened activity, just more interpersonal alignment.*

We address this comment in two parts: the theoretical framework first, then the relationship between arousal and INS.

**Theoretical framework.** As in the Introduction, we have explicitly clarified our guiding theoretical framework. Specifically, we clarify that we view joint attention and perspective taking as processes likely to underpin mutual prediction (Bilek et al., 2022; Kingsbury et al., 2019; Wolpert et al., 2003), that merit further empirical investigation. Reference numbers in excerpts from the manuscript: 40 = Hamilton (2021), 45 = Bilek et al. (2022), 46 = Wolpert (2003), 47 = Kingsbury et al. (2019).

> Lines 538-541: "Considered in the context of the mutual prediction framework (45-47), these findings suggest that collaborative drawing results in heightened integration of social information (LTPJ~LTPJ) via joint attention (LIFG~RIFG), and perhaps, integration of the other person's maintenance of joint attention as social information (RIFG~LTPJ and RIFG~RTPJ)."

> Lines 546-548: "This interpretation requires further investigation using paradigms that can quantify joint attention and cognitive control, both of which would deepen our understanding of mutual prediction as a mechanism of INS."

> Lines 560-567: "We observed that while dyads drew together, ROI pairs that include right TPJ (RTPJ~RTPJ, LIFG~RTPJ, and RIFG~RTPJ) showed decreasing INS among intergenerational dyads but increasing INS among same generation dyads. This has potential implications for the mutual prediction framework that merit further investigation. For example, this may suggest that, in the context of collaborative drawing, intergenerational dyads' monitoring and integration of their partner's levels of joint attention may decrease with increasing familiarity, whereas same generation dyads may monitor each other's levels of joint attention more closely as familiarity increases."

Lines 576-579: "Greater similarity in dyadic levels of loneliness predicted greater INS when dyads drew together and lower INS when they drew alone. This may point to how differences in loneliness can shape processes involved in mutual prediction, such as joint attention and perspective taking (40)."

**Arousal and INS.** There is mounting evidence of an association between arousal and INS that is complemented by behaviours (Bast et al., 2025; Lachat et al., 2012; Long et al., 2022; Zhang et al., 2023). We have added the following paragraph to the introduction to describe the two studies most relevant our research. We have adapted the mention of arousal in the Discussion, adding context and references to the studies listed above. Reference numbers in excerpts from the manuscript: 70 = Zhang et al. (2023), 87 = Long et al. (2022), 100 = Bast et al. (2025), 101 = Lachat et al. (2012).

Lines 210-219, new paragraph in Introduction: "Other cross-sectional hyperscanning studies provide evidence that social closeness can shape the arousal that people experience while interacting, as well as INS. In a conversation study, Long et al. (87) recorded brain activity with fNIRS while participants engaged in supportive and conflictual conversations. The authors observed that romantic couples showed greater INS than control dyads when engaging in debate. INS was associated with dyads' arousal ratings, suggesting the dynamic range of arousal in conflict-related conversations with a romantic partner moderates INS. Further, Zhang et al. (70) compared INS during a competitive button-pressing task performed by older romantic couples and older strangers and found that romantic couples were more competitive and showed greater INS than strangers. These studies suggest that dyads who are socially closer explore more of the 'dynamic range' of arousal."

Lines 588-593, Discussion: "We interpret the increasing INS in RIFG~RTPJ as meaning that dyads who feel close to one another monitor and integrate each other's joint attention more than dyads who feel less socially close. Drawing on hyperscanning studies that find links between arousal and INS (70,87,100,101), we tentatively propose that the increasing INS for intergenerational dyads in RIFG~RIFG may reflect joint attention as well as heightened arousal or the facilitation of joint attention via heightened arousal."

***Reviewer #3:***
*Thank you very much for giving me the opportunity to read the manuscript "Longitudinal intergenerational hyperscanning indexes changes in social connection" submitted to PLOS Biology. The manuscript describes findings from a very impressive longitudinal and intergenerational fNIRS hyperscanning study, for which I would like to strongly commend the authors. I also found the theoretical background as well as methodological and analytical approaches to be of very high standard. I only have a few minor comments regarding the general theoretical framework, task setup, and data pre-processing and analysis that are outlined below.*

We thank Reviewer 3 for this very positive feedback.

*1) I would appreciate if the authors could explain in a bit more detail how exactly the drawing task unfolded and what its exact timing was. Am I right in assuming that the overall drawing task, including the one individual drawing phase and the two collaborative drawing phases, lasted for 5 minutes overall (as indicated on line 647)? If so, how long did the individual drawing phase last (on average) and how long did the collaborative drawing task last (on average)? Since the authors then compare INS during the individual versus the collaborative drawing phases, could they comment on the likely discrepancy in the durations and therefore the amount of available data for their statistical analyses?*

Participants drew once alone and twice together, each for 5 minutes. As such, participants drew for 15 minutes total per session. WTC estimates were computed for each 5-minute drawing block separately. This means that there are twice as many WTC values for drawing together as for drawing alone. As described on Line 635-637 of our discussion, our Bayesian estimation approach is suited for handling such differences in sample size.

To improve the clarity of our explanation of the drawing task (also noted as confusing by Reviewer 2; comment 6), we have altered the phrasing in the Procedure section:

Lines 752-756: "Next, participants drew three drawings with oil pastels, without talking. Participants were allotted 5 min per drawing for each of 3 drawings, totalling 15 min of drawing per session. Participants were instructed that they would complete three drawings, the first drawing alone and the

second and third together with their partner on a single piece of paper. They were instructed that they would have 5 min per drawing and could draw anything they liked."

Moreover, we verified that the WTC values for the first and second instances of drawing together did not differ systematically for any ROI pair (all sessions combined). Please see our response to comment 7 from Reviewer 2 on this matter.

*2) The authors also mention a "short collaborative activity" after the drawing. Was the data from this session also assessed?*

The collaborative behaviour data was not assessed in this manuscript. We have reported the puzzle collaboration score and scores reflecting the collaboration in the drawings in a newly published manuscript on cardiac synchrony in the same sample (Moffat et al., 2026). The other collaborative tasks have not been analysed. We have added this to the Methods and further detailed that the dataset will be made publicly available in the near future. Reference numbers in excerpts from the manuscript: 109 = Moffat & Cross (in preparation), 116 = Moffat et al. (2026).

Lines 762-764: "These include sorting pastels, a divergent thinking task, and a joint verbal fluency task, and a 54-piece jigsaw puzzle (to date only the puzzle collaboration is reported elsewhere (116))."

Lines 657-658: "The data presented here are part of a longitudinal hyperscanning dataset, the public sharing of which is forthcoming (109)."

We planned to disseminate our findings across multiple manuscripts in our preregistration, where we wrote that "we reserve the option to include other factors such as gender in our analyses and anticipate that this study might be reported across several papers".

*3) In line with my first question, I am wondering how exactly WTC was calculated. Was this done for each of the drawing phases independently (i.e., one WTC for individual drawing, and one WTC for collaborative drawing - or maybe even 2 WTCs for each collaborative drawing phase separately), or was WTC derived for the entire duration of 5 minutes? My question particularly pertains to the cone of influence (COI) that is present for each WTC analysis and that necessitates the removal of all INS values within the COI (as I could not find any indication of this being implemented). When assessing WTC in a frequency range of 5-50 period seconds, approximately 2 x 1.4 times the period length is lost within the COI at any given frequency, and thus approximately 140 seconds (~2.33 minutes) of data at 50 period seconds. This only leaves about 160 seconds of reliable task-related INS values in the middle of the task at this frequency - if one single WTC analysis is computed. For two, or potentially three, separate WTC analyses (i.e., one per phase), the frequency range would need to be decreased accordingly by removing lower frequency bands.*

Thank you for raising this concern. We computed WTC values separately for each 5-min drawing phase (5-min for alone, 5-min for together 1 and 5-min for together 2). As stated in the comment, this means that our WTC values reflect ~2.33 minutes per drawing phases, which we believe is well suited to the analyses we present. To make this more explicit in the manuscript, we have added adapted the Methods section describing how WTC was computed:

Lines 811-815 (grey highlights specific sections that were altered): "We computed INS values separately for each 5-min drawing task (alone, 1st drawing together, and 2nd drawing together), all possible ROI pairs (N = 10; homologous ROI pairs = LIFG~LIFG, RIFG~RIFG, LTPJ~LTPJ, RTPJ~RTPJ; non homologous ROI pairs = LIFG~RIFG, LIFG~LTPJ, LIFG~RTPJ, LTPJ~RTPJ, RIFG~LTPJ, RIFG~RTPJ) for each real dyad and pseudo dyad (Supplementary Methods Figure 8)."

*4) As far as I can see from Supplementary Figure 11* [Supplementary Methods Figure 5 in revised version]*, the authors had four ROIs with 2 x 6 and 2 x 5 standard-distance channels and one short-distance channel, respectively. Although I do appreciate that consistent optode placement is challenging due to the reasons the authors state in their manuscript, I am a bit surprised that they only used one single channel per ROI per participant for their final analyses. In doing so, potentially high-quality data from 4 to 5 channels is lost. This includes a loss of potentially meaningful variance, especially because changes in INS tend to be rather small.*

*Furthermore, the "best" channel may not always be the closest channel to the centre or the ROI (after channel exclusion due to bad signal quality). Would it not be better to use all available channels and to average (or statistically "nest") all the available data per ROI per participant?*

Our approach of selecting the channel with the combination of closest position to the centre of the ROI and best signal quality (based on De Felice et al., 2024) reduces variability between participants globally, as well as within participants from session to session. This is important because the assumption that sensors fall in identical positions on each participant's unique heads is inherently flawed (De Felice et al., 2024; Zimeo Morais et al., 2018). We overcome inter-participant differences in sensor position by ensuring that the signal we analyse is measured near a specific anatomical coordinate. Our approach maximises the session-to-session stability of the position from which we measure our signal within individuals, which is critical for test-retest reliability, which is assumed in longitudinal designs (Blasi et al., 2014; Collins-Jones et al., 2021).

Averaging all channels that meet the basic inclusion criteria (channel length, evidence of cardiac oscillation, and sufficient scalp coupling index) comes with numerous drawbacks. First, the number of channels included in the averaging analysis is highly variable between people, ROIs, and sessions (e.g., in a single participant, there may be 1 channel in one session, vs. the average of 5 channels in the next session, leading to analyses of signals from non-comparable brain area for a single participant). Second, every additional channel introduces 'worse' data, based on the evaluation criteria we used to rank them (closeness to centre of ROI and scalp coupling index). Moreover larger ROIs can obscure haemodynamic responses in focal areas (Shader et al., 2021), which is particularly unwarranted when coordinates can be used to select the optimal channels for a pre-defined brain region.

We remain convinced that our approach of selecting the 'best' channel following a stringent ranking procedure is the most conservative and reliable approach that we can responsibly apply. We have included more of this information in the Method section detailing channel selection. Reference numbers in excerpts from the manuscript:118 = Zimeo Morais et al. (2018), 119 = De Felice et al. (2024), 120 = Pollonini et al. (2014), 121 = Blasi et al. (2014), 122 = Collins-Jones et al. (2021), 123 = Shader et al. (2021).

> Lines 780-799: **Channel selection.** We aimed to record from the identical position on participants' heads at each session. However, due to the stretchy nature of neoprene caps and the inevitability of hair growth/haircuts, optode positions varied from session to session. This is also an acknowledged challenge in single-session studies, where a single optode arrangement will fall differently on individual participants' heads (118,119). To overcome inter-session and inter-subject differences in optode positioning, we prioritise proximity to the centre of each ROI (119). That is, for each participant at each session, we selected the best channel per ROI. First, we excluded channels without visible cardiac rhythms (~1 Hz), with a scalp-coupling index (SCI; 120) of <.7 for frequencies between 0.7-1.35 Hz, and with an interoptode distance <20 mm or >40 mm. Next, we ranked the remaining channels by distance from the centre of the ROI (closest to furthest) and selected the closest channel as the 'best channel' (Supplementary Methods Figure 8). The 'best channel' approach optimises the test-retest reliability of signals across sessions and between individuals–both assumed to be stable in longitudinal group-based analyses (121,122). In other words, selecting the 'best channel' according to our criteria ensures that all included signals stem from comparable brain regions, both in anatomical location and in number (e.g., we avoid comparing INS values computed from a single adequate channel to INS values reflecting the average of 5 channels, most of which lie further from the centre of the ROI). Spatial/anatomical consistency aside, consistency in ROI size is particularly relevant given evidence that ROI size impacts the amplitude of measured hemodynamic responses in task-based designs (123). See Supplementary Methods Table 2 for a description of the number of recordings and sessions excluded, as well as reasons for exclusion.

We compared the INS levels estimates for 'best channels' and 'averaged adequate channels' per ROI pair for each group and found the INS levels to be numerically very similar for both approaches. We describe this additional analysis in the discussion section on limitations. For interested readers, we have added a figure showing this similarity to the Supplementary Materials (Supplementary Methods Figure 3).

> Lines 637-641, Discussion: …and our selection of the 'best channel' per ROI helped ensure the quality of signals included in the analysis. Another approach would be to aggregate signals from all channels of adequate signal quality per ROI. In Supplementary Methods Figure 3, we show that this approach yields a comparable pattern of numerically similar point estimates. Interested readers can find the strengths and weaknesses of each approach in the Methods.

Page 8 of Supplementary Materials:

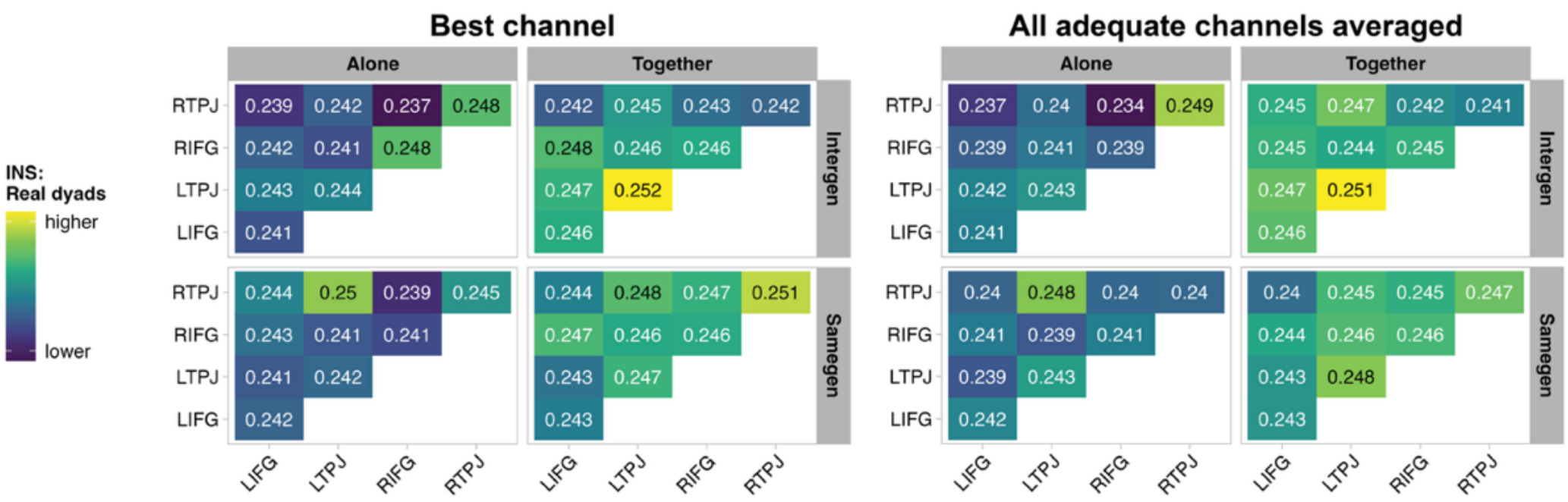


**Supplementary Methods Figure 3.** Comparison of INS point estimates using the best channel (left) and the averaged INS values for all channels in each ROI (right). The best channels were selected as described in the Methods. Adequate channels had visual evidence of heart beats, channel lengths between 20 and 40 mm, and scalp-coupling indices < .7. Visual inspection reveals INS levels to be numerically similar, and the overall pattern of levels to be similar. For example, in both RIFG~RTPJ Intergenerational Alone is the lowest value and LTPJ~LTPJ Intergenerational Together is the highest value.

*5) The authors indicate in Supplementary Table 0* [Supplementary Methods Table 2 in revised version] *that 69 dyadic sessions were impacted by "No short channels of adequate quality". Would this mean that for certain participants/dyads (at certain time points), no short channel regression could be carried out during pre-processing? If so, would the authors know whether this had an impact on the standard-distance channel data (especially in the frequency range of 5-10 period seconds where physiological artifacts are more common)?*

Recordings with 'no short channels of adequate quality' were not included in the analysis. All included channels were subject to the identical short-channel regression pre-processing. We did not want to mix two approaches to account for physiological artifacts. This is why we reported the number alongside our "missing data". The total number of channels included per ROI pair and session is presented in Supplementary Methods Figure 2. We have added a sentence to the Preprocessing section justifying the exclusion of short channels and referencing the supplementary table reporting missing/excluded data:

> Lines 804-805: "Recordings with no short channels with an SCI <.7 were excluded (Supplementary Methods Table 2)."

*6) In their multi-level regressions, did the authors consider the beta-like distribution of WTC values that are bound between 0 and 1?*

Following the Bayesian estimation approach, we used our prior knowledge about the plausibility of different values to set weakly informed priors (referred to as reasonably diffuse priors by some researchers) for models predicting INS levels (WTC values) and self-report scores. For INS levels, we used a normal distribution with a mean of 0 and standard deviation of 1. While this prior is wider than 0-1, it is markedly narrower and more aligned with plausible INS values than the default non-informative priors, that range from infinity to infinity (typical in both Bayesian and Frequentist modelling packages). We have added all priors in Supplementary methods Table 4 (see comment 2 from Reviewer 1), as well as the following text to our methods section. Reference numbers in excerpts from the manuscript: 89 = Kruschke & Liddell (2018), 90 = McElreath (2020).

> Lines 843-845: "We defined weakly informed priors for our models (reported in Supplementary Methods Table 4), thereby imposing a constrained distribution on our expected results (89,90)."

*7) In the introduction, it may be better to concentrate on fNIRS hyperscanning studies since this is the method also employed by the authors for their own analyses. Especially EEG studies tend to look at quite different frequency ranges and therefore potentially rather distinct neurobiological processes and time windows. It may also be useful to mention a bit more in detail what some of the benefits and constraints of WTC analysis are (also, and particularly, in the context of fNIRS hyperscanning research).*

Our research team spans fNIRS (Ryssa Moffat and Emily S. Cross) and EEG (Guillaume Dumas). We would argue that it is beneficial to the field of social (and relational) neuroscience to consider parallels between complementary techniques. This is particularly true in the context of the ongoing debate about the mechanisms of INS, which is pertinent across all measurement techniques. For this reason, we would prefer to keep the text describing how EEG INS reflecting sensorimotor integration makes the mutual prediction framework relevant for understanding dyadic collaboration.

Thank you for the encouragement to discuss the constraints of WTC in the context of fNIRS research. We have elaborated on this topic in the discussion section on limitations. Reference numbers in excerpts from the manuscript: 107 = Hakim et al. (2023), 108 = Chidichimo et al. (2025).

> Lines 641-645: "Finally, we used WTC (a linear measure) to quantify INS. WTC is the dominant method for computing INS in fNIRS signals (107), even though the interpersonal synchrony is widely believed to be non-linear. Approaches from information theory show promise for delivering insights into non-linear aspects of INS (108) and should be considered in future research."

*8) Finally, also in the introduction, and/or in the discussion, it would be good if a slightly more extended discussion of fNIRS hyperscanning within the realm of relational neuroscience could be implemented (De Felice et al., 2025, Neuroscience & Biobehavioral Reviews). This includes considerations of interpersonal neural synchrony spanning from real-time joint experiences to long-term social bonds, current interpretations of the role of interpersonal neural synchrony for human social interactions and relationships as well as important questions pertaining to causality and mechanistic explanations. That said, I would like to commend the authors for not using any causal language and for consistently discussing their findings in a very careful and thoughtful manner.*

Thank you for this suggestion. We are pleased to share that De Felice et al.'s perspective on relational neuroscience runs throughout our introduction and discussion. Reference numbers in excerpts from the manuscript: 40 = Hamilton (2021), 41 = Novembre & Iannetti (2021), 42 = Moreau & Dumas (2021), 43 = De Felice et al. (2025), 53 = Sun et al. (2021), 70 = Zhang et al. (2023), 74 = Adel et al. (2025), 75 = Bi et al. (2023), 76 = Hoehl et al. (2025), 80 = Bevilacqua et al. (2019), 86 = Djalovski et al. (2021), 87 = Long et al. (2022), 88 = Speer et al. (2024).

> Lines 94-95, Introduction: "The role of INS in social behaviour remains debated (40–43), with two main theoretical conceptualisations: common cognitive processing and mutual prediction."
>
> Lines 184-187, Introduction: "Taking a cross-sectional approach to understand social closeness and relationship development, some studies compare romantic couples, friends and strangers at single moments in time. Such studies suggest that the nature of a dyad's relationship influences INS during forms of interaction including joint motor action, conversation, and classroom learning (43, 53,70,75,80,86–88)."
>
> Lines 156-163, Introduction: Numerous reviews from different domains of social neuroscience (e.g., developmental, clinical, communication, etc.) recognise the lack of longitudinal insights into INS and call for longitudinal hyperscanning studies to be conducted (e.g., 43,74–79). Concretely, multi-session longitudinal designs can fill in the blanks in our understanding of the dynamics of forming and maintaining social connections–a field recently coined 'relational neuroscience' (43).
>
> Lines 562-564, Discussion: This has potential implications for the mutual prediction framework, as well as the field of relational neuroscience, that merit further investigation.

*I hope that my comments are useful and that they will help further improving an already very impressive and theoretically and methodologically strong report.*

**References:**


Adel, L., Moses, L., Irvine, E., Greenway, K. T., Dumas, G., & Lifshitz, M. (2025). A systematic review of hyperscanning in clinical encounters. *Neuroscience & Biobehavioral Reviews*, *176*, 106248. https://doi.org/10.1016/j.neubiorev.2025.106248

Bast, N., Polzer, L., Raji, N., Schnettler, L., Kleber, S., Lemler, C., Kitzerow-Cleven, J., Kim, Z., Schaer, M., & Freitag, C. M. (2025). Early intervention increases reactive joint attention in autistic preschoolers with arousal regulation as mediator. *European Child & Adolescent Psychiatry*. https://doi.org/10.1007/s00787-025-02738-1

Bevilacqua, D., Davidesco, I., Wan, L., Chaloner, K., Rowland, J., Ding, M., Poeppel, D., & Dikker, S. (2019). Brain-to-brain synchrony and learning outcomes vary by student–teacher dynamics: Evidence from a real-world classroom electroencephalography study. *Journal of Cognitive Neuroscience*, *31*(3), 401–411. https://doi.org/10.1162/jocn_a_01274

Bi, X., Cui, H., & Ma, Y. (2023). Hyperscanning Studies on Interbrain Synchrony and Child Development: A Narrative Review. *Neuroscience*, *530*, 38–45. https://doi.org/10.1016/j.neuroscience.2023.08.035

Bilek, E., Zeidman, P., Kirsch, P., Tost, H., Meyer-Lindenberg, A., & Friston, K. (2022). Directed coupling in multi-brain networks underlies generalized synchrony during social exchange. *NeuroImage*, *252*, 119038. https://doi.org/10.1016/j.neuroimage.2022.119038

Blasi, A., Lloyd-Fox, S., Johnson, Mark. H., & Elwell, C. (2014). Test–retest reliability of functional near infrared spectroscopy in infants. *Neurophotonics*, *1*(2), 025005. https://doi.org/10.1117/1.NPh.1.2.025005

Burgess, A. P. (2013). On the interpretation of synchronization in EEG hyperscanning studies: A cautionary note. *Frontiers in Human Neuroscience*, *7*. https://doi.org/10.3389/fnhum.2013.00881

Chidichimo, E., Luppi, A. I., Mediano, P. A. M., Leong, V., Dumas, G., Canales-Johnson, A., & Bethlehem, R. A. I. (2025). Towards an informational account of interpersonal coordination. *Nature Reviews Neuroscience*. https://doi.org/10.1038/s41583-025-00989-0

Collins-Jones, L. H., Cooper, R. J., Bulgarelli, C., Blasi, A., Katus, L., McCann, S., Mason, L., Mbye, E., Touray, E., Ceesay, M., Moore, S. E., Lloyd-Fox, S., & Elwell, C. E. (2021). Longitudinal infant fNIRS channel-space analyses are robust to variability parameters at the group-level: An image reconstruction investigation. *NeuroImage*, *237*, 118068. https://doi.org/10.1016/j.neuroimage.2021.118068

Cumming, G. (2014). The New Statistics: Why and How. *Psychological Science*, *25*(1), 7–29. https://doi.org/10.1177/0956797613504966

De Felice, S., Chand, T., Croy, I., Engert, V., Goldstein, P., Holroyd, C. B., Kirsch, P., Krach, S., Ma, Y., Scheele, D., Schurz, M., Schweinberger, S. R., Hoehl, S., & Vrticka, P. (2025). Relational neuroscience: Insights from hyperscanning research. *Neuroscience & Biobehavioral Reviews*, *169*, 105979. https://doi.org/10.1016/j.neubiorev.2024.105979

De Felice, S., Hakim, U., Gunasekara, N., Pinti, P., Tachtsidis, I., & Hamilton, A. (2024). Having a chat and then watching a movie: How social interaction synchronises our brains during co-watching. *Oxford Open Neuroscience*, *3*, kvae006. https://doi.org/10.1093/oons/kvae006

Dirnagl, U. (2020). Preregistration of exploratory research: Learning from the golden age of discovery. *PLOS Biology*, *18*(3), e3000690. https://doi.org/10.1371/journal.pbio.3000690

Djalovski, A., Dumas, G., Kinreich, S., & Feldman, R. (2021). Human attachments shape interbrain synchrony toward efficient performance of social goals. *NeuroImage*, *226*, 117600. https://doi.org/10.1016/j.neuroimage.2020.117600

Frank, M. C., Braginsky, M., Cachia, J., Coles, N. A., Hardwicke, T. E., Hawkins, R. D., Mathur, M. B., & Williams, R. (2025). *Experimentology: An open science approach to experimental psychology methods*. The MIT Press.

Hakim, U., De Felice, S., Pinti, P., Zhang, X., Noah, J., Ono, Y., Burgess, P. W., Hamilton, A., Hirsch, J., & Tachtsidis, I. (2023). Quantification of inter-brain coupling: A review of current methods used in haemodynamic and electrophysiological hyperscanning studies. *NeuroImage*, *280*, 120354. https://doi.org/10.1016/j.neuroimage.2023.120354

Hamilton, A. F. de C. (2021). Hyperscanning: Beyond the hype. *Neuron*, *109*(3), 404–407. https://doi.org/10.1016/j.neuron.2020.11.008

Hoehl, S., Bánki, A., Brzozowska, A., Carollo, A., Kostorz, K., Nguyen, T., Pletti, C., Reisner, S., Schäfer, V. T., Schaetz, C., & Tünte, M. R. (2025). A developmental framework of interpersonal neural synchrony. *Developmental Review*, *78*, 101234. https://doi.org/10.1016/j.dr.2025.101234

Kingsbury, L., Huang, S., Wang, J., Gu, K., Golshani, P., Wu, Y. E., & Hong, W. (2019). Correlated neural activity and encoding of behavior across brains of socially interacting animals. *Cell*, *178*(2), 429-446.e16. https://doi.org/10.1016/j.cell.2019.05.022

Kruschke, J. K., & Liddell, T. M. (2018). The Bayesian New Statistics: Hypothesis testing, estimation, meta-analysis, and power analysis from a Bayesian perspective. *Psychonomic Bulletin & Review*, *25*(1), 178–206. https://doi.org/10.3758/s13423-016-1221-4

Lachat, F., Hugueville, L., Lemaréchal, J.-D., Conty, L., & George, N. (2012). Oscillatory brain correlates of live joint attention: A dual-EEG study. *Frontiers in Human Neuroscience*, *6*. https://doi.org/10.3389/fnhum.2012.00156

Lakens, D. (2022). Sample Size Justification. *Collabra: Psychology*, *8*(1), 33267. https://doi.org/10.1525/collabra.33267

Long, Y., Chen, C., Wu, K., Zhou, S., Zhou, F., Zheng, L., Zhao, H., Zhai, Y., & Lu, C. (2022). Interpersonal conflict increases interpersonal neural synchronization in romantic couples. *Cerebral Cortex*, *32*(15), 3254–3268. https://doi.org/10.1093/cercor/bhab413

McElreath, R. (2020). *Statistical Rethinking; A Bayesian Course with Examples in R and Stan* (2nd Edition). Chapman and Hall/CRC.

Moffat, R., & Cross, E. S. (in preparation). *A longitudinal fNIRS hypercanning data set of same-generation and intergenerational relationship development and collaboration* [Dataset]. Social Brain Sciences Lab, ETH Zurich.

Moffat, R., Naudszus, L. A., & Cross, E. S. (2026). Cardiac synchrony during collaborative drawing: A longitudinal comparison of same generation and intergenerational dyads. *Annals of the New York Academy of Sciences*, *1558*(1), e70272. https://doi.org/10.1111/nyas.70272

Moreau, Q., & Dumas, G. (2021). Beyond correlation versus causation: Multibrain neuroscience needs explanation. *Trends in Cognitive Sciences*, *25*(7), 542–543. https://doi.org/10.1016/j.tics.2021.02.011

Novembre, G., & Iannetti, G. D. (2021). Hyperscanning alone cannot prove causality. Multibrain stimulation can. *Trends in Cognitive Sciences*, *25*(2), 96–99. https://doi.org/10.1016/j.tics.2020.11.003

Pollonini, L., Olds, C., Abaya, H., Bortfeld, H., Beauchamp, M. S., & Oghalai, J. S. (2014). Auditory cortex activation to natural speech and simulated cochlear implant speech measured with functional near-infrared spectroscopy. *Hearing Research*, *309*, 84–93. https://doi.org/10.1016/j.heares.2013.11.007

Shader, M. J., Luke, R., Gouailhardou, N., & Mckay, C. M. (2021). The use of broad vs restricted regions of interest in functional near-infrared spectroscopy for measuring cortical activation to auditory-only and visual-only speech. *Hearing Research*, *406*, 108256. https://doi.org/10.1016/j.heares.2021.108256

Speer, S. P. H., Mwilambwe-Tshilobo, L., Tsoi, L., Burns, S. M., Falk, E. B., & Tamir, D. I. (2024). Hyperscanning shows friends explore and strangers converge in conversation. *Nature Communications*, *15*(1), 7781. https://doi.org/10.1038/s41467-024-51990-7

Sun, B., Xiao, W., Lin, S., Shao, Y., Li, W., & Zhang, W. (2021). Cooperation with partners of differing social experience: An fNIRS-based hyperscanning study. *Brain and Cognition*, *154*, 105803. https://doi.org/10.1016/j.bandc.2021.105803

Wolpert, D. M., Doya, K., & Kawato, M. (2003). A unifying computational framework for motor control and social interaction. *Philosophical Transactions of the Royal Society of London. Series B: Biological Sciences*, *358*(1431), 593–602. https://doi.org/10.1098/rstb.2002.1238

Zhang, Q., Liu, Z., Qian, H., Hu, Y., & Gao, X. (2023). Interpersonal competition in elderly couples: A functional near-infrared spectroscopy hyperscanning study. *Brain Sciences*, *13*(4), 600. https://doi.org/10.3390/brainsci13040600

Zimeo Morais, G. A., Balardin, J. B., & Sato, J. R. (2018). FNIRS Optodes' Location Decider (fOLD): A toolbox for probe arrangement guided by brain regions-of-interest. *Scientific Reports*, *8*, 1–11. https://doi.org/10.1038/s41598-018-21716-z
