# Supplementary material for: Social interactions between people of same and different generations shape longitudinal changes in interpersonal neural synchrony, loneliness, and social connection
Source: PLoS Biol. 2026 Aug 20;24(8):e3003899. doi: 10.1371/journal.pbio.3003899 (PMC13492753; doi:10.1371/journal.pbio.3003899)
Supplement: Supplementary file 3 [file pbio.3003899.s005.pdf]

Dear Dr. Schnell,

We are pleased to submit our revised manuscript entitled "Longitudinal intergenerational hyperscanning indexes changes in social connection" for your consideration.

In response to Reviewer 1's further constructive comments, in the revised manuscript we now discuss in more detail the deviation between the preregistered hypothesis and findings in the main text and emphasize the effect sizes observed for loneliness and social connection in the discussion.

Below, we provide detailed responses to each of Reviewer 1's comments. References to the updated (tracked) manuscript are made by line number (e.g., Line XX). Reviewer 1's comments are provided in *blue italics*. Our responses and excerpts from the original manuscript are in black. New or altered text excerpts are presented in red. We use grey highlighting to show where we have made changes to the existing text.

We appreciate the time and thoughtful feedback provided by all three reviewers throughout the peer review process and hope that the revised manuscript will now be suitable for publication. Should we have missed a clear way in your eyes to address the remaining comments, we would welcome the opportunity for a follow-up zoom call.

Thank you very much for considering our work.

Yours sincerely,

Ryssa Moffat, Emily S. Cross and Guillaume Dumas

Reviewer #1:
Thank you very much for the detailed and thoughtful responses to the previous round of reviews. The revision has clearly improved the transparency of the manuscript, particularly with respect to the statistical approach and the exploratory nature of the analyses. I appreciate the authors' efforts to clarify their methodological choices and to engage with the relevant statistical literature.

I also find it entirely acceptable that the authors frame their work as a fully exploratory, preregistered analysis. However, I remain concerned about the handling of the preregistered hypotheses and their relationship to the reported findings. In the preregistration, the following hypothesis is explicitly stated:"We hypothesise that interpersonal synchrony during collaboration, both behavioural and neural, will increase within dyads over repeated interactions. Further, we expect the increase to be greater in intergenerational dyads than same-generation dyads, as greater cognitive load might be incurred to overcome generational differences (Dikker et al., 2022)." This hypothesis is clearly directional and mechanistically motivated, and it is not consistent with the reported findings (i.e., decreasing synchrony in intergenerational dyads). Thus, while the authors now characterize the study as exploratory, it is evident that there were prior expectations and theoretical considerations that guided the work. In their response, the authors state that Supplementary Table 1 "summarizes the alignment of our preregistered predictions with our findings." However, such a summary is not, in fact, provided in a meaningful way. Most importantly, the fundamental discrepancy between the preregistered hypothesis and the observed results is not explicitly acknowledged or discussed anywhere in the revised manuscript. I strongly encourage the authors to address this point directly in the main text. A transparent discussion of why the preregistered expectation was not supported, and how this informs the theoretical framework, would substantially strengthen the manuscript.

Relatedly, I thank the authors for their extensive references to the statistical literature and their clarification of the Bayesian estimation approach. However, I would appreciate further clarification on how this estimation-focused strategy aligns with the presence of clearly formulated (albeit exploratory) hypotheses in the preregistration. Specifically:
* *If the goal is not to evaluate hypotheses, how should readers interpret the discrepancy between the preregistered expectations and the posterior estimates?*
* *How do the authors reconcile the existence of directional hypotheses with an analytical framework that explicitly avoids hypothesis testing?*

Clarifying this conceptual link would improve the coherence between the study's theoretical motivation and its statistical implementation.

We thank Reviewer 1 for the opportunity to further clarify the difference between our preregistered hypothesis and the observed data in the main text, as well as the statistical approach.

We included the weak hypotheses in the preregistration to guide our model building (i.e., to justify the inclusion of parameters). We consider these hypotheses weak, given the novelty of the study (both the longitudinal and the intergeneration aspects) and the exceedingly small amount of literature available to support them. Having considered Reviewer 1's comments, we understand that the phrasing "we hypothesise" relays a strong, mechanistic hypothesis suited for a confirmatory analysis. To provide readers more nuanced insight on this matter, we now include a clear description of the preregistered hypothesis and the sparse literature it was based on to the beginning of the discussion paragraph about changes in INS across sessions. One potential mechanism for the difference in predicted and observed patterns is that the research by Dikker and colleagues was largely based on verbal communication, whereas our paradigm relied more on non-verbal collaboration. This difference in experimental paradigms may contribute to the difference between the observed. The grey highlighted text is the new text.

> Lines 563-577: "In our preregistration, we hypothesised that INS would increase across sessions for both groups, with a steeper rate of change for intergenerational dyads (see Section 5.1 for preregistration details). We included this (weak) hypothesis to document our initial thoughts collected from the limited intergenerational and/or longitudinal hypercanning research. Specifically, the prediction of increasing INS with a steeper slope for intergenerational dyads was based on Dikker and colleagues' theoretical and simulated predictions about age-related differences in INS during verbal communication (72,97). In contrast, our real-world data were collected while participants were specifically instructed to refrain from verbal communication during collaborative drawing, potentially leading to the difference between predicted and observed results. We observed that while dyads drew together, ROI pairs that include right TPJ (RTPJ~RTPJ, LIFG~RTPJ, and RIFG~RTPJ) showed decreasing INS across sessions among intergenerational dyads but increasing INS among same generation dyads. This has potential implications for the mutual prediction framework, as well as the field of relational neuroscience, that merit further empirical investigation. For example, this may suggest that, in the context of collaborative drawing, intergenerational dyads' monitoring and integration of their partner's levels of joint attention may decrease with increasing familiarity, whereas same generation dyads may monitor each other's levels of joint attention more closely as familiarity increases…"

With respect to the statistical approach, we do evaluate hypotheses and the Bayesian estimation approach is suited to the task of evaluating such hypotheses, as described by Kruschke and Liddell (2018). As per Kruschke and Liddell, hypothesis testing is not a necessary requirement for hypothesis evaluation. Specifically, in Figure 1 of their paper, Kruschke and Liddell visualise four ways to evaluate hypotheses using a 2x2 matrix (frequentist and Bayesian x hypothesis testing and estimation with uncertainty). We apply the Bayesian estimation approach in the bottom right quadrant of Figure 1. As detailed by Kruschke and Liddel and in our manuscript, the parameters can be interpreted according to the point estimate reflecting the centre of the posterior distribution and the 95 % HPD reflecting the breadth of the most credible values contained by the distribution. These values, like frequentist point estimates and confidence intervals, describe the sign, size and width of an estimated effect, with the added knowledge of the shape of the distribution (e.g., normal).

Kruschke, J. K., & Liddell, T. M. (2018). The Bayesian New Statistics: Hypothesis testing, estimation, meta-analysis, and power analysis from a Bayesian perspective. *Psychonomic Bulletin & Review*, *25*(1), 178–206. https://doi.org/10.3758/s13423-016-1221-4

Finally, I appreciate the authors' efforts to better contextualize the magnitude of their findings. For example, the revised Results now state: "Across sessions, feelings of loneliness showed small reductions for both groups (~1% per session), with no observed difference in rate of reduction between groups." This is a welcome improvement. However, this more nuanced interpretation should also be consistently reflected in the Discussion. Currently, the manuscript states: "Individuals' levels of loneliness and social closeness decreased and increased, respectively, across sessions, for both generational constellations. These findings match the target outcomes of the community programs proposed by policy makers, researchers, and health practitioners […] and indicate

that social connections were indeed formed." I encourage the authors to align the Discussion more closely with the quantified effect sizes and their practical significance.

We appreciate the chance to elaborate on this discussion component. We have added the following (grey highlighted text) to the paragraph in question:

> Lines 500-507: "Individuals' levels of loneliness and social closeness decreased and increased, respectively, across sessions, for both generational constellations. These findings match the target outcomes of the community programs proposed by policy makers, researchers, and health practitioners (1,5,21,22) and indicate that social connections were indeed formed. Practically, our results reveal a 1% reduction in loneliness scores per session and a 4% increase in social closeness per session. These incremental changes highlight the potential of repeated encounters. Further research with more than 6 sessions would be useful for ascertaining if and at which point these changes in loneliness and social closeness may plateau."
