# Supplementary material for: Social interactions between people of same and different generations shape longitudinal changes in interpersonal neural synchrony, loneliness, and social connection
Source: PLoS Biol. 2026 Aug 20;24(8):e3003899. doi: 10.1371/journal.pbio.3003899 (PMC13492753; doi:10.1371/journal.pbio.3003899)
Supplement: Peer review history

# Editor report: Decision Letter 0

**DOI:** 10.1371/journal.pbio.3003899.r001

**Authors:** Christian Schnell, PhD

2 Feb 2026

Dear Ryssa,

Thank you for submitting your manuscript entitled "Longitudinal intergenerational hyperscanning indexes changes in social connection" for consideration as a Short Reports by PLOS Biology.

Your manuscript has now been evaluated by the PLOS Biology editorial staff as well as by an academic editor with relevant expertise and I am writing to let you know that we would like to send your submission out for external peer review.

However, before we can send your manuscript to reviewers, we need you to complete your submission by providing the metadata that is required for full assessment. To this end, please login to Editorial Manager where you will find the paper in the 'Submissions Needing Revisions' folder on your homepage. Please click 'Revise Submission' from the Action Links and complete all additional questions in the submission questionnaire.

Once your full submission is complete, your paper will undergo a series of checks in preparation for peer review. After your manuscript has passed the checks it will be sent out for review. To provide the metadata for your submission, please Login to Editorial Manager (https://www.editorialmanager.com/pbiology) within two working days, i.e. by Feb 04 2026 11:59PM.

If your manuscript has been previously peer-reviewed at another journal, PLOS Biology is willing to work with those reviews in order to avoid re-starting the process. Submission of the previous reviews is entirely optional and our ability to use them effectively will depend on the willingness of the previous journal to confirm the content of the reports and share the reviewer identities. Please note that we reserve the right to invite additional reviewers if we consider that additional/independent reviewers are needed, although we aim to avoid this as far as possible. In our experience, working with previous reviews does save time.

If you would like us to consider previous reviewer reports, please edit your cover letter to let us know and include the name of the journal where the work was previously considered and the manuscript ID it was given. In addition, please upload a response to the reviews as a 'Prior Peer Review' file type, which should include the reports in full and a point-by-point reply detailing how you have or plan to address the reviewers' concerns.

During the process of completing your manuscript submission, you will be invited to opt-in to posting your pre-review manuscript as a bioRxiv preprint. Visit http://journals.plos.org/plosbiology/s/preprints for full details. If you consent to posting your current manuscript as a preprint, please upload a single Preprint PDF.

Feel free to email us at plosbiology@plos.org if you have any queries relating to your submission.

Kind regards,

Christian

Christian Schnell, PhD

Senior Editor

PLOS Biology

cschnell@plos.org

# Peer review: Decision Letter 1

**DOI:** 10.1371/journal.pbio.3003899.r002

**Authors:** Christian Schnell, PhD

10 Mar 2026

Dear Ryssa,

Thank you for your patience while your manuscript "Longitudinal intergenerational hyperscanning indexes changes in social connection" was peer-reviewed at PLOS Biology. It has now been evaluated by the PLOS Biology editors, an Academic Editor with relevant expertise, and by several independent reviewers.

In light of the reviews, which you will find at the end of this email, we would like to invite you to revise the work to thoroughly address the reviewers' reports.

As you will see below, the reviewers are impressed by the scope of the study. However, they also identified critical issues regarding transparency and statistical interpretation that must be addressed. In particular, the concerns about transparency regarding preregistration, the statistical rigor for the null results, lack of methodological clarity and the theoretical framework would need to be fully addressed.

Given the extent of revision needed, we cannot make a decision about publication until we have seen the revised manuscript and your response to the reviewers' comments. Your revised manuscript is likely to be sent for further evaluation by all or a subset of the reviewers.

In addition to these revisions, you will need to complete some formatting changes, which you will receive in a follow up email. A member of our team will be in touch with a set of requests shortly.

We expect to receive your revised manuscript within 3 months. Please email us (plosbiology@plos.org) if you have any questions or concerns, or would like to request an extension.

At this stage, your manuscript remains formally under active consideration at our journal; please notify us by email if you do not intend to submit a revision so that we may withdraw it.

**IMPORTANT - SUBMITTING YOUR REVISION**

Your revisions should address the specific points made by each reviewer. Please submit the following files along with your revised manuscript:

1. A 'Response to Reviewers' file - this should detail your responses to the editorial requests, present a point-by-point response to all of the reviewers' comments, and indicate the changes made to the manuscript.

*NOTE: In your point-by-point response to the reviewers, please provide the full context of each review. Do not selectively quote paragraphs or sentences to reply to. The entire set of reviewer comments should be present in full and each specific point should be responded to individually, point by point.

You should also cite any additional relevant literature that has been published since the original submission and mention any additional citations in your response.

2. In addition to a clean copy of the manuscript, please also upload a 'track-changes' version of your manuscript that specifies the edits made. This should be uploaded as a "Revised Article with Changes Highlighted" file type.

*Re-submission Checklist*

When you are ready to resubmit your revised manuscript, please refer to this re-submission checklist: https://plos.io/Biology_Checklist

To submit a revised version of your manuscript, please go to https://www.editorialmanager.com/pbiology/ and log in as an Author. Click the link labelled 'Submissions Needing Revision' where you will find your submission record.

Please make sure to read the following important policies and guidelines while preparing your revision:

*Published Peer Review*

Please note while forming your response, if your article is accepted, you may have the opportunity to make the peer review history publicly available. The record will include editor decision letters (with reviews) and your responses to reviewer comments. If eligible, we will contact you to opt in or out. Please see here for more details:

https://blogs.plos.org/plos/2019/05/plos-journals-now-open-for-published-peer-review/

*PLOS Data Policy*

Please note that as a condition of publication PLOS' data policy (http://journals.plos.org/plosbiology/s/data-availability) requires that you make available all data used to draw the conclusions arrived at in your manuscript. If you have not already done so, you must include any data used in your manuscript either in appropriate repositories, within the body of the manuscript, or as supporting information (N.B. this includes any numerical values that were used to generate graphs, histograms etc.). For an example see here: http://www.plosbiology.org/article/info%3Adoi%2F10.1371%2Fjournal.pbio.1001908#s5

*Blot and Gel Data Policy*

We require the original, uncropped and minimally adjusted images supporting all blot and gel results reported in an article's figures or Supporting Information files. We will require these files before a manuscript can be accepted so please prepare them now, if you have not already uploaded them. Please carefully read our guidelines for how to prepare and upload this data: https://journals.plos.org/plosbiology/s/figures#loc-blot-and-gel-reporting-requirements

*Protocols deposition*

To enhance the reproducibility of your results, we recommend that if applicable you deposit your laboratory protocols in protocols.io, where a protocol can be assigned its own identifier (DOI) such that it can be cited independently in the future. Additionally, PLOS ONE offers an option for publishing peer-reviewed Lab Protocol articles, which describe protocols hosted on protocols.io. Read more information on sharing protocols at https://plos.org/protocols?utm_medium=editorial-email&utm_source=authorletters&utm_campaign=protocols

Thank you again for your submission to our journal. We hope that our editorial process has been constructive thus far, and we welcome your feedback at any time. Please don't hesitate to contact us if you have any questions or comments.

Sincerely,

Christian

Christian Schnell, PhD

Senior Editor

PLOS Biology

cschnell@plos.org

------------------------------------

REVIEWS:

Reviewer #1: This manuscript addresses an important and timely topic and makes a potentially valuable contribution to social neuroscience. The longitudinal hyperscanning design is a clear strength, as multi-session interbrain studies remain rare. The inclusion of older adults further enhances the relevance of the work.

However, several conceptual and analytical issues substantially limit the interpretability of the findings. In its current form, the manuscript reads largely exploratory, despite being preregistered.

1. A major concern is the limited alignment between the preregistered hypotheses and the reported analyses. The preregistration explicitly stated that interpersonal synchrony (INS) would increase across repeated interactions, particularly in intergenerational dyads. Instead, INS decreased across sessions in intergenerational dyads. This reversal of the primary hypothesis is not adequately addressed or theoretically reconciled. In addition, there appear to be further discrepancies between the preregistration and the manuscript. For example, the preregistration specifies an age cutoff of 70+ for older adults, whereas the manuscript reports 69+. Such deviations may be minor, but they should be explicitly documented and justified to maintain transparency and credibility.

Moreover, no specific hypotheses were formulated for ROI-level effects. As a result, the numerous ROI-specific findings are de facto exploratory. This should be clearly acknowledged, and the confirmatory versus exploratory components of the manuscript should be more transparently separated.

2. The manuscript frequently distinguishes between "substantial effects" and "no effect," but this binary framing obscures effect magnitude and evidential strength. Statements such as "intergenerational dyads reported feeling substantially less lonely" lack contextualization. Substantial in what sense? How meaningful is it in practical terms? Crucially, Bayes Factors are not reported. If conclusions such as "INS is largely comparable between groups" are drawn, the strength of evidence for the null hypothesis should be quantified. Without this, claims of comparability remain ambiguous.

In addition, the rationale for the sample size is not clearly justified. In a Bayesian framework, evidential strength depends in part on prior assumptions; without a transparent discussion of priors and design considerations, it remains difficult to evaluate how informative the data truly are for both null and alternative effects.

3. The analytical scope is extremely broad: multiple ROI pairs, two tasks, two signal types, longitudinal slopes, interaction models, and several self-report measures (+ non-reported measures like empathy). This creates a highly complex analytical space with a substantial risk of overinterpretation. This concern is amplified by the fact that the preregistered behavioral hypothesis (movement synchrony) is not reported. The omission of the hypothesized behavioral outcome further shifts the manuscript toward exploratory neural analyses. A clearer prioritization of analyses and stronger constraints on interpretation are needed.

4. The manuscript does not sufficiently characterize baseline differences between groups. If intergenerational and same-generation dyads differed in loneliness or social closeness at baseline, these differences may partly drive longitudinal effects. Additionally, the drawing sessions are framed as an intervention to increase social connection, yet it remains unclear whether participants were meaningfully lonely to begin with. Without clearer baseline characterization, the interpretation of change effects remains limited.

5. The abstract claims that the findings "can inform program design choices," yet the manuscript does not clearly articulate which design elements should be altered based on the results. Given the mixed and partially contradictory INS trajectories, the translational implications appear overstated.

6. There appears to be inconsistency between main text and supplementary results regarding loneliness effects, which requires clarification. Main text: "We observed no substantial main, task, or group effect on the relationship between INS and summed loneliness scores." Supplement: "We observed a substantial main effect, whereby greater loneliness predicted lower INS in LIFG~RIFG, as well as a matching substantial group effect for same generation dyads, and a matching substantial task effect for drawing alone."

7. It is also unclear whether prior acquaintance between dyad members or gender composition were statistically controlled. Given unequal gender distributions, this is a non-trivial issue.

Reviewer #2: The manuscript reports rare longitudinal and intergenerational study using fNIRS hyperscanning. An impressive dataset from 61 dyads across 6 time points was collected, including young adult dyads and intergenerational younger-older adult dyads creating artworks together or alone. I think this is a very timely and interesting study with sound methods and exemplary open science practices. I should note that I am not an expert on Bayesian statistics, so I won't comment on the statistical analyses.

While the manuscript is generally well-written, I do have some requests for clarification and recommendations.

Introduction:

The empirical background is nicely sketched out, but I was missing a guiding theoretical framework. Of course, in the hyperscanning literature there are many, so I'm not suggesting an exhaustive review. Rather, I'm interested in the authors' notion of why and how they would expect synchrony to emerge in their task and what could be the underlying psychological mechanisms, if any. I noted that predictability of behavior was mentioned in the introduction, which is of course discussed a lot in the INS literature, but I wasn't sure whether the authors suggest predictability/prediction as a core mechanism underlying INS. Please elaborate on your theoretical model. This will also help to contextualize and interpret the results in the discussion section which in part seems a bit speculative.

Methods/ Results:

The excluded dyads/ sessions are listed in the supplementary material, but I was wondering whether there were any differences in compliance/ dropout across the groups (same generation/ intergenerational).

I suggest to briefly mention the instruments for the self-reports in the results section. I'm not sure all readers are familiar with the IOS, so the abbreviation has to be properly introduced.

How exactly were the pseudo dyads created and how many?

Did you assess any more sociodemographic data that would allow to contextualize the results? E.g. socioeconomic status, education, cultural background…

The description of the procedures is not entirely clear: Did the participants spend 5 minutes per drawing or five minutes in total drawing together and alone? I assume the former, but I wasn't sure based on the description.

The sequence of conditions was fixed with participants always drawing alone first. This is a bit of a concern for the condition effect. Could increased INS when drawing together vs. alone be (in part) due to increasing INS levels over the course of each session? Have you looked at differences between the second and third drawing?

Have there been any diversions from the pre-registration? If yes, these should be listed somewhere.

Discussion:

As mentioned above, the manuscript would benefit from a clearer theoretical framework. Predictions are mentioned again in the discussion, but it is not clear how mutual predictability/ predictions relate to INS in the authors' view. Joint attention and arousal are also discussed, but given the complexities of functions associated with the involved brain areas that seems a bit speculative again. Also, INS in these regions does not equate to heightened activity, just more interpersonal alignment.

Reviewer #3: Thank you very much for giving me the opportunity to read the manuscript "Longitudinal intergenerational hyperscanning indexes changes in social connection" submitted to PLOS Biology. The manuscript describes findings from a very impressive longitudinal and intergenerational fNIRS hyperscanning study, for which I would like to strongly commend the authors. I also found the theoretical background as well as methodological and analytical approaches to be of very high standard. I only have a few minor comments regarding the general theoretical framework, task setup, and data pre-processing and analysis that are outlined below.

1) I would appreciate if the authors could explain in a bit more detail how exactly the drawing task unfolded and what its exact timing was. Am I right in assuming that the overall drawing task, including the one individual drawing phase and the two collaborative drawing phases, lasted for 5 minutes overall (as indicated on line 647)? If so, how long did the individual drawing phase last (on average) and how long did the collaborative drawing task last (on average)? Since the authors then compare INS during the individual versus the collaborative drawing phases, could they comment on the likely discrepancy in the durations and therefore the amount of available data for their statistical analyses?

2) The authors also mention a "short collaborative activity" after the drawing. Was the data from this session also assessed?

3) In line with my first question, I am wondering how exactly WTC was calculated. Was this done for each of the drawing phases independently (i.e., one WTC for individual drawing, and one WTC for collaborative drawing - or maybe even 2 WTCs for each collaborative drawing phase separately), or was WTC derived for the entire duration of 5 minutes? My question particularly pertains to the cone of influence (COI) that is present for each WTC analysis and that necessitates the removal of all INS values within the COI (as I could not find any indication of this being implemented). When assessing WTC in a frequency range of 5-50 period seconds, approximately 2 x 1.4 times the period length is lost within the COI at any given frequency, and thus approximately 140 seconds (~2.33 minutes) of data at 50 period seconds. This only leaves about 160 seconds of reliable task-related INS values in the middle of the task at this frequency - if one single WTC analysis is computed. For two, or potentially three, separate WTC analyses (i.e., one per phase), the frequency range would need to be decreased accordingly by removing lower frequency bands.

4) As far as I can see from Supplementary Figure 11, the authors had four ROIs with 2 x 6 and 2 x 5 standard-distance channels and one short-distance channel, respectively. Although I do appreciate that consistent optode placement is challenging due to the reasons the authors state in their manuscript, I am a bit surprised that they only used one single channel per ROI per participant for their final analyses. In doing so, potentially high-quality data from 4 to 5 channels is lost. This includes a loss of potentially meaningful variance, especially because changes in INS tend to be rather small. Furthermore, the "best" channel may not always be the closest channel to the centre or the ROI (after channel exclusion due to bad signal quality). Would it not be better to use all available channels and to average (or statistically "nest") all the available data per ROI per participant?

5) The authors indicate in Supplementary Table 0 that 69 dyadic sessions were impacted by "No short channels of adequate quality". Would this mean that for certain participants / dyads (at certain time points), no short channel regression could be carried out during pre-processing? If so, would the authors know whether this had an impact on the standard-distance channel data (especially in the frequency range of 5-10 period seconds where physiological artifacts are more common)?

6) In their multi-level regressions, did the authors consider the beta-like distribution of WTC values that are bound between 0 and 1?

7) In the introduction, it may be better to concentrate on fNIRS hyperscanning studies since this is the method also employed by the authors for their own analyses. Especially EEG studies tend to look at quite different frequency ranges and therefore potentially rather distinct neurobiological processes and time windows. It may also be useful to mention a bit more in detail what some of the benefits and constraints of WTC analysis are (also, and particularly, in the context of fNIRS hyperscanning research).

8) Finally, also in the introduction, and/or in the discussion, it would be good if a slightly more extended discussion of fNIRS hyperscanning within the realm of relational neuroscience could be implemented (De Felice et al., 2025, Neuroscience & Biobehavioral Reviews). This includes considerations of interpersonal neural synchrony spanning from real-time joint experiences to long-term social bonds, current interpretations of the role of interpersonal neural synchrony for human social interactions and relationships as well as important questions pertaining to causality and mechanistic explanations. That said, I would like to commend the authors for not using any causal language and for consistently discussing their findings in a very careful and thoughtful manner.

I hope that my comments are useful and that they will help further improving an already very impressive and theoretically and methodologically strong report.

# Author comment: Author response to Decision Letter 2

**DOI:** 10.1371/journal.pbio.3003899.r003

30 Apr 2026

Attachment Submitted filename: ResponseToReviewers.pdf

# Peer review: Decision Letter 2

**DOI:** 10.1371/journal.pbio.3003899.r004

**Authors:** Christian Schnell, PhD

26 May 2026

Dear Ryssa,

Thank you for your patience while we considered your revised manuscript "Longitudinal intergenerational hyperscanning indexes changes in social connection" for consideration as a Short Reports at PLOS Biology. Your revised study has now been evaluated by the PLOS Biology editors, the Academic Editor and the original reviewers.

In light of the reviews, which you will find at the end of this email, we are pleased to offer you the opportunity to address the remaining points from Reviewer 1 in a revision that we anticipate should not take you very long. We will then assess your revised manuscript and your response to the reviewers' comments with our Academic Editor aiming to avoid further rounds of peer-review, although we might need to consult with the reviewers, depending on the nature of the revisions.

In addition to these revisions, you may need to complete some formatting changes, which you will receive in a follow up email. A member of our team will be in touch with a set of requests shortly. If you do not receive a separate email within a few days, please assume that checks have been completed, and no additional changes are required.

We expect to receive your revised manuscript within 1 month. Please email us (plosbiology@plos.org) if you have any questions or concerns, or would like to request an extension.

At this stage, your manuscript remains formally under active consideration at our journal; please notify us by email if you do not intend to submit a revision so that we withdraw the manuscript.

**IMPORTANT - SUBMITTING YOUR REVISION**

Your revisions should address the specific points made by each reviewer. Please submit the following files along with your revised manuscript:

1. A 'Response to Reviewers' file - this should detail your responses to the editorial requests, present a point-by-point response to all of the reviewers' comments, and indicate the changes made to the manuscript.

*NOTE: In your point-by-point response to the reviewers, please provide the full context of each review. Do not selectively quote paragraphs or sentences to reply to. The entire set of reviewer comments should be present in full and each specific point should be responded to individually.

You should also cite any additional relevant literature that has been published since the original submission and mention any additional citations in your response.

2. In addition to a clean copy of the manuscript, please also upload a 'track-changes' version of your manuscript that specifies the edits made. This should be uploaded as a "Revised Article with Changes Highlighted " file type.

*Resubmission Checklist*

When you are ready to resubmit your revised manuscript, please refer to this resubmission checklist: https://plos.io/Biology_Checklist

To submit a revised version of your manuscript, please go to https://www.editorialmanager.com/pbiology/ and log in as an Author. Click the link labelled 'Submissions Needing Revision' where you will find your submission record.

Please make sure to read the following important policies and guidelines while preparing your revision:

*Published Peer Review*

Please note while forming your response, if your article is accepted, you may have the opportunity to make the peer review history publicly available. The record will include editor decision letters (with reviews) and your responses to reviewer comments. If eligible, we will contact you to opt in or out. Please see here for more details:

https://blogs.plos.org/plos/2019/05/plos-journals-now-open-for-published-peer-review/

*PLOS Data Policy*

Please note that as a condition of publication PLOS' data policy (http://journals.plos.org/plosbiology/s/data-availability) requires that you make available all data used to draw the conclusions arrived at in your manuscript. If you have not already done so, you must include any data used in your manuscript either in appropriate repositories, within the body of the manuscript, or as supporting information (N.B. this includes any numerical values that were used to generate graphs, histograms etc.). For an example see here: http://www.plosbiology.org/article/info%3Adoi%2F10.1371%2Fjournal.pbio.1001908#s5

*Blot and Gel Data Policy*

We require the original, uncropped and minimally adjusted images supporting all blot and gel results reported in an article's figures or Supporting Information files. We will require these files before a manuscript can be accepted so please prepare them now, if you have not already uploaded them. Please carefully read our guidelines for how to prepare and upload this data: https://journals.plos.org/plosbiology/s/figures#loc-blot-and-gel-reporting-requirements

*Protocols deposition*

To enhance the reproducibility of your results, we recommend that if applicable you deposit your laboratory protocols in protocols.io, where a protocol can be assigned its own identifier (DOI) such that it can be cited independently in the future. Additionally, PLOS ONE offers an option for publishing peer-reviewed Lab Protocol articles, which describe protocols hosted on protocols.io. Read more information on sharing protocols at https://plos.org/protocols?utm_medium=editorial-email&utm_source=authorletters&utm_campaign=protocols

Thank you again for your submission to our journal. We hope that our editorial process has been constructive thus far, and we welcome your feedback at any time. Please don't hesitate to contact us if you have any questions or comments.

Sincerely,

Christian

Christian Schnell, PhD

Senior Editor

PLOS Biology

cschnell@plos.org

----------------------------------------------------------------

REVIEWS:

Reviewer #1: Thank you very much for the detailed and thoughtful responses to the previous round of reviews. The revision has clearly improved the transparency of the manuscript, particularly with respect to the statistical approach and the exploratory nature of the analyses. I appreciate the authors' efforts to clarify their methodological choices and to engage with the relevant statistical literature.

I also find it entirely acceptable that the authors frame their work as a fully exploratory, preregistered analysis. However, I remain concerned about the handling of the preregistered hypotheses and their relationship to the reported findings.

In the preregistration, the following hypothesis is explicitly stated:

"We hypothesise that interpersonal synchrony during collaboration, both behavioural and neural, will increase within dyads over repeated interactions. Further, we expect the increase to be greater in intergenerational dyads than same-generation dyads, as greater cognitive load might be incurred to overcome generational differences (Dikker et al., 2022)."

This hypothesis is clearly directional and mechanistically motivated, and it is not consistent with the reported findings (i.e., decreasing synchrony in intergenerational dyads). Thus, while the authors now characterize the study as exploratory, it is evident that there were prior expectations and theoretical considerations that guided the work.

In their response, the authors state that Supplementary Table 1 "summarizes the alignment of our preregistered predictions with our findings." However, such a summary is not, in fact, provided in a meaningful way. Most importantly, the fundamental discrepancy between the preregistered hypothesis and the observed results is not explicitly acknowledged or discussed anywhere in the revised manuscript.

I strongly encourage the authors to address this point directly in the main text. A transparent discussion of why the preregistered expectation was not supported, and how this informs the theoretical framework, would substantially strengthen the manuscript.

Relatedly, I thank the authors for their extensive references to the statistical literature and their clarification of the Bayesian estimation approach. However, I would appreciate further clarification on how this estimation-focused strategy aligns with the presence of clearly formulated (albeit exploratory) hypotheses in the preregistration. Specifically:

*If the goal is not to evaluate hypotheses, how should readers interpret the discrepancy between the preregistered expectations and the posterior estimates?

*How do the authors reconcile the existence of directional hypotheses with an analytical framework that explicitly avoids hypothesis testing?

Clarifying this conceptual link would improve the coherence between the study's theoretical motivation and its statistical implementation.

Finally, I appreciate the authors' efforts to better contextualize the magnitude of their findings. For example, the revised Results now state:

"Across sessions, feelings of loneliness showed small reductions for both groups (~1% per session), with no observed difference in rate of reduction between groups."

This is a welcome improvement. However, this more nuanced interpretation should also be consistently reflected in the Discussion. Currently, the manuscript states:

"Individuals' levels of loneliness and social closeness decreased and increased, respectively, across sessions, for both generational constellations. These findings match the target outcomes of the community programs proposed by policy makers, researchers, and health practitioners […] and indicate that social connections were indeed formed."

I encourage the authors to align the Discussion more closely with the quantified effect sizes and their practical significance.

Reviewer #2: The authors have addressed my previous questions and concerns sufficiently. I have no further comments.

Reviewer #3: Thank you very much for this thorough revision. I have no further comments.

# Author comment: Author response to Decision Letter 3

**DOI:** 10.1371/journal.pbio.3003899.r005

3 Jun 2026

Attachment Submitted filename: R2R.pdf

# Editor report: Decision Letter 3

**DOI:** 10.1371/journal.pbio.3003899.r006

**Authors:** Christian Schnell, PhD

12 Jun 2026

Dear Ryssa,

Thank you for your patience while we considered your revised manuscript "Longitudinal intergenerational hyperscanning indexes changes in social connection" for publication as a Short Reports at PLOS Biology. This revised version of your manuscript has been evaluated by the PLOS Biology editors and the Academic Editor.

Based on our Academic Editor's assessment of your revision, we are likely to accept this manuscript for publication, provided you satisfactorily address the following data and other policy-related requests:

* We would like to suggest a different title to improve its accessibility for our broad audience:

Longitudinal hyperscanning indexes changes in social connection in inter- and same-generation dyads

* DATA POLICY:

You may be aware of the PLOS Data Policy, which requires that all data be made available without restriction: http://journals.plos.org/plosbiology/s/data-availability. For more information, please also see this editorial: http://dx.doi.org/10.1371/journal.pbio.1001797

Note that we do not require all raw data. Rather, we ask that all individual quantitative observations that underlie the data summarized in the figures and results of your paper be made available in one of the following forms:

1) Supplementary files (e.g., excel). Please ensure that all data files are uploaded as 'Supporting Information' and are invariably referred to (in the manuscript, figure legends, and the Description field when uploading your files) using the following format verbatim: S1 Data, S2 Data, etc. Multiple panels of a single or even several figures can be included as multiple sheets in one excel file that is saved using exactly the following convention: S1_Data.xlsx (using an underscore).

2) Deposition in a publicly available repository. Please also provide the accession code or a reviewer link so that we may view your data before publication.

Regardless of the method selected, please ensure that you provide the individual numerical values that underlie the summary data displayed in the following figure panels as they are essential for readers to assess your analysis and to reproduce it: 1C and S3C.

NOTE: the numerical data provided should include all replicates AND the way in which the plotted mean and errors were derived (it should not present only the mean/average values).

Please also ensure that figure legends in your manuscript include information on where the underlying data can be found, and ensure your supplemental data file/s has a legend.

Please ensure that your Data Statement in the submission system accurately describes where your data can be found.

* CODE POLICY

Per journal policy, if you have generated any custom code during the course of this investigation, please make it available without restrictions. Please ensure that the code is sufficiently well documented and reusable, and that your Data Statement in the Editorial Manager submission system accurately describes where your code can be found. More information on our Code Policy, what and how to share can be found here: https://journals.plos.org/plosbiology/s/code-availability

Please note that we cannot accept sole deposition of code in GitHub, as this could be changed after publication. However, you can archive this version of your publicly available GitHub code to Zenodo. Once you do this, it will generate a DOI number, which you will need to provide in the Data Accessibility Statement (you are welcome to also provide the GitHub access information). See the process for doing this here: https://docs.github.com/en/repositories/archiving-a-github-repository/referencing-and-citing-content

As you address these items, please take this last chance to review your reference list to ensure that it is complete and correct. If you have cited papers that have been retracted, please include the rationale for doing so in the manuscript text, or remove these references and replace them with relevant current references. Any changes to the reference list should be mentioned in the cover letter that accompanies your revised manuscript.

In addition to these revisions, you may need to complete some formatting changes, which you will receive in a follow up email. A member of our team will be in touch with a set of requests shortly. If you do not receive a separate email within a few days, please assume that checks have been completed, and no additional changes are required.

We expect to receive your revised manuscript within two weeks.

To submit your revision, please go to https://www.editorialmanager.com/pbiology/ and log in as an Author. Click the link labelled 'Submissions Needing Revision' to find your submission record. Your revised submission must include the following:

- a cover letter that should detail your responses to any editorial requests, if applicable, and whether changes have been made to the reference list

- a Response to Reviewers file that provides a detailed response to the reviewers' comments (if applicable, if not applicable please do not delete your existing 'Response to Reviewers' file.)

- a track-changes file indicating any changes that you have made to the manuscript.

NOTE: If Supporting Information files are included with your article, note that these are not copyedited and will be published as they are submitted. Please ensure that these files are legible and of high quality (at least 300 dpi) in an easily accessible file format. For this reason, please be aware that any references listed in an SI file will not be indexed. For more information, see our Supporting Information guidelines:

https://journals.plos.org/plosbiology/s/supporting-information

*Published Peer Review History*

Please note that you may have the opportunity to make the peer review history publicly available. The record will include editor decision letters (with reviews) and your responses to reviewer comments. If eligible, we will contact you to opt in or out. Please see here for more details:

https://plos.org/published-peer-review-history/

*Press*

Should you, your institution's press office or the journal office choose to press release your paper, please ensure you have opted out of Early Article Posting on the submission form. We ask that you notify us as soon as possible if you or your institution is planning to press release the article.

*Protocols deposition*

To enhance the reproducibility of your results, we recommend that if applicable you deposit your laboratory protocols in protocols.io, where a protocol can be assigned its own identifier (DOI) such that it can be cited independently in the future. Additionally, PLOS ONE offers an option for publishing peer-reviewed Lab Protocol articles, which describe protocols hosted on protocols.io. Read more information on sharing protocols at https://plos.org/protocols?utm_medium=editorial-email&utm_source=authorletters&utm_campaign=protocols

Please do not hesitate to contact me should you have any questions.

Sincerely,

Christian

Christian Schnell, PhD

Senior Editor

cschnell@plos.org

PLOS Biology

# Editor report: Decision Letter 4

**DOI:** 10.1371/journal.pbio.3003899.r007

**Authors:** Christian Schnell, PhD

26 Jun 2026

Dear Ryssa,

Thank you for the submission of your revised Short Reports "Longitudinal hyperscanning indexes changes in social connection in intergenerational and same generation dyads" for publication in PLOS Biology. On behalf of my colleagues and the Academic Editor, Yina Ma, I am pleased to say that we can in principle accept your manuscript for publication, provided you address any remaining formatting and reporting issues. These will be detailed in an email you should receive within 2-3 business days from our colleagues in the journal operations team; no action is required from you until then. Please note that we will not be able to formally accept your manuscript and schedule it for publication until you have completed any requested changes.

As discussed, please update the references to the source data in the figure legends while you attend to those other requests to come.

Please take a minute to log into Editorial Manager at http://www.editorialmanager.com/pbiology/, click the "Update My Information" link at the top of the page, and update your user information to ensure an efficient production process.

PRESS

We frequently collaborate with press offices. If your institution or institutions have a press office, please notify them about your upcoming paper at this point, to enable them to help maximise its impact. If the press office is planning to promote your findings, we would be grateful if they could coordinate with biologypress@plos.org. If you have previously opted in to the early version process, we ask that you notify us immediately of any press plans so that we may opt out on your behalf.

We also ask that you take this opportunity to read our Embargo Policy regarding the discussion, promotion and media coverage of work that is yet to be published by PLOS. As your manuscript is not yet published, it is bound by the conditions of our Embargo Policy. Please be aware that this policy is in place both to ensure that any press coverage of your article is fully substantiated and to provide a direct link between such coverage and the published work. For full details of our Embargo Policy, please visit http://www.plos.org/about/media-inquiries/embargo-policy/.

Thank you again for choosing PLOS Biology for publication and supporting Open Access publishing. We look forward to publishing your study.

Sincerely,

Christian

Christian Schnell, PhD

Senior Editor

PLOS Biology

cschnell@plos.org
